# Mutual Diffusivities of Mixtures of Carbon Dioxide and Hydrogen and Their Solubilities in Brine: Insight from Molecular Simulations

**DOI:** 10.1021/acs.iecr.4c01078

**Published:** 2024-05-31

**Authors:** Thejas Hulikal Chakrapani, Hadi Hajibeygi, Othonas A. Moultos, Thijs J. H. Vlugt

**Affiliations:** † Reservoir Engineering, Geoscience and Engineering Department, Faculty of Civil Engineering and Geosciences, Delft University of Technology, Delft 2628 CN, The Netherlands; ‡ Engineering Thermodynamics, Process and Energy Department, Faculty of Mechanical Engineering, Delft University of Technology, Delft 2628 CB, The Netherlands

## Abstract

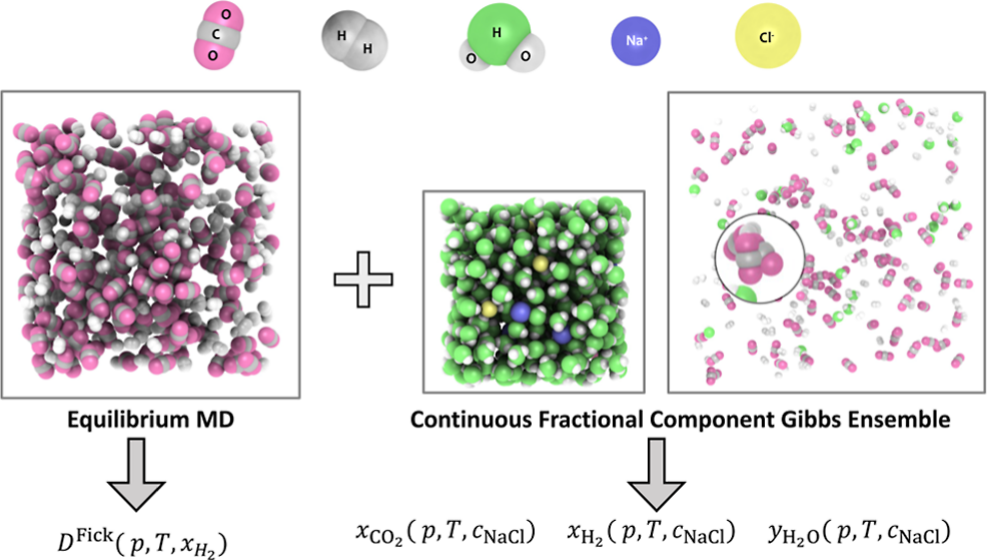

H_2_-CO_2_ mixtures find wide-ranging applications, including their growing significance as synthetic fuels in the transportation industry, relevance in capture technologies for carbon capture and storage, occurrence in subsurface storage of hydrogen, and hydrogenation of carbon dioxide to form hydrocarbons and alcohols. Here, we focus on the thermodynamic properties of H_2_-CO_2_ mixtures pertinent to underground hydrogen storage in depleted gas reservoirs. Molecular dynamics simulations are used to compute mutual (Fick) diffusivities for a wide range of pressures (5 to 50 MPa), temperatures (323.15 to 423.15 K), and mixture compositions (hydrogen mole fraction from 0 to 1). At 5 MPa, the computed mutual diffusivities agree within 5% with the kinetic theory of Chapman and Enskog at 423.15 K, albeit exhibiting deviations of up to 25% between 323.15 and 373.15 K. Even at 50 MPa, kinetic theory predictions match computed diffusivities within 15% for mixtures comprising over 80% H_2_ due to the ideal-gas-like behavior. In mixtures with higher concentrations of CO_2_, the Moggridge correlation emerges as a dependable substitute for the kinetic theory. Specifically, when the CO_2_ content reaches 50%, the Moggridge correlation achieves predictions within 10% of the computed Fick diffusivities. Phase equilibria of ternary mixtures involving CO_2_-H_2_-NaCl were explored using Gibbs Ensemble (GE) simulations with the Continuous Fractional Component Monte Carlo (CFCMC) technique. The computed solubilities of CO_2_ and H_2_ in NaCl brine increased with the fugacity of the respective component but decreased with NaCl concentration (salting out effect). While the solubility of CO_2_ in NaCl brine decreased in the ternary system compared to the binary CO_2_-NaCl brine system, the solubility of H_2_ in NaCl brine increased less in the ternary system compared to the binary H_2_-NaCl brine system. The cooperative effect of H_2_-CO_2_ enhances the H_2_ solubility while suppressing the CO_2_ solubility. The water content in the gas phase was found to be intermediate between H_2_-NaCl brine and CO_2_-NaCl brine systems. Our findings have implications for hydrogen storage and chemical technologies dealing with CO_2_-H_2_ mixtures, particularly where experimental data are lacking, emphasizing the need for reliable thermodynamic data on H_2_-CO_2_ mixtures.

## 1 Introduction

The need to meet growing global energy demands while controlling carbon emissions has brought underground hydrogen storage to the forefront.^1−4^ Given the intermittent production of renewable energy sources such as wind and solar, storing excess energy becomes essential to ensure consistent supply.^5^ Converting surplus energy into hydrogen by water electrolysis is a desirable method, given the appeal of hydrogen as an energy carrier: low carbon footprint and high mass-specific energy density.^1−4^ At present, worldwide hydrogen consumption stands at approximately 115 million metric tons (115 × 10^9^ kg) per year^3^ and is expected to increase 10-fold (to the gigatonnes per year scale) to accommodate the expanding global energy needs.^3^ Storing a gigatonne of hydrogen would necessitate immense storage volumes of the order of 10^11^ m^3^, even at significant pressures of 10 MPa and temperatures of 323.15 K.^3^ Only geological formations such as depleted gas reservoirs and salt caverns can offer such large volumes for hydrogen storage.^3^ Depleted gas reservoirs, used initially for natural gas storage, can also be used for hydrogen storage.^1−3,6^ A so-called cushion gas is injected into a gas reservoir to maintain its pressure,^2,3^ while brine (or salt water) is naturally present within the reservoir.^2,3^ Processes such as the mixing of hydrogen with the cushion gas via molecular diffusion, and its dissolution into brine can decrease the purity of the stored hydrogen.^1,3,6^ Thus, estimating the mutual diffusion coefficients characterizing gas mixing and the solubility of the resulting gas mixtures in brine is essential for enabling underground storage of hydrogen.^2,3^ Oldenburg and colleagues^7,8^ showed that using carbon dioxide (CO_2_) as a cushion gas facilitates efficient injection and production of natural gas due to its high compressibility at reservoir-relevant thermodynamic conditions. CO_2_ is opted as the cushion gas in our study due to the similarities between hydrogen and natural gas storage.^4^

### 1.1 Fick Diffusivities of CO_2_-H_2_ Mixtures

Mass transport in binary gas mixtures, in the absence of temperature and pressure gradients, is described by Fick’s law, which linearly relates the diffusive flux to the concentration gradient of a species.^9−11^ An alternative description of the same phenomenon can be formulated by the Maxwell–Stefan (MS) framework,^12^ which describes diffusive mass transport based on chemical-potential gradients. The proportionality constant in Fick’s law, denoted by the mutual or Fick diffusion coefficient,^10,11^ can exhibit a strong dependence on the temperature, pressure, and mixture composition.^10,11^ Analytic expressions, experimental measurements, semiempirical methods guided by theory, and numerical simulations have been used to calculate mutual diffusivities in binary gas mixtures.^11,13−15^ Chapman and Enskog^16−18^ independently derived an analytic expression to predict the mutual diffusion coefficients of binary gas mixtures by solving the Boltzmann transport equation in the dilute gas limit in a framework commonly referred to as the kinetic theory of gases.^14,16−18^ Kinetic theory assumes gases are made of monatomic, spherical, nonpolar particles that undergo elastic collisions, i.e., there are no strong molecular interactions between them. The prediction of diffusivities by Chapman and Enskog has been shown to be highly accurate in the dilute gas regime.^19−21^ At moderate to high pressures (less than tens of MPa^11^), the applicability of kinetic theory for dilute gases is in doubt due to its simplistic assumptions of intermolecular interactions.^11,17,18^ Many researchers have expanded kinetic theory to address scenarios involving dense gases,^14,17,22^ notably the work of van Beijeren and Ernst, who derived diffusion coefficients and other properties for moderately dense, multicomponent mixtures of hard spheres, known as the revised Enskog theory (RET).^22^ Jervell and Wilhelmsen^23^ recently presented an RET for multicomponent mixtures of Mie fluids (RET-Mie), which is claimed to accurately predict transport properties such as diffusion coefficients, thermal diffusivities, viscosities, and thermal conductivities. With parameters of the Mie-potentials fitted to equilibrium properties of the respective components, predictions of mutual diffusivities of noble gases for pressures ca. 0.1 MPa for a wide temperature range (200–1400 K) are within 4% of the experimental data.^24,25^ RET-Mie predictions of self-diffusivities of H_2_ are within 10% of the available experimental data up to 200 MPa and at temperatures between 171 and 372 K, and mutual diffusivities of noble gases are predicted within 4% of the experimentally determined values. Fick diffusivities of carbon dioxide-methane (CH_4_) mixtures are within 20% of experimental measurements and molecular simulations for pressures up to 14.7 MPa.^26^ Except for xenon in the vicinity of the critical point, the thermal conductivity of noble gases and their mixtures is reproduced within 10% of the experimental data at 300 K and pressures between 0.01 and 10 MPa. RET-Mie predictions of the viscosity are within ca. 10% of the experimental data for methane, nitrogen, and argon up to 300 bar for temperatures ranging between 233 and 523 K. At pressures up to 500 bar and temperatures from 200 to 800 K, the predictions are within ca. 15% of the most accurate correlation for the viscosity of air. Larger inaccuracies in the predictions of mutual diffusivities of CO_2_-CH_4_ mixtures reflect the inability of the theory to account for nonspherical shapes of molecules. In conclusion, kinetic theory is incapable of predicting the mutual diffusivities of CO_2_-H_2_ mixtures at reservoir-relevant thermodynamic conditions due to its simplistic assumptions. Advanced theories such as the RET-Mie (or RET) are cumbersome to use and still require significant efforts to account for nonspherical molecules.^14,23^

Marrero and Mason^19^ and Li et al.^27^ provide an extensive review of experimental data on the mutual diffusivities in binary mixtures of CO_2_ and H_2_, a summary of which can be found in Table 1. This table highlights the extensive literature containing data for a wide range of temperatures, albeit confined to pressures near the atmospheric pressure (0.1 MPa). Chou and Martin^28^ measured the self-diffusion coefficients of infinitely diluted Carbon-14 labeled C^14^O_2_ in ternary mixtures of C^14^O_2_, CO_2_, and H_2_ for temperatures of 308.15 and 373.15 K, and pressures of 0.6 and 25.33 MPa. Literature covering experimental studies of trace solutes in dense gases, such as supercritical CO_2_, has been summarized by Poling et al.^13^ To the best of our knowledge, experimental data for the Fick diffusivities of binary mixtures of CO_2_ and H_2_ at pressures exceeding the atmospheric pressure have not been reported.

**Table 1:** List of Experimental Studies Reporting Diffusion Coefficients of Binary Mixtures of Hydrogen and Carbon Dioxide^a^

study	temperature/[K]	experimental method^11,19^
Loschmidt^141^	273 and 286	closed tube
Loschmidt^142^	273–289	closed tube
Schmidt^143^	288	closed tube
Lonius^144^	286–294	closed tube
Boardman and Wild^145^	288	closed tube
Suetin^146^	273	closed tube
Wrestschko	297	closed tube
Suetin and Ivakin^147^	292	closed tube
Ivakin and Suetin^148^	292–473	closed tube
von Obermayer^149^	285–335	open tube
von Obermayer^150^	284–293	open tube
von Obermayer^151^	280–294	open tube
Schäfer et al.^152^	252–308	two-bulb
Lonsdale and Mason^153^	259–358	two bulb
Saxena and Mason^136^	250–368	two bulb
Bondarenko and Golubev^154^	323–363	two bulb
Miller and Carman^155^	293	two bulb
Annis et al.^156^	295	two bulb
Miller and Carman^157^	293	two bulb
Waldmann^158,159^	293	Dufour effect
Mason et al.^160^	296	Dufour effect
Weissman^137^	298–500	from mixture viscosities
Kestin et al.^161^	295.15, 300.65 and 303.15	from mixture viscosities
Wicke and Hugo^162^	295	diffusion bridge
Schneider and Schäfer^163^	273–990	diffusion bridge
Kosov and Zhalgasov^164^	196–298	diffusion bridge
Giddings and Seager^165^	300	gas chromatography
Gavril et al.^135^	315.2–343.9	reverse liquid gas chromatography
Boyd et al.^168^	298	thermal separation
Vyshenskaya and Kosov^166^	293–1083	capillary leak
McCarty and Mason^167^	303	Kirkendall effect

a The pressures in the various studies are all close to atmospheric pressure. A detailed discussion of the experimental methods can be found in the article by Marrero and Mason^19^ and the textbook by Cussler^11^ and in references therein.

To facilitate rapid and precise predictions of Fick diffusivities in gas mixtures, numerous semiempirical expressions guided by the kinetic theory have been developed.^11,13^ These expressions aim to fill the gap resulting from the scarcity of experimental measurements, specifically for predicting binary diffusion coefficients in both dilute and dense gases.^13^ Correlations by Wilke-Lee^29^ and Fuller et al.^30−32^ are often used to predict diffusion coefficients in dilute gas mixtures. These correlations fail at high pressures,^11,13^ just as Chapman and Enskog’s analytic expression for mutual diffusivities, and cannot take into account the compositional dependence of the mixture. Several methods have been proposed to estimate diffusion coefficients in dense gas mixtures.^13^ Takahashi and Hongo^33^ and Riazi and Whitson^34^ suggested empirical equations based on the method of corresponding states to correlate the product of pressure and diffusion coefficient at low and high pressures.^13^ Takahashi and Hongo’s correlation for the effect of pressure and temperature on the binary diffusion coefficient requires the evaluation of an empirical equation using look-up tables that are tabulated as functions of the critical temperature and pressure of the components in the mixture.^13^ Riazi and Whitson’s correlation requires the viscosity of the mixture as an input and does not yield the correct value of mutual diffusivities in the limit of low pressure.^13^ No correlation is entirely satisfactory for predicting mutual diffusivities at both low and high pressures, primarily due to the simplistic assumptions of the theory of corresponding states.^13^ Although semiempirical methods are relatively easy to use and computationally fast, their accuracy depends on the extent and quality of the experimental data that was used for their development. Generally, one needs to exercise caution when using semiempirical methods outside the range used for its calibration.

Predictions of Fick diffusivities for binary mixtures of CO_2_-H_2_ using semiempirical methods can be accurate between low to intermediate pressures but can be inaccurate due to neglecting strong molecular interactions emanating at large pressures (≳10 MPa). Molecular dynamics (MD) simulations, a potent tool in materials research,^35,36^ operate at atomistic length and time scales, offering insights at the molecular-scale.^35,36^ The formulation of accurate force fields for multiple components, the design of efficient algorithms, coupled with advancements in computational power, has made MD a go-to method for calculating the diffusivities of pure components, mixtures, and other transport properties of fluids.^35,36^ The data obtained from MD simulations has been used for the development of engineering models and the validation of semiempirical approaches.^37−39^ Mutual diffusivities in binary and ternary fluid mixtures have been computed using MD simulations and have been successfully validated with experimental data.^40−42^ In sharp contrast, the Fick diffusivities of binary gas mixtures computed from MD simulations have not received much attention. Vella^43^ computed Fick diffusivities for CO_2_-CH_4_ mixtures using MD simulations at temperatures and pressures corresponding to the dilute and dense regimes. The computed diffusivities (within statistical uncertainties), described faithfully by Fuller’s correlation,^30−32^ agreed with the experimental data between 293 and 500 K and 1.01325 MPa (dilute gas regime). Deviations exceeding 40% were observed between Fuller’s correlation and the computed diffusivities at ca. 310 K and pressures above ca. 2 MPa (the dense gas regime). Due to the lack of experimental data in the dense gas regime, Vella^43^ was unable to confirm the accuracy of the Fick diffusivities computed by MD simulations. Guevara-Carrion^26^ compared the measured Fick diffusivities of very dilute CH_4_ in supercritical CO_2_ at temperatures of 292.55 and 332.85 K and pressures of 9, 12.5, and 14.7 MPa. The mutual diffusivities changed from liquid-like to gas-like values within a small temperature range near the supercritical region across the so-called Widom line.^26^ Saric et al.^44^ measured the mutual diffusivities of binary mixtures of H_2_ diluted in CO_2_, along with other binary gas mixtures. Three mole fractions of H_2_, representing the infinite-dilution limit (mostly 0.5, 1.0, and 1.5 mol %), were examined between 290 and 350 K at 9 MPa. Saric et al.^44^ proposed a simple equation to predict the Widom line of dilute supercritical CO_2_ mixtures as a function of pressure, solute mole fraction, and critical properties of the mixture components. In a separate study, Saric et al.^45^ examined the mutual diffusion of various hydrocarbons dispersed in supercritical carbon dioxide via MD simulations near the Widom line, between 290 and 345 K at 9 MPa. Fick diffusivities were strongly affected close to the critical region of the mixture due to the pronounced clustering of solute molecules. Saric et al.^45^ pointed out that the validity of the Stokes–Einstein relation near the Widom line was questionable. A correlation function was proposed to facilitate quick calculations of Fick diffusivities for various binary mixtures.

Rosenfeld^46^ proposed an empirical correlation connecting transport properties to the excess entropy of a fluid at a given temperature and pressure. This semiquantitative model has garnered renewed interest since the early 2000s,^47−50^ demonstrating its broader applicability beyond simple liquids.^49−51^ Despite its nonrigorous nature, the concept of excess entropy could serve as a valuable tool in connecting thermodynamics and dynamics for predicting transport properties in mixtures.^47,48^

To summarize, neither experimental data nor MD simulations have reported the Fick diffusivities of CO_2_-H_2_ mixtures under reservoir conditions. Analytic expressions can be cumbersome and require effort to include nonspherical molecules.^14,17,23^ Semiempirical methods, though easy to use, are not accurate for the entire range of thermodynamic conditions of interest.^11,13^

### 1.2 Vapor–Liquid Equilibria for Systems Involving H_2_, CO_2_, and NaCl Brine

#### 1.2.1 CO_2_-NaCl Brine Systems

Understanding the dissolution of H_2_ in saline water, referred to as brine, at various thermodynamic conditions is necessary for estimating hydrogen purity during subsurface storage. This involves predicting solubilities by assessing equilibrium between vapor and liquid phases in a multicomponent system at different temperatures and pressures, known as vapor–liquid equilibria (VLE).^13,52,53^ VLE of CO_2_-NaCl brine (or H_2_-NaCl brine) is determined via experiments, equation-of-state models, and force field-based molecular simulations.^54−67^

Studies by Spycher et al.^54,55^ and Mutailipu provide an overview of the VLE of various CO_2_-brine systems compiled from experimental studies. Spycher et al.^54^ compared data on mutual solubilities in CO_2_-H_2_O mixtures from various experiments for pressures up to 50 MPa and temperatures below 373.15 K. Solubilities measured in the two-phase region, where a CO_2_-rich phase (usually gas) coexists with a H_2_O-rich liquid phase, were consistent across various studies.^54^ A thermodynamic model was developed by Spycher et al.^54^ based on the Redlich–Kwong equation-of-state to enable quick predictions of mutual solubilities in CO_2_-H_2_O systems. This model predicts mutual solubilities with an accuracy of a few percent of the experimental data for pressures up to 60 MPa and a temperature ca. 383.15 K. In a subsequent study, Spycher et al.^55^ extended the thermodynamic model developed initially for CO_2_-H_2_O mixtures^54^ to include the influence of chloride salts dissolved in the aqueous phase. The extended thermodynamic model to predict mutual solubilities included activity coefficients for aqueous CO_2_ derived from studies developed primarily for sodium chloride (NaCl). The activity coefficients of NaCl provided by Duan and Sun^56^ and Rumpf et al.^68^ were shown to yield CO_2_ solubilities in NaCl brine that were closest to the experiments by Duan and Sun,^56^ Drummond,^69^ Malinin and Saveleva,^70^ Malinin and Kurovskaya,^71^ Rumpf et al.,^68^ Nighswander et al.,^72^ Bando et al.,^73^ and Kiepe et al.,^74^ between pressures 3.5 and 6.8 MPa and between 293.15 K and 373.15 K. The extended solubility model provided by Spycher and Pruess,^55^ including the effect of salts, was shown to be accurate to within the experimental uncertainty for solutions up to 6 molal (= moles of salt per kg of water) for NaCl and 4 molal for CaCl_2_. Spycher and Pruess^75^ extended their solubility model^55^ to cover temperatures up to 573 K (from 285 K) and pressures below 60 MPa.

Molecular simulations have been extensively applied to investigate VLE in CO_2_-H_2_O and CO_2_-NaCl brine systems. Vorholz et al.^63^ studied the VLE of H_2_O between 323 and 573 K, CO_2_ between 230 and 290 K, and CO_2_-H_2_O mixtures between 348 and 393 K using *NVT* and *NPT* versions of Gibbs Ensemble Monte Carlo^35,36^ (GEMC) simulations. Three different H_2_O force fields, namely SPC, SPC-E, and TIP4P, the EPM2 force field for CO_2_, and combinations thereof were examined to find the optimal VLE description for binary mixtures of CO_2_-H_2_O. The best prediction for the VLE of CO_2_-H_2_O binary mixtures, up to pressures of 20 MPa, was achieved by combining the SPC and TIP4P force fields for water with the EPM2 force field for carbon dioxide. Liu et al.^60^ used histogram-reweighting Grand-Canonical Monte Carlo (GCMC)^35,36^ simulations to obtain the phase behavior of CO_2_-H_2_O mixtures for 323.15 and 723.15 K, and 0 and 100 MPa. Several different force fields for H_2_O and CO_2_ were tested using the conventional Lorentz–Berthelot mixing rules^35,36^ for the interaction between unlike atoms. Experimentally measured solubilities deviated from solubilities computed using force field based simulations for the entire range of temperatures and pressures spanning 323.15 to 723.15 K and 0 to 100 MPa, respectively. The work of Liu et al.^61^ suggests the need for improved atomistic force fields to accurately predict the VLE of CO_2_-H_2_O systems. Liu et al.^61^ investigated the phase behavior and interfacial tension of CO_2_-H_2_O-NaCl mixtures using MD simulations between 323.15 and 523.15 K, 0–60 MPa, and NaCl concentrations 1–4 molal. TraPPE^76,77^/TIP4P2005^78^ performed the most effectively among various combinations of force fields evaluated for predicting the VLE of the H_2_O-CO_2_-NaCl system. The dependence of the interfacial tension on temperature, pressure, and salt concentration was also examined, revealing insights into the predictive capabilities of different force field combinations. Orozco et al.^62^ conducted *NPT* GEMC simulations to obtain optimized intermolecular potential parameters to describe the phase behavior of CO_2_-H_2_O mixtures. The simulations covered a temperature range of 423–523 K and pressures from 20 to 80 MPa, addressing conditions pertinent to carbon capture and sequestration processes. Lobanova et al.^64^ simulated the VLE for CO_2_-H_2_O systems at 423 and 548 K using the SAFT-CG Mie force-fields for CO_2_ and H_2_O. The solubilities agreed well with various experimental data sources, thus allowing for an accurate description of CO_2_-H_2_O mixtures. Using GEMC simulations, Jiang et al.^65^ explored the phase equilibria of H_2_O/CO_2_ and H_2_O/*n*-alkane mixtures between 323 and 523 K and 20 and 80 MPa. CO_2_ solubility in water was computed for combinations of three polarizable models for water and a polarizable Gaussian charge model for CO_2_. Accurate compositions for both H_2_O and CO_2_-rich phases were achieved using hydrogen bonding parameters derived from the second virial coefficient for H_2_O-CO_2_ mixtures.

#### 1.2.2 H_2_-NaCl Brine Systems

A summary of H_2_ solubilities in pure H_2_O and brine can be found in the works by Chabab et al.,^57,59^ Ansari,^58^ and Torín-Ollarves and Trusler.^79^ Recognizing the lack of data for pressures exceeding 0.1 MPa, Chabab et al.^57^ conducted experiments to measure the solubility of H_2_ in brine, specifically NaCl brine, with molality ranging from 0 to 6 molal. Experiments were conducted between 323 and 373 K and 0.1–23 MPa. To enable quick and accurate predictions of H_2_ solubilities in brine as functions of temperature, pressure, and molality, the e-PR-CPA^80^ (electrolyte-Peng–Robinson^81^-Cubic Plus Association^82−84^) model was used, which was parametrized using the H_2_ solubilities from experiments. Torín-Ollarves and Trusler^79^ reported H_2_ solubilities in NaCl brine at a salt concentration of 2.5 molal between 323.15 and 423.15 K, up to pressures of 40 MPa. Following Krichevsky and Kasarnovsky,^85^ Torín-Ollarves and Trusler^79^ developed a correlation describing the solubility of H_2_ in H_2_O and NaCl brines for temperatures between 273.15 and 423.15 K and for pressures up to 100 MPa. Although the solubilities of H_2_ in salt-free water, as reported by Chabab et al.,^57^ conformed with the thermodynamic model proposed by Torín-Ollarves and Trusler,^79^ the solubilities of H_2_ in NaCl brine (up to 5 molal) were underestimated by approximately 25% when compared to an extended Krichevsky-Kasarnovsky model.^85^ This discrepancy was observed at temperatures of 323.2 and 372.7 K, considering H_2_ partial pressures up to 20 MPa. In a recent study, Chabab et al.^59^ addressed inconsistencies in H_2_ solubilities in NaCl brine for different experimental sources. These authors contributed new solubility data for H_2_ in NaCl brine, covering the temperature range of 298 to 373 K, with a NaCl concentration of 4 molal and pressures up to 20 MPa. The H_2_ solubilities measured in the work by Chabab^59^ are between those measured in an earlier study by Chabab et al.^57^ and Torín-Ollarves and Trusler.^79^ The H_2_ solubility data were used to parametrize and assess three different thermodynamic models: (1) the e-NRTL activity coefficient model,^86,87^ (2) the Duan-type (noniterative) model,^88,89^ and (3) the Søreide and Whitson’s equation of state.^90^ These models were shown to be highly accurate in predicting the phase equilibria of H_2_-NaCl brine systems, with an average absolute deviation of less than 3%.^59^

H_2_ solubilities in H_2_O and NaCl brine have also been studied using molecular simulations.^66,91^ Rahbari et al.^66^ reported H_2_ solubilities in H_2_O calculated from Monte Carlo simulations for pressures between 1 and 100 MPa and temperatures between 283 and 423 K. The H_2_ solubilities calculated using molecular simulations were in excellent agreement with the experimental data at high pressures by Wiebe and Gaddy.^92^ In another article, Rahbari et al.^93^ studied the VLE of hydrogen-water mixtures along the melting line of ice Ih, corresponding to temperatures between 264.21 and 272.4 K. Based on the computed solubility data of H_2_ in H_2_O, the freezing point depression of water caused by NaCl was calculated for temperatures between 264.21 and 272.4 K. Lopez-Lazaro et al.^94^ computed the solubilities of H_2_ in aqueous NaCl solutions using molecular simulations for molalities up to a maximum of 2 molal between temperatures of 280 and 350 K. Recently, van Rooijen et al.^95^ reported H_2_ solubilities in aqueous NaCl solutions for temperatures between 298 and 363 K, pressures ranging between 0.1 and 100 MPa, and molalities between 0 and 6 molal, together with other thermodynamic properties such as interfacial tensions and transport properties of H_2_-H_2_O-NaCl systems. The H_2_ concentrations in NaCl brine, calculated using molecular simulations,^95^ are larger compared to the predictions by Chabab et al.^57^ and lower than the prediction by Torín-Ollarves and Trusler^79^ for NaCl molalities larger than 0.5 molal. Despite numerous studies exploring solubilities in CO_2_-NaCl brine and H_2_-NaCl brine systems for diverse temperatures and pressures, there is a lack of data regarding solubilities in CO_2_-H_2_-NaCl brine systems.

Recognizing the lack of information on binary Fick diffusivities in CO_2_-H_2_ mixtures at high pressures, temperatures, and various compositions and the limited understanding of the VLE in ternary systems of CO_2_-H_2_-NaCl brine, molecular simulations are performed to address this gap. This study investigates the Fick diffusion coefficients computed from equilibrium MD simulations of H_2_-CO_2_ mixtures. Driven by the thermodynamic conditions relevant to subsurface hydrogen storage, simulations are performed covering temperatures between 323.15 and 423.15 K, 5–50 MPa, and mole fractions of hydrogen (*x*_H_2__) varying from 0 to 1. Solubilities of H_2_-CO_2_ mixtures in pure water and NaCl brine at concentrations of 1 and 2 mol of NaCl per kg water are calculated using the isothermal–isobaric version of the Continuous Fractional Component Gibbs Ensemble (CFCGE) Monte Carlo technique.^96−100^ Temperatures between 323.15 and 423.15 K and pressures between 5 and 50 MPa are explored. We only examine ternary mixtures where the overall mole fractions of CO_2_, H_2_, and Brine, calculated using the total number of molecules in both the gas and liquid phases, are 1/6, 1/6, and 2/3, respectively.

This paper is organized as follows: Section 2 provides details on the force fields and molecular simulation techniques. In Section 3, the self-diffusivities of H_2_ and CO_2_ are compared, along with the mutual diffusivities and solubilities obtained via molecular simulations. Comparisons to available experimental data and analytic expressions are included. Concluding remarks are presented in Section 4. All computed raw data from this study are tabulated in the Supporting Information.

## 2 Methods

### 2.1 Force Fields

The TraPPE force field is used to simulate CO_2_ as a rigid linear molecule.^76,77^ TraPPE compares favorably with experimental data for the VLE of pure CO_2_ and its multicomponent mixtures for a wide range of temperatures, pressures, and compositions.^76,77^ The three-site Marx force field^101,102^ is used to simulate H_2_ as a rigid body for its accuracy in reproducing the bulk densities and fugacities of H_2_ at pressures up to 100 MPa.^66,103^ Quantum effects emanating at low temperatures are insignificant for the temperatures considered in this work (323.15–423.15 K).^104,105^ Water is simulated as a rigid body using the TIP4P/μ force field, a modified version of the TIP4*P*/2005 model^78^ developed by Rahbari and colleagues.^66^ The TIP4P/μ force field accurately predicts the VLE of H_2_O-H_2_ mixtures between 323 and 423 K and 10–100 MPa^66^ as it was partially trained to reproduce the excess chemical potential of liquid water. The Madrid-2019 force field^106^ is used to model the Na^+^ and Cl^–^ ions, which are parametrized for the TIP4*P*/2005 H_2_O force field.^78^ Lennard-Jones (LJ) and electrostatic interactions are calculated for all species. All interaction parameters used in this study are listed in Table S1 of the Supporting Information. The Lorentz–Berthelot (LB) mixing rules^35,36^ are applied in most cases barring a few exceptions, see Table S1 of the Supporting Information.

### 2.2 Molecular Dynamics

MD simulations were performed using the Large-scale Atomic/Molecular Massively Parallel Simulator (LAMMPS)^107^ (version 23 June 2022) to calculate the self- and Fick diffusivities in CO_2_-H_2_ mixtures. Pressures between 5 and 50 MPa in 5 MPa increments, temperatures between 323.15 and 423.15 K in 25 K increments, and hydrogen mole fractions between 0 and 1 in 0.1 increments were explored. At these thermodynamic conditions, H_2_-CO_2_ mixtures exhibit either gas- or dense-gas-like behavior.^108^ Simulation boxes are periodic in all three directions. Equations of motion are integrated using the velocity-Verlet algorithm using a time step of 0.5 fs. Lennard-Jones and long-range electrostatic molecular interactions are calculated with a cutoff radius of 10 Å, supplemented by tail corrections for energy and pressure. All long-range electrostatic interactions are computed using the Particle–Particle Particle-Mesh method with a relative precision of 10^–6^. Neighbor lists for every molecule are generated each time step using a binning procedure by considering a radius equal to the force cutoff of 10 Å plus an additional “skin” of thickness 3 Å.

Transport properties such as viscosities, self-, MS, and Fick diffusivities are computed in the *NVE* ensemble using systems of 120 molecules. Small systems of 120 molecules are chosen to manage the high computational demands associated with the gas phase simulations for the calculation of (self- and MS) diffusivities for trajectories typically lasting 100 ns. Accurate computation of transport properties requires correction for finite-size effects, a topic addressed in Section 2.2.1. To ensure that simulations in the *NVE* ensemble yield the correct average pressure and temperature, the box sizes and total system energy (in kJ·mol^–1^) are determined from independent simulations of 2000 molecules. Small systems of 120 molecules are unsuitable for computations of densities due to the possibility of the interaction cutoff radii exceeding half of the box size, violating the nearest image convention. Long simulations in the *NVT* phase are necessary to obtain an average temperature due to the substantial fluctuations resulting from the small system size. The procedure and the raw data of the simulations for the larger systems are described and tabulated in Section S1.1 and Table S2 of the Supporting Information.

#### 2.2.1 Calculation of Transport Properties

Viscosities describe momentum transport,^109^ while self-, MS, and Fick diffusivities elucidate mass transport.^10,109,110^ Self-diffusion of a molecule in a fluid is caused by Brownian motion and occurs irrespective of gradients in chemical potential, temperature, or pressure.^110^ In contrast, MS and Fick diffusion^10,109^ describe the collective motion of molecules in response to gradients in chemical potential and concentration, respectively. Self-, MS, and Fick diffusion all contribute to mass transport via random or directed molecular motion, as explained in various texts.^10,109,110^ Viscosities, self-, MS, and Fick diffusivities are calculated in the *NVE* ensemble. The simulation box size corresponds to the density determined from systems of 2000 molecules, and the (scaled) total energy is imposed to obtain the desired temperature. Simulations consist of an initialization phase lasting 1 ns to achieve equilibration, followed by a production phase lasting between ca. 30–150 ns. The duration of the production phase varies widely due to density variations, affecting computational demands. Simulations at higher densities (e.g., systems at 50 MPa and 323.15 K) are computationally cheaper than those at lower densities (e.g., systems at 5 MPa and 423.15 K). During the production phase, the OCTP plugin^111^ in LAMMPS^107^ is used to compute the shear viscosities and self- and MS diffusivities based on mean-squared displacements. A discussion of the equations and methods to compute transport properties in the OCTP plugin can be found in the article by Jamali et al.^111^ Calculation of Fick diffusivities from MS diffusivities requires the so-called thermodynamic factors of diffusion.^10,11,40−42,112,113^ Recently, Vlugt and co-workers^114^ showed that the thermodynamic factors (Γ) computed using the CFCMC technique^96−100,115^ for various CO_2_-H_2_ mixtures at 323.15 K and pressures of 5 and 50 MPa agreed within 3% of the REFPROP data.^108^ Thus, for convenience and efficiency, thermodynamic factors for H_2_-CO_2_ mixtures are computed by numerically differentiating the Margules activity coefficient model based on the excess Gibbs energies obtained from REFPROP,^108^ as discussed in Section S5. To obtain accurate values of transport properties, an average value is calculated over 5 independent simulations at each thermodynamic state point. Uncertainties in the computed transport properties are calculated as the standard deviation across these 5 independent simulations.

While viscosities are usually unaffected by finite-size effects,^116,117^ self-, MS, and Fick diffusivities can depend on the system size,^116,118,119^ particularly in dense systems.^117^ The finite-size correction to the self-diffusion coefficient proposed by Yeh and Hummer^118,120^ is
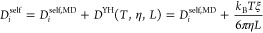
1where *D*_*i*_^self^ is the finite-size corrected self-diffusivity of the *i*th species, ξ is a dimensionless constant equal to 2.837298, and *L* is the box length of a cubic simulation box. Jamali et al.^116^ showed that the MS diffusion coefficients can be corrected according to
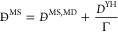
2where *D̵*^MS^ is the finite-size corrected MS diffusivity, *D*^YH^ is the Yeh–Hummer correction term in eq 1,^118^ and Γ is the thermodynamic factor for diffusion. For ideal mixtures where cross-correlations between molecular displacements of different species are negligible, the MS diffusivities follow the Darken equation^11,116,121,122^
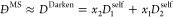
3

Taking advantage of the relation between *D̵*^MS^ and *D*^Fick^,^12^ Jamali et al.^116^ showed that the correction for the Fick diffusivity follows^116,118^
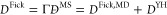
4

### 2.3 Monte Carlo Simulations

VLEs of CO_2_-NaCl brine mixtures, H_2_-NaCl brine mixtures, and CO_2_-H_2_-NaCl brine mixtures are determined via MC simulations performed using the open-source Brick-CFCMC software.^98,100^ The focus is on thermodynamic conditions relevant to subsurface storage of H_2_: pressures between 5 and 50 MPa, temperatures between 323.15 and 423.15 K, and NaCl concentrations varying between 0 and 2 molal. The reaction equilibria of CO_2_-H_2_O systems are not considered as bicarbonate concentrations are low.^123^

The CFCMC method is based on an expanded ensemble and uses a coupling parameter λ to incrementally insert or remove molecules, leading to improved insertion/removal efficiency compared to single-step insertions and deletions.^96−100,115^ Simulations are performed using the *NPT* version of the CFCGE technique, developed by Poursaeidesfahani et al.,^115^ to determine the VLEs of mixtures consisting of H_2_, CO_2_, and NaCl brine. A single fractional molecule per component *i*, where *i* can be either CO_2_, H_2_O, or H_2_, but not NaCl, is introduced in the simulations that is distinguishable from the whole molecules.^115^ Fractional molecules can be located in either of the two simulation boxes. The Lennard-Jones and electrostatic interactions of the fractional molecule with surrounding molecules are modulated by the coupling parameter λ_*i*_ ∈ [0, 1]. At λ_*i*_ = 0, the interactions resemble those of an ideal gas, while at λ_*i*_ = 1, interactions are fully scaled like those of a whole molecule. For the exact expressions of the scaled interactions the reader is referred to articles by Hens et al.^98^ and Polat et al.^100^ The details of the trial moves relevant for the CFCGE method: (1) molecule translations, (2) molecule rotations, (3) volume changes, and (4) hybrid insertions/deletions, are provided in the articles by Hens et al.,^98^ Polat et al.,^100^ and Rahbari et al.^99^ To ensure that all values of λ_*i*_ are sampled in both simulation boxes, a weight function or a biasing function *W*(λ_*i*_, *j*) is introduced for species *i* in box *j*, where *j* = 1 or 2.^98,100,115^ The excess chemical potentials of each species *i* in box *j* in the *NPT* ensemble (μ_*i*,*j*_^Ex^) equals^66,93,98,100,115^
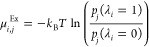
5where *p*_*j*_(λ_*i*_) is the probability that a fractional molecule of species *i* samples a value of λ_*i*_ in box *j*, *k*_B_ is the Boltzmann constant, and *T* is the absolute temperature. In addition to assessing equilibrium between systems, the excess chemical potential aids in determining fugacity coefficients, crucial for describing phase-coexistence in mixtures,^52,53^ particularly used in equation of state-based thermodynamic modeling for phase equilibria. Fugacity coefficients of species *i* (ϕ_*i*_) are directly related to its excess chemical potential in the *NPT* ensemble as^66,93^
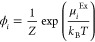
6where  is the compressibility factor for a mixture in the gas phase whose average volume equals ⟨*V*⟩. For further details of the CFC method the reader is referred to the articles by Hens et al.^98^ and Polat et al.^100^

In the systems of H_2_-NaCl brine and CO_2_-NaCl brine, 200 molecules of H_2_ (or CO_2_) in the gas-rich phase and 400 molecules of H_2_O in the liquid-rich phase are initialized. For the ternary system of H_2_-CO_2_-NaCl, 100 molecules each of H_2_ and CO_2_ are initialized in the gas-rich phase, and 400 molecules of H_2_O is initialized in the liquid-rich phase. 0, 7, and 15 ions of Na^+^ and Cl^–^, corresponding to NaCl concentrations of 0, 1, and 2 molal, are distributed randomly inside the H_2_O-rich simulation box. A cutoff radius of 10 Å is applied for the LJ interactions, and analytic tail corrections are applied. Electrostatic energies are computed using the Ewald summation^124^ with a real-space cutoff of 10 Å and a damping parameter α = 0.32 Å^–1^ for both simulation boxes. The number of *k*-vectors in Fourier space is *k* = 8 in the liquid-rich box. To ensure convergence of electrostatic energies in the expansive gas-phase boxes, the maximum *k*-vectors are varied between 9 and 19. The relation between the real-space cutoff length, damping parameter α, and the maximum value of the *k* vectors is provided in the manual of Brick-CFCMC.^98−100^ These settings ensure that the electrostatic energies are computed with a relative precision of 10^–6^.

Every cycle of a CFCGE simulation contains *N*_Total_ Monte Carlo (MC) trial moves, where *N*_Total_ is the total number of molecules. In the binary and ternary systems, molecule translations, rotations, and volume changes are selected with probabilities of 0.35, 0.24, and 0.01, respectively. Volume changes are computationally expensive since all configurational energies need recalculation based on the updated intermolecular distances after rescaling the simulation box. Consequently, volume changes are typically executed with a probability of 0.01.^98,100^ Changes of λ_*i*_ and molecule transfer moves (swapping and identity changes) are selected with probabilities of 0.2 each. The swapping of a fractional molecule of species *i* between boxes is attempted if λ_*i*_ < 0.3, and the identity change of the fractional molecules is attempted only if λ_*i*_ > 0.7.^115^ The maximum displacements, rotations, volume changes, and λ changes are adjusted to obtain acceptance of ca. 50%. Molecule transfers involving salt ions Na^+^ and Cl^–^ are excluded. For all simulations, an initialization phase lasting 10^4^ cycles is simulated to remove overlaps between molecules. In an equilibration phase lasting 15 × 10^6^ cycles, the weight functions *W*(λ_*i*_, *j*) are developed in each box, and a production phase lasting 10^6^ cycles is used to calculate solubilities and fugacity coefficients (see eq 6). To obtain accurate values of solubilities and fugacity coefficients, 40 independent simulations are performed at each thermodynamic state point. The uncertainties (error bars) in the computation of solubilities and fugacity coefficients are estimated by dividing the 40 independent simulations into blocks of 5.^36^ The solubilities of H_2_ and CO_2_ are determined as the ratio of the number of gas molecules to the total molecules in the liquid phase, with sodium and chloride ions considered separately. Liquid solubility in the gas phase is determined by the fraction of water molecules in the gas-rich phase.

## 3 Results and Discussion

### 3.1 Densities and Compressibilities of CO_2_-H_2_ Mixtures

**Figure 1:**
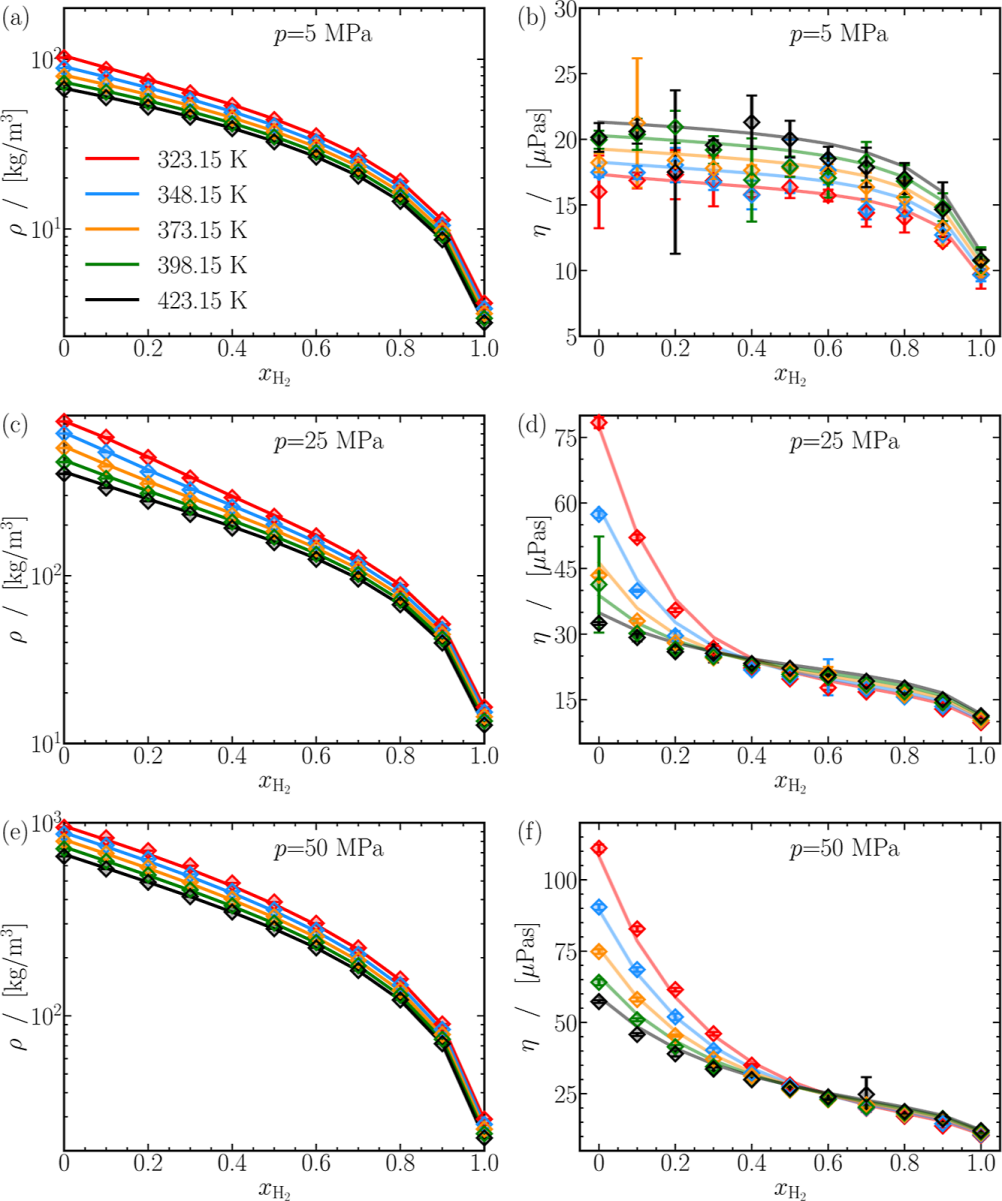
H_2_-CO_2_ mixture densities (ρ) and viscosities (η) at various pressures and temperatures. Computed properties (symbols) are compared to the REFPROP^108^ data (solid lines). Uncertainties in simulation results are smaller than the symbol sizes. Lines are colored as per the legend in subfigure (a). Raw data on the densities and viscosities for all mixtures are provided in Tables S2 and S3 of the Supporting Information, respectively.

H_2_-CO_2_ mixture densities (ρ) at 5, 25, and 50 MPa are plotted as a function of the mole fraction of H_2_ (*x*_H_2__) in Figure 1a,c,e. The computed densities are compared with predictions from equations of state found in REFPROP.^108^ Clearly, densities decrease monotonically and nonlinearly with the hydrogen content in the mixture. In Figure 1, densities computed from MD simulations closely match predictions from REFPROP,^108^ typically within 5% at all thermodynamic state points. The relative deviations between the computed and predicted densities are shown in Figures S5–S7 of the Supporting Information.

The ratio of the densities of pure CO_2_ and H_2_ can vary between 20 and 35, considering all pressures and temperatures. The large density contrast between the pure components also impacts the mixture densities, as seen by the positive curvature  in Figure 1a,c,e, suggesting a strong influence of CO_2_ content on the mixture densities. As expected, and as evident from Figure 1a,c,e, densities at a fixed pressure and mixture composition decreases with temperature. A ca. 31% increase in temperature (323.15 to 423.15 K), results in ca. 30% and ca. 40% reductions of densities of pure H_2_ and CO_2_, respectively, at 5 and 50 MPa. Variations in H_2_-CO_2_ mixture densities with temperature lie between the bounds set by the pure components. It can be concluded that density and temperature have a linear-like relationship. A closer look at the vertical axis in Figure 1a,c,,e, indicates that the density also increases with pressure. Barring the mixture at *x*_H_2__ = 0 and *T* = 323.15 K, ρ(*p*) can be fitted to a second-degree polynomial with an error of up to ca. 10%. At *x*_H_2__ = 0 and *T* = 323.15 K, densities sharply increase until *p* ≈ 10 MPa, followed by a more gradual rise. The proximity of this specific mixture to the critical point of CO_2_ (*T*_C_ = 304.15 K, *p*_C_ = 7.38 MPa)^125^ could explain the transition from gas-like behavior (*p* ≲ 10 MPa) to fluid-like behavior (*p* ≳ 10 MPa). The reader is referred to Figures S1–S4 of the Supporting Information for additional plots of densities as functions of mole fraction of H_2_ and pressure.

The compressibility factors (*Z*) of H_2_-CO_2_ mixtures are investigated to understand intermolecular interactions. Compressibilities vary nonlinearly with *x*_H_2__, showing values both above and below unity. Excellent agreement between simulations and REFPROP predictions is observed for various pressures and temperatures. In general, mixtures rich in CO_2_ exhibit attractive forces (*Z* < 1), while H_2_-enriched mixtures display repulsive forces (*Z* > 1). Temperature affects compressibilities differently at various pressures. Compressibilities tend to approach unity at low pressures and high temperatures. The behavior of compressibilities becomes more complex at higher pressures. Plots of compressibilities as a function of pressure and mole fraction of H_2_ are provided in Figures S8–S10 of the Supporting Information.

To summarize, CO_2_-H_2_ mixture densities are dominated by the CO_2_ content in the mixture. In mixtures with a high proportion of H_2_ (*x*_H_2__ ≳ 0.7), repulsive interactions dominate, leading to densities lower than those of an ideal gas. Conversely, CO_2_-rich mixtures have higher densities due to attractive forces compared to ideal gas densities. Gas- and liquid-like behavior of H_2_-CO_2_ mixtures is observed depending on the pressure, temperature, and mixture composition. The densities and compressibilities computed from MD simulations and REFPROP^108^ for all mixtures are tabulated in Table S2 of the Supporting Information.

### 3.2 Viscosities of CO_2_-H_2_ Mixtures

Viscosities (η) are usually independent of finite-size effects.^116,118^ The viscosities of various CO_2_-H_2_ mixtures as a function of *x*_H_2__ for 3 different pressures are shown in Figure 1b,d,f, while the remaining plots are displayed in Figures S12 and S13 of the Supporting Information. Similar to the mixture densities, the viscosities of H_2_-CO_2_ mixtures also show gas- and liquid-like behavior. Gas-like behavior is evident at 5 MPa, as depicted by viscosities within the range of a few μPas in Figure 1b.^100^ Regardless of temperature, viscosities decrease monotonically with *x*_H_2__. As the mixture becomes enriched with H_2_, the viscosities transition toward a more gas-like behavior, resulting in a gradual decrease of approximately 20% until *x*_H_2__ ≲ 0.8, followed by a steeper drop of about 40%. By increasing the pressure to 25 and 50 MPa, specifically below 373.15 K, the behavior of viscosities differs from that shown in Figure 1b, indicating a liquid-like behavior. For example, at 25 MPa and 323.15 K, the viscosity decreases sharply by ca. 67% from about 75 μPas for pure CO_2_ to around 25 μPas at *x*_H_2__ = 0.5. Subsequently, viscosities decrease nearly linearly with the mole fraction of H_2_. At 25 MPa and 423.15 K, η(*x*_H_2__) resembles gas-like behavior. Viscosities are significantly impacted by the H_2_ concentration in the H_2_-CO_2_ mixture, as indicated by the negative curvature of η(*x*_H_2__) in Figure 1d,f .

The variation of viscosities with temperature further exemplifies the gas- and liquid-like behavior of the H_2_-CO_2_ mixtures. At 5 MPa and any fixed *x*_H_2__, viscosities increase nearly linearly with temperature. An increase from 323.15 to 423.15 K results in a ca. 20% rise in the viscosities. The increase in viscosities with temperature reflects a gas-like behavior wherein the kinetic component of the viscosity exceeds the virial contribution due to molecular interactions, signifying the escalating influence of thermal motion on momentum transport in molecules. In contrast to gases, liquids experience a drop in viscosity with temperature.^13^ At 25 MPa in Figure 1d, for *x*_H_2__ ≲ 0.4, viscosities decrease with temperature. While the same behavior is observed at 50 MPa, the liquid-like behavior is prominent for *x*_H_2__ ≲ 0.55 in Figure 1f. Above a crossover concentration, *x*_H_2__ ≈ 0.4 at 25 MPa and *x*_H_2__ ≈ 0.55 at 50 MPa, viscosities increase with temperature due to the gas-like behavior of H_2_-enriched mixtures. The crossover concentration signals the transition from a liquid-like (or dense-gas like) behavior in CO_2_-enriched mixtures to a gas-like behavior in H_2_-enriched mixtures.

The viscosities can change significantly with pressure. Pure CO_2_ shows a 4 to 5 times increase in viscosity upon increasing the pressure between 5 and 50 MPa, typical of dense gas-like behavior.^11^ The addition of H_2_ to the mixture substantially reduces viscosity. Pure H_2_ maintains relatively low viscosities, with an insignificant dependence on pressure. The transition from gas-like to liquid-like behavior typically occurs within a pressure range of 30–40 MPa. These observations suggest that viscosity behavior is highly dependent on both pressure and composition, with clear distinctions between gas-like and liquid-like regimes. Plots for the viscosities as a function of pressure for various mole fractions of H_2_ are shown in Figures S14 and S15 of the Supporting Information.

Computed viscosities agree (on average) within 5% with REFPROP,^108^ barring a few systems at 5 and 10 MPa. Deviations up to 28% are observed, noticeable from the large uncertainties at 5 MPa in Figure 1b, for instance, the data point at 373.15 K. The sparse intermolecular interactions in the gas phase lead to large fluctuations in *p*(111) and require up to 40 ns to accurately compute viscosities. Another way to improve the accuracy of the computed viscosities is through more independent simulations. We refrained from conducting additional or longer simulations due to the satisfactory accuracy of the computed viscosities and the substantial computational costs associated with simulating the gas phase using MD. For instance, on a single core of an AMD7H12 2.6 GHz processor, simulating 1 ns of pure H_2_ at 5 MPa and 423.15 K requires ca. 15 CPU hours, while pure CO_2_ at 50 MPa and 323.15 K requires roughly 1 CPU hour. Computed and predicted values of viscosities for H_2_-CO_2_ mixtures are tabulated in Table S3 of the Supporting Information.

### 3.3 Self-Diffusion Coefficients of CO_2_-H_2_ Mixtures

Self-diffusivities computed from MD simulations might need to be corrected for finite-size effects,^116−119^ depending on the density of the system. Self-diffusivities are computed for systems containing 120, 250, 500, 1000, and 2000 molecules for 2 equimolar H_2_-CO_2_ mixtures: (1) 5 MPa, 423.15 K (33 kg/m^3^), and (2) 50 MPa, 323.15 K (389 kg/m^3^). For a system of 120 molecules, finite-size corrections for self-diffusivities in system (2) can reach up to 18% for CO_2_ and around 7% for H_2_, while in system (1), H_2_ and CO_2_ diffusivities remain unaffected by system size. The finite-size corrected self-diffusivity (*D*^self^) of pure CO_2_ at 2 MPa and 323.15 K (2.2 × 10^–8^ m^2^/s) agreed within a percent of the value reported by Moultos et al.^126^ Given the considerable variation in densities of H_2_-CO_2_ mixtures spanning nearly 2 orders of magnitude, finite-size effects are expected to affect the self-diffusivities of CO_2_ and H_2_ only at specific densities. Consequently, the Yeh–Hummer correction is applied when the correction term in eq 1 exceeds 1% of the uncorrected self-diffusivities. The reader is referred to Figures S17 and S18 for the plots of the finite-size effects.

**Figure 2:**
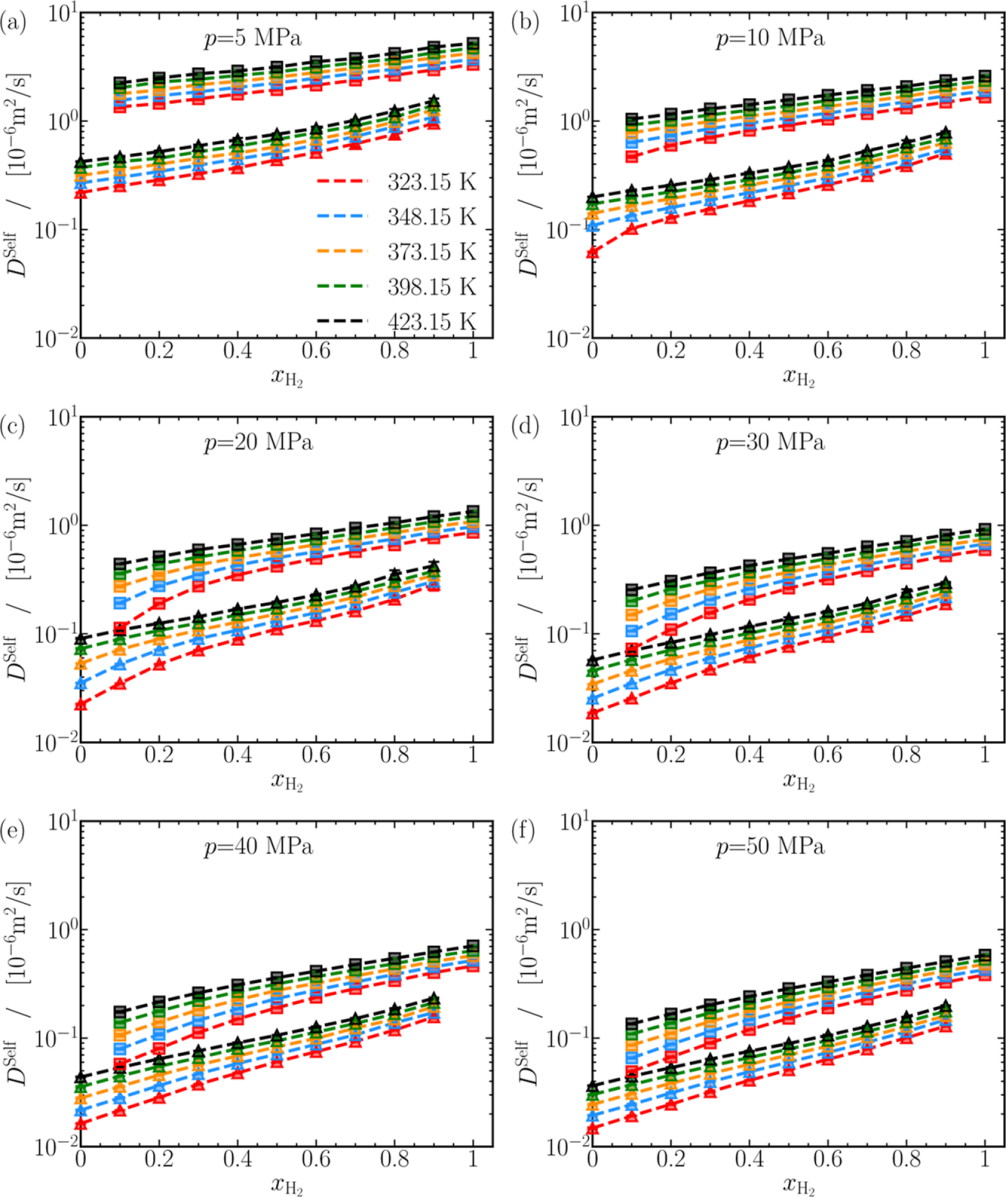
Self-diffusion coefficients (*D*^self^) of CO_2_ (triangles) and H_2_ (squares) in mixtures of CO_2_ and H_2_, computed from MD simulations. The computed self-diffusion coefficients are plotted as a function of the mole fraction of H_2_ in the mixture at various pressures. The uncertainties in the computed self-diffusion coefficients are smaller than the symbol sizes. The dashed lines act as guides to the eye. Lines are colored as per the legend in (a). Self-diffusivities of CO_2_ and H_2_ plotted on a linear scale are displayed in Figure S19a–f of the Supporting Information to aid comparison. The raw data of the self-diffusion coefficients of CO_2_ and H_2_ for all mixtures are provided in Table S3 of the Supporting Information.

**Figure 3:**
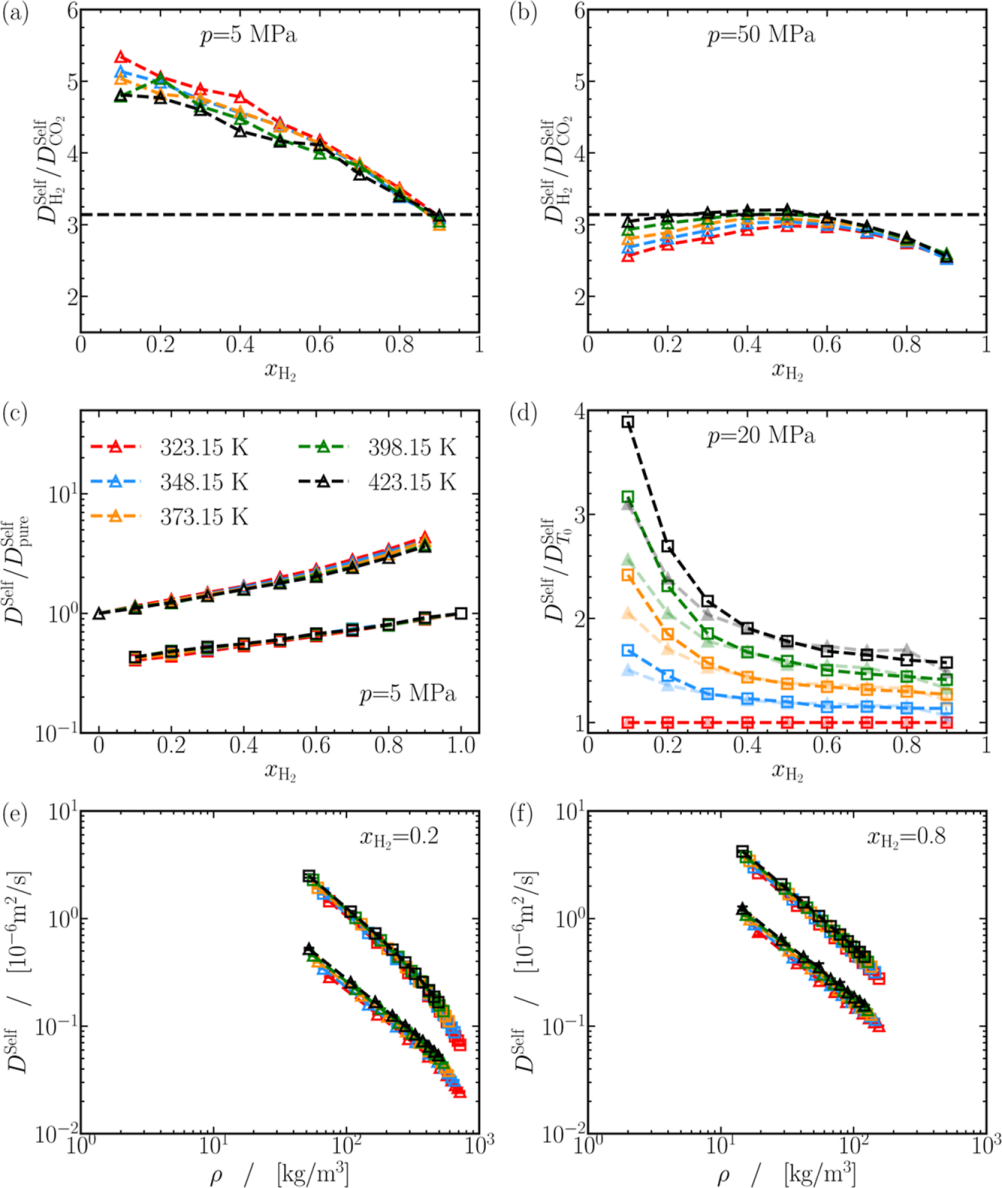
(a,b) Ratio of self-diffusivities of H_2_ and CO_2_ as a function of H_2_ mole fraction at 5 and 50 MPa, respectively. The dashed line represents the ratio predicted by the Stokes–Einstein relation. (c) Comparison of the self-diffusivities of CO_2_ (triangles) and H_2_ (squares) in CO_2_-H_2_ mixtures with their values in pure fluids at the same temperature at 5 MPa. (d) Comparison of the self-diffusivities of CO_2_ (triangles) and H_2_ (squares) in CO_2_-H_2_ mixtures with their values at *T*_0_ = 323.15 K at 20 MPa. (e,f) Self-diffusivities of CO_2_ (triangles) and H_2_ (squares) as a function of mixture densities at *x*_H_2__ = 0.2 and 0.8, respectively, with symbols colored according to the legend in (c). The ratios of self-diffusivities in (c) are displayed on a linearly scaled vertical axis in Figure S22 of the Supporting Information along with other pressures. Similarly, Figure S25 displays the densities and self-diffusivities in (e,f) plotted on linearly scaled horizontal and vertical axis. Figures S20–S25 of the Supporting Information contain additional plots for (a,b,d) at various pressures.

The range between 10^–8^ and 10^–6^ m^2^/s for self-diffusivities in Figure 2, implies dense-gas and gas-like behavior of H_2_-CO_2_ mixtures.^11^ As clearly shown in Figure 2, the self-diffusivities of H_2_ are always larger than those of CO_2_ at any pressure, temperature, or mixture composition by a factor between ca. 2.5 and 6. To explain why the self-diffusivities of H_2_ exceed those of CO_2_, the Stokes–Einstein (SE) relation^11,127^ is used. The SE equation links the self-diffusivity of a microscopic entity in liquid, viscosity, temperature, and effective radius (or a hydrodynamic radius, *R*^eff^) as^11,127^
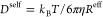
7Here, stick boundary conditions are assumed,^11,127^ resulting in a factor of 6π in the SE equation, in contrast to the 4π factor used with slip boundary conditions.^11,127^ It is also implicitly assumed that *R*^eff^ in eq 7 is unaffected by *T*.^11,45^ Treating CO_2_ and H_2_ as spherical entities with an *R*^eff^ equal to the bond length between carbon and oxygen (1.16 Å), and half the bond length between the two hydrogen atoms in H_2_ (0.37 Å), the ratio of their self-diffusivities as per eq 7 amounts to ca. 3.14, a value larger than unity. According to the SE relation, by the virtue of geometry, H_2_ diffuses faster than CO_2_ by roughly 3 times. In contrast to the expected ratio of ca. 3.14, according to the SE relation, the ratio of self-diffusivities for H_2_ and CO_2_ ranges from 2.5 to 6, indicating significant deviations. These deviations, as shown in Figure 3a, are particularly pronounced at 5 MPa, possibly due to the gas-like behavior of the mixtures, suggesting potential limitations in the applicability of the SE relation for gaseous systems. The agreement with the SE relation improves at larger pressures. For instance, Figure 3b indicates deviations less than 20% at 50 MPa, where the behavior of the mixtures resembles a liquid. The reader is referred to Figure S20a–d of the Supporting Information for similar plots between 10 and 40 MPa. While the SE equation provides a qualitative understanding of the self-diffusivities of CO_2_ and H_2_, quantitative predictions may deviate due to (1) the incorrect assumption that *R*^eff^ remains constant with temperature, as also noted by Saric and colleagues,^44^ and/or, (2) the oversimplified treatment of linear molecules like CO_2_ and H_2_ as spherical entities.

The impact of mole fraction of H_2_ and temperature on the self-diffusivities is now discussed. As anticipated and shown in Figure 2a–f, self-diffusivities of CO_2_ and H_2_ increase as the proportion of H_2_ increases in the mixture. This is because molecules can diffuse more in a gaseous medium than in liquid. To evaluate the effect of mole fraction of H_2_ on self-diffusivities, the values of *D*^self^ for CO_2_ and H_2_ in H_2_-CO_2_ mixtures are compared with corresponding values in pure fluids, as shown for the diffusivities at 5 MPa in Figure 3c. Adding H_2_ to the mixture can lead to a nearly exponential rise in the self-diffusivities of both CO_2_ and H_2_, with a maximum increase of approximately 2 to 4 times. A similar exponential increase in the self-diffusivities is observed also at other pressures, as shown in Figures S21 and S22 of the Supporting Information.

As anticipated, increasing temperature leads to larger self-diffusivities of H_2_ and CO_2_, as seen in Figure 2a–f due to the more gas-like nature of systems at larger *T*. The ratio of self-diffusivities of H_2_ and CO_2_ relative to their values at 323.15 K is plotted in Figure 3d at 20 MPa. The self-diffusivity of CO_2_ and H_2_ increases up to 300% at *x*_H_2__ = 0.1, while a modest increase up to 60% is observed for both species at *x*_H_2__ = 0.9. A similar trend is observed at all pressures, barring 5 MPa, where the self-diffusivity of both species increases by a maximum of ca. 100% at *x*_H_2__ = 0.1, but at *x*_H_2__ = 0.9, the increase is between 50 and 60%. At 5 MPa, however, self-diffusivities of H_2_ and CO_2_ increase by ca. 60% at all mixture compositions, exhibiting a linear-like response to temperature. It can be concluded that self-diffusivities of H_2_ and CO_2_ in mixtures rich in H_2_ respond similarly to temperature at all pressures. For mixtures rich in CO_2_ the response of self-diffusivities to temperature varies depending on the pressure, indicating a complex behavior. This complexity may be associated with the proximity of CO_2_ to the supercritical phase (*T*_C_ = 304.15 K, *p*_C_ = 7.38 MPa).^125^

The self-diffusivities of CO_2_ and H_2_ increase with the mole fraction of H_2_ and temperature but decrease with pressure. Conversely, mixture densities decrease with the mole fraction of H_2_ and temperature while increasing with pressure. The contrasting responses of self-diffusivities and densities imply a possible correlation between the two quantities. Figure 3e,f show the variation of self-diffusivities for CO_2_ and H_2_ with density at *x*_H_2__ = 0.2 and 0.8, respectively. A linear-like relationship, displayed on a log–log scale, between the self-diffusivities of both H_2_ and CO_2_ and density is apparent. A linear fit is calculated to best describe this relationship at all mixture compositions. The average value of the slopes of the linear fits for the case of CO_2_ is −1.00 ± 0.05 and −1.1 ± 0.1 for H_2_. Minor deviations from the calculated slope of around −1 are found for ρ exceeding ca. 400 kg/m^3^, in Figure 3e. Similar deviations in the slopes are also observed for pure CO_2_ for ρ ≳ 500 kg/m^3^. Barring these exceptions, it can be concluded that the self-diffusivities for CO_2_ and H_2_ are inversely proportional to the mixture density. When comparing Figure 3e,f, a significant vertical shift of the data points is observed, along with a minor horizontal shift. The shifts imply dependencies of self-diffusivities not only on the density but also on *x*_H_2__ and *T*. It appears improbable to establish a master equation linking self-diffusivities solely to densities. Studies by Van Loef^128^ and Harris and Trappeniers^129^ studying pure liquids, found that self-diffusivities are functions of density and temperature. In a binary mixture, an extra thermodynamic degree of freedom, the mole fraction of a component, must be considered alongside density and temperature to fully describe self-diffusivities. Establishing a function correlating self-diffusivities to density, temperature, and *x*_H_2__ will require additional efforts. Currently, no experimental data are available to compare the computed self-diffusivities in H_2_-CO_2_ mixtures. Additional plots of self-diffusivities as a function of ρ are shown in Figure S24a–d. The computed self-diffusivities in H_2_-CO_2_ mixtures are tabulated in Table S3 of the Supporting Information.

### 3.4 Thermodynamic Factors for Diffusion for CO_2_-H_2_ Mixtures

**Figure 4:**
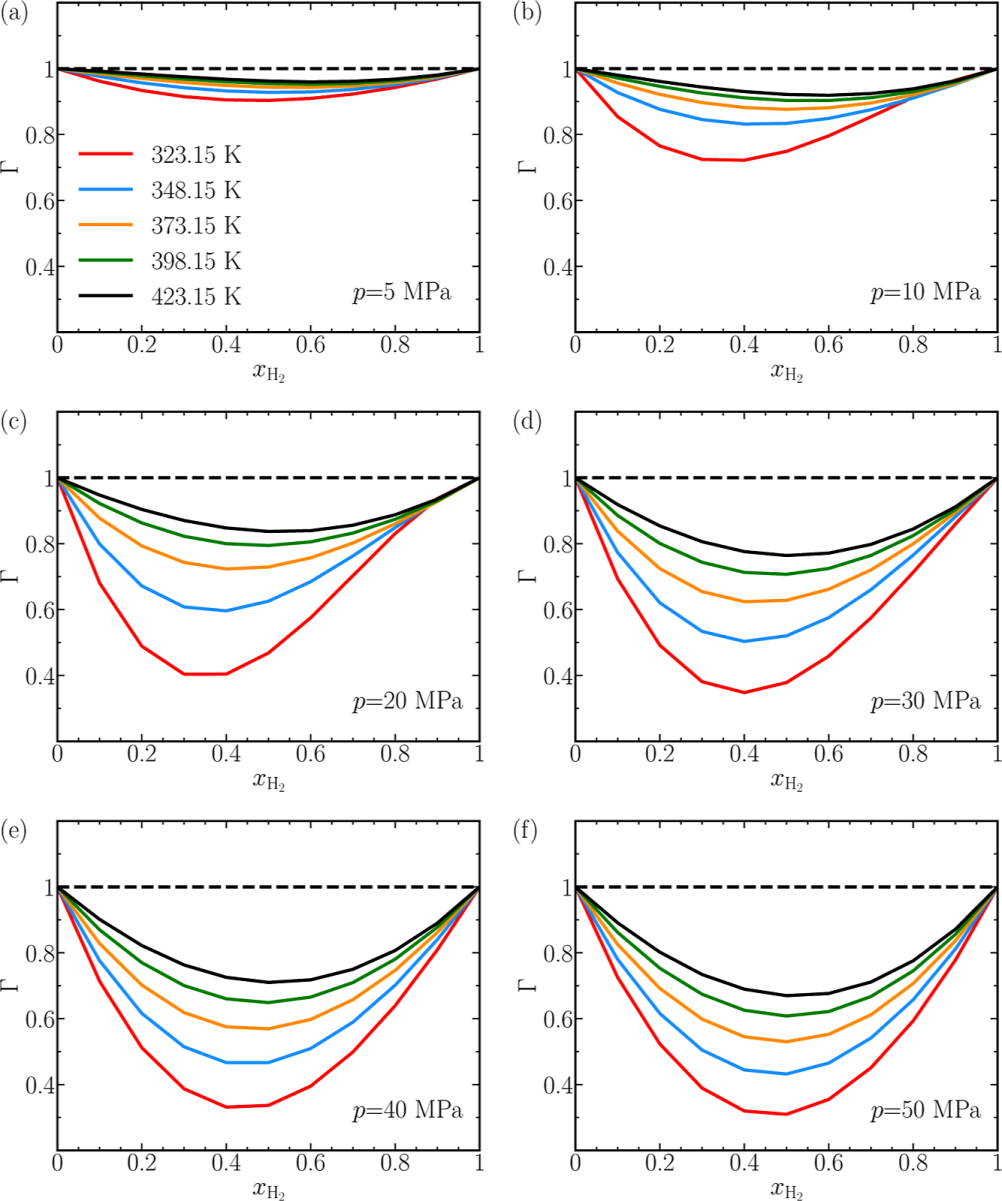
Thermodynamic factors (Γ) of CO_2_-H_2_ mixtures plotted as a function of the mole fraction of H_2_ (*x*_H_2__) for various pressures. Values of Γ are obtained from the Gibbs excess energies of H_2_-CO_2_ mixtures from REFPROP.^108^ The dashed line corresponds to the ideal-mixture limit, where Γ equals 1. Lines are colored as per the legend in (a). The raw data of the thermodynamic factors of CO_2_ and H_2_ for all mixtures are provided in Table S3 of the Supporting Information.

Figure 4 shows the thermodynamic factors calculated from the Gibbs excess energies obtained from REFPROP.^108^ The values of Γ for the thermodynamic states considered in this work are positive and vary between 0.25 and 1. Positive values of Γ signify that the mixtures are thermodynamically stable and do not undergo phase separation.^10^ A notable deviation from unity in the values of Γ for the CO_2_-H_2_ mixtures suggests highly nonideal behavior. Γ of a binary system must approach unity when approaching the pure-component limit.^10,12^

At a fixed mixture composition, the values of Γ tend to decrease with pressure. For instance, in Figure 4, for an equimolar mixture of CO_2_-H_2_ the value of Γ for the mixture drops from ca. 0.9 at *p* = 5 MPa (Figure 4a) to ca. 0.3 at *p* = 50 MPa (Figure 4f). This holds true for the values of Γ at other mixture compositions. For a fixed mixture composition, the value of Γ approaches 1 as the temperature rises from 323.15 to 423.15 K, regardless of the pressure. For instance, at *p* = 50 MPa (Figure 4f), the value of Γ increases from ca. 0.3 at *T* = 323.15 K to ca. 0.7. At a specific temperature and pressure, the values of Γ exhibit a slight asymmetry ca. *x*_H_2__ = 0.5, see Figure 4c (*p* = 20 MPa). The thermodynamic factors of all CO_2_ and H_2_ mixtures considered in our study are tabulated and displayed in Table S3 of the Supporting Information.

### 3.5 MS and Fick-Diffusion Coefficients for CO_2_-H_2_ Mixtures

MS diffusivities computed from MD simulations require finite-size corrections.^116−119^ To investigate finite-size effects in MS diffusivity calculations and to confirm the validity of eq 2 for our systems, equimolar H_2_-CO_2_ mixtures at 50 MPa and 323.15 K (389 kg/m^3^) consisting of 120, 250, 500, 1000, and 2000 molecules were chosen. Both methods, extrapolation of the line fitted to the MS diffusivities up to *N*_tot_ → ∞ and the prediction from eq 2, agreed within 3%. The finite-size correction to the MS diffusivity for the system of 120 molecules was ca. 20%, underscoring the significance of finite-size effects in the computation of MS diffusivities. Consequently, the correction suggested in eq 2 is applied if it exceeds 1% of the computed MS-diffusivities. The reader is referred to Figure S27 for the plots of the finite-size effects in MS diffusivities.

**Figure 5:**
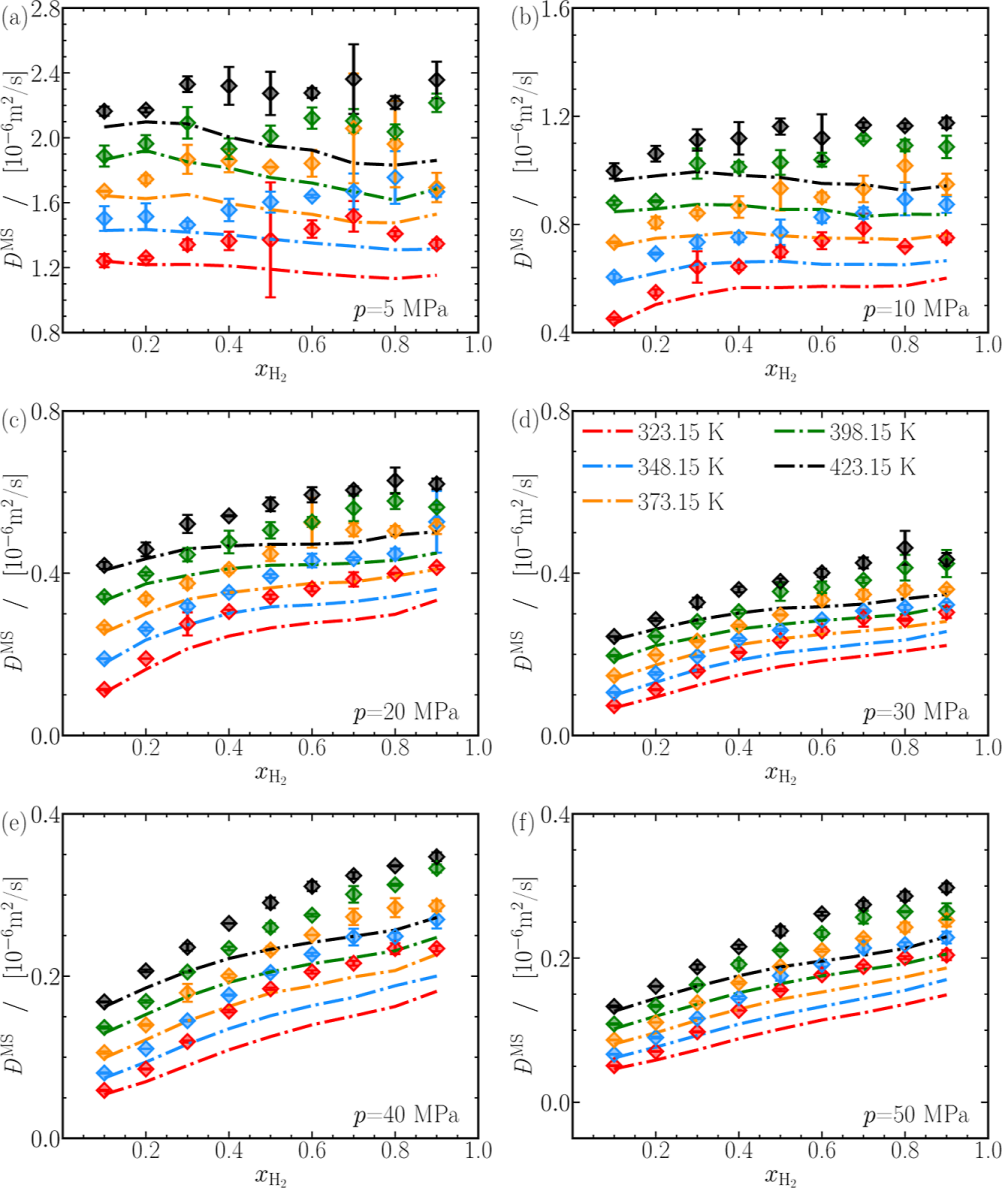
Finite-size-corrected Maxwell–Stefan diffusion coefficients as a function of the mole fraction of hydrogen (*x*_H_2__). The dashed-dotted lines are predictions from the Darken relation (eq 3, where the self-diffusion coefficients for CO_2_ and H_2_ are taken from Figure 2). Lines are colored as per the legend in (d). The raw data of the Maxwell–Stefan diffusion coefficients of CO_2_ and H_2_ mixtures are provided in Table S3 of the Supporting Information.

Figure 5a–f shows the finite-size corrected MS diffusivities (*D̵*^MS^) for various CO_2_-H_2_ mixtures, compared to the Darken prediction from eq 3. Finite-size corrected self-diffusivities of CO_2_ and H_2_ are used to compute the Darken predictions. The Darken prediction underestimates the computed MS diffusivities for the mixtures considered in our study. The maximum deviations between eq 3 and the computed MS diffusivities can reach ca. 30% around *x*_H_2__ ≈ 0.7, while a minimum deviation of about 5% occurs at *x*_H_2__ ≈ 0.1.

**Figure 6:**
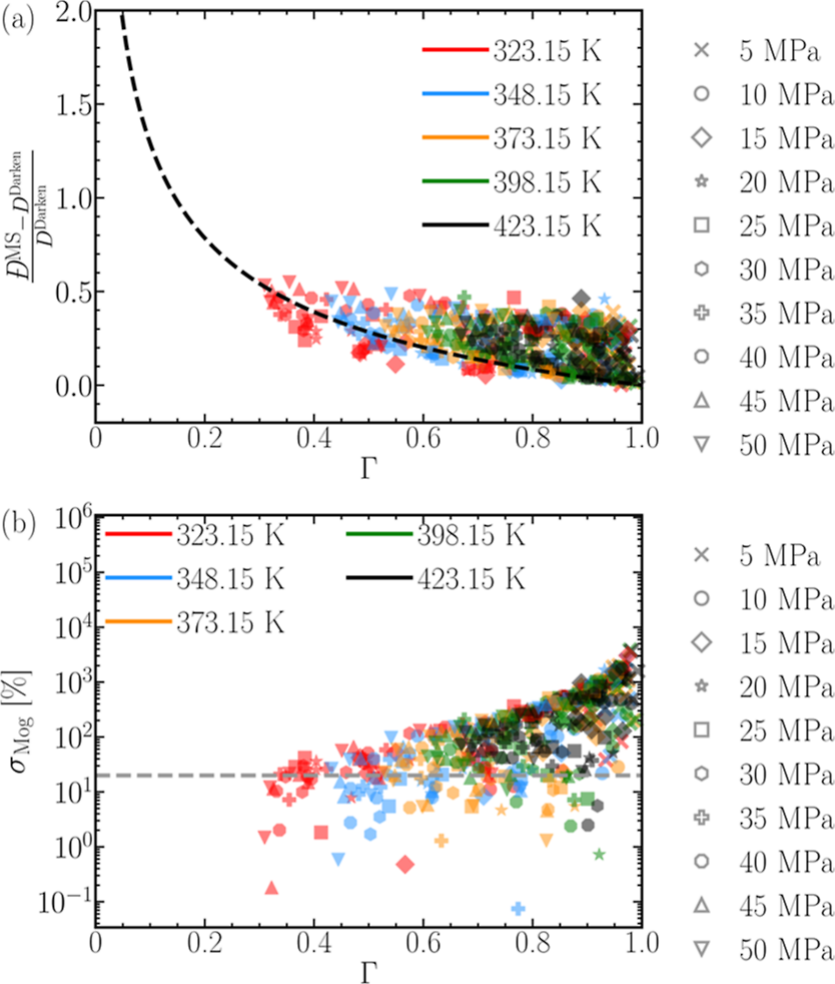
(a) Relative differences between the Darken relation and the computed MS diffusivities plotted as a function of the thermodynamic factor Γ for all pressure, temperature, and mixture compositions. The dashed line is the Moggridge correlation,^122,130−134^ see eq 8. The symbols code for the pressure and the colors code for the temperature. For example, a blue star would correspond to 20 MPa and 348.15 K. (b) Relative deviations, in percentage, of the symbols in (a) from the Moggridge correlation (σ_Mog_). The dashed line corresponds to a deviation of 20% to aid visual comparison. The percentage deviation extends across 5 orders of magnitude.

The significant deviations between the computed MS diffusivities and Darken predictions imply a significant nonideality of the H_2_-CO_2_ mixtures. Since the thermodynamic factor (Γ) reflects the nonideality of the mixture, it is valuable to explore the potential correlation between *D̵*^MS^ – *D*^Darken^ and Γ, as suggested by Wolff et al.^122^Figure 6a shows the relative deviation of the MS diffusion coefficient from the Darken relation as a function of the thermodynamic factor, for all mixtures considered in our study. The Moggridge correlation^122,130−134^ when recast into a suitable form
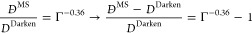
8reasonably describes the relative deviation of *D̵*^MS^ from *D*^Darken^ and the thermodynamic factor, with the best agreement achieved at low temperatures. Figure 6b helps carefully assess the performance of the Moggridge correlation^122,130−134^ by showing the percentage deviations between the symbols and the dashed line in Figure 6a. The deviations in Figure 6b are within 20% when describing mixtures below 348.15 K and above 40 MPa. In other words, the deviation between the computed MS diffusivities and the Darken relation is accurately captured in the liquid-like phase. The Moggridge correlation becomes less accurate when considering H_2_-CO_2_ mixtures at high temperatures and low pressures. For instance, the black cross representing 5 MPa and 423.15 K exhibits the maximum deviation. To conclude, Moggridge correlation can be advantageous in predicting the deviation between the computed MS diffusivities and the Darken relation for mixtures behaving as liquids. In summary, knowing the self-diffusivities of H_2_ and CO_2_ and the thermodynamic factor can facilitate the prediction of *D̵*^MS^ of the mixture with an accuracy of approximately 20% for mixtures between 40 and 348.15 MPa.

**Figure 7:**
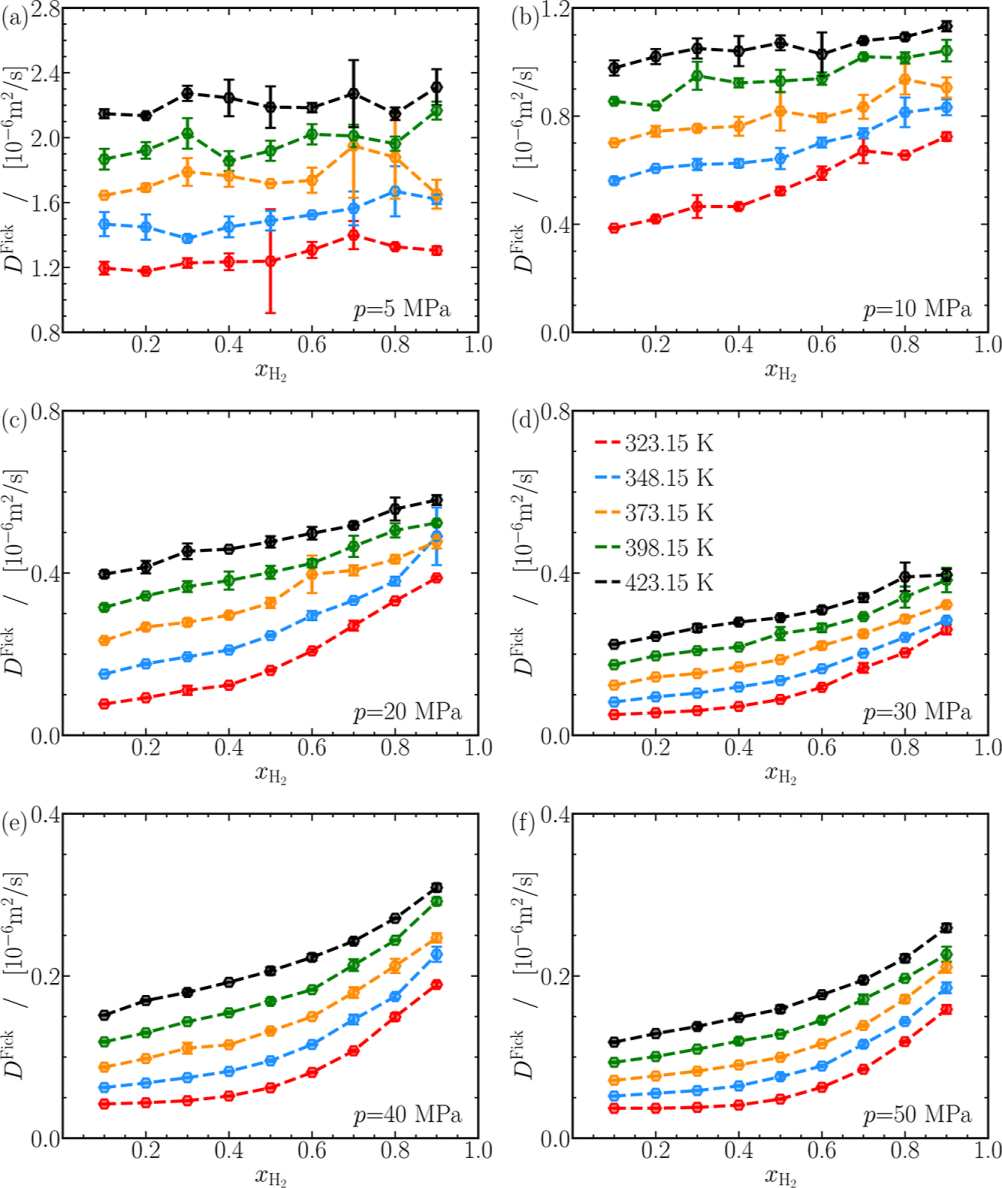
Finite-size-corrected Fick diffusion coefficients as a function of the mole fraction of hydrogen (*x*_H_2__). Lines are colored as per the legend in (d). The dashed lines act as guides to the eye. The raw data of the Fick diffusion coefficients of CO_2_ and H_2_ for all mixtures are provided in Table S3 of the Supporting Information.

Fick diffusivities for CO_2_-H_2_ mixtures are the products of the MS diffusivities and the thermodynamic factors, as shown in eq 4. Similar to the self- and MS diffusivities, finite-size corrections are disregarded if the corrections are less than 1% of the computed values, as shown in eq 4. Figure 7a–f shows the Fick diffusivities as a function of the mole fraction of hydrogen. The statistical uncertainties in the computed Fick diffusivities are noticeable, particularly below ca. 10 MPa, as shown in Figure 7a–c. This increased uncertainty, such as at 5 MPa, 323.15 K, and *x*_H_2__ = 0.5, is attributed to sparse intermolecular collisions in the gas phase, leading to slow convergence toward diffusive behavior of the cross-correlation of mean-squared displacements of H_2_ and CO_2_. To enhance accuracy, more independent simulations could be performed, but we refrained from doing so due to the significant computational costs associated with gas-phase MD simulations. The Fick diffusivities computed for all pressures, temperatures, and mixture compositions vary between 10^–8^ m^2^/s at 50 MPa and 10^–6^ m^2^/s at 5 MPa, indicating dense-gas/liquid-like behavior at high pressures and gas-like behavior at low pressures. In contrast to the smooth increase observed for self-diffusivities of H_2_ and CO_2_ in Figure 2a–f, the behavior of Fick diffusivities varies as the content of H_2_ increases in the mixture depending on the pressure. At 5 MPa in Figure 7a, Fick diffusivities exhibit minimal dependence on mixture composition, whereas at 50 MPa in Figure 7f, they can increase by 3 to 4 times with higher H_2_ enrichment in the mixture. The variation in Fick diffusivities contrasts with the consistent increase observed in the self-diffusivities of H_2_ and CO_2_ with the content of H_2_ in the mixture.

In general, temperature elevation increases Fick diffusivities regardless of mixture composition or pressure. The extent of this increase, however, varies for different mixtures. Notably, for mixtures abundant in H_2_ (*x*_H_2__ = 0.9), Fick diffusivities consistently double when the temperature rises from 323.15 to 423.15 K across all pressures. Conversely, for mixtures rich in CO_2_ (*x*_H_2__ ≲ 0.2), the increase ranges between ca. 100% (at 5 MPa) and ca. 300% (at 20 MPa) depending on pressure, emphasizing the significant impact of temperature on Fick diffusivities, particularly for CO_2_-rich mixtures. The effect of temperature on Fick diffusivities aligns with trends seen in the self-diffusivities of both CO_2_ and H_2_.

#### 3.5.1 Prediction of Fick Diffusion Coefficients Using Kinetic Theory, Fuller Correlation, the Corresponding States Principle, and Moggridge’s Correlation

Due to the lack of experimental data or a specific theory to predict Fick diffusivities in CO_2_-H_2_ mixtures at pressures larger than atmospheric pressure, the computed diffusivities are compared to various models: (1) Chapman Enskog expression from kinetic theory,^11^ (2) the correlation established by Fuller, Schettler, and Giddings (FSG),^30−32^ (3) the corresponding states principle,^11^ and (4) predictions from MS diffusivities using Moggridge’s correlation.^122,130−134^

**Figure 8:**
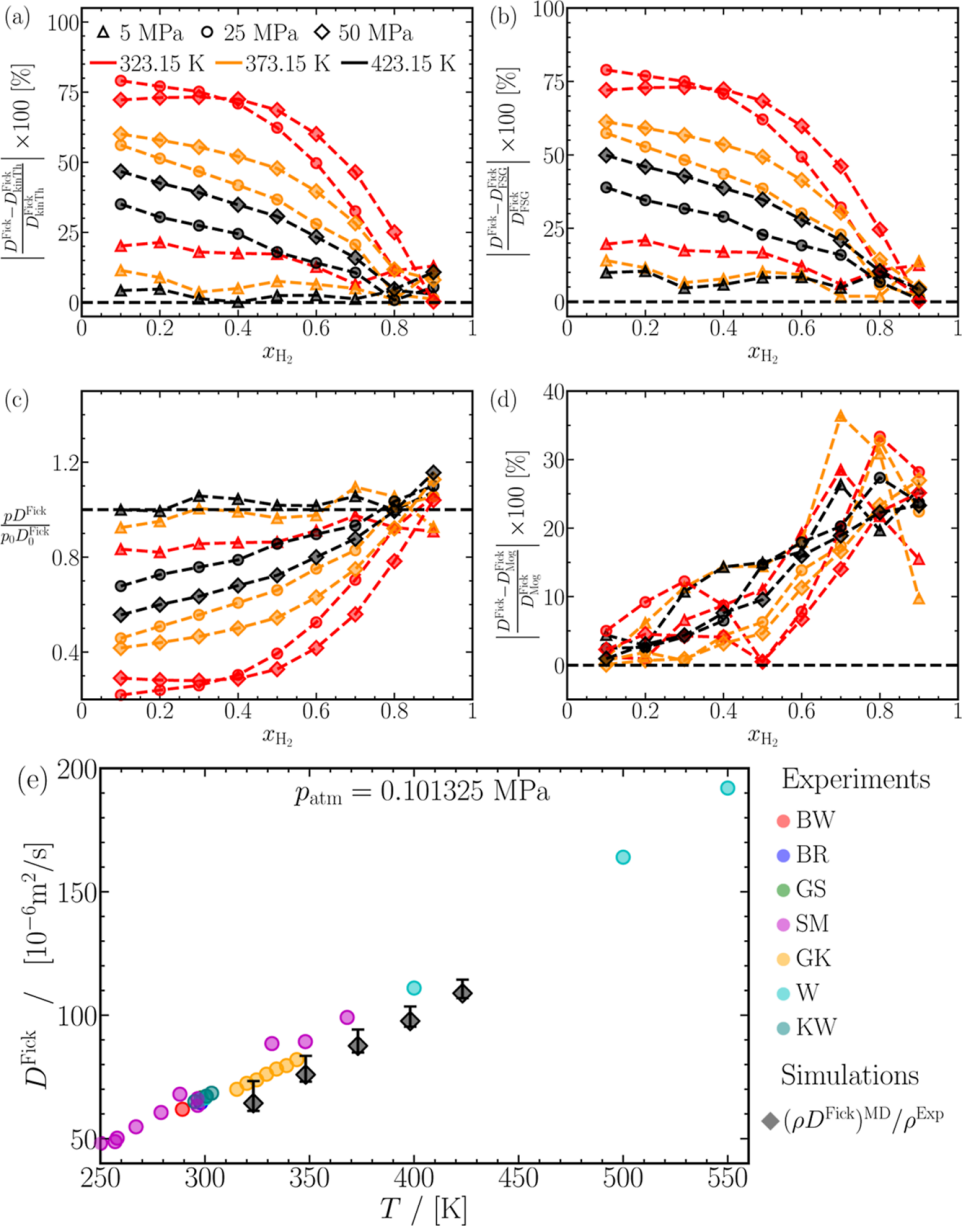
Absolute relative deviations between Fick diffusivities computed from MD simulations and predictions using (a) kinetic theory,^16−18^ (b) FSG correlation,^11,30−32^ (c) corresponding states principle^11^ for *pD*^Fick^, where *D*_0_^Fick^ = *D*^Fick^(*p*_0_ = 0.1 MPa) is evaluated using kinetic theory,^16−18^ and (d) Moggridge correlation^122,130−134^ for *DH*^MS^. (e) Experimentally measured Fick diffusivities of CO_2_-H_2_ mixtures at atmospheric pressure (*p*_atm_) compared to Fick diffusivities extrapolated from MD simulations of equimolar CO_2_-H_2_ mixtures at 5 MPa and temperature *T* using the principle of corresponding-states^11^ (also see eq 11). The asymmetric error bars indicate Fick diffusivities calculated using *x*_H_2__ = 0.1 and 0.9. Experiments by Boardman and Wild^145^ (BW), Boyd et al.^168^ (BR), Giddings and Seager^165^ (GS), Saxena and Mason^136^ (SM), Gavril et al.^135^ (GK), Weissman^137^ (W), and Kestin et al.^161^ (KW) are chosen for comparison.

Figure 8a shows the relative deviations of the computed Fick diffusivities from the kinetic theory expression of Chapman and Enskog. It can be expected that kinetic theory effectively predicts Fick diffusivities when mixtures behave ideally, typically observed at low pressures, high temperatures, and in mixtures rich in H_2_. In line with this expectation, a close agreement within 5% is evident at 423.15 K and 5 MPa for all mixtures. Below 423.15 K, predictions of kinetic theory are accurate within ca. 10% at 373.15 K and ca. 20% at 323.15 K. The computed Fick diffusivities of mixtures rich in H_2_ (*x*_H_2__ ≳ 0.8) can be predicted within 15% regardless of the pressure. As anticipated, a severe loss of accuracy in kinetic theory (up to 75%) is observed for CO_2_-rich mixtures (*x*_H_2__ ≳ 0.5) at 25 and 50 MPa, regardless of the temperature. In conclusion, predictions of *D*^Fick^ from kinetic theory are reliable up to ca. 20% at pressures of 5 MPa for CO_2_-H_2_ mixtures.

The FSG correlation^30−32^ is a widely used method for estimating Fick diffusivities.^30−32^ The relative deviations between the computed and predicted diffusivities of CO_2_-H_2_ mixtures are shown in Figure 8b. Figure 8a,b exhibits striking similarity, indicating predictive capabilities of the FSG correlation comparable to the kinetic theory. This is not surprising, provided that Fuller’s empirical relation is based on the Chapman and Enskog expressions from kinetic theory. It can be concluded that the kinetic theory and Fuller correlation can reliably estimate the Fick diffusivities within 25% in H_2_-CO_2_ mixtures up to 5 MPa and temperatures between 323.15 and 423.15 K. For pressures below ca. 5 MPa and temperatures above ca. 423.15 K, the accuracy of predicted Fick diffusivities from kinetic theory, and the Fuller correlation are expected to increase, consistent with findings in the literature.^11,13^

To test if accurate predictions of Fick diffusivities can be made at larger pressures, the performance of the corresponding state principle is examined. The ratio *pD*^Fick^/*p*_0_*D*_0_^Fick^ is evaluated at all thermodynamic state points, where *D*_0_^Fick^ is the Fick diffusivity evaluated at a reference pressure *p*_0_ = 0.1 MPa and the corresponding temperature *T*. When the ratio equals unity, *D*^Fick^ at any pressure and temperature can be predicted since the normalization factor is readily accessible. Figure 8c shows that the ratio is within 3% of unity at 5 MPa and 423.15 K. Like kinetic theory and FSG correlation, predictions are less accurate (deviations up to ca. 40%) at high pressures for mixtures rich in CO_2_. Conversely, mixtures rich in H_2_ (*x*_H_2__ ≳ 0.8) agree within 15–20% with kinetic theory and Fuller correlation. Rather than using pressure *p* to assess the ratio *pD*^Fick^/*p*_0_*D*_0_^Fick^, an alternative approach is to use density ρ, as illustrated in Figure S31 of the Supporting Information. While the performance of the corresponding states principle remains qualitatively similar, there is a marginal improvement of ca. 10% in the prediction of Fick diffusivities at 5 MPa, as shown in Figure S31 of the Supporting Information. In a nutshell, predicting Fick diffusivities is feasible through the kinetic theory, FSG correlation, or the corresponding states principle, specifically for mixtures at low pressures and high temperatures or enriched with hydrogen.

Due to the superior performance of Moggridge’s correlation, specifically at high pressures and low temperatures, this empirical method could be advantageous for predicting Fick diffusivities at these conditions (see Figure 6a,b). By combining eqs 4 and 8, Moggridge’s correlation can be extended to describe Fick diffusivities as
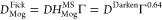
9

As anticipated, in Figure 8d, the Fick diffusivities predicted using eq 9 closely match the computed values, staying within 5% at 25 and 50 MPa for temperatures of 323.15 and 348.15 K. For CO_2_-rich mixtures (*x*_H_2__ ≲ 0.2), the computed and predicted Fick diffusivities deviate by less than ca. 5%, irrespective of pressure and temperature. In sharp contrast, for H_2_-rich mixtures (*x*_H_2__ ≳ 0.7) and mixtures at low pressures, deviations from the computed Fick diffusivities can reach approximately 35%.

In summary, Chapman and Enskog’s kinetic theory, FSG correlation, and the corresponding states principle perform well for ideal-gas-like mixtures. Fick diffusivities for H_2_-CO_2_ mixtures below 5 MPa can be accurately predicted within 5–10%. These kinetic theory-based approaches also demonstrate good performance for mixtures rich in H_2_ (*x*_H_2__ ≳ 0.8), with an accuracy of approximately 15–20%. Deviations can be as high as 75% when predicting Fick diffusivities of nonideal mixtures. For higher pressures and CO_2_-rich mixtures (*x*_H_2__ < 0.5), using Moggridge’s correlation for MS diffusivities yields Fick diffusivities accurate to within 5–10%. Values of MS and Fick diffusivities for all CO_2_ and H_2_ mixtures considered in our study are provided in Table S3 of the Supporting Information.

#### 3.5.2 Comparison of MD-Computed Fick Diffusivities with Experiments

We aim to (indirectly) compare the Fick diffusivities of CO_2_-H_2_ mixtures from MD simulations to existing experiments at *p*_atm_ = 1 atm (see Table 1). Computing Fick diffusivities at atmospheric pressure using MD simulations is possible but impractical due to the severe computational demands accompanying gas phase simulations.

The principle of corresponding states can indirectly compute Fick diffusivity at an unknown pressure, in our case, the atmospheric pressure, by extrapolating known data from a reference pressure.^11,13^ For comparison with experiments, Fick diffusivities computed from MD simulations at a reference pressure of 5 MPa are used to estimate mutual diffusivities at atmospheric pressure. Comparison of the computed Fick diffusivities at 5 MPa with those estimated using kinetic theory at 0.1 MPa reveals that the product of pressure (or density) and mutual diffusivities remains nearly constant (see Figure 8e), with deviations of approximately 20%. This trend is consistent when analyzing densities instead of pressures, showing agreement within 10%, as shown in Figure S31 of the Supporting Information. The principle of corresponding states for the product of density and Fick diffusivity offers a practical approach to extrapolating computed Fick diffusivities to conditions of experimental relevance. The Chapman-Enskog expression for mutual diffusivity, denoted as *D*_AB_, of rigid spheres with unequal masses is given by^11,17,18^
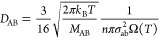
10where *D*_AB_ [m^2^ s^–1^] is the mutual diffusivity of the A-B mixture, *M*_AB_ is the reduced mass of A and B, *k*_B_ is the Boltzmann constant, *n* [m^–3^] is the number density, σ_AB_ [m] is the characteristic length of the intermolecular force law, and Ω(*T*) is the temperature-dependent collision integral for diffusion. Multiplying both sides of eq 10 by *nM*_mix_/*N*_Av_, where *M*_mix_ = *M*_A_*x*_A_ + *m*_B_*x*_B_ is the molecular mass of the mixture in kg·mol^–1^, and *N*_Av_ [mol^–1^] is the Avogadro number, which allows us to express it as
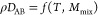
11

The functional form of eq 11 suggests that the product of density and Fick diffusivity can be equated between two thermodynamic states under the same temperature and mixture composition, as *M*_mix_ depends on the mole fraction of species A. Although eq 10 assumes spherical molecules, the equation has been shown to be effective at low pressures, irrespective of the shape of the molecules.^11,13,17^

From Figure 8e, we observe good agreement (within ca. 15%) between the Fick diffusivities predicted from the principle of corresponding states using eq 11 (filled diamonds) for an equimolar mixture composition, and experimental data by Gavril et al.,^135^ Saxena and Mason,^136^ and Weissman.^137^ The asymmetric error bars associated with each symbol in Figure 8e, representing the values of *D*^Fick^ calculated using eq 11 with mixture compositions of *x*_H_2__ = 0.1 and 0.9, respectively, imply a weak dependence of Fick diffusivities on mixture concentration. Accounting for this variability in the Fick diffusivities by choosing different mixture compositions enhances agreement with the experiments (between ca. 1 and 5%). Leveraging the close agreement between MD computed densities and REFPROP^108^ predictions (see Figures 1 and S5–S7 of the Supporting Information), Fick diffusivities can be evaluated at experimentally relevant conditions for mixture compositions of 0.001 and 0.999, yielding consistent results. Experimental measurements used in Figure 8e are not corrected for composition effects, known to affect diffusivities by ca. 5%,^19^ potentially influencing agreement between MD simulations and experimental measurements. Using the product of pressure and Fick diffusivity in eq 11 yields similar conclusions. In summary, MD simulations provide reliable estimates of Fick diffusivities at conditions relevant to atmospheric pressure and temperatures between 323.15 and 423.15 K.

### 3.6 Phase Equilibria of CO_2_-NaCl Brine Mixtures

**Figure 9:**
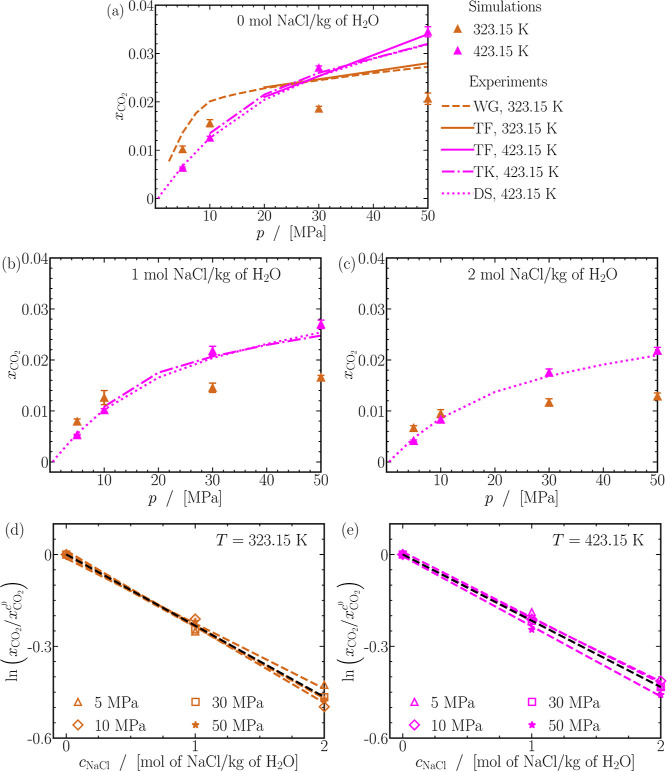
(a–c) CO_2_ solubilities in NaCl brine computed from MC simulations (symbols) compared to experiments (lines) for varying brine concentrations. Experimental studies of Weibe and Gaddy (WG),^169^ Tödheide and Frank (TF),^138^ Takenouchi and Kennedy (TK),^170,171^ and Duan and Sun (DS)^56,88^ are considered for the comparison. (d,e) Reduction in the solubility of CO_2_ as a function of NaCl concentration in brine. The logarithm of the ratio of CO_2_ solubility in brine to its solubility in pure water is plotted as a function of NaCl concentration. Linear fits to the data are shown as dashed lines. The raw data of the solubilities are provided in Table S4 of the Supporting Information.

The solubilities of CO_2_ in pure water and NaCl brine at 323.15 and 423.15 K between 5 and 50 MPa and for salt concentrations of 1 and 2 mol of NaCl/kg of H_2_O. At 323.15 K in Figure 9a–c, CO_2_ undergoes a significant linear-like increase in solubility within the liquid-rich phase up to *p* ≲ 10 MPa. Beyond this pressure, the solubility continues to increase linearly, albeit more gradually. The distinct slopes in these two regions reflect a transition from a gas-like to a liquid-like behavior of CO_2_. At 423.15 K, *x*_CO_2__ varies almost quadratically with pressure. As *p* → 0, extrapolation of *x*_CO_2__ shows that the solubility of CO_2_ also approaches 0, in agreement with the definition of the Henry regime. In general, insensitive to the concentration of NaCl in H_2_O, increasing pressure from 5 to 50 MPa results in a 2-fold increase in *x*_CO_2__ at 323.15 K and a 5-fold increase in *x*_CO_2__ at 423.15 K. The effect of temperature on the solubility of CO_2_ is nontrivial. Beyond a certain threshold pressure *p*_Th_, the solubility of CO_2_ in the NaCl brine increases with temperature. Below *p*_Th_, temperature has the opposite effect, leading to a decrease in CO_2_ solubility. This threshold pressure appears to depend on the NaCl concentration in H_2_O.

Solubilities of CO_2_ in NaCl brine computed from simulations are compared to experimental data obtained from Weibe and Gaddy (WG),^137^ Takenouchi and Kennedy (TK),^33^ Duan and Sun (DS),^56^ and Tödheide and Frank (TF).^138^ In Figure 9a–c the computed solubilities at 323.15 K deviate from experimental measurements by between 20% at *p* ≲ 10 MPa and 30% at *p* ≳ 30 MPa. In contrast, an agreement between 5 and 10% is observed between simulations and most experiments at 423.15 K and for all salt concentrations. Previous simulation studies by Liu et al.^60,61^ and Orozco et al.^62^ report similar disagreements between experiments and MC simulations at low temperatures. The culprit behind the discrepancies between simulations and experiments could either be the incorrect mixing rules describing the molecular interactions between CO_2_ and H_2_O, or the inaccuracies in the force fields developed for H_2_O and CO_2_ with respect to the solubility calculations.

The salting-out effect occurs in solutions when certain salts (ionic compounds) are added, leading to a decrease in the solubility of nonionic solutes in the solvent, typically organic compounds. This is due to the presence of ions in the solution altering the interactions between solvent and solute molecules, resulting in reduced solubility.^139^ The salting-out effect has been well studied in the context of CO_2_ solubility in aqueous solutions of many ionic salts, including NaCl.^55,56,88^ The influence of salt concentration on the solubility of CO_2_ becomes evident when plotting the ratio of *x*_CO_2__ relative to its solubility in pure water under the same thermodynamic conditions, as a function of the salt concentration. This is the well-known Setschenow equation^140^
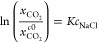
12where  is the reference solubility of CO_2_ in H_2_O at the same pressure at a chosen temperature. In Figure 9d,e, the solubility ratios are plotted on the vertical axis as a function of the salt concentration, in a format consistent with the Setschenow equation (eq 12). A linear relationship is observed between the solubility ratios and salt concentration, consistent with eq 12, exhibiting a slope of ca. −0.21 regardless of temperature and pressure. The negative slope implies a reduced solubility of CO_2_ in brine upon increasing the salt concentration. Specifically, the solubility of CO_2_ reduces, at any chosen pressure and temperature, by ca. 20% when a mole of NaCl is added to pure water results. A subsequent rise in salt concentration to 2 mol NaCl/kg H_2_O results in a further reduction of CO_2_ by another ca. 20%.

As an additional check of our simulations, the fugacity coefficients of CO_2_ in the gas phase computed along the VLE of CO_2_-NaCl brine at a given composition (*y*_CO_2__, *y*_H_2_O_), pressure, and temperature are compared to the data from REFPROP.^108^ Fugacity coefficients are a direct reflection of the excess chemical and mixture density, as indicated by eq 6. In general, the values of φ_CO_2__ and  were below unity, signifying the nonideality of CO_2_-H_2_O mixtures in the gas phase. The fugacity coefficients were found to decrease with pressure and increase with temperature. For all thermodynamic conditions investigated in our study, the values of φ_CO_2__ and  agreed with REFPROP^108^ within 2–3%.

The water content in the gas phase  at 323.15 and 423.15 K is plotted in Figure 12e,f. Clearly, from Figure 12e,f, a substantial quantity of water is present in the gas phase at 423.15 K in comparison to 323.15 K. At 323.15 K, the water content in the gas phase is unaffected by pressure, while the water content decreases by ca. 3 times between 5 and 50 MPa at 423.15 K. The quantity of water in the gas phase is insensitive to the NaCl concentration in brine.

Additional plots pertaining to the effect of temperature, pressure, and salt concentration on the VLE CO_2_-NaCl brine systems is shown in Figures S35–S37 of the Supporting Information.

### 3.7 Phase Equilibria of H_2_-NaCl Brine Mixtures

**Figure 10:**
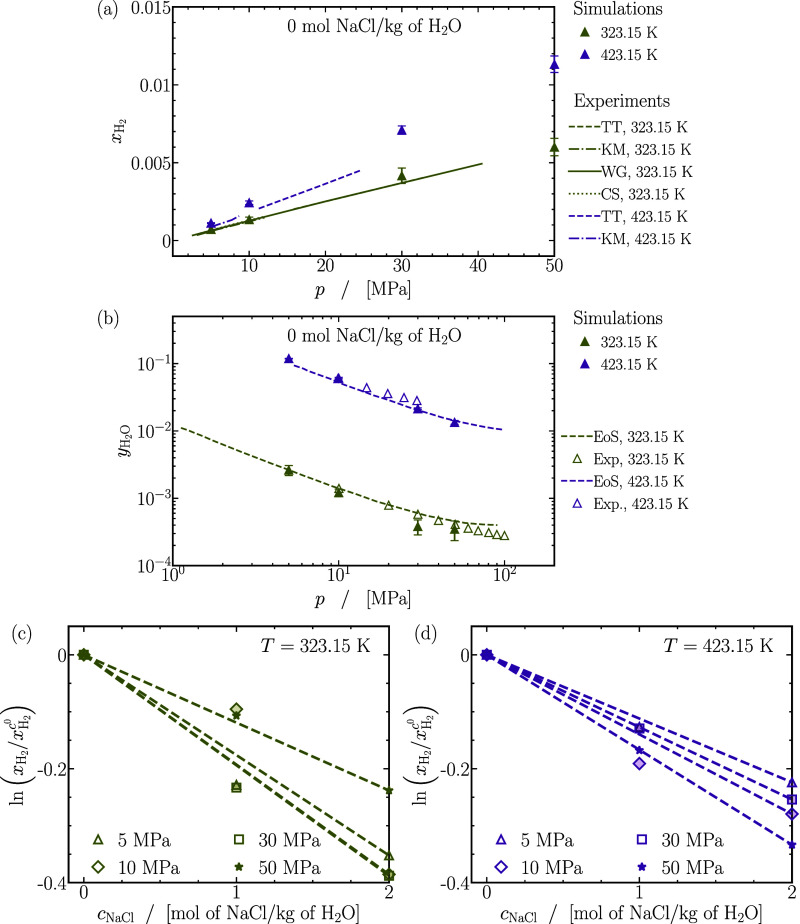
(a) Comparison of simulations (symbols) with experimental (lines) predictions for H_2_ solubilities in pure H_2_O. Experimental studies of Torín Ollarves and Trusler (TT),^79^ Kling and Maurer (KM),^172^ Wiebe and Gaddy (WG),^173^ and Chabab et al. (SC)^57^ are considered for the comparison. (b) Comparison of the water content in the gas phase between our simulations and the experiments from Torín Ollarves and Trusler (TT). Predictions from the equation of state from Torín Ollarves and Trusler (TT) are also plotted alongside. (c,d) Reduction in the solubility of H_2_ as a function of NaCl concentration in brine. The logarithm of the ratio of H_2_ solubility in brine to its solubility in pure water is plotted against NaCl concentration. Linear fits to the data are shown as dashed lines. The raw data of the solubilities are provided in Table S5 of the Supporting Information.

Figure 10a shows the solubilities of H_2_ in pure water at 323.15 and 423.15 K for pressures between 5 and 50 MPa. In Figure 10a, the H_2_ solubility in pure H_2_O shows a linear increase with pressure, indicating adherence to the Henry regime across the pressure range, with solubilities approaching 0 in the limit of *p* → 0. Increasing the pressure from 5 to 50 MPa leads to a near-10-fold increase in the solubility of H_2_. Interestingly, the impact of pressure on H_2_ solubility remains consistent regardless of salt concentration or temperature. In contrast to CO_2_, increasing temperature consistently enhances the dissolution of H_2_ in both H_2_O and NaCl brine. The impact of temperature however is less pronounced in NaCl brine, where a smaller increase in solubility is observed compared to pure H_2_O. For instance, an increase in temperature from 323.15 to 423.15 K results in a ca. 80% increase in solubility in pure H_2_O, whereas the increase is around 40% in brine containing 1 mol NaCl/kg of H_2_O, and nearly 30% in brine containing 2 mol NaCl/kg of H_2_O.

The solubilities of H_2_ in NaCl brine computed from MC simulations are compared to the available literature data in Figure 10a. Computed solubilities agree within ca. 5–10%, of the experimental data from Torín-Ollarves and Trusler^79^ for the entire range of pressures at 323.15 K. Disagreements between simulations and experiments arise at 423.15 K, with the solubilities being overestimated by simulations by a maximum of ca. 27% at *p* = 30 MPa. In Figure 10a, the disagreement between simulations and experiments at 423.15 K increases with pressure. The water content in the gas phase () shown in Figure 10b at 323.15 and 423.15 K aligns well with the experimental data from Torín-Ollarves and Trusler,^79^ with deviations between 5 and 10% for various pressures.

The salting-out effect in H_2_ solubility on NaCl brine has been explored in experiments by Chabab et al.^57,59^ Plotting the solubility ratios as a function of the salt concentration according to the Setschenow equation (eq 12) yields a linear relation, as shown in Figure 10c,d. Unlike CO_2_ in Figure 9d,e, the fitted lines have different slopes, suggesting a minor sensitivity of the salting-out effect on the pressure. The slopes of the fitted lines in Figure 10c,d fall between [−0.11, −0.19]. The solubility of H_2_ reduces, at any chosen pressure and temperature, by ca. 15% when a mole of NaCl is added to pure water results. A subsequent rise in salt concentration to 2 mol NaCl/kg H_2_O results in a further reduction of CO_2_ by another ca. 15%.

The mole fraction of water in the gas phase () at 323.15 and 423.15 K is plotted in Figure 12e,f. Figure 12e,f clearly shows that the water content in the gas phase at 423.15 K can be 20 to 40 times higher compared to 323.15 K. Similar to the CO_2_-NaCl brine system, the salt concentration in brine appears to have little or negligible effect on the quantity of water in the gas phase. Figure 12e,f also show that the water content in the gas-rich phase decreases with pressure. The fugacity coefficients of H_2_ and H_2_O obtained from MC simulations (eq 6) closely align with REFPROP predictions, showing agreement within a 5% margin for identical H_2_-H_2_O mixtures at various compositions, pressures, and temperatures. Notably, ϕ_H_2__ consistently surpasses unity, indicating prevalent repulsive interactions among H_2_ molecules in the gas phase. This contrasts with the fugacity coefficients for CO_2_ in CO_2_-NaCl brine systems, which are below 1, suggesting attractive interactions between CO_2_ molecules in the gas phase. Additional plots pertaining to the effect of temperature, pressure, and salt concentration on the VLE H_2_-NaCl brine systems is shown in Figures S35–S37 of the Supporting Information.

### 3.8 Phase Equilibria of CO_2_-H_2_-NaCl Brine Mixtures

The phase equilibria of H_2_-CO_2_-NaCl brine systems are investigated to compare the solubilities of CO_2_ and H_2_ in pure water and NaCl brine with their solubilities in binary systems: CO_2_-NaCl brine and H_2_-NaCl. The dissolution of CO_2_ and H_2_ in NaCl brine is examined as a function of fugacity rather than pressure, as fugacity serves as a measure of the chemical potential responsible for gas dissolution in water. The fugacity of component *i* (*f*_*i*_), where *i* is either CO_2_ or H_2_, is^52,53^
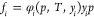
13where ϕ_*i*_ is the fugacity coefficient, as defined in eq 6, *y*_*i*_ is the mole fraction of species *i* in the gas phase, and *p* is the pressure.

**Figure 11:**
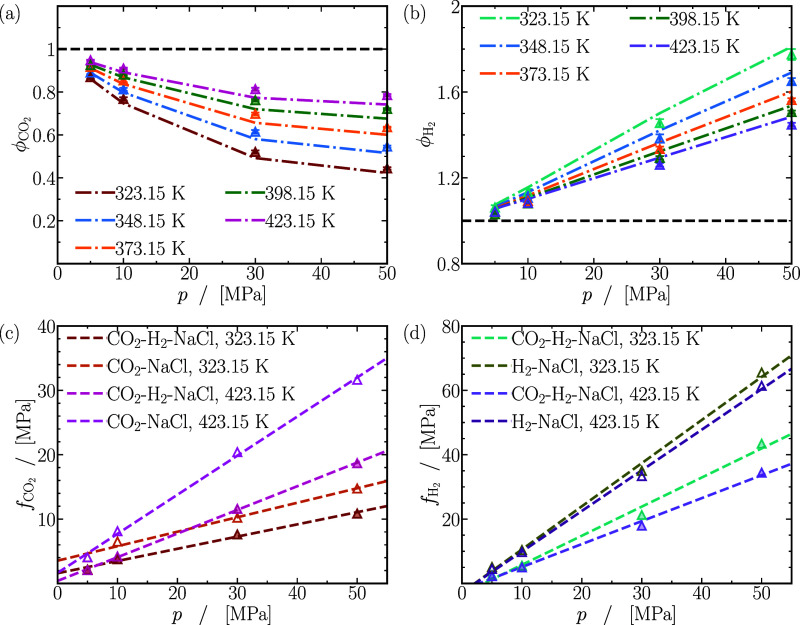
Fugacity coefficients and fugacities of CO_2_ and H_2_ in the CO_2_-H_2_-NaCl brine system computed from MC simulations using eq 6. Fugacity coefficients of (a) CO_2_ and (b) H_2_ in the gas-rich phase compared to predictions from REFPROP^108^ based on the gas-phase compositions from MC simulations depicted by dashed lines. (c,d) Fugacities of CO_2_ and H_2_ computed from eq 13 using fugacity coefficients from (a,b), respectively. The dashed lines are linear fits to the data points. The open symbols represent fugacities computed for CO_2_ and H_2_ in the respective binary systems of CO_2_-NaCl and H_2_-NaCl, respectively. The raw data of the fugacity coefficients are provided in Table S7 of the Supporting Information.

The fugacity coefficients and the fugacities of CO_2_ and H_2_ are shown in Figure 11a–d as a function of pressure between 323.15 and 423.15 K along the VLE of CO_2_-H_2_-NaCl brine. In Figure 11a, the fugacity coefficients of CO_2_ behave similarly to those in the CO_2_-NaCl brine system. Specifically, ϕ_CO_2__ decreases with pressure but increases with temperature. Computed fugacity coefficients derived from gas phase compositions along the VLE of CO_2_-H_2_-NaCl brine agree within ca. 5–10% of the REFPROP predictions. In Figure 11c, fugacities of CO_2_ computed using eq 13 vary linearly with pressure, as indicated by the fits at 323.15 and 423.15 K. Unlike CO_2_, the fugacity coefficients of H_2_ shown in Figure 11b exhibit an increase with pressure but a decrease with temperature, qualitatively reproducing its behavior in the binary mixture of H_2_-NaCl brine. Fugacities of H_2_ computed using eq 13 vary linearly with pressure, as indicated by the fits at 323.15 and 423.15 K in Figure 11d. To facilitate comparison, open symbols representing the fugacities of CO_2_ and H_2_ in the binary systems of CO_2_-NaCl and H_2_-NaCl are plotted alongside the ternary system in Figure 11c,d. The fugacities of CO_2_ and H_2_ in the ternary CO_2_-H_2_-NaCl systems are lower than those in their respective binary systems. For instance, in Figure 11c, the fugacity of CO_2_ in the ternary system at 50 MPa and 423.15 K is lower by ca. 45% than *f*_CO_2__ in the binary system. Similarly, in the case of H_2_ in Figure 11d, the fugacities in the ternary system are lower by ca. 40% at 50 MPa and 423.15 K.

**Figure 12:**
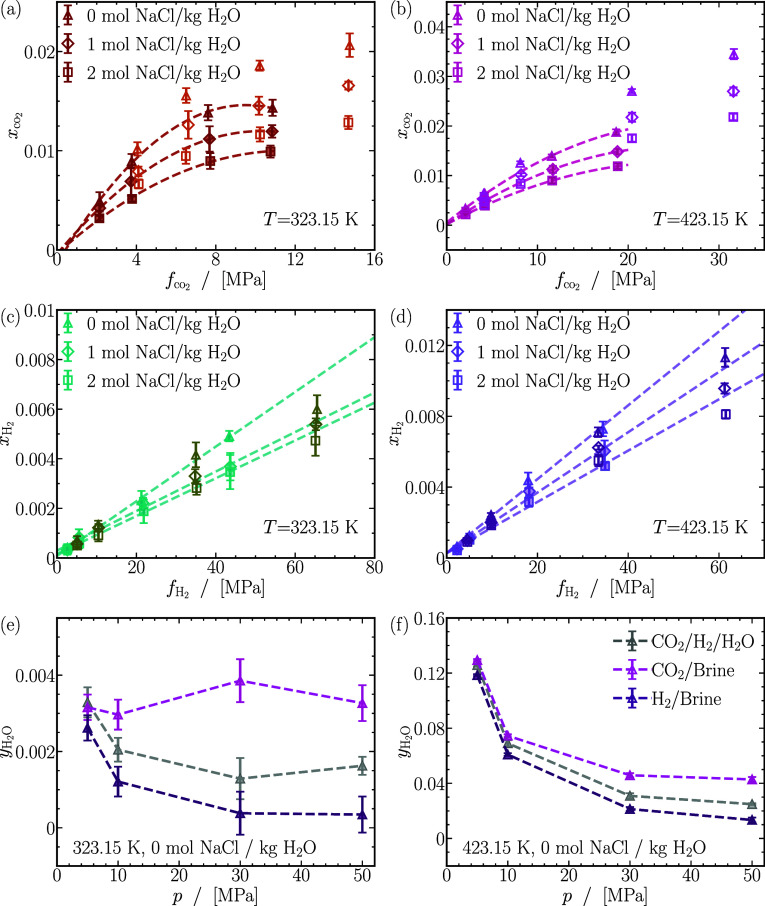
Mole fractions of CO_2_ (*x*_CO_2__) and H_2_ (*x*_H_2__) in the liquid-rich phase are presented for the H_2_-CO_2_-NaCl system (closed symbols) and compared with the CO_2_-NaCl and H_2_-NaCl systems (open symbols). (a,b) *x*_CO_2__ as a function of the fugacity of CO_2_ in the gas-rich phase at 323.15 and 423.15 K, respectively. Quadratic fits for *x*_CO_2__ in the H_2_-CO_2_-NaCl system are represented by dashed lines. (c,d) serve the same purpose for H_2_. (e,f) Comparison of the water content in the CO_2_-H_2_-H_2_O (ternary) system with the CO_2_-H_2_O and H_2_-H_2_O (binary) systems at 323.15 and 423.15 K. The raw data of the solubilities are provided in Table S6 of the Supporting Information.

The solubilities of CO_2_ and H_2_ in NaCl brine solutions are shown in Figure 12a–d, comparing binary and ternary systems. The solubility of CO_2_ varies quadratically with the fugacity of CO_2_ in both binary and ternary systems, as shown in Figure 12a. In the low-fugacity regime (*f*_CO_2__ ≲ 4 MPa), the solubilities of CO_2_ in NaCl brine are similar for the binary and ternary systems. For intermediate and large fugacities (*f*_CO_2__ ≳ 4 MPa), the solubility of CO_2_ in NaCl brine becomes substantially different in the binary and ternary systems. Specifically, CO_2_ solubility in the ternary system is lower compared to the binary system at (approximately) the same fugacity. An even stronger suppression in the dissolution of CO_2_ occurs at 423.15 K, as shown in Figure 12b. The solubilities of H_2_ in NaCl brine shown in Figure 12c,d is analyzed next. In the low-fugacity regime (*f*_H_2__ ≲ 40 MPa), the solubilities of H_2_ are nearly identical in the binary and ternary systems. Differences in the solubilities arise for *f*_H_2__ ≳ 40, wherein a greater H_2_ dissolution is evident in the ternary case when compared to the H_2_-NaCl brine system. The enhanced dissolution occurs also at 423.15 K, as shown in Figure 12d. In contrast to CO_2_, the solubility of H_2_ as a function of *f*_H_2__ is enhanced in ternary systems.

Figure 12e,f show the water content in the gas phase for both binary and ternary systems. The water content in the gas phase varies negligibly with the salt concentration in brine. In Figure 12e at 323.15 K, the mole fraction of H_2_O in the gas phase decreases with pressure for CO_2_-H_2_-H_2_O systems. As expected, the mole fraction of water in the ternary system falls between those of the CO_2_-H_2_O and H_2_-H_2_O binary systems. At 423.15 K, the water content in the gas phase is higher, yet it follows a trend similar to that at 323.15 K. In conclusion, an enhanced dissolution of water is observed compared to the H_2_-brine system but suppression compared to the CO_2_-brine system.

## 4 Conclusions

Understanding the thermodynamic behavior of mixtures containing H_2_ and CO_2_ is essential for addressing hydrogen losses in underground hydrogen storage within depleted gas fields. The loss of purity of H_2_ resulting from molecular diffusion follows Fick’s law, highlighting the importance of knowing the Fick diffusion coefficients for a variety of temperatures, pressures, and mixture compositions. The dissolution of H_2_ in salt water, particularly in the presence of CO_2_, presents another avenue for hydrogen loss. Thus, a comprehensive knowledge of the Fick diffusion coefficients for H_2_-CO_2_ mixtures and their phase equilibria with NaCl brine is crucial. The thermodynamic conditions relevant to subsurface hydrogen storage typically span temperatures of 323.15 to 423.15 K, pressures ranging from 5 to 50 MPa, and salt concentrations of 1 to 2 mol of NaCl/kg of H_2_O.

The Fick diffusivities of H_2_-CO_2_ mixtures were investigated via MD simulations at storage-relevant conditions. At low pressures (5 Mpa), gas-like diffusivities of ca. 10^–6^ m^2^/s remained composition-independent. The kinetic theory accurately predicted these diffusivities within 5–10% at 423.15 K, with deviations of up to 20% at 323.15 K. At high pressures (40–50 Mpa), kinetic theory predictions deviated up to 75% for CO_2_-rich mixtures and around 15% for hydrogen-enriched ones. Approaches based on kinetic theory, such as the Fuller correlation^30−32^ and the corresponding-states approaches,^11,13^ yielded similar results. Kinetic theory-based methods can predict Fick diffusivities within 15% for H_2_-CO_2_ mixtures exhibiting near-ideal gas behavior (1) at low pressures (ca. 5 Mpa) and high temperatures (423.15 K) and (2) for H_2_-enriched mixtures (mole fraction of H_2_ ≳ 0.8). Moggridge correlation^122,130−134^ provides an alternative to accurately predicting diffusion coefficients within 5–10% at high pressures (40–50 MPa) and low temperatures (*T* ≲ 348.15 K) for CO_2_-rich mixtures, making it suitable for the high-pressure regime. For intermediate pressures (10–35 MPa), molecular simulations are preferred. The efficacy of the Moggridge correlation stems from the fluid-like behavior of mixtures at high pressures. Mixtures at 323.15 K and pressures of 15–25 MPa exhibit oscillatory trends in Fick diffusivities due to the proximity of CO_2_ to its critical point.

Phase equilibria of CO_2_-H_2_-NaCl mixtures are examined using CFCMC simulations showing close agreement with experimental results for CO_2_ solubilities in NaCl brines. The Setschenow equation^140^ used to describe the salting-out effect of CO_2_ captures the exponentially decreasing solubilities of CO_2_ in the liquid-rich phase with an increasing concentration of NaCl in brine. The water content in the gas phase decreased with pressure at 423.15 K but remained nearly constant at 323.15 K, regardless of salt concentration. H_2_ solubilities in brine followed the Henry regime, varying linearly with pressure. The impact of salt concentration on H_2_ solubility relative to pure water is quantified using the Setschenow equation. Similar to the CO_2_-NaCl brine system, water concentrations in the gas phase consistently decrease with pressure at both 323.15 and 423.15 K. Ternary systems of H_2_-CO_2_-NaCl brine mixtures revealed a suppressed CO_2_ solubility and an enhanced H_2_ solubility, compared to the solubilities in the respective binary mixtures. The water content in the gas phase is enhanced compared to H_2_-NaCl brine systems but suppressed compared to CO_2_-NaCl brine systems.

## Supporting Information Available

Mixture densities, mixture compressibility factors, mixture viscosities, self-diffusion coefficients, finite system-size effects on self-diffusion coefficients, self-diffusivities, ratio of self-diffusion coefficients, ratio of self-diffusivities, effect of temperature on self-diffusion coefficients, density dependence of self-diffusion coefficients, Maxwell-Stefan diffusion coefficients from cross-correlations of displacements, finite-size effects for the mutual diffusion coefficients, Fick diffusion coefficients, effect of mixture composition and temperature on Fick diffusion coefficients, principle of corresponding states for Fick diffusivities, effect of pressure, temperature, and NaCl concentration on the solubility, force field parameters, densities, compressibilities, and total energies from MD simulations, viscosities, thermodynamic factors, and self, Maxwell-Stefan, and Fick diffusion coefficients, phase equilibria, and fugacity coefficients (PDF)
