# Supplementary material for: Mutual Diffusivities of Mixtures of Carbon Dioxide and Hydrogen and Their Solubilities in Brine: Insight from Molecular Simulations
Source: Ind Eng Chem Res. 2024 May 31;63(23):10456–81. doi: 10.1021/acs.iecr.4c01078 (PMC11177264; doi:10.1021/acs.iecr.4c01078)
Supplement: Supplementary file 1 — ie4c01078_si_001.pdf [file ie4c01078_si_001.pdf]

Supporting Information for: Mutual diffusivities of mixtures of carbon dioxide and hydrogen and their solubilities in brine: Insight from molecular simulations

Thejas Hulikal Chakrapani,[†] Hadi Hajibeygi,[†] Othonas A. Moultos,[‡] and Thijs J. H. Vlugt[*,‡]

†*Reservoir Engineering, Geoscience and Engineering Department, Faculty of Civil Engineering and Geosciences, Delft University of Technology, 2628 CN Delft, The Netherlands*

‡*Engineering Thermodynamics, Process and Energy Department, Faculty of Mechanical Engineering, Delft University of Technology, 2628 CB Delft, The Netherlands;*

E-mail: t.j.h.vlugt@tudelft.nl

List of Figures

S1 H_2-CO_2 mixture densities as a function of x_{H_2} for $p \in [10, 30]$ MPa S5
S2 H_2-CO_2 mixture densities as a function of x_{H_2} for $p \in [35, 45]$ MPa S6
S3 H_2-CO_2 mixture densities as a function of p for $x_{H_2} \in [0, 0.5]$ S7
S4 H_2-CO_2 mixture densities as a function of p for $x_{H_2} \in [0.6, 1]$ S8
S5 H_2-CO_2 mixture densities: MD vs. REFPROP, $p \in [5, 15]$ MPa S9
S6 H_2-CO_2 mixture densities: MD vs. REFPROP, $p \in [20, 30]$ MPa S10
S7 H_2-CO_2 mixture densities: MD vs. REFPROP, $p \in [35, 50]$ MPa S11
S8 H_2-CO_2 mixture compressibility factors for $x_{H_2} \in [0, 0.4]$ S13
S9 H_2-CO_2 mixture compressibility factors for $x_{H_2} \in [0.5, 1]$ S14
S10 H_2-CO_2 mixture compressibility factors for $p \in [5, 50]$ MPa S15
S11 H_2-CO_2 mixture viscosity determination from MSDs S17
S12 H_2-CO_2 mixture viscosities as a function of x_{H_2} for $p \in [10, 30]$ MPa S18
S13 H_2-CO_2 mixture viscosities as a function of x_{H_2} for $p \in [35, 45]$ MPa S19
S14 H_2-CO_2 mixture viscosities as a function of p for $x_{H_2} \in [0.1, 0.4]$ S20
S15 H_2-CO_2 mixture viscosities as a function of p for $x_{H_2} \in [0.6, 1]$ S21
S16 Self-diffusion coefficients from MSDs in a H_2-CO_2 mixture. S23
S17 Finite system-size effects on self-diffusion coefficients: low densities S25
S18 Finite system-size effects on self-diffusion coefficients: high densities S25
S19 Self-diffusivities of CO_2 and H_2 . . . . S26
S20 Ratio of self-diffusion coefficients of CO_2 and H_2 . . . . S28
S21 Ratio of self-diffusivities of CO_2 and H_2 relative to pure components, I . . . S29
S22 Ratio of self-diffusivities of CO_2 and H_2 relative to pure components, II . . . S30
S23 Effect of temperature on self-diffusion coefficients of CO_2 and H_2 . . . . S31
S24 Density dependence of self-diffusion coefficients of CO_2 and H_2, I S32

S25 Density dependence of self-diffusion coefficients of CO_2 and H_2, II S33
S26 Maxwell-Stefan diffusion coefficients from cross-correlations of displacements. S36
S27 Finite-size effects for the mutual diffusion coefficients S37
S28 Fick diffusion coefficients of H_2-CO_2 mixtures for $p \in [15, 45]$ MPa S38
S29 Effect of mixture composition on Fick diffusion coefficients S39
S30 Effect of temperature on Fick diffusion coefficients S40
S31 Principle of corresponding states for Fick diffusivities S41
S32 Effect of pressure on the solubility of CO_2, CO_2-NaCl brine S42
S33 Effect of temperature on the solubility of CO_2, CO_2-NaCl brine S42
S34 Effect of NaCl concentration on the solubility of CO_2 in NaCl brine S43
S35 Effect of pressure on the solubility of H_2, H_2-NaCl brine S44
S36 Effect of temperature on the solubility of H_2, H_2-NaCl brine S44
S37 Effect of NaCl concentration on the solubility of H_2, H_2-NaCl brine S45

List of Tables

S1 Force field parameters used in this study S46
S2 Densities, compressibilities, and total energies from MD simulations. S47
S3 Viscosities, thermodynamic factors, and self, Maxwell-Stefan and Fick diffusion coefficients. S72
S4 Phase equilibria of CO_2-NaCl brine systems from CFCMC simulations. . . . S97
S5 Phase equilibria of H_2-NaCl brine systems from CFCMC simulations. S99
S6 Phase equilibria of H_2-CO_2-NaCl brine systems from CFCMC simulations. . S101
S7 Fugacity coefficients of H_2,CO_2 and H_2O in H_2-CO_2-NaCl brine systems . . . S104

S1 Mixture Densities for H_2-CO_2 mixtures

S1.1 Preparation of systems for production runs in the NVE ensemble

The procedure to prepare systems for production runs in the NVE ensemble using systems consisting of 2000 molecules is described below. To avoid overlaps between molecules, binary mixtures of H_2 and CO_2, simulated as rigid bodies, are placed inside a large cubic simulation box ($\approx$ 100 Å). Simulations of 2000 molecules are performed in the isotropic version of the NPT ensemble using the Nosé-Hoover barostat and thermostat as described originally by Martyna *et al.*[1] and Kamberaj *et al.*[2], using damping constants of 1 and 0.1 ps, respectively. In the initialization phase, the time-step is gradually increased from 10^{-4} (fs) towards a final value of 0.5 fs, to allow the system to relax towards equilibrium. Following the initialization phase lasting 12.5 ps, a production phase lasting 250 ps ensues during which the density of the system is recorded every time step. The NPT production phase is followed by a short simulation in the NVT ensemble lasting 50 ps. In this phase, the size of the simulation box is uniformly scaled at a constant rate, until it reaches a size that aligns with the average density obtained in the NPT production phase. The simulations are continued with a fixed box size for 25 ps (initialization phase) and 250 ps (production phase) in the NVT ensemble, where the Nosé-Hoover thermostat is used with a damping constant of 0.1 ps. During the production phase in the NVT ensemble, the average total energy is calculated, which is then used to scale the molecule velocities to achieve a desired temperature in the NVE ensemble. After scaling the velocities of all molecules, the system is simulated in the NVE ensemble for 25 ps (initialization phase) and 250 ps (production phase). During the production phase the temperature, pressure and the total energy of the system are recorded and checked for conservation. The raw data for the simulations of the larger systems are provided in Table S2.

S1.2 Mixture density vs mixture composition

H_2-CO_2 mixture densities for p between 10 and 45 MPa, and T between 323.15 and 423.15 K, in increments of 25 K are plotted in Figs. S1-S2. Average densities obtained from MD simulations in the NPT ensemble (symbols), are compared to the corresponding values from the thermodynamic database REFPROP[3] (solid lines).

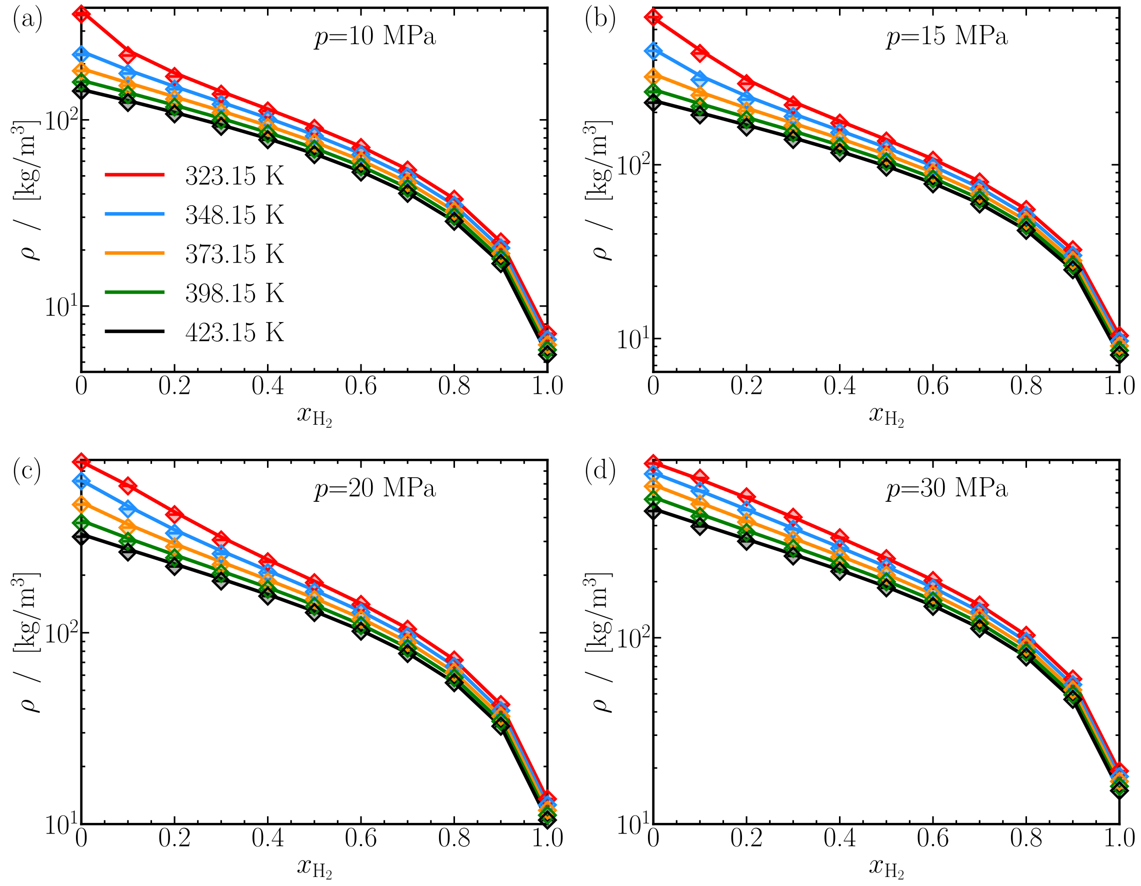

Figure S1: H_2-CO_2 mixture densities (ρ) at 10, 15, 20 and 30 MPa as a function of the hydrogen mole fraction x_{H_2}, and temperatures between 323.15 K and 423.15 K. Comparison between MD simulations (symbols) and REFPROP[3] (solid lines). Error bars are smaller than the symbols. Lines are colored as per the legend in subfigure (a).

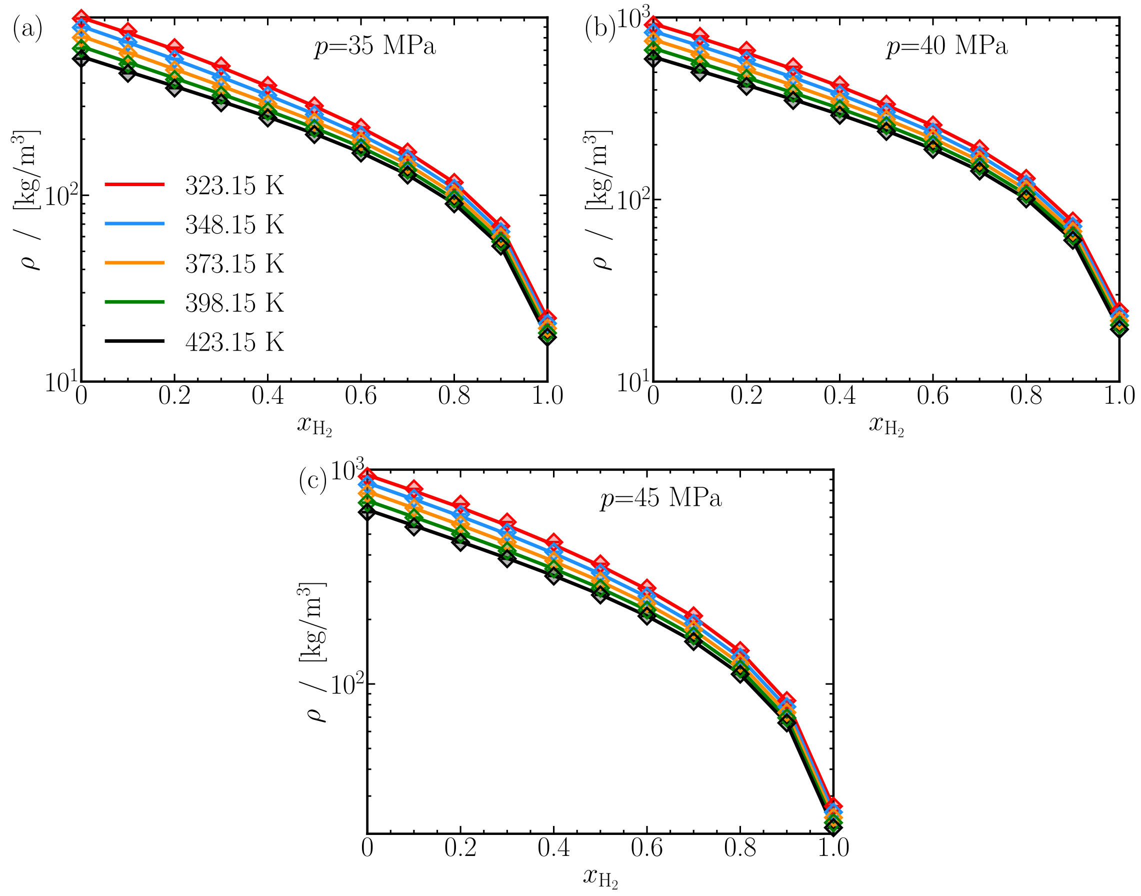

Figure S2: H_2-CO_2 mixture densities (ρ) at 35, 40, and 45 MPa as a function of the hydrogen mole fraction x_{H_2}, and temperatures between 323.15 K and 423.15 K. Comparison between MD simulations (symbols) and REFPROP[3] (solid lines). Error bars are smaller than the symbols. Lines are colored as per the legend in subfigure (a). See the caption of Fig. S1.

S1.3 Mixture density vs pressure

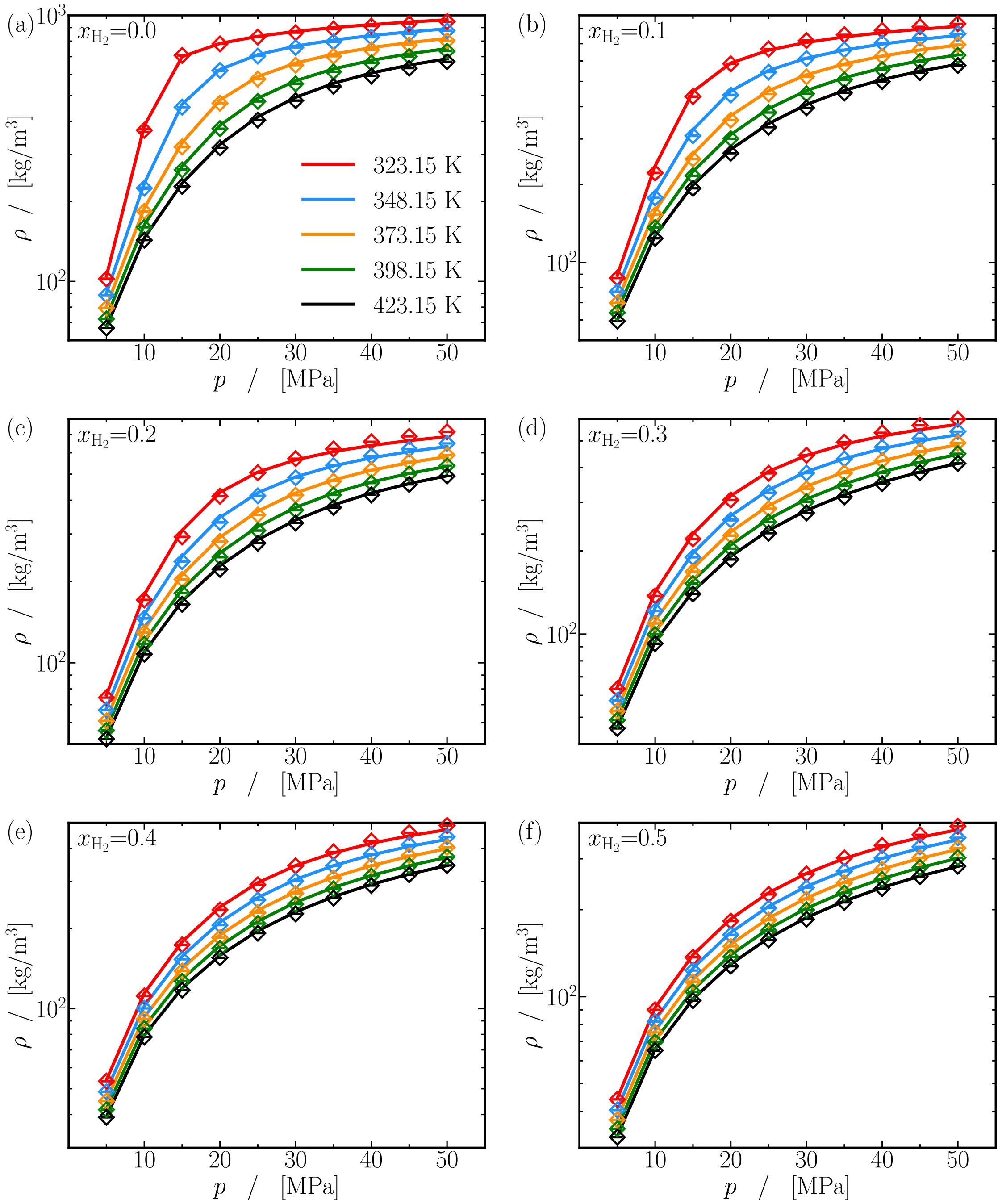

Figure S3: H_2-CO_2 mixture densities (ρ) for different hydrogen mole fractions between 0 and 0.5, as a function of the pressure p, and temperatures between 323.15 K and 423.15 K. Comparison between MD simulations (symbols) and REFPROP[3] (solid lines). Error bars are smaller than the symbols. Lines are colored as per the legend in subfigure (a).

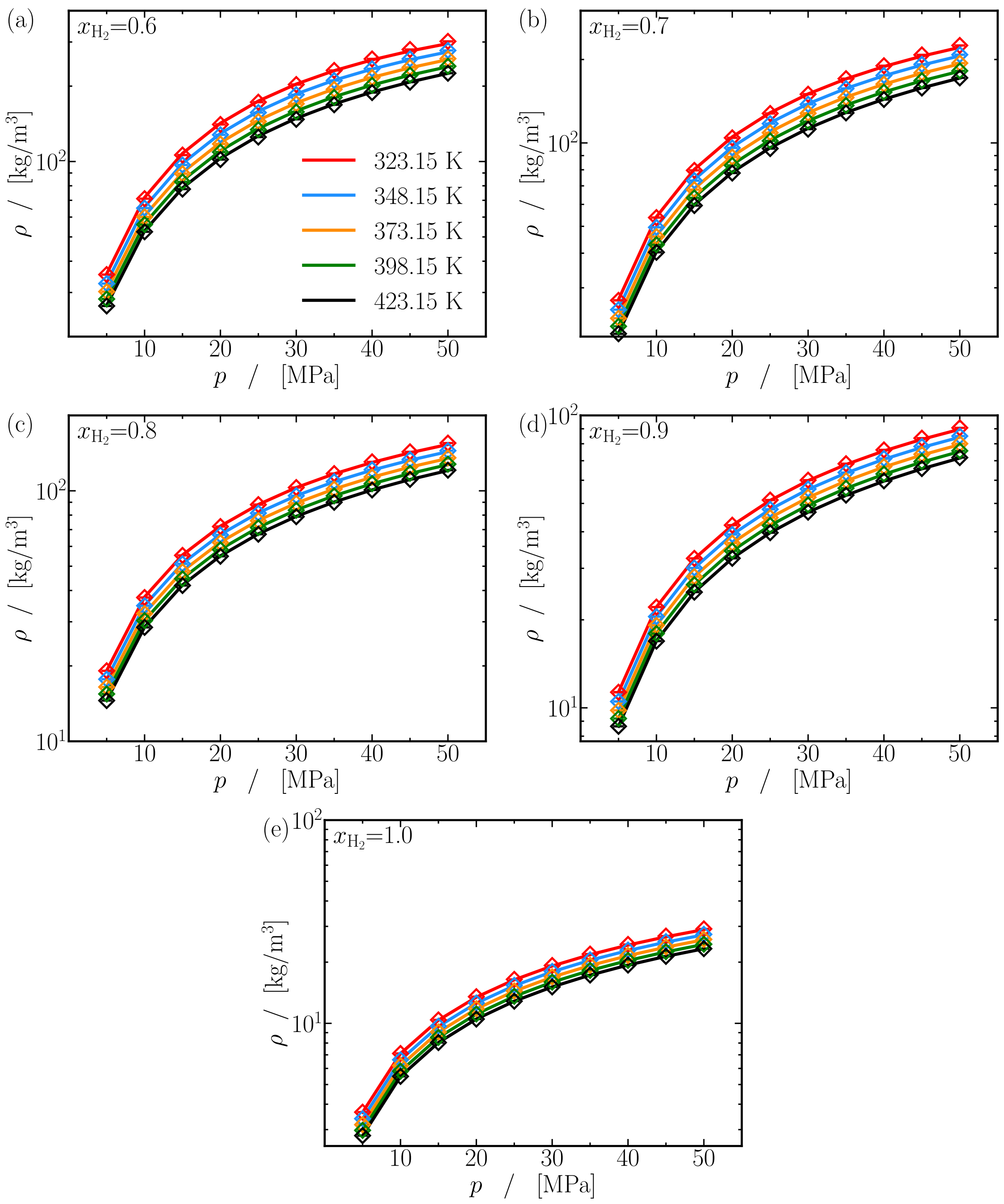

Figure S4: H_2-CO_2 mixture densities (ρ) for different hydrogen mole fractions between 0.6 and 1, as a function of the pressure p, and temperatures between 323.15 K and 423.15 K. Comparison between MD simulations (symbols) and REFPROP[3] (solid lines). Error bars are smaller than the symbols. Lines are colored as per the legend in subfigure (a). See the caption of Fig. S3.

S1.4 Relative deviations between simulations and REFPROP

Relative deviations, expressed as percentage, between the densities computed from MD simulations in the NPT ensemble and those acquired from the REFPROP[3] database are displayed in Figs. S5-S7.

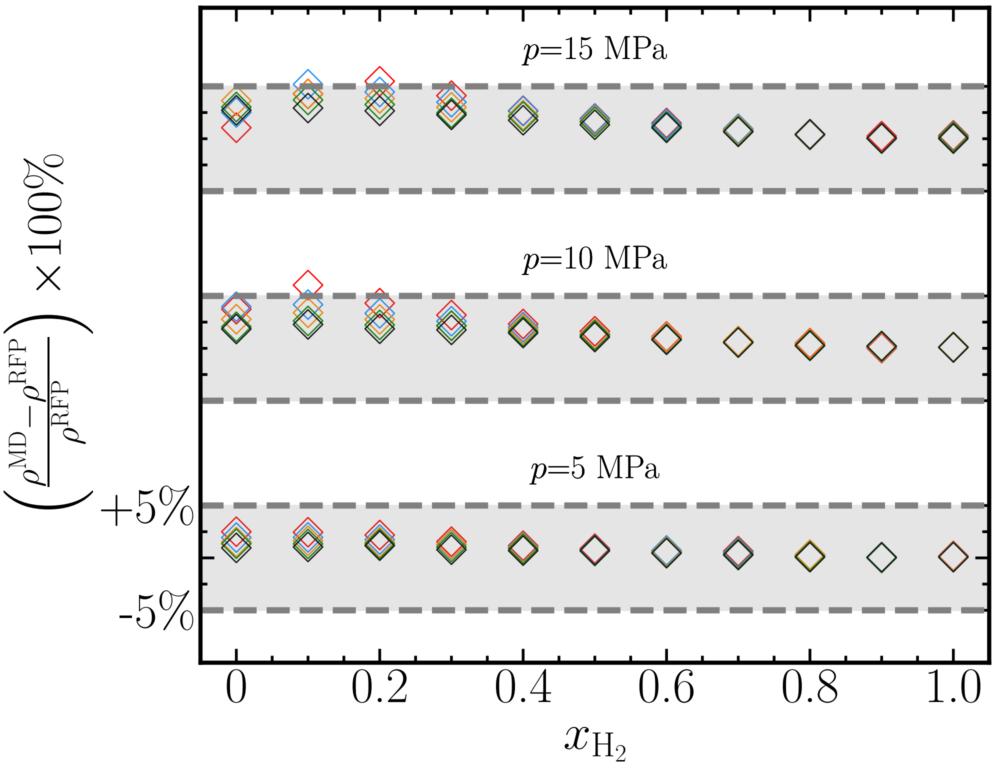

Figure S5: Relative deviations of the CO_2-H_2 mixture densities obtained from MD simulations (ρ^{MD}) from the REFPROP[3] database (ρ^{RFP}), plotted for $p \in [5, 15]$ MPa, as a function of $x_{\mathrm{H_2}}$ in the mixture. At each pressure the symbols are color coded (see Fig. S1) for T between 323.15 K and 423.15 K. The grey dashed lines indicate deviations of $\pm 5\%$.

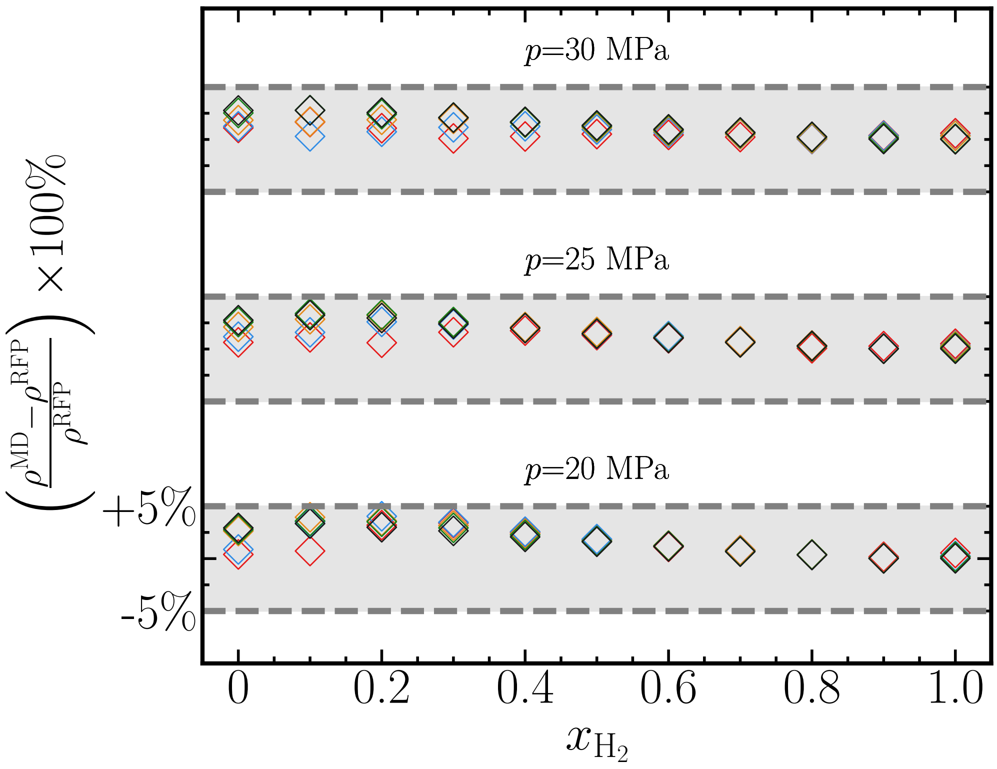

Figure S6: Relative deviations of the CO_2-H_2 mixture densities obtained from MD simulations (ρ^{MD}) from the REFPROP[3] database (ρ^{RFP}), plotted for $p \in [20, 30]$ MPa, as a function of x_{H_2} in the mixture. At each pressure the symbols are color coded (see Fig. S1) for T between 323.15 K and 423.15 K. The grey dashed lines indicate deviations of ±5%. See Fig. S5.

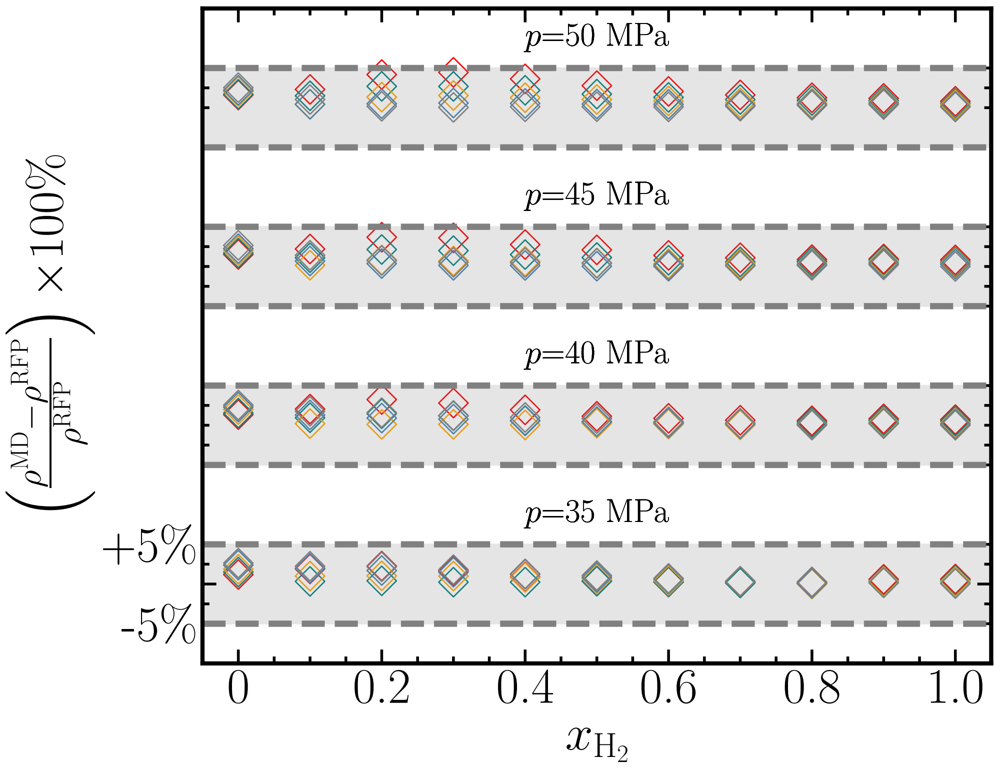

Figure S7: Relative deviations of the CO_2-H_2 mixture densities obtained from MD simulations (ρ^{MD}) from the REFPROP[3] database (ρ^{RFP}), plotted for $p \in [5\ ,\ 15]$ MPa, as a function of x_{H_2} in the mixture. At each pressure the symbols are color coded (see Fig. S1) for T between 323.15 K and 423.15 K. The grey dashed lines indicate deviations of ±5%. See Fig. S5.

S2 Compressibility Factors for H_2-CO_2 mixtures

The compressibility factor Z of a mixture is defined as,

$$Z = \frac{pV}{N_{\text{tot}} k_B T} \tag{S1}$$

where p is the pressure, V is the average volume of the simulation box, N_{tot} is the total number of molecules in the simulation box, k_B is the Boltzmann constant, and T is the absolute temperature. The variation of Z with p is plotted in Figs. S8-S9, and $Z(x_{\text{H}_2})$ is shown in Fig. S10.

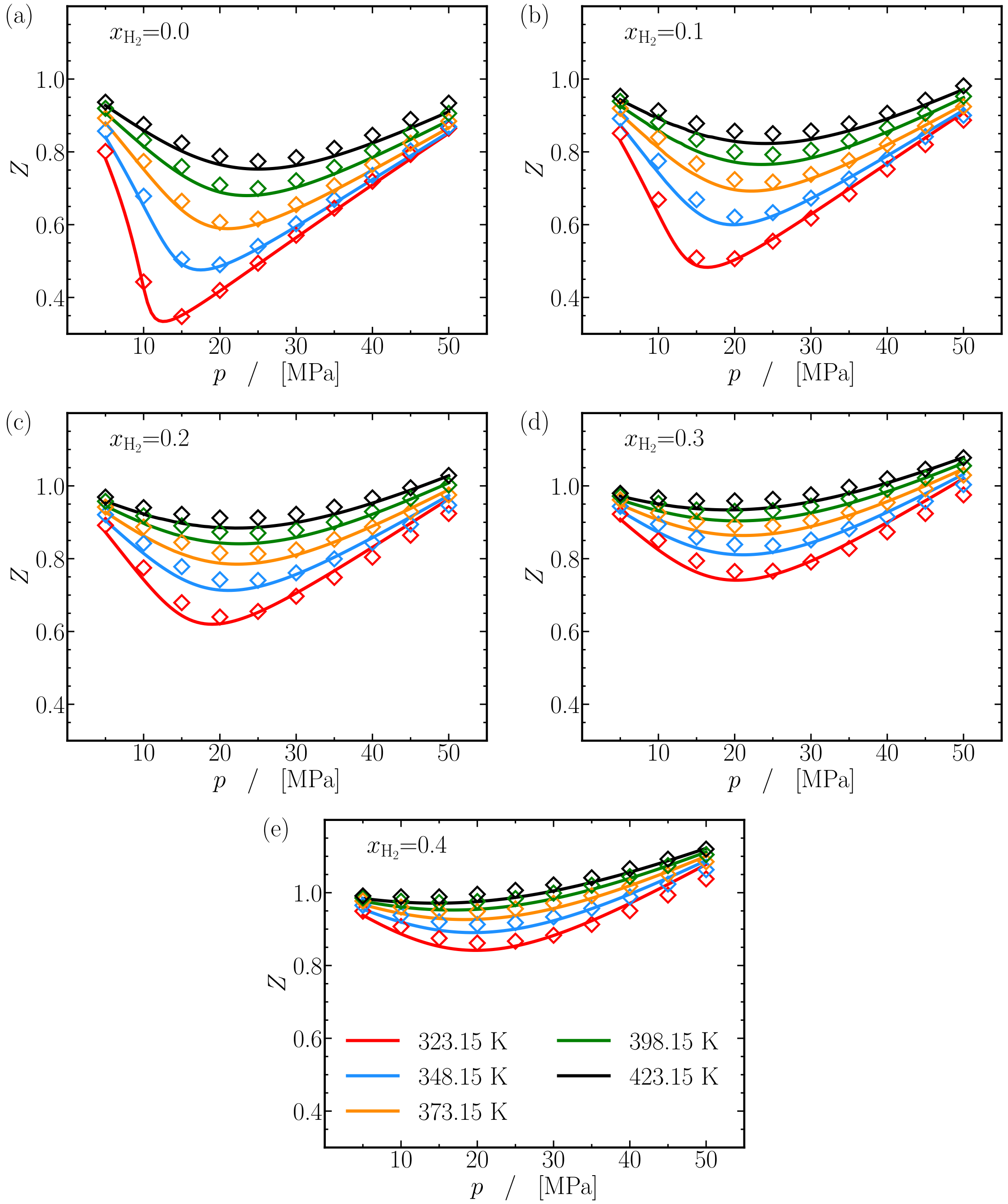

Figure S8: Compressibility factors (Z) of H_2-CO_2 mixtures for $x_{H_2} \in [0, 0.4]$ and temperatures between 323.15 K and 423.15 K. Comparison between MD simulations (symbols) and REFPROP predictions (lines). Error bars are smaller than the symbols. Lines are colored according to the legend in subfigure (e).

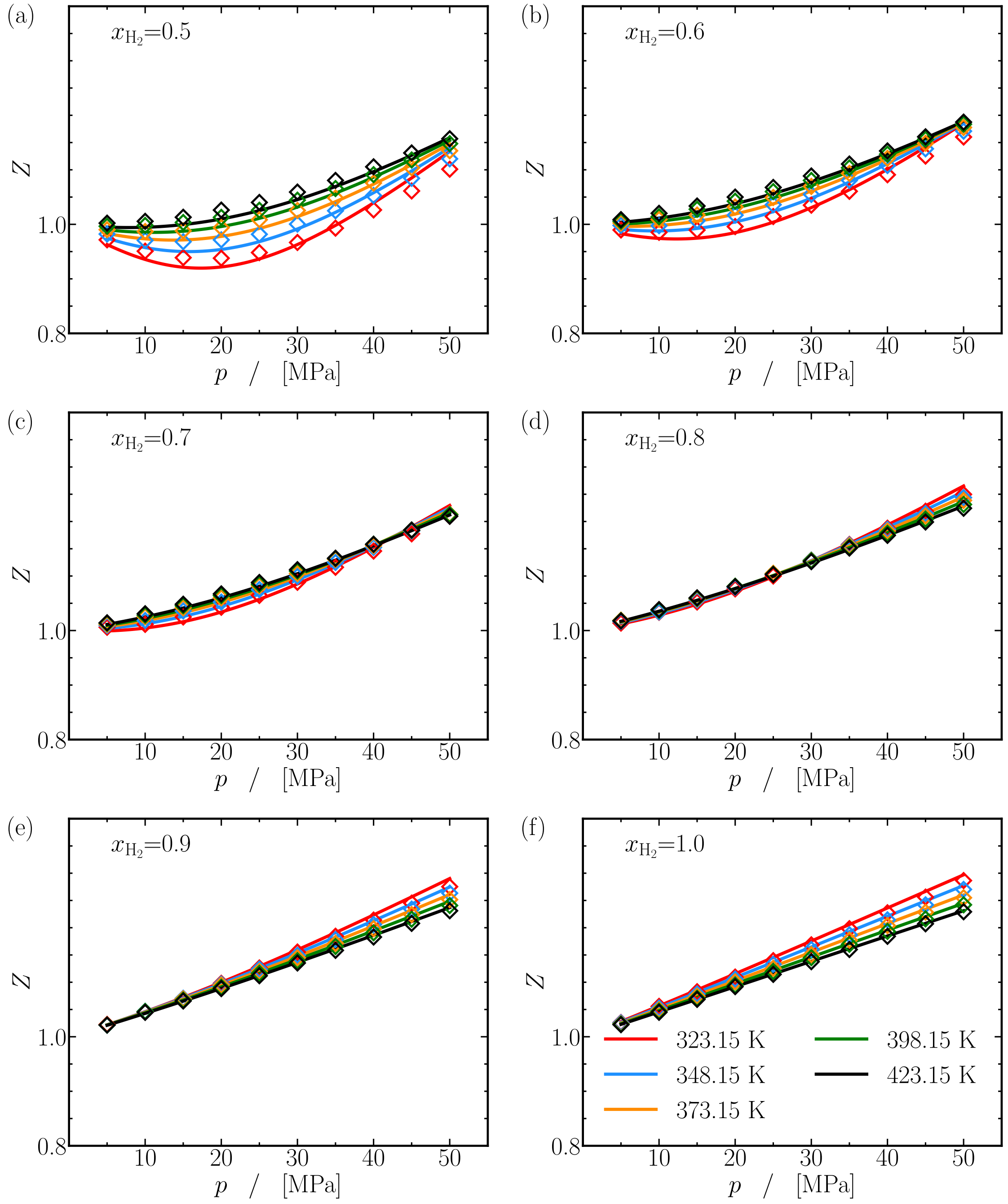

Figure S9: Compressibility factors (Z) of H_2-CO_2 mixtures for $x_{H_2} \in [0.5, 1]$ and temperatures between 323.15 K and 423.15 K. Comparison between MD simulations (symbols) are REFPROP predictions (lines). Error bars smaller than the symbols. Lines are colored according to the legend in subfigure (e). See Fig. S8.

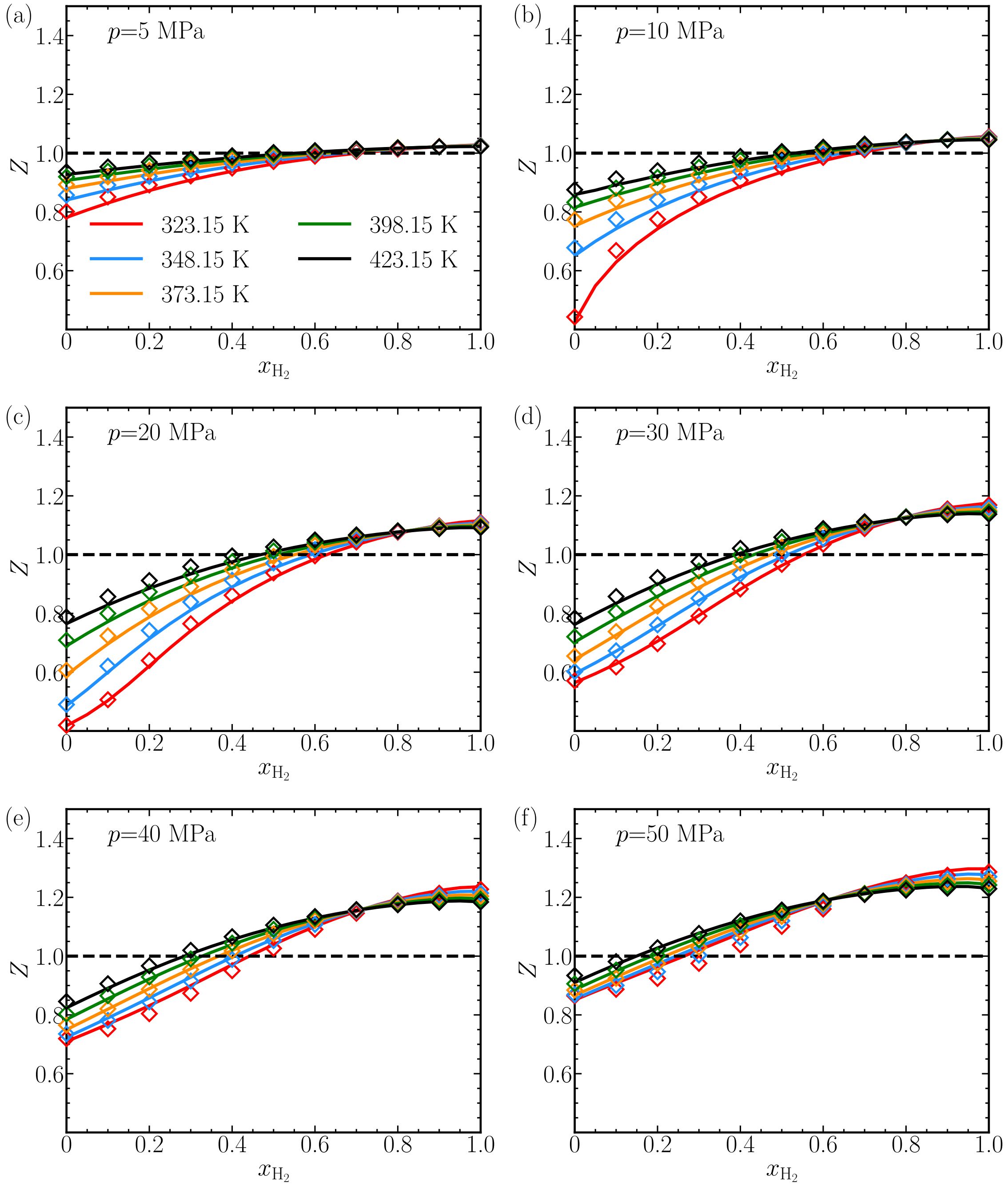

Figure S10: Compressibility factors (Z) of H_2-CO_2 mixtures plotted as a function of x_{H_2}. Comparison between MD simulations (symbols) are REFPROP predictions (lines). Error bars smaller than the symbols. Lines are colored according to the legend in subfigure (a). See also Figs. S8 and S9.

S3 Viscosities of H_2-CO_2 mixtures

The viscosities are computed from the time integral of the autocorrelation function of all components of the traceless pressure tensor[4],

$$\eta = \lim_{t\to\infty} \left[\frac{1}{10 \cdot 2t} \frac{V}{k_B T} \left\langle \sum_{\alpha\beta} \left(\int_0^t dt' p_{\alpha\beta}^{\mathrm{Tr}}(t') \right)^2 \right\rangle \right] \tag{S2}$$

$$p_{\alpha\beta}^{\mathrm{Tr}} = \frac{p_{\alpha\beta} + p_{\beta\alpha}}{2} - \delta_{\alpha\beta} \left(\frac{1}{3} \sum_k p_{kk} \right) \tag{S3}$$

where t is time, V is the volume of the simulation box, k_B is the Boltzmann constant, T is the temperature, $p_{\alpha\beta}^{\mathrm{Tr}}$ are the components of a traceless tensor derived from the pressure tensor $p_{\alpha\beta}$ using Eq. S3, $\delta_{\alpha\beta}$ is the Kronecker delta, and $\langle \cdots \rangle$ indicates an ensemble average. η is calculated by evaluating the slope of the linear segment of the so-called mean-square displacement, shown within the brackets $[\cdots]$ in Eq. S2. For calculation of viscosities, the mean square displacements of Eq. S2 are plotted on a log-log scale as a function of time, and the segment with a slope between 0.99 and 1.01 is chosen for computing η, see Fig. S11.

S3.1 Determination of viscosities from MD simulations

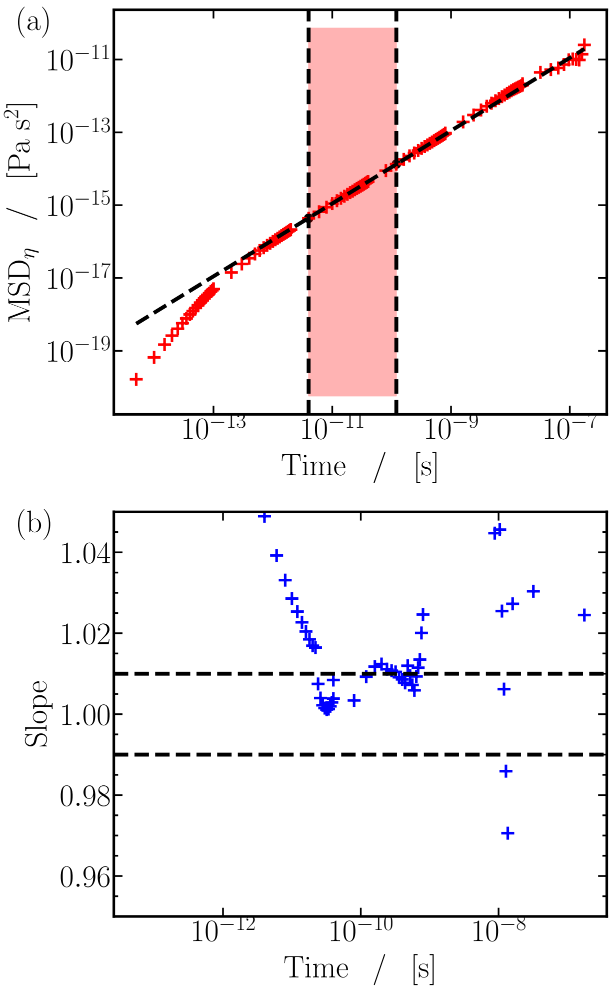

Figure S11: (a) Mean-squared displacements (symbols) of η (MSD_η) calculated using Eq. S2, as a function of time for pure CO_2 at 50 MPa and 323.15 K. The black dashed line is a linear fit of the data within the highlighted region in red. (b) Slopes of the MSDs, calculated using 10 successive points, of the points falling within the shaded region have a slope between 0.99 and 1, and are used for the calculation of viscosities. We follow the same procedure to calculate viscosities of mixtures.

S3.2 Viscosity vs mixture composition

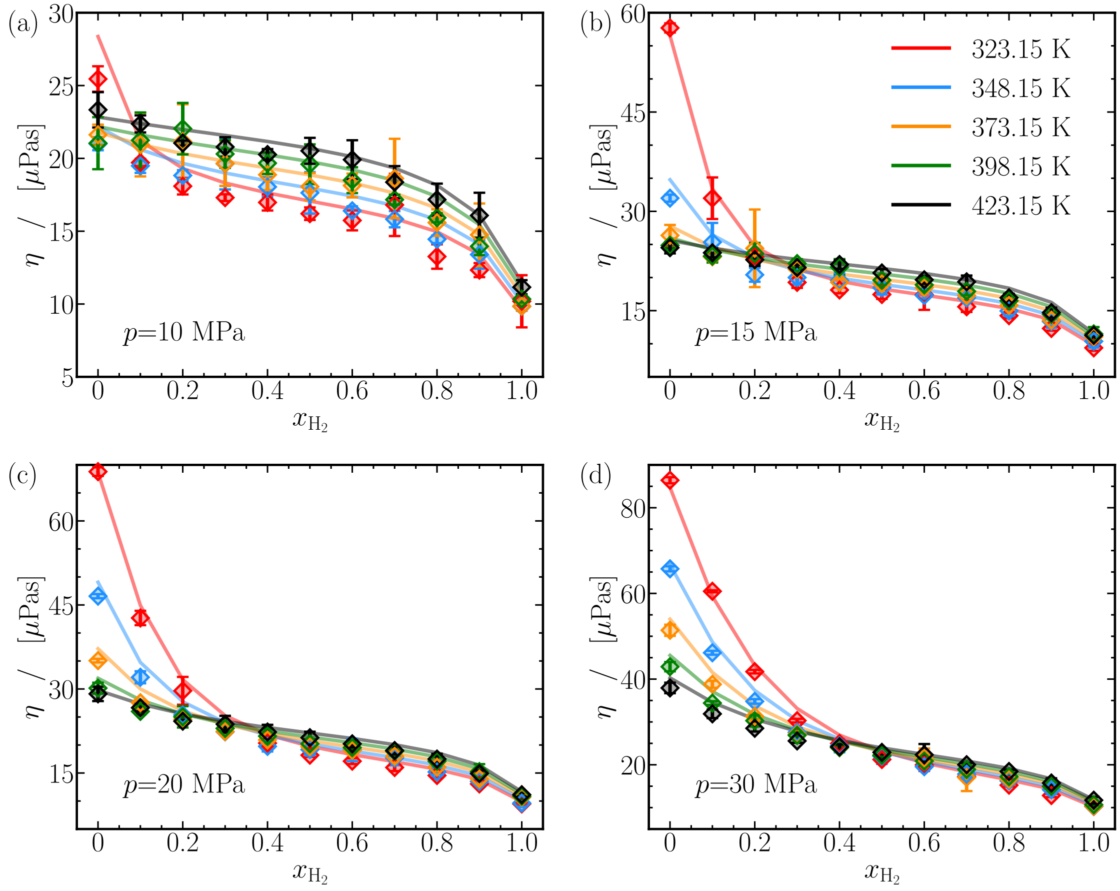

Figure S12: H_2-CO_2 mixture viscosities (η) at 10, 15, 20 and 30 MPa as a function of the hydrogen mole fraction x_{H_2}, and temperatures between 323.15 K and 423.15 K. Comparison between MD simulations (symbols) and REFPROP[3] (solid lines). Error bars are smaller than the symbols. Lines are colored as per the legend in subfigure (b).

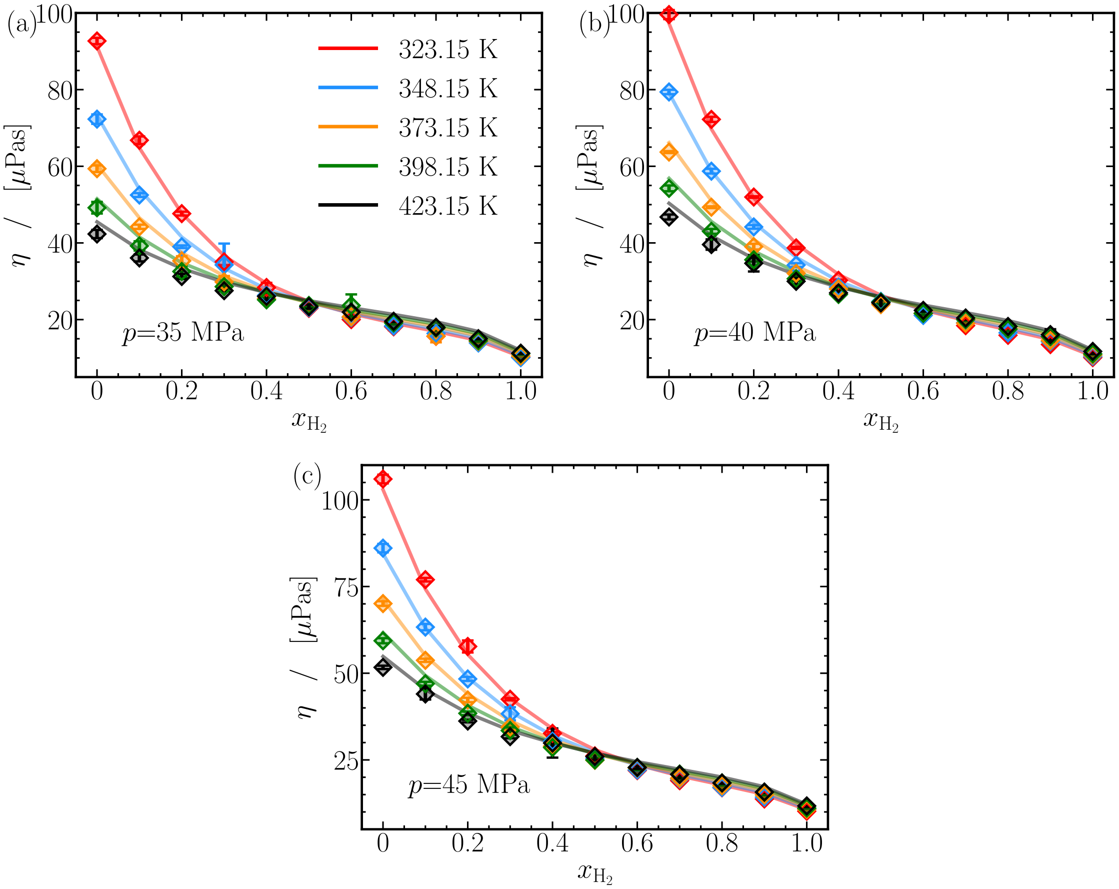

Figure S13: H_2-CO_2 mixture viscosities (η) at 35, 40, and 45 MPa as a function of the hydrogen mole fraction x_{H_2}, and temperatures between 323.15 K and 423.15 K. Comparison between MD simulations (symbols) and REFPROP[3] (solid lines). Error bars are smaller than the symbols. Lines are colored as per the legend in subfigure (a). See Fig. S12.

S3.3 Viscosity vs pressure

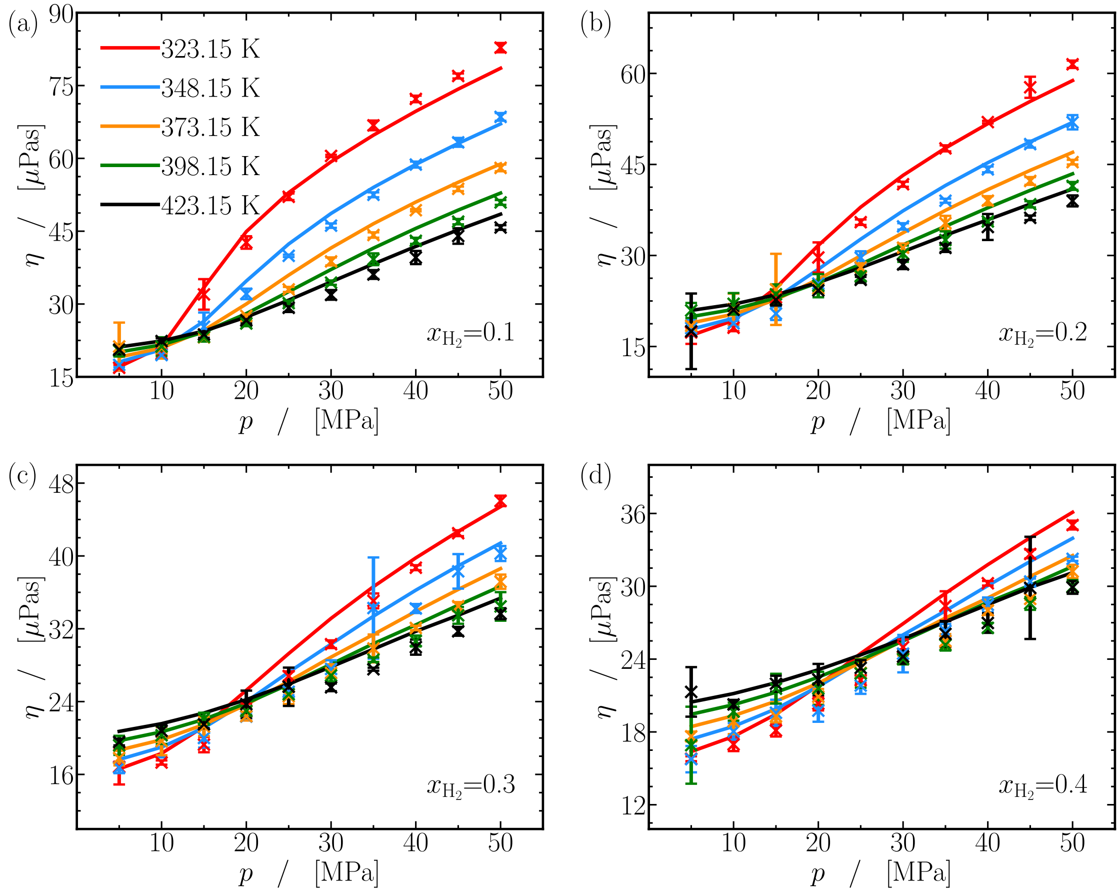

Figure S14: H_2-CO_2 mixture viscosities (η) for different hydrogen mole fractions between 0.1 and 0.4, as a function of the pressure p, and temperatures between 323.15 K and 423.15 K. Comparison between MD simulations (symbols) and REFPROP[3] (solid lines). Lines are colored as per the legend in subfigure (a).

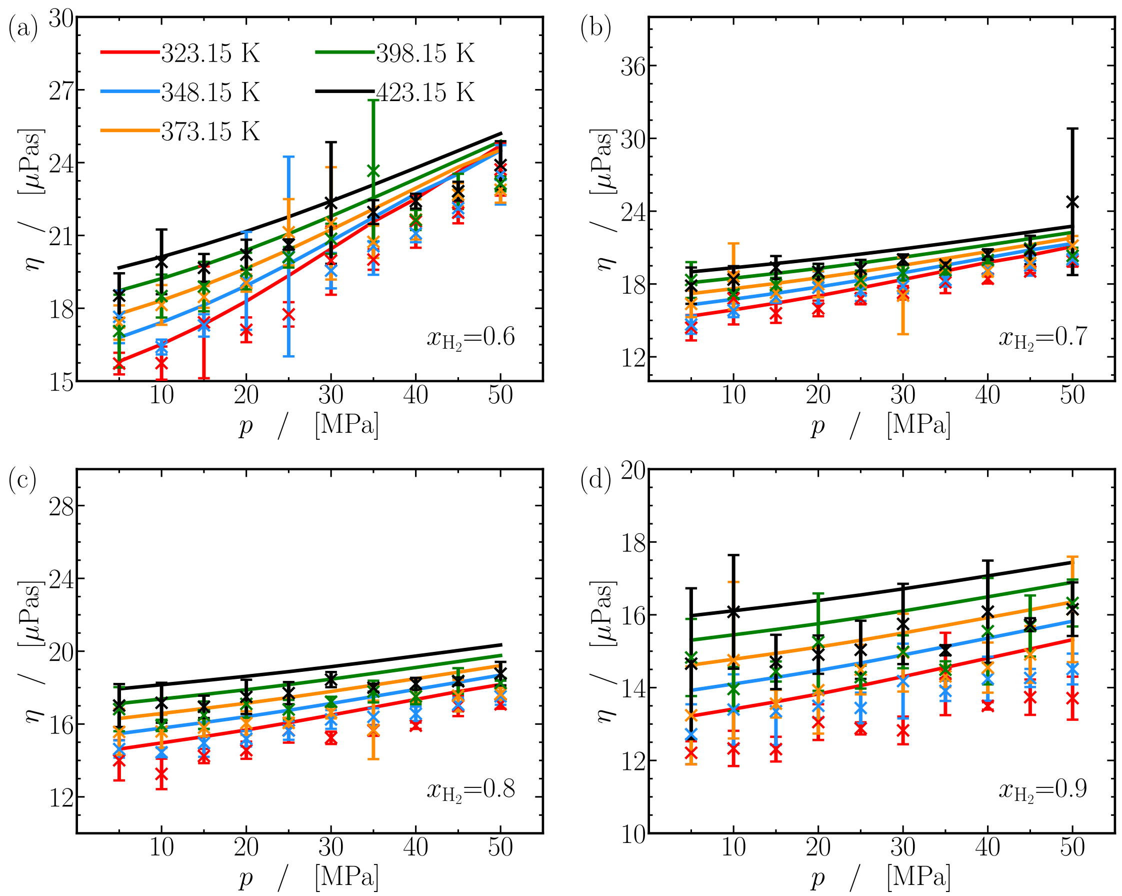

Figure S15: H_2-CO_2 mixture viscosities (η) for different hydrogen mole fractions between 0.6 and 1, as a function of the pressure p, and temperatures between 323.15 K and 423.15 K. Comparison between MD simulations (symbols) and REFPROP[3] (solid lines). Lines are colored as per the legend in subfigure (a). See Fig. S14.

S4 Self-diffusion coefficients

The self diffusivity of the ith species as calculated in an MD simulation is given by[4–6],

$$D_i^{\mathrm{self,MD}} = \lim_{t\to\infty} \frac{\mathrm{d}}{\mathrm{dt}} \left[\frac{1}{6N_i} \left\langle \sum_{j=1}^{N_i} \left(\mathbf{r}_{j,i}(t) - \mathbf{r}_{j,i}(0)\right)^2 \right\rangle \right] \tag{S4}$$

where N_i is the number of molecules of type i, $\mathbf{r}_{j,i}$ is the position vector of the jth molecule at time t, and the diffusion coefficient in the three orthogonal directions x, y and z each contributes a factor 2 in the denominator.

S4.1 Determination of self-diffusion coefficients from MD simulations

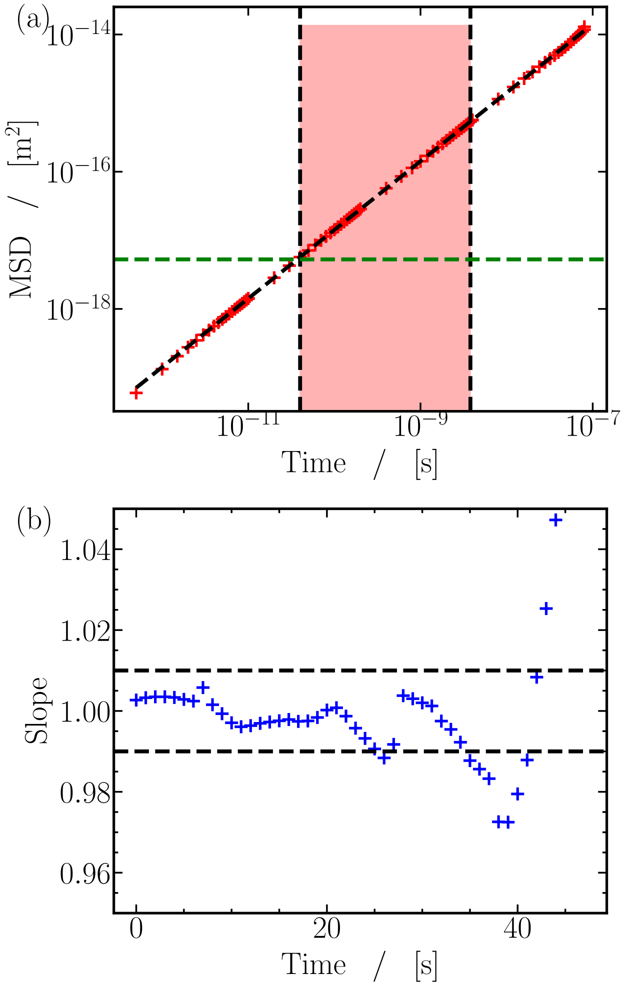

Figure S16: (a) Mean-squared displacements (symbols) of H_2 (MSD), computed as per Eq. S4, as a function of time for H_2 in an equimolar mixture of CO_2 and H_2 at 50 MPa and 323.15 K. The green dashed line represents the square of the box length 22.97 Å, indicating a complete box traversal. The black dashed line is a linear fit based on data points within the red region. (b) Slopes of the MSDs, calculated using 10 successive points, of the points falling within the shaded region have a slope between 0.99 and 1, and are used for the calculation of self-diffusivities.

S4.2 Finite system-size effects for D^{self}

Based on the density of the system, the self-diffusion coefficients are corrected for the finite-size effects using the Yeh-Hummer correction term D^{YH}[7,8],

$$D_i^{\text{self}} = D_i^{\text{self,MD}} + D^{\text{YH}}(T, \eta, L) = D_i^{\text{self,MD}} + \frac{k_B T \xi}{6\pi\eta L} \tag{S5}$$

where D_i^{self} is the finite-size corrected self diffusivity of the ith species, ξ is a dimensionless constant equal to 2.837298, and L is the box length of a cubic simulation box whose volume is V. Similar to the calculation of η, the segment of the molecular mean-square displacements with a slope between 0.99 and 1.01 on a log-log plot is chosen for the calculation of D_i^{self}, see Fig. S16. As pointed out by Moultos *et al.*[9] the Yeh-Hummer correction to $D_i^{\text{self,MD}}$ is significant for dense systems and can be neglected otherwise, as will be shown in a subsequent section. Self-diffusion coefficients are corrected for finite sizes as per Eq. S5, if the correction is larger than 1% percent of $D_i^{\text{self,MD}}$.

In Figs. S17 and S18, D^{self} of H_2 and CO_2 are plotted as a function of $N_{\text{tot}}^{-\frac{1}{3}}$, since the size of the simulation box size scales as $L \propto N_{\text{tot}}^{\frac{1}{3}}$. For the dilute system (33 kg/m^3 in Fig. S17), D^{self} of CO_2 and H_2 are nearly independent of the system size, while for the denser system (389 kg/m^3 in Fig. S18), D^{self} of CO_2 and H_2 are linearly related to $N_{\text{tot}}^{-\frac{1}{3}}$ with a non-zero slope, suggesting a strong system-size dependence of D^{self}.

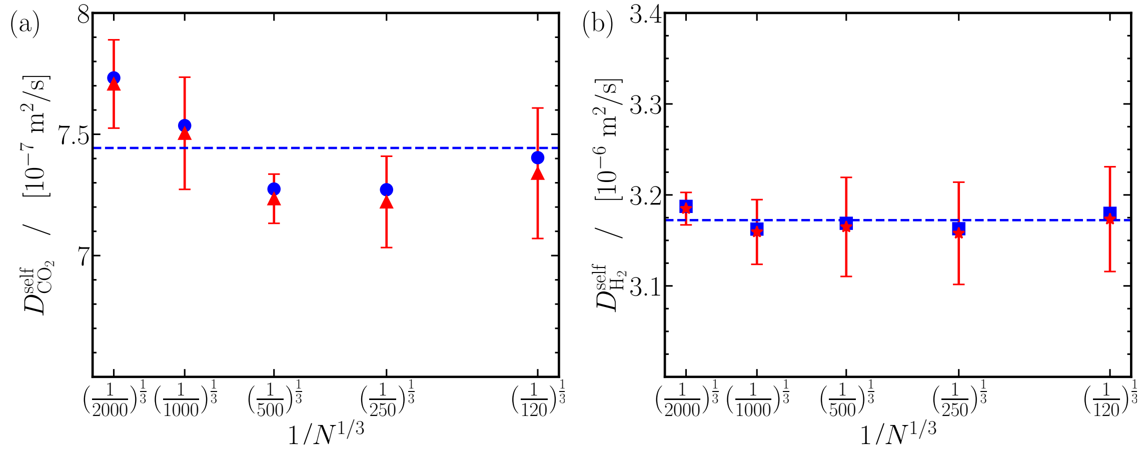

Figure S17: Finite-size corrections for self-diffusion coefficients of (a) CO_2 and (b) H_2 in an equimolar mixture, obtained from MD simulations for various system sizes (N=120, 250, 500, 1000, and 2000 molecules) at 5 MPa and 423.15 K. The mixture density is ca. 33 kg/m^3. The red symbols represent self-diffusion coefficients from MD simulations, while the blue symbols are the Yeh-Hummer[7] corrected self-diffusion coefficients (refer to Eq. 4 in the main text). The blue dashed line displays the mean value of the blue symbols. Self-diffusion coefficients are nearly independent of the system size.

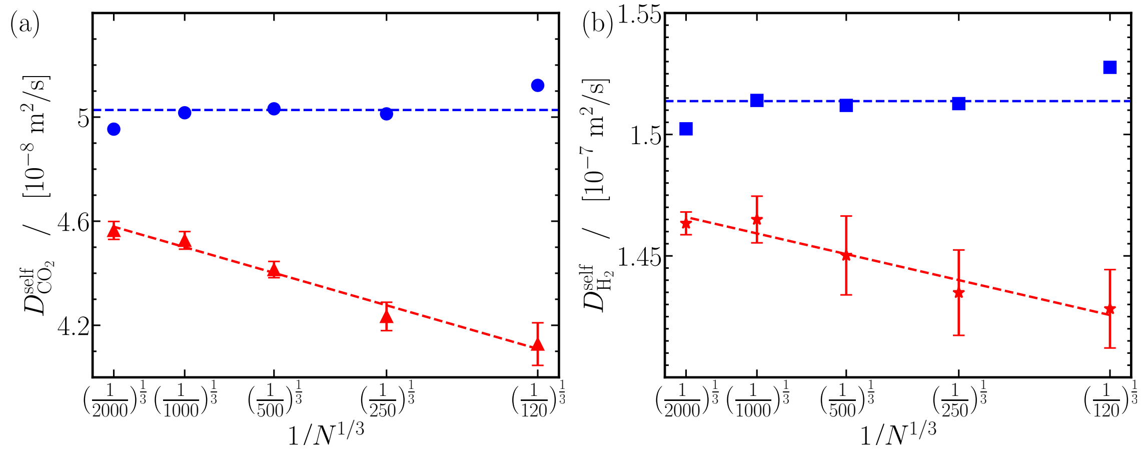

Figure S18: Finite-size corrections for self-diffusion coefficients of (a) CO_2 and (b) H_2 in an equimolar mixture, obtained from MD simulations for various system sizes (N=120, 250, 500, 1000, and 2000 molecules) at 50 MPa and 323.15 K. The mixture density is ca. 389 kg/m^3. The red symbols represent self-diffusion coefficients from MD simulations, while the blue symbols are the Yeh-Hummer[7] corrected self-diffusion coefficients (refer to Eq. 4 in the main text). The red dashed line is a linear fit to the red symbols, whereas the blue dashed line displays the mean value of the blue symbols. Self-diffusion coefficients are strongly dependent of the system size. See Fig. S17.

S4.3 Self-diffusion coefficients of CO_2 and H_2

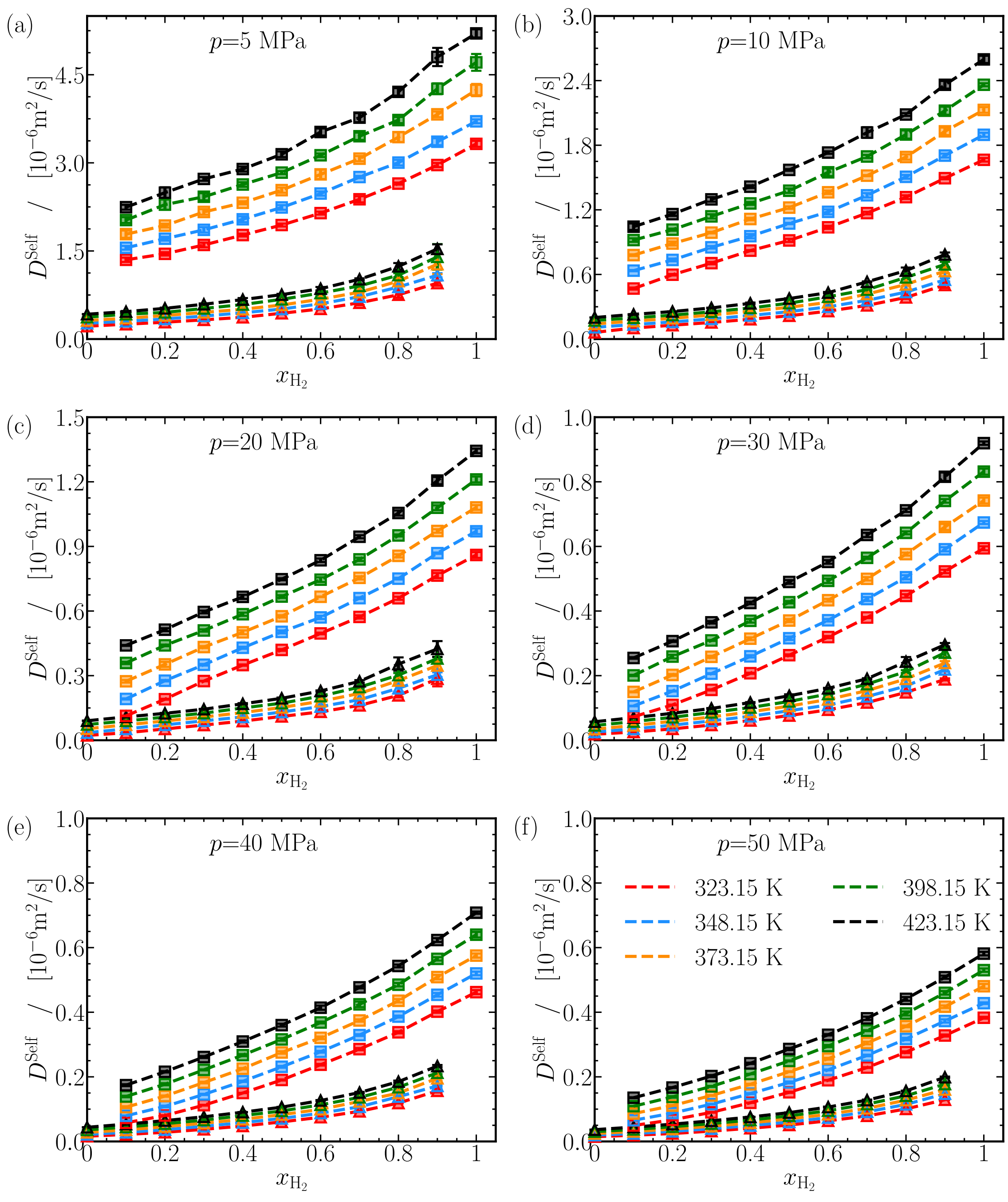

Figure S19: Self diffusivities of H_2 and CO_2, represented on a linear vertical axis, as a function of the mole fraction of hydrogen, for various pressures. The symbols are colored according to the legend in subfigure (f).

S4.4 Ratio of self-diffusion coefficients of CO_2 and H_2

To test the validity of the Stokes-Einstein[10] relation for mixtures of CO_2 and H_2, assuming perfect-stick boundary conditions[10,11], we calculate the ratio,

$$\frac{D^{\mathrm{self}}_{H_2}}{D^{\mathrm{self}}_{CO_2}} = \frac{k_B T}{6\pi\eta R^{\mathrm{eff}}_{\mathrm{H_2}}} \cdot \frac{6\pi\eta R^{\mathrm{eff}}_{\mathrm{CO_2}}}{k_B T} = \frac{R^{\mathrm{eff}}_{\mathrm{CO_2}}}{R^{\mathrm{eff}}_{\mathrm{H_2}}} \approx 3.14, \tag{S6}$$

where $R^{\mathrm{eff}}_{\mathrm{CO_2}}$ represents the separation between the carbon and oxygen atoms within the CO_2 molecule (1.16 Å), while $R^{\mathrm{eff}}_{\mathrm{H_2}}$ denotes half the distance between the two hydrogen atoms in H_2 (0.37 Å).

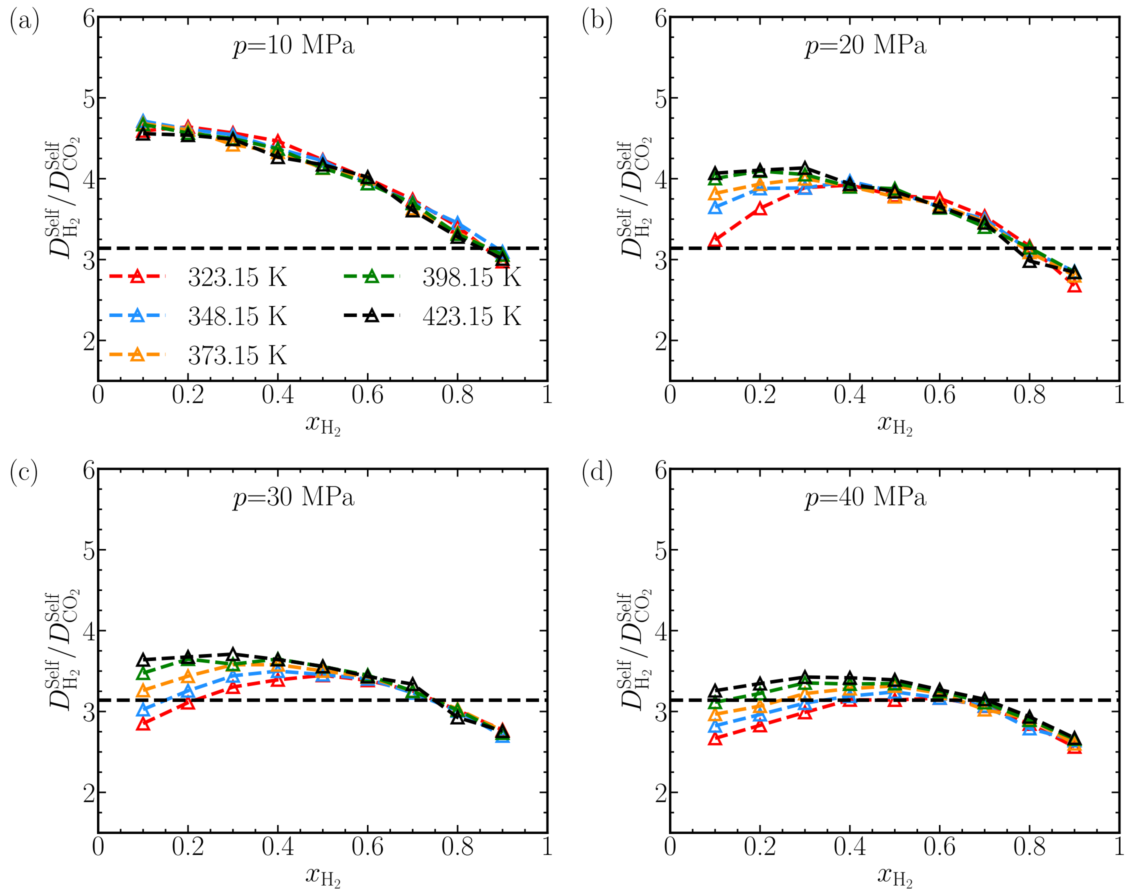

Figure S20: Ratio of self diffusion coefficients of H_2 and CO_2 as a function of the mole fraction of hydrogen, for various pressures. The dashed line represents the ratio of the Stokes-Einstein relation used to evaluate D^{self} of CO_2 and H_2, see Eq. S6. The symbols are colored according to the legend in subfigure (a).

S4.5 Ratio of self-diffusion coefficients in CO_2-H_2 mixtures and pure components of CO_2 and H_2

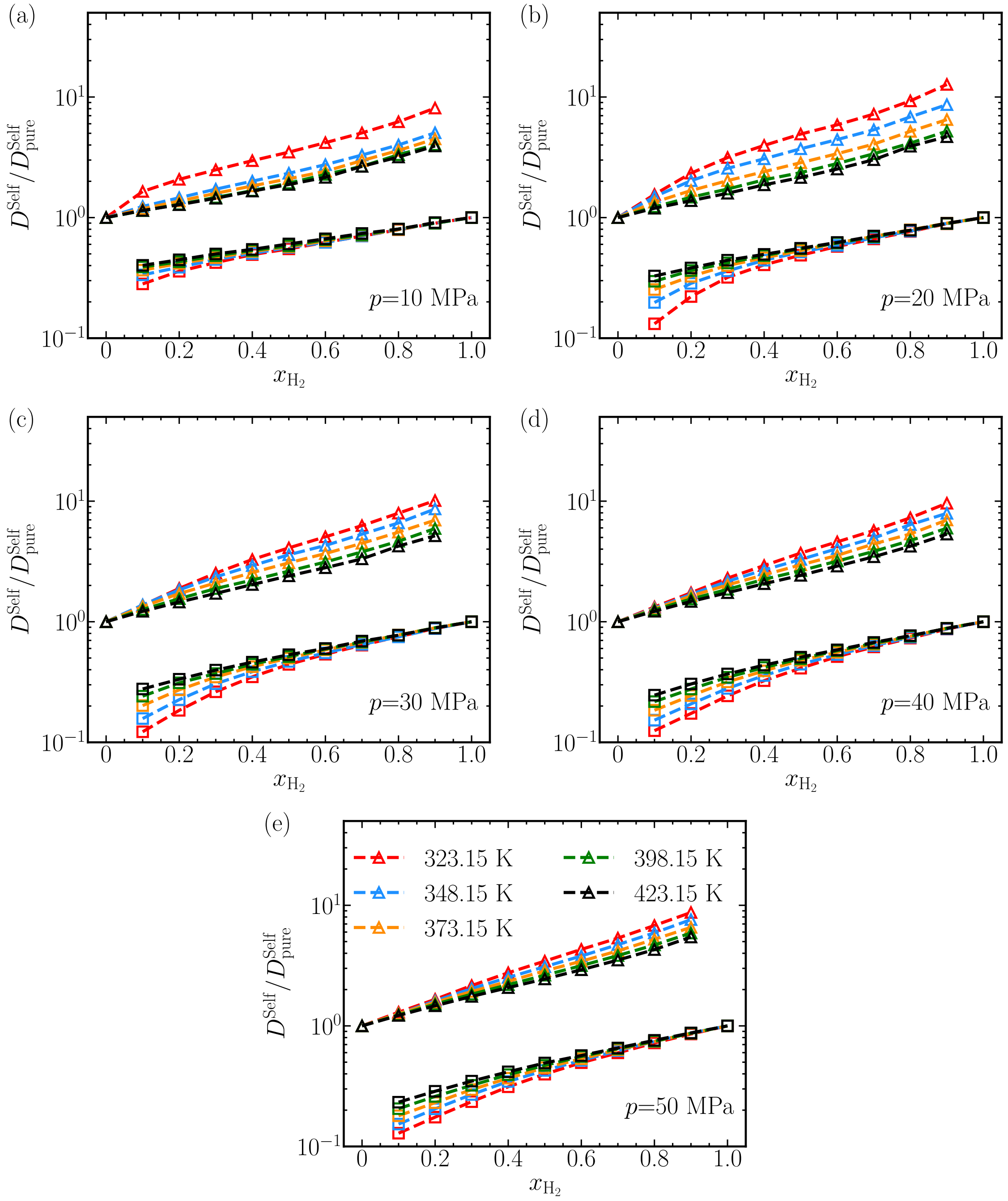

Figure S21: Comparing self-diffusion coefficients of CO_2 (triangles) and H_2 (squares) in CO_2-H_2 mixtures versus their values in pure fluids at the same temperature and pressure, considering different pressures, temperatures, and hydrogen mole fractions. The symbols are colored according to the legend in subfigure (e). See Fig. S20.

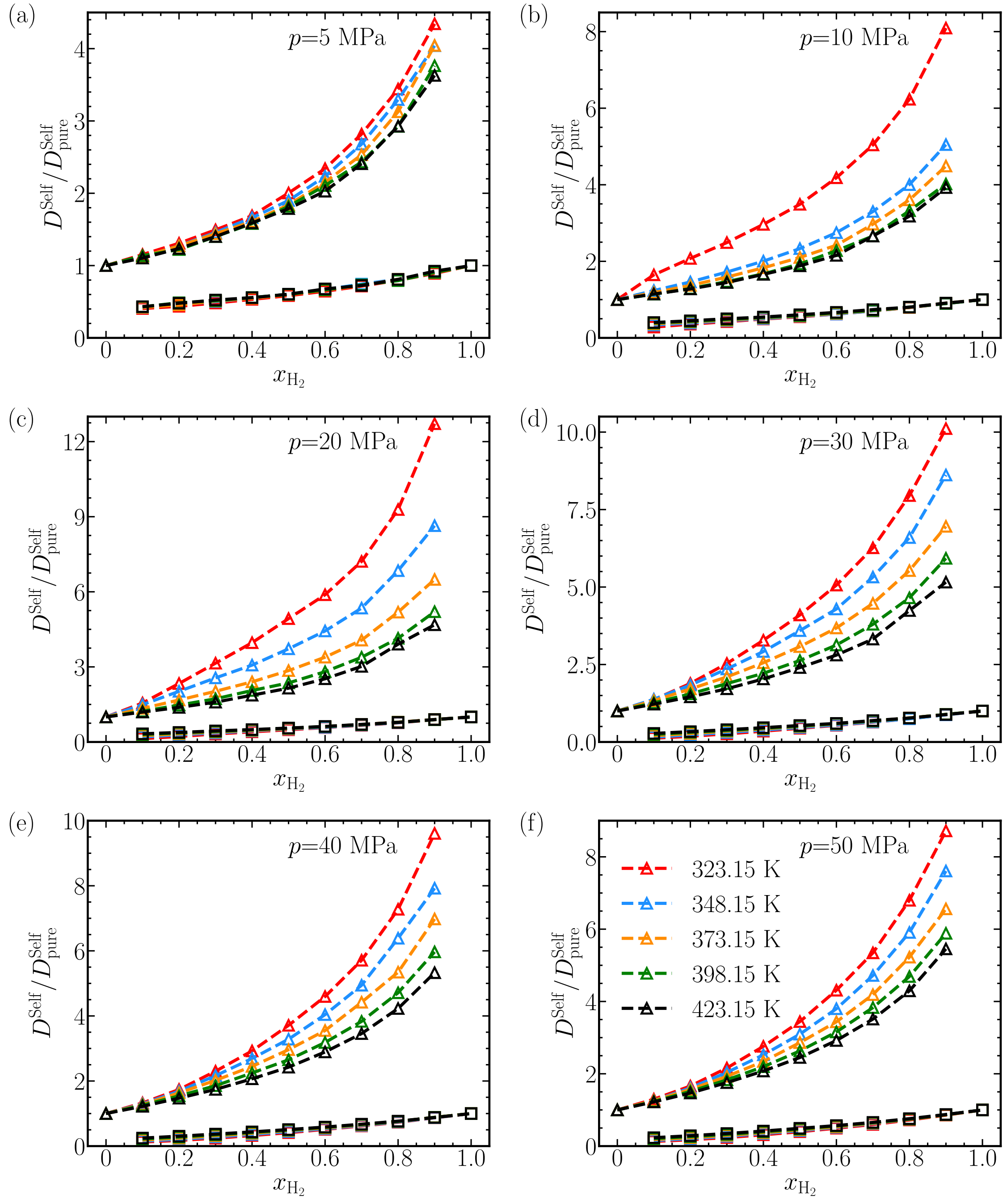

Figure S22: Comparison of self-diffusivities of CO_2 (triangles) and H_2 (squares) in CO_2-H_2 mixtures with their values in pure fluids at the same temperature and pressure, for different pressures, temperatures, and mole fractions of hydrogen. Subfigures (b)-(f) are identical to Figs. S21(a)-(e), except that the data are plotted on a vertical axis that is scaled linearly. The symbols are colored according to the legend in subfigure (f). See also Fig. S20.

S4.6 Effect of temperature on the self-diffusion coefficients of CO_2 and H_2 in CO_2-H_2 mixtures

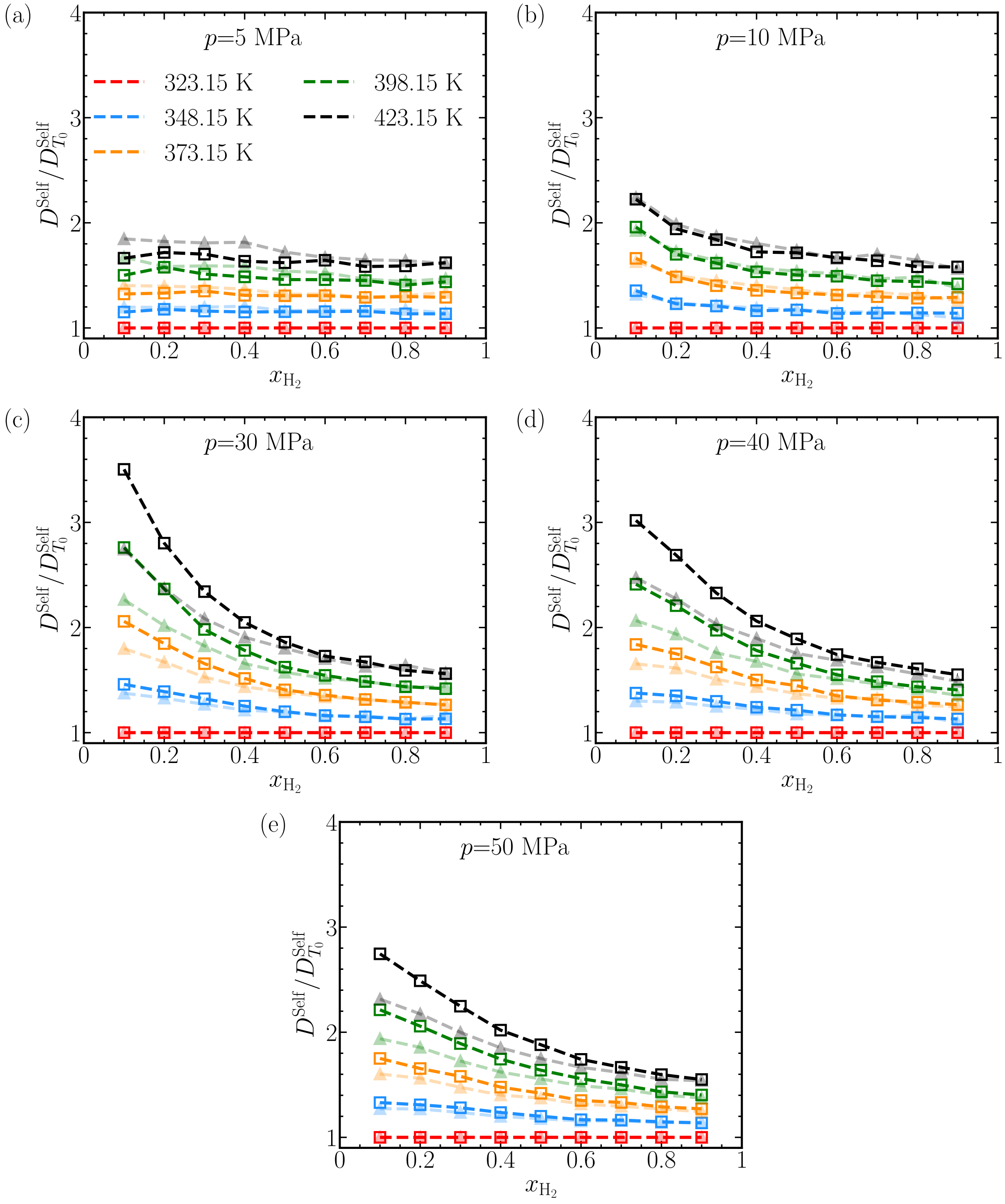

Figure S23: Comparing self-diffusion coefficients of CO_2 (triangles) and H_2 (squares) in CO_2-H_2 mixtures versus their values at $T_0 = 323.15$ K, considering different pressures, temperatures, and hydrogen mole fractions. The symbols are colored according to the legend in subfigure (a). See also Figs. S21 and S22.

S4.7 Self-diffusion coefficients of H_2 and CO_2 as a function of mixture density

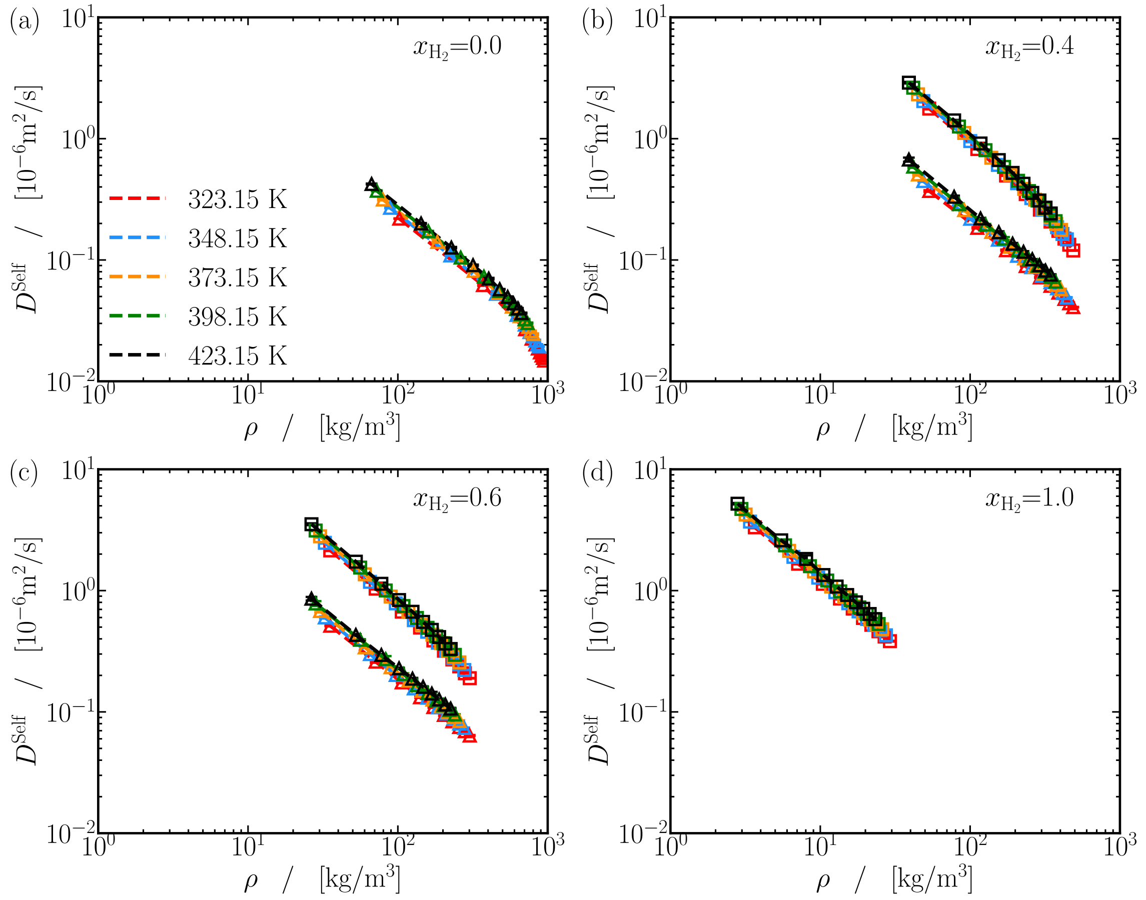

Figure S24: Finite system-size corrected self-diffusion coefficients of CO_2 (triangles) and H_2 (squares) as a function of the mixture densities at various mole fractions of H_2. The uncertainties in the computed self-diffusion coefficients are smaller than the symbol sizes. The symbols are colored according to the legend in subfigure (a).

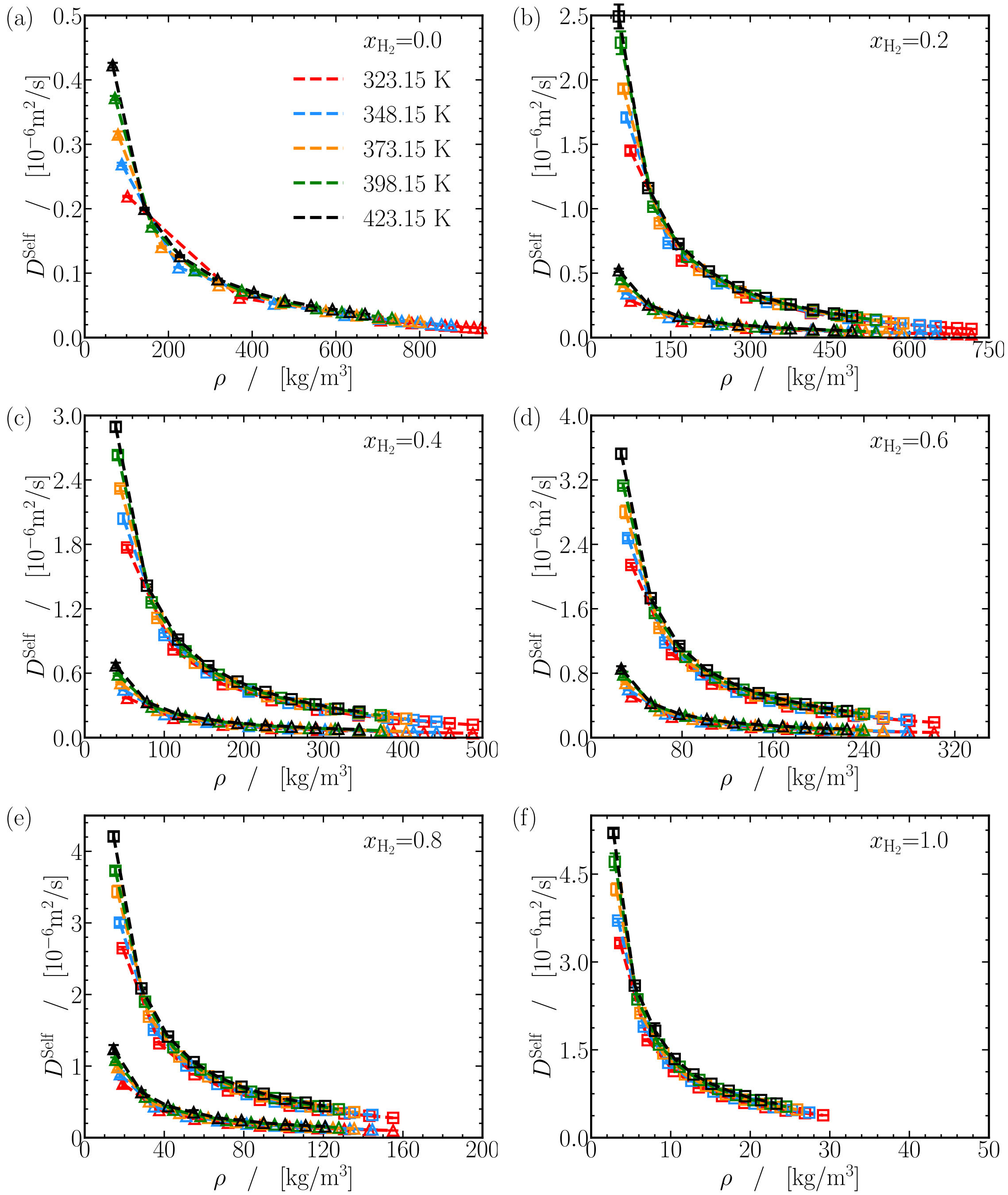

Figure S25: Finite system-size corrected self-diffusion coefficients of CO_2 (triangles) and H_2 (squares) as a function of the mixture densities, represented on linear horizontal and vertical axes. The uncertainties in the computed self-diffusion coefficients are smaller than the symbol sizes. The symbols are colored according to the legend in subfigure (a).

S5 Thermodynamic factors of diffusion for H_2-CO_2 mixtures

To compute the thermodynamic factors of binary mixtures of CO_2 and H_2, we obtain the Gibbs excess energy $G(p, T, x_{\mathrm{H_2}})$ from REFPROP[3], in units of J/mol. Values of G are normalized by RT, where R is the universal gas constant, and we define $Q = G(p, T, x_{\mathrm{H_2}})/RT$. A least-squares regression fitting procedure is used to fit Q to a suitable model, such as the one proposed by Margules[12,13]:

$$Q^{\mathrm{Mar}} = x_{\mathrm{H_2}}\,(1 - x_{\mathrm{H_2}})\,(A_{12}\,(1 - x_{\mathrm{H_2}}) + A_{21}x_{\mathrm{H_2}}) \tag{S7}$$

where A_{12} and A_{21} are the fitting parameters. The value of Γ can be obtained by differentiating Q[12],

$$\Gamma = 1 + 2x_{\mathrm{H_2}}\,(1 - x_{\mathrm{H_2}})\,(\,(A_{21} - A_{12})(1 - 3x_{\mathrm{H_2}}) - A_{12}). \tag{S8}$$

The van Laar model[12] is an alternative activity coefficient model[12]. We found that the thermodynamic factors predicted by the Margules and van Laar models exhibit a variation of less than 1% for all values of p, T, and $x_{\mathrm{H_2}}$, suggesting that the values of Γ are minimally affected by the underlying activity coefficient model.

S6 Maxwell-Stefan and Fick Diffusion coefficients of H_2-CO_2 mixtures

The MS diffusion coefficients are determined by first calculating the Onsager coefficients Λ_{ik} at zero total linear momentum[14–16]. Λ_{ik} is computed from the cross-correlations between the molecular displacements of species i and k[4,14–16],

$$\Lambda_{ik} = \lim_{t\to\infty} \frac{\mathrm{d}}{\mathrm{dt}} \left[\frac{1}{6N_{\mathrm{tot}}} \left\langle \left(\sum_{l=1}^{N_i} \left(\mathbf{r}_{l,i}(t) - \mathbf{r}_{l,i}(0) \right) \right) \times \left(\sum_{m=1}^{N_k} \left(\mathbf{r}_{m,k}(t) - \mathbf{r}_{m,k}(0) \right) \right) \right\rangle \right] \tag{S9}$$

where N_{tot} is the total number of molecules in a binary system consisting of N_i and N_k molecules of type i and k, respectively. Similar to the calculation of η and D^{self}, the segment of the cross-correlations with a slope between 0.99 and 1.01 on a log-log plot is chosen for the calculation of the Onsager coefficients, also see Fig. S26 of the Supporting Information. The MS diffusion coefficient $D^{\mathrm{MS,MD}}$ for a binary mixture is then expressed as a linear combination of the Onsager coefficients[4,14–16],

$$D^{\mathrm{MS,MD}} = \frac{x_2}{x_1}\Lambda_{11} + \frac{x_1}{x_2}\Lambda_{22} - 2\Lambda_{12} \tag{S10}$$

where x_1 and x_2 are the mole fractions of the components in the binary mixture.

S6.1 Determination of Maxwell-Stefan diffusion coefficient from MD simulations

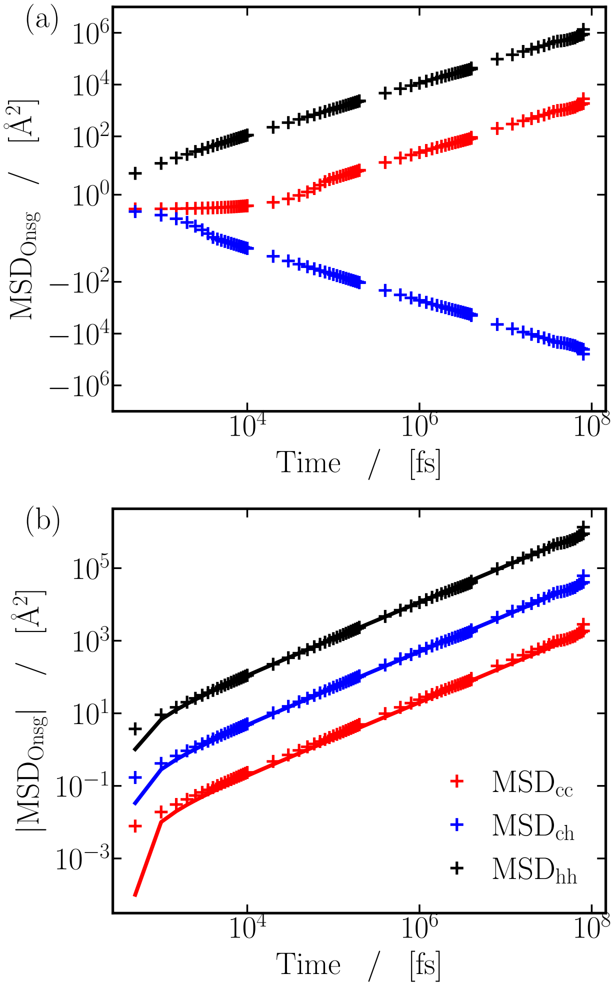

Figure S26: (a) Cross correlations of molecular displacements (Eq. S9 in the main text) (MSD$_{Onsg}$) are examined as a function of time in an equimolar mixture of H_2 and CO_2 at 323.15 K and 50 MPa. These correlations can have positive values for like species interactions (e.g., Carbon-Carbon in CO_2 shown in red symbols) and negative values for unlike species interactions (e.g., Carbon-Hydrogen shown in blue symbols). (b) Onsager coefficients (as per Eq. 9 in the main text) are derived by first plotting the absolute values of the cross correlations on a log-log scale and identifying the region with a slope within 1% of unity, see also Figs. S11 and S16. The sign of the slopes are adjusted based on the displacements shown in (a), are used for evaluation of the Maxwell-Stefan diffusion coefficient using Eq. 9 of the main text.

S6.2 Finite system-size effects at ρ=389 kg/m^3

Jamali *et al.*[17] showed that the MS diffusion coefficients can be corrected according to,

$$D^{\mathrm{MS}} = D^{\mathrm{MS,MD}} + \frac{1}{\Gamma} D^{\mathrm{YH}} \tag{S11}$$

where D^{MS} is the finite size corrected MS diffusivity, D^{YH} is the correction proposed by Yeh and Hummer[7] for correcting self-diffusion coefficients computed from finite-size systems (see Eq. S5) and Γ is the thermodynamic factor. In the same article, Jamali *et al.*[17] suggested that one only needs the Yeh-Hummer correction term[7] D^{YH} to obtain the Fick diffusion coefficients in the thermodynamic limit[12,17],

$$D^{\mathrm{Fick}} = \Gamma D^{\mathrm{MS}} = D^{\mathrm{Fick,MD}} + D^{\mathrm{YH}}. \tag{S12}$$

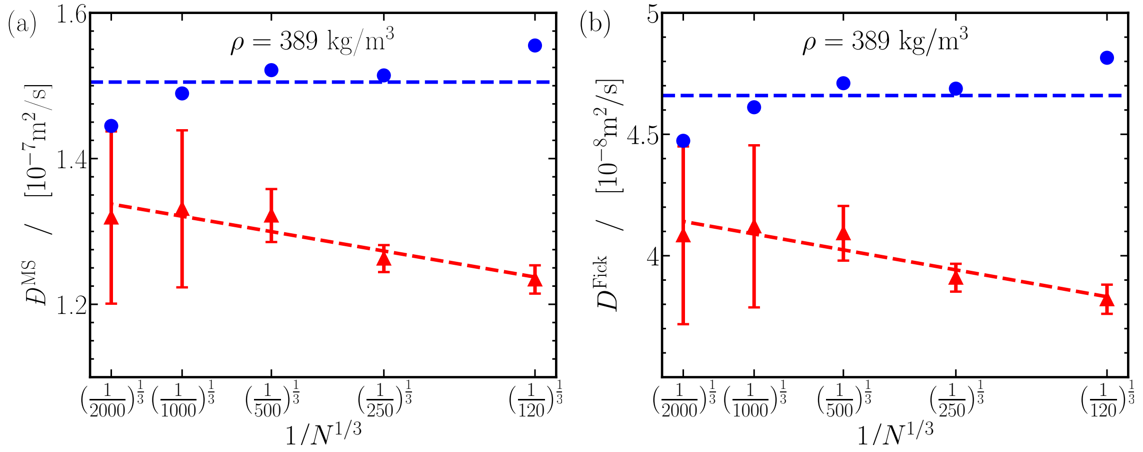

Figure S27: Illustration of finite-size effects in the calculation of Maxwell-Stefan and Fick diffusion coefficients for an equimolar mixture at 50 MPa and 323.15 K ($\rho \approx 389$ kg/m^3). The thermodynamic factor Γ used for the correction of the Maxwell-Stefan (see Eq. S11) and Fick diffusion coefficients (see Eq. S12) equals 0.31.

S6.3 Fick diffusion coefficients of H_2-CO_2 mixtures

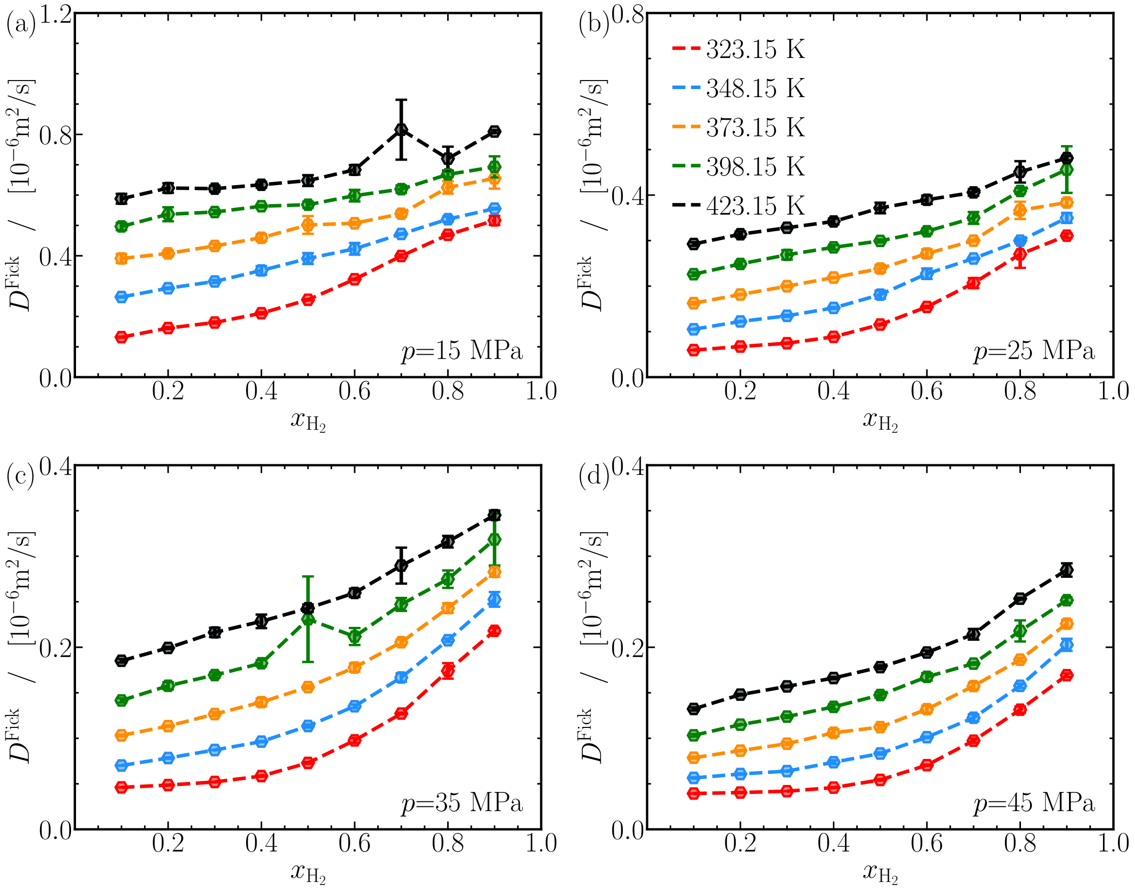

Figure S28: Finite-system-size-corrected Fick diffusion coefficients as a function of the mole fraction of hydrogen (x_{H_2}). Lines are colored as per the legend in subfigure (b). The dashed lines act as guides to the eye.

S6.4 Effect of H_2 mole fraction on the Fick diffusion coefficients of H_2-CO_2 mixtures

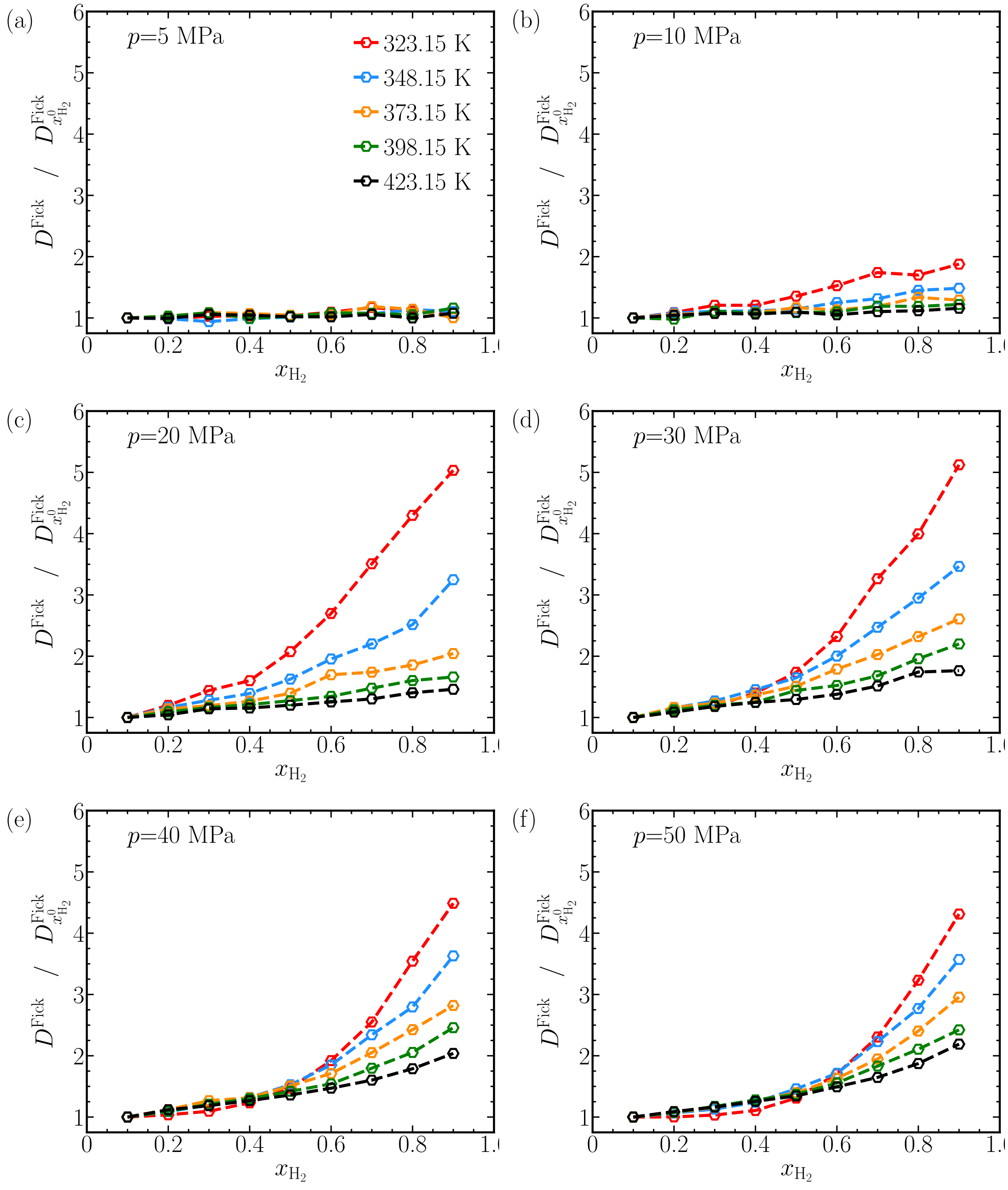

Figure S29: Ratio of the Fick diffusion coefficients, computed from MD simulations, at a given pressure and temperature with respect to the corresponding value at a reference hydrogen mole fraction $x^0_{H_2} = 0.1$.

S6.5 Effect of temperature on the Fick diffusion coefficients of H_2-CO_2 mixtures

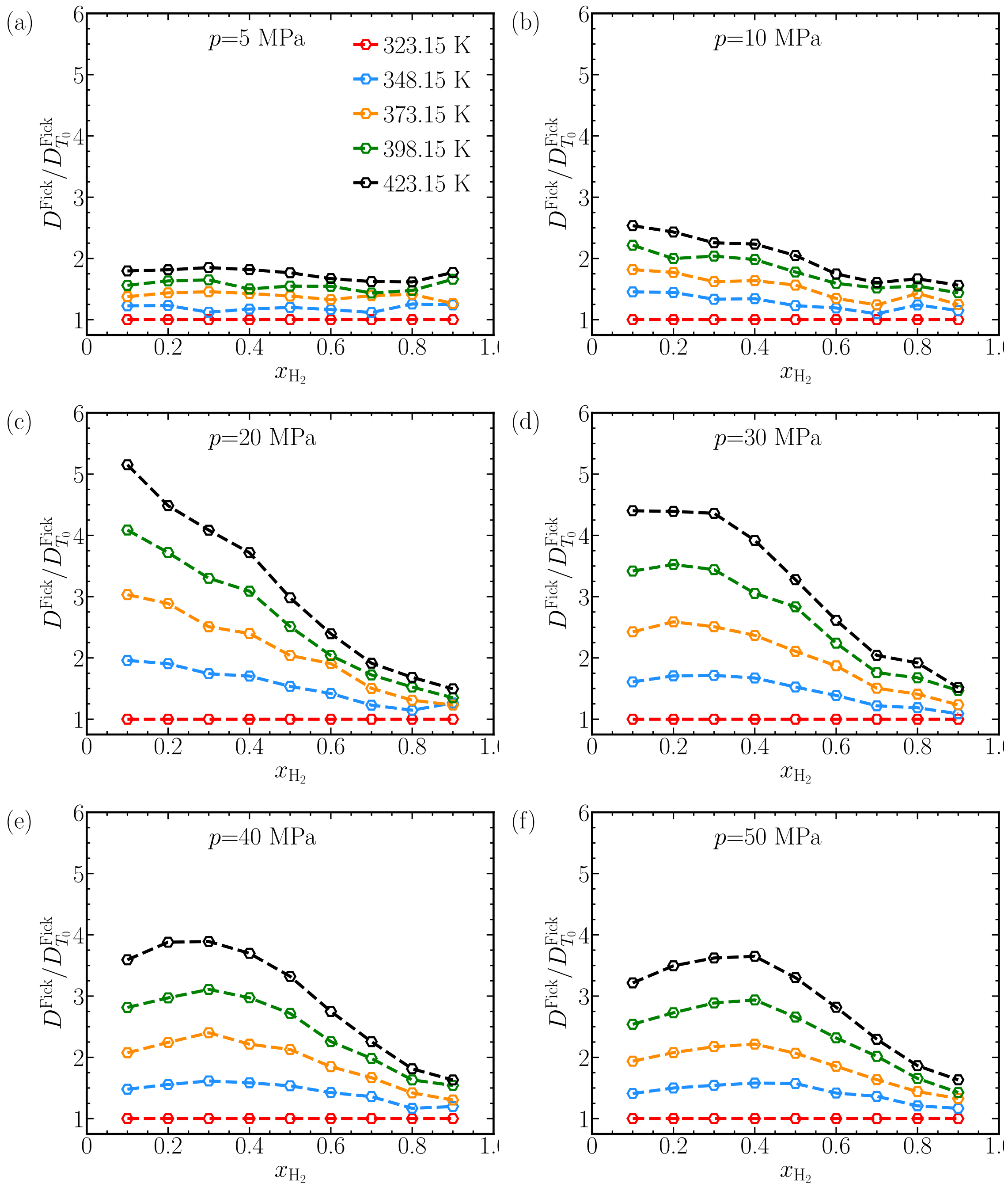

Figure S30: The ratio of Fick diffusion coefficients at a particular mole fraction of H_2 and temperature compared to their values at $T_0 = 323.15$ K.

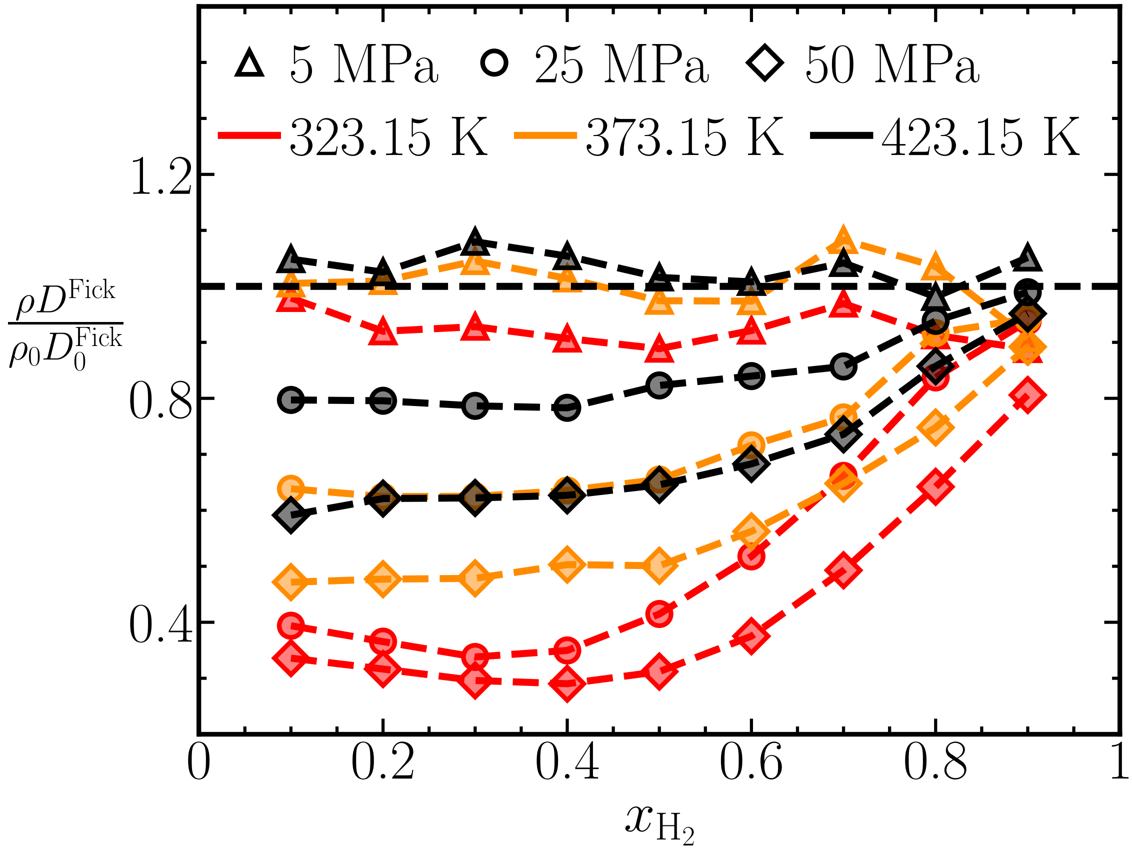

Figure S31: Principle of corresponding states applied to the product of the computed Fick diffusivity and the corresponding mixture density. Here, the reference density ρ_0 for a mixture is calculated at 0.1 MPa and 298.15 K using the ideal-gas equation of state. The black dashed line at unity is plotted to identify the corresponding states.

S7 Phase equilibria of CO_2-NaCl brine systems

S7.1 Effect of pressure on the solubility of CO_2

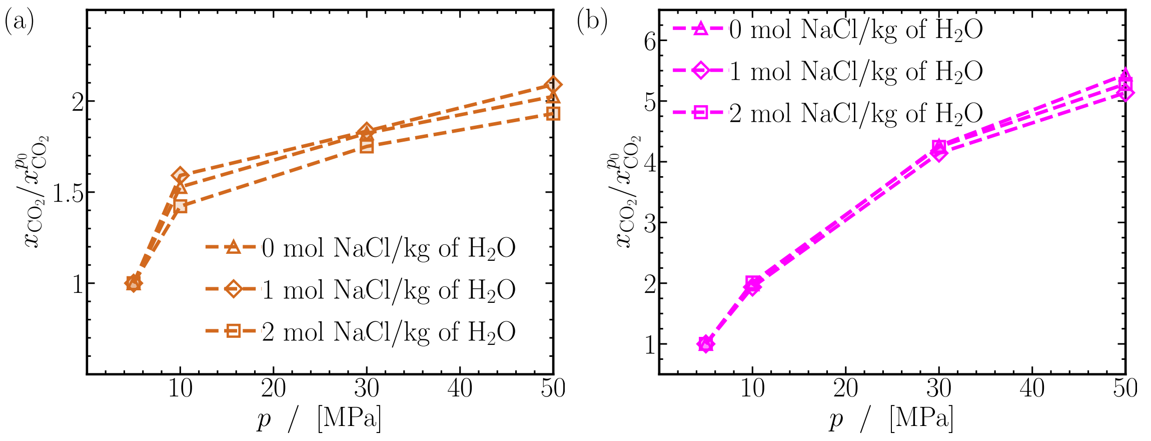

Figure S32: CO_2 solubilities, computed from MC simulations, in NaCl brine compared to the corresponding solubility at 5 MPa ($x_{CO_2}^{p^0}$), at (a) 323.15 K and (b) 423.15 K.

S7.2 Effect of temperature on the solubility of CO_2

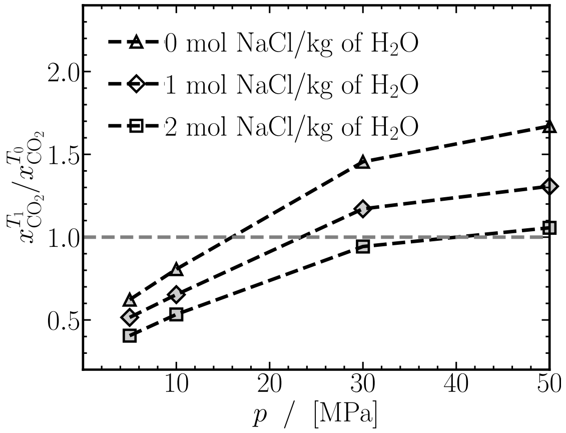

Figure S33: CO_2 solubilities, computed from MC simulations, in NaCl brine at $T_1 = 423.15$ K compared to the corresponding solubility at $T_0 = 323.15$ K. The dashed line is an indicator to identify the regime in which the solubility does not depend on the temperature.

S7.3 Effect of NaCl concentration on the solubility of CO_2

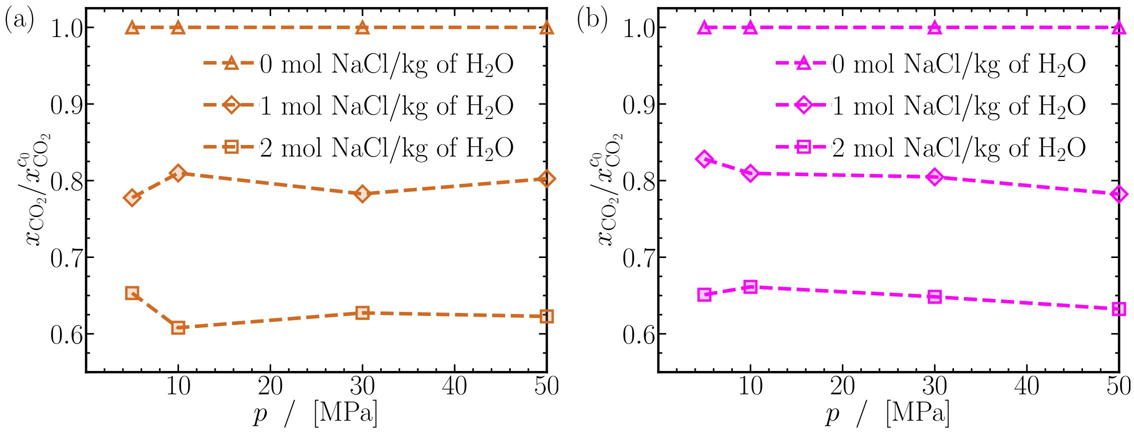

Figure S34: CO_2 solubilities, computed from MC simulations, in NaCl brine compared to the corresponding solubilities in pure H_2O ($x^{c^0}_{CO_2}$), at (a) 323.15 K and (b) 423.15 K. Subfigures (c) and (d) depict the natural logarithm of the ratio between the solubilities of CO_2 and its solubility in pure water at the same pressure and temperature computed from MC simulations, plotted as a function of the NaCl concentration in brine. Linear fits to the data points, indicated by dashed lines, reveal consistent slopes of ca. -0.23 and ca -0.21 for all lines in subfigures (c) and (d), respectively.

S8 Phase equilibria of H_2-NaCl brine systems

S8.1 Effect of pressure on the solubility of H_2

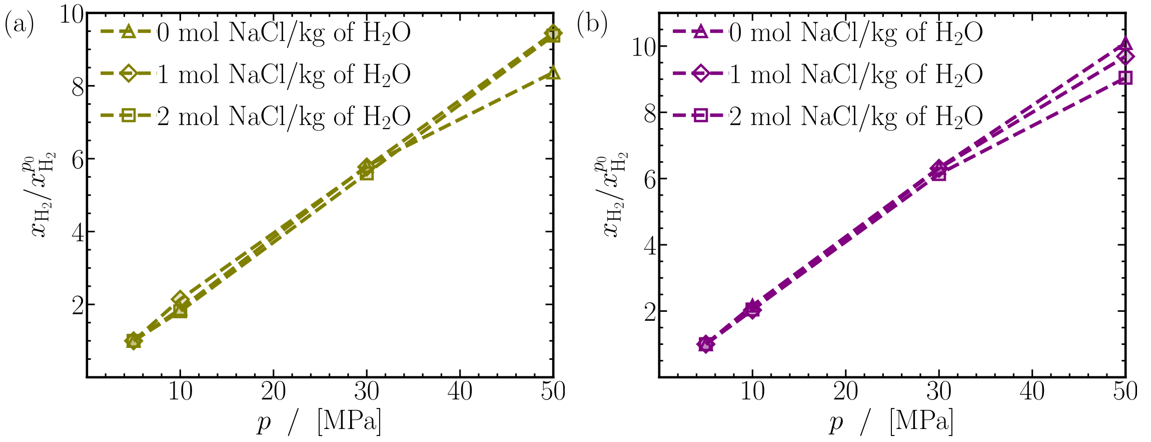

Figure S35: H_2 solubilities, computed from MC simulations, in NaCl brine compared to the corresponding solubility at 5 MPa ($x_{H_2}^{p^0}$), at (a) 323.15 K and (b) 423.15 K.

S8.2 Effect of temperature on the solubility of H_2

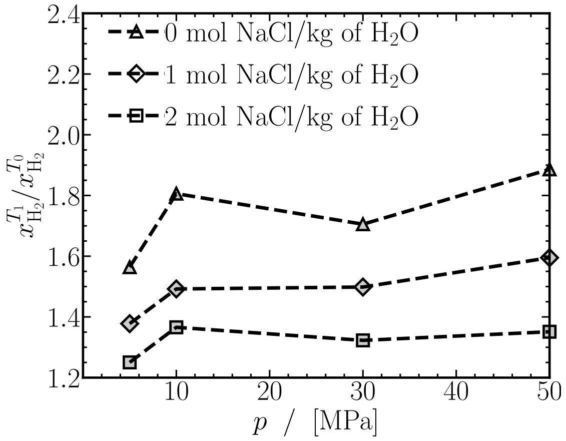

Figure S36: H_2 solubilities, computed from MC simulations, in NaCl brine at $T_1 = 423.15$ K compared to the corresponding solubilities at $T_0 = 323.15$ K.

S8.3 Effect of NaCl concentration on the solubility of H_2

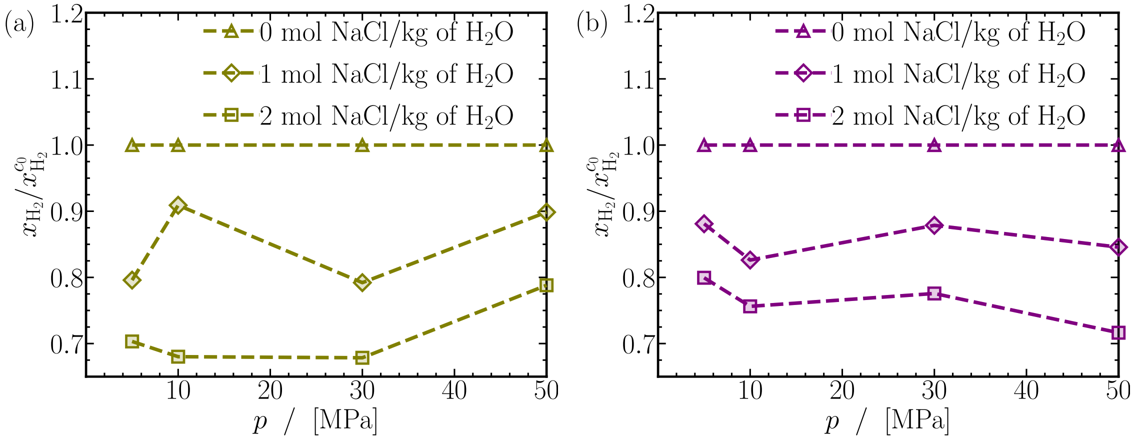

Figure S37: H_2 solubilities, computed from MC simulations, in NaCl brine compared to the corresponding solubilities in pure H_2O ($x^0_{H_2}$), at (a) 323.15 K and (b) 423.15 K. Subfigures (c) and (d) depict the natural logarithm of the ratio between the solubilities of CO_2 and its solubility in pure water at the same pressure and temperature computed from MC simulations, plotted as a function of the NaCl concentration in brine. Linear fits to the data points, indicated by dashed lines, reveal slopes between -0.11 and -0.16 in subfigure (c) and slopes between -0.11 and -0.19 subfigure (d).

S9 Force field parameters

Table S1: Interaction parameters of the TraPPE force field of carbon dioxide O=C=O[18], and the three-site Marx force field for hydrogen[19–21]. Each row contains the LJ and the electrostatic interaction parameters for the atom highlighted between brackets [ ]. Parameters between different species are calculated using the Lorentz-Berthelot mixing rules[5,6], except between Na^+ and Cl^- ions[22,23], Na^+ ions and the oxygen atom in water (Ow), and Cl^- ions and oxygen atoms in water (Ow)[22,23].

**Atom**	ε/k_B / $[K]$	σ / [Å]	q / [e]
O=[C]=O	27.0	2.80	0.70
[O]=C=O	79.0	3.05	-0.35
H-[L]-H	36.7	2.958	-0.936
[H]-L-H	-	-	0.468
M<br>H-[O]-H	79.86	3.1589	0
M<br>H-O-[H]	-	-	0.53136
[M]<br>H- O -H	-	-	-1.06272
[Na^+]Cl^-	177.0848	2.21737	0.85
Na^+[Cl^-]	9.251769	4.69906	-0.85
[Na^+][Cl^-]	173.06027	3.00512	-
[Na^+][Ow]	95.423247	2.60738	-
[Cl^-][Ow]	7.4548886	4.23867	-

S10 Raw simulation data

S10.1 Densities, compressibilities and total energies from MD simulations

Table S2: Densities (ρ) and compressibilities (Z) obtained from MD simulations of CO_2-H_2 mixtures comprising 2000 molecules are presented as functions of pressure, temperature, and mole fraction of H_2. The average box length L of the cubic simulation box is reported in units of angstrom (Å). Densities and compressibilities from the REFPROP database[3] are included for comparison. Total energies (E_{tot}) calculated for the systems in the NVE ensemble are also included.

p	T	x_{H_2}	L	ρ / [kg/m^3]		Z		E_{tot}
[MPa]	[K]		[Å]	ρ^{MD}	ρ^{RFP}	Z^{MD}	Z^{RFP}	[kJ/mol]
5	323.15	0.0	112.66 ± 0.13	102.22 ± 0.34	104.84	0.80	0.78	5.38
5	323.15	0.1	114.94 ± 0.06	87.06 ± 0.14	89.25	0.85	0.81	5.65
5	323.15	0.2	116.75 ± 0.02	74.31 ± 0.03	75.97	0.89	0.83	5.87
5	323.15	0.3	118.08 ± 0.04	63.37 ± 0.06	64.37	0.92	0.85	6.06
5	323.15	0.4	119.21 ± 0.06	53.35 ± 0.08	53.98	0.95	0.87	6.21
5	323.15	0.5	120.14 ± 0.04	44.08 ± 0.04	44.47	0.97	0.89	6.34
5	323.15	0.6	120.88 ± 0.02	35.38 ± 0.02	35.61	0.99	0.91	6.44
5	323.15	0.7	121.54 ± 0.05	27.03 ± 0.03	27.21	1.01	0.92	6.52
5	323.15	0.8	121.85 ± 0.04	19.12 ± 0.02	19.14	1.01	0.94	6.59
5	323.15	0.9	122.20 ± 0.07	11.31 ± 0.02	11.32	1.02	0.95	6.65
5	323.15	1.0	122.34 ± 0.01	3.66 ± 0.01	3.65	1.03	0.96	6.68
5	348.15	0.0	118.11 ± 0.02	88.71 ± 0.04	90.46	0.86	0.97	6.13
5	348.15	0.1	119.67 ± 0.04	77.14 ± 0.09	78.68	0.89	0.98	6.35
5	348.15	0.2	120.98 ± 0.06	66.80 ± 0.11	67.99	0.92	0.99	6.52

Continued on next page

p [MPa]	T [K]	x_{H_2}	L [Å]	ρ / [kg/m^3] ρ^{MD}	ρ^{RFP}	Z Z^{MD}	Z^{RFP}	E_{tot} [kJ/mol]
5	348.15	0.3	121.97 ± 0.02	57.49 ± 0.03	58.23	0.94	1.00	6.67
5	348.15	0.4	122.89 ± 0.01	48.70 ± 0.01	49.21	0.97	1.01	6.79
5	348.15	0.5	123.60 ± 0.05	40.47 ± 0.05	40.78	0.98	1.01	6.91
5	348.15	0.6	124.27 ± 0.03	32.56 ± 0.02	32.79	1.00	1.02	7.00
5	348.15	0.7	124.73 ± 0.02	25.01 ± 0.01	25.14	1.01	1.02	7.07
5	348.15	0.8	125.04 ± 0.03	17.69 ± 0.02	17.73	1.02	1.03	7.13
5	348.15	0.9	125.22 ± 0.04	10.51 ± 0.01	10.50	1.02	1.03	7.17
5	348.15	1.0	125.40 ± 0.03	3.40 ± 0.01	3.39	1.03	0.84	7.21
5	373.15	0.0	122.54 ± 0.03	79.44 ± 0.05	80.65	0.89	0.86	6.82
5	373.15	0.1	123.74 ± 0.06	69.78 ± 0.11	70.94	0.92	0.87	6.99
5	373.15	0.2	124.74 ± 0.04	60.94 ± 0.06	61.85	0.94	0.89	7.14
5	373.15	0.3	125.61 ± 0.09	52.64 ± 0.12	53.34	0.96	0.90	7.26
5	373.15	0.4	126.28 ± 0.01	44.88 ± 0.01	45.32	0.98	0.92	7.37
5	373.15	0.5	126.84 ± 0.03	37.46 ± 0.03	37.71	0.99	0.93	7.46
5	373.15	0.6	127.36 ± 0.07	30.25 ± 0.05	30.42	1.00	0.94	7.54
5	373.15	0.7	127.74 ± 0.01	23.29 ± 0.01	23.38	1.01	0.96	7.61
5	373.15	0.8	128.05 ± 0.02	16.47 ± 0.01	16.53	1.02	0.97	7.66
5	373.15	0.9	128.18 ± 0.03	9.80 ± 0.01	9.80	1.02	0.97	7.70
5	373.15	1.0	128.26 ± 0.04	3.17 ± 0.01	3.17	1.02	0.98	7.73
5	398.15	0.0	126.45 ± 0.01	72.29 ± 0.01	73.28	0.92	0.99	7.47
5	398.15	0.1	127.33 ± 0.03	64.04 ± 0.05	64.91	0.94	1.00	7.60
5	398.15	0.2	128.17 ± 0.05	56.17 ± 0.07	56.92	0.96	1.00	7.73
5	398.15	0.3	128.81 ± 0.03	48.81 ± 0.03	49.32	0.97	1.01	7.83

Continued on next page

p [MPa]	T [K]	x_{H_2}	L [Å]	ρ / [kg/m^3] ρ^{MD}	ρ^{RFP}	Z Z^{MD}	Z^{RFP}	E_{tot} [kJ/mol]
5	398.15	0.4	129.41 ± 0.02	41.70 ± 0.02	42.07	0.99	1.01	7.93
5	398.15	0.5	129.88 ± 0.08	34.88 ± 0.06	35.11	1.00	1.02	8.01
5	398.15	0.6	130.29 ± 0.02	28.25 ± 0.01	28.39	1.01	1.02	8.08
5	398.15	0.7	130.61 ± 0.02	21.78 ± 0.01	21.86	1.01	1.02	8.14
5	398.15	0.8	130.82 ± 0.06	15.45 ± 0.02	15.47	1.02	1.03	8.19
5	398.15	0.9	130.96 ± 0.01	9.19 ± 0.01	9.19	1.02	0.88	8.23
5	398.15	1.0	131.03 ± 0.02	2.98 ± 0.01	2.97	1.02	0.89	8.25
5	423.15	0.0	129.83 ± 0.02	66.79 ± 0.03	67.43	0.94	0.90	8.07
5	423.15	0.1	130.59 ± 0.01	59.37 ± 0.02	60.00	0.95	0.92	8.20
5	423.15	0.2	131.33 ± 0.05	52.21 ± 0.06	52.83	0.97	0.93	8.30
5	423.15	0.3	131.79 ± 0.03	45.57 ± 0.03	45.93	0.98	0.94	8.40
5	423.15	0.4	132.31 ± 0.02	39.02 ± 0.02	39.29	0.99	0.95	8.48
5	423.15	0.5	132.80 ± 0.08	32.63 ± 0.06	32.87	1.00	0.96	8.56
5	423.15	0.6	133.08 ± 0.03	26.51 ± 0.02	26.63	1.01	0.97	8.62
5	423.15	0.7	133.33 ± 0.03	20.48 ± 0.02	20.54	1.01	0.98	8.67
5	423.15	0.8	133.48 ± 0.03	14.54 ± 0.01	14.55	1.02	0.98	8.72
5	423.15	0.9	133.65 ± 0.02	8.65 ± 0.01	8.65	1.02	0.99	8.75
5	423.15	1.0	133.68 ± 0.01	2.80 ± 0.01	2.80	1.02	1.00	8.77
10	323.15	0.0	73.37 ± 0.32	369.98 ± 4.89	384.33	0.44	1.00	2.23
10	323.15	0.1	84.18 ± 0.05	221.63 ± 0.43	235.85	0.67	1.01	4.10
10	323.15	0.2	88.43 ± 0.06	171.04 ± 0.33	178.75	0.77	1.01	4.82
10	323.15	0.3	91.20 ± 0.02	137.51 ± 0.09	142.02	0.85	1.02	5.31
10	323.15	0.4	93.19 ± 0.05	111.66 ± 0.18	114.31	0.91	1.02	5.66

Continued on next page

p [MPa]	T [K]	x_{H_2}	L [Å]	ρ / [kg/m^3] ρ^{MD}	ρ^{RFP}	Z Z^{MD}	Z^{RFP}	E_{tot} [kJ/mol]
10	323.15	0.5	94.67 ± 0.01	90.09 ± 0.04	91.58	0.95	1.02	5.95
10	323.15	0.6	95.80 ± 0.02	71.06 ± 0.05	71.87	0.99	1.02	6.17
10	323.15	0.7	96.64 ± 0.02	53.78 ± 0.04	54.13	1.01	1.03	6.34
10	323.15	0.8	97.34 ± 0.04	37.50 ± 0.04	37.69	1.03	0.91	6.48
10	323.15	0.9	97.74 ± 0.01	22.11 ± 0.01	22.11	1.05	0.92	6.58
10	323.15	1.0	98.03 ± 0.02	7.11 ± 0.01	7.10	1.06	0.93	6.65
10	348.15	0.0	86.71 ± 0.18	224.16 ± 1.40	233.43	0.68	0.94	4.55
10	348.15	0.1	90.64 ± 0.06	177.55 ± 0.38	185.28	0.77	0.95	5.24
10	348.15	0.2	93.20 ± 0.08	146.10 ± 0.37	151.15	0.84	0.95	5.70
10	348.15	0.3	95.11 ± 0.04	121.25 ± 0.14	124.46	0.90	0.96	6.06
10	348.15	0.4	96.61 ± 0.03	100.22 ± 0.10	102.29	0.94	0.97	6.34
10	348.15	0.5	97.72 ± 0.04	81.91 ± 0.10	83.05	0.97	0.98	6.57
10	348.15	0.6	98.61 ± 0.04	65.16 ± 0.08	65.78	1.00	0.98	6.75
10	348.15	0.7	99.29 ± 0.02	49.58 ± 0.03	49.86	1.02	0.99	6.90
10	348.15	0.8	99.81 ± 0.04	34.78 ± 0.04	34.88	1.03	1.00	7.02
10	348.15	0.9	100.21 ± 0.03	20.51 ± 0.02	20.53	1.05	1.00	7.11
10	348.15	1.0	100.39 ± 0.03	6.62 ± 0.01	6.61	1.05	1.01	7.18
10	373.15	0.0	92.73 ± 0.08	183.33 ± 0.47	188.57	0.77	1.01	5.65
10	373.15	0.1	95.29 ± 0.07	152.81 ± 0.33	158.17	0.84	1.01	6.11
10	373.15	0.2	97.04 ± 0.07	129.41 ± 0.26	133.09	0.89	1.02	6.45
10	373.15	0.3	98.44 ± 0.07	109.35 ± 0.24	111.73	0.93	1.02	6.74
10	373.15	0.4	99.66 ± 0.04	91.30 ± 0.10	93.02	0.96	1.02	6.97
10	373.15	0.5	100.56 ± 0.02	75.16 ± 0.05	76.20	0.99	1.02	7.17

Continued on next page

p [MPa]	T [K]	x_{H_2}	L [Å]	ρ / [kg/m^3] ρ^{MD}	ρ^{RFP}	Z Z^{MD}	Z^{RFP}	E_{tot} [kJ/mol]
10	373.15	0.6	101.30 ± 0.03	60.10 ± 0.05	60.74	1.01	1.02	7.33
10	373.15	0.7	101.85 ± 0.02	45.94 ± 0.03	46.26	1.03	0.93	7.46
10	373.15	0.8	102.27 ± 0.04	32.34 ± 0.04	32.48	1.04	0.94	7.57
10	373.15	0.9	102.53 ± 0.03	19.15 ± 0.02	19.17	1.05	0.94	7.65
10	373.15	1.0	102.64 ± 0.02	6.19 ± 0.01	6.18	1.05	0.95	7.70
10	398.15	0.0	97.09 ± 0.05	159.68 ± 0.23	163.03	0.83	0.96	6.51
10	398.15	0.1	98.95 ± 0.04	136.47 ± 0.14	140.13	0.88	0.97	6.86
10	398.15	0.2	100.29 ± 0.04	117.23 ± 0.16	119.91	0.92	0.97	7.14
10	398.15	0.3	101.53 ± 0.05	99.68 ± 0.14	101.88	0.95	0.98	7.39
10	398.15	0.4	102.40 ± 0.03	84.17 ± 0.07	85.56	0.98	0.98	7.59
10	398.15	0.5	103.14 ± 0.01	69.66 ± 0.02	70.54	1.00	0.99	7.75
10	398.15	0.6	103.69 ± 0.02	56.05 ± 0.04	56.50	1.01	0.99	7.89
10	398.15	0.7	104.17 ± 0.02	42.94 ± 0.03	43.19	1.03	1.00	8.01
10	398.15	0.8	104.47 ± 0.04	30.33 ± 0.04	30.40	1.04	1.00	8.10
10	398.15	0.9	104.78 ± 0.02	17.95 ± 0.01	17.99	1.05	1.01	8.18
10	398.15	1.0	104.80 ± 0.03	5.82 ± 0.01	5.81	1.05	1.01	8.23
10	423.15	0.0	100.76 ± 0.02	142.88 ± 0.07	145.56	0.88	1.01	7.26
10	423.15	0.1	102.18 ± 0.04	123.95 ± 0.14	126.84	0.91	1.02	7.55
10	423.15	0.2	103.19 ± 0.07	107.64 ± 0.22	109.69	0.94	1.02	7.79
10	423.15	0.3	104.17 ± 0.05	92.29 ± 0.13	93.95	0.97	1.02	8.00
10	423.15	0.4	104.93 ± 0.05	78.22 ± 0.12	79.38	0.99	1.02	8.17
10	423.15	0.5	105.52 ± 0.01	65.05 ± 0.03	65.76	1.01	1.02	8.32
10	423.15	0.6	106.03 ± 0.02	52.42 ± 0.03	52.86	1.02	0.75	8.45

Continued on next page

p [MPa]	T [K]	x_{H_2}	L [Å]	ρ / [kg/m^3] ρ^{MD}	ρ^{RFP}	Z Z^{MD}	Z^{RFP}	E_{tot} [kJ/mol]
10	423.15	0.7	106.40 ± 0.03	40.30 ± 0.03	40.52	1.03	0.78	8.55
10	423.15	0.8	106.66 ± 0.02	28.51 ± 0.02	28.59	1.04	0.81	8.64
10	423.15	0.9	106.87 ± 0.02	16.91 ± 0.01	16.94	1.04	0.84	8.70
10	423.15	1.0	106.88 ± 0.01	5.48 ± 0.01	5.48	1.04	0.86	8.75
15	323.15	0.0	59.13 ± 0.04	707.13 ± 1.36	699.76	0.35	0.88	-1.16
15	323.15	0.1	67.13 ± 0.05	437.04 ± 0.97	456.58	0.51	0.90	1.91
15	323.15	0.2	73.93 ± 0.04	292.72 ± 0.53	309.70	0.68	0.92	3.61
15	323.15	0.3	77.88 ± 0.04	220.86 ± 0.37	230.33	0.79	0.93	4.50
15	323.15	0.4	80.43 ± 0.03	173.67 ± 0.17	178.43	0.87	0.95	5.12
15	323.15	0.5	82.35 ± 0.03	136.85 ± 0.17	139.54	0.94	0.96	5.56
15	323.15	0.6	83.81 ± 0.02	106.15 ± 0.09	107.77	0.99	0.97	5.90
15	323.15	0.7	84.79 ± 0.01	79.61 ± 0.04	80.26	1.02	0.98	6.17
15	323.15	0.8	85.55 ± 0.02	55.25 ± 0.03	55.45	1.05	0.99	6.37
15	323.15	0.9	86.01 ± 0.01	32.45 ± 0.01	32.37	1.07	1.00	6.51
15	323.15	1.0	86.36 ± 0.03	10.40 ± 0.01	10.36	1.08	1.01	6.62
15	348.15	0.0	68.65 ± 0.02	451.71 ± 0.47	463.34	0.50	1.01	2.16
15	348.15	0.1	75.39 ± 0.08	308.56 ± 1.02	325.46	0.67	1.02	3.88
15	348.15	0.2	79.30 ± 0.06	237.18 ± 0.52	248.25	0.78	1.02	4.80
15	348.15	0.3	81.94 ± 0.04	189.59 ± 0.29	196.44	0.86	1.03	5.43
15	348.15	0.4	83.86 ± 0.03	153.22 ± 0.14	157.36	0.92	1.03	5.89
15	348.15	0.5	85.29 ± 0.03	123.18 ± 0.12	125.55	0.97	0.82	6.24
15	348.15	0.6	86.40 ± 0.02	96.86 ± 0.06	98.22	1.01	0.84	6.53
15	348.15	0.7	87.24 ± 0.05	73.10 ± 0.12	73.80	1.04	0.86	6.75

Continued on next page

p [MPa]	T [K]	x_{H_2}	L [Å]	ρ / [kg/m^3]		Z		E_{tot} [kJ/mol]
				ρ^{MD}	ρ^{RFP}	Z^{MD}	Z^{RFP}	
15	348.15	0.8	87.80 ± 0.02	51.11 ± 0.04	51.30	1.06	0.88	6.93
15	348.15	0.9	88.20 ± 0.01	30.09 ± 0.01	30.08	1.07	0.90	7.06
15	348.15	1.0	88.42 ± 0.01	9.69 ± 0.01	9.66	1.08	0.91	7.15
15	373.15	0.0	76.99 ± 0.08	320.29 ± 1.04	332.35	0.66	0.93	4.19
15	373.15	0.1	80.78 ± 0.02	250.81 ± 0.15	261.96	0.77	0.94	5.11
15	373.15	0.2	83.41 ± 0.05	203.82 ± 0.40	211.96	0.84	0.95	5.74
15	373.15	0.3	85.29 ± 0.07	168.14 ± 0.44	173.34	0.90	0.96	6.22
15	373.15	0.4	86.74 ± 0.03	138.50 ± 0.13	141.69	0.95	0.97	6.58
15	373.15	0.5	87.88 ± 0.06	112.60 ± 0.22	114.56	0.99	0.98	6.88
15	373.15	0.6	88.73 ± 0.04	89.44 ± 0.11	90.44	1.02	0.99	7.13
15	373.15	0.7	89.44 ± 0.02	67.83 ± 0.05	68.40	1.04	1.00	7.32
15	373.15	0.8	89.91 ± 0.03	47.59 ± 0.05	47.78	1.06	1.00	7.48
15	373.15	0.9	90.23 ± 0.03	28.10 ± 0.03	28.11	1.07	1.01	7.60
15	373.15	1.0	90.37 ± 0.01	9.07 ± 0.01	9.05	1.07	1.02	7.68
15	398.15	0.0	82.26 ± 0.11	262.54 ± 1.10	270.74	0.76	1.02	5.43
15	398.15	0.1	84.87 ± 0.03	216.27 ± 0.24	224.58	0.83	1.02	6.06
15	398.15	0.2	86.74 ± 0.02	181.23 ± 0.11	187.35	0.89	1.03	6.55
15	398.15	0.3	88.13 ± 0.04	152.40 ± 0.21	156.25	0.93	1.03	6.93
15	398.15	0.4	89.35 ± 0.02	126.69 ± 0.07	129.43	0.97	0.87	7.25
15	398.15	0.5	90.26 ± 0.06	103.92 ± 0.21	105.62	1.00	0.88	7.50
15	398.15	0.6	90.98 ± 0.02	82.96 ± 0.06	83.95	1.03	0.89	7.71
15	398.15	0.7	91.51 ± 0.02	63.34 ± 0.03	63.80	1.05	0.91	7.88
15	398.15	0.8	91.90 ± 0.01	44.56 ± 0.01	44.73	1.06	0.92	8.02

Continued on next page

p [MPa]	T [K]	x_{H_2}	L [Å]	ρ / [kg/m^3] ρ^{MD}	ρ^{RFP}	Z Z^{MD}	Z^{RFP}	E_{tot} [kJ/mol]
15	398.15	0.9	92.14 ± 0.02	26.39 ± 0.02	26.39	1.07	0.93	8.13
15	398.15	1.0	92.24 ± 0.04	8.53 ± 0.01	8.52	1.07	0.94	8.21
15	423.15	0.0	86.28 ± 0.04	227.57 ± 0.29	233.93	0.82	0.95	6.39
15	423.15	0.1	88.10 ± 0.03	193.34 ± 0.18	199.18	0.88	0.96	6.88
15	423.15	0.2	89.55 ± 0.03	164.68 ± 0.18	169.18	0.92	0.97	7.27
15	423.15	0.3	90.73 ± 0.02	139.66 ± 0.09	142.91	0.96	0.98	7.60
15	423.15	0.4	91.66 ± 0.02	117.36 ± 0.08	119.47	0.99	0.99	7.87
15	423.15	0.5	92.41 ± 0.02	96.85 ± 0.06	98.16	1.01	1.00	8.10
15	423.15	0.6	93.03 ± 0.02	77.60 ± 0.04	78.42	1.03	1.00	8.28
15	423.15	0.7	93.47 ± 0.01	59.44 ± 0.02	59.83	1.05	1.01	8.44
15	423.15	0.8	93.81 ± 0.03	41.89 ± 0.04	42.07	1.06	1.01	8.57
15	423.15	0.9	93.98 ± 0.02	24.87 ± 0.01	24.88	1.07	1.02	8.66
15	423.15	1.0	94.07 ± 0.01	8.04 ± 0.01	8.04	1.07	1.02	8.73
20	323.15	0.0	57.20 ± 0.02	781.16 ± 0.72	784.30	0.42	1.02	-1.94
20	323.15	0.1	60.90 ± 0.04	585.22 ± 1.09	589.51	0.51	1.03	0.51
20	323.15	0.2	65.84 ± 0.02	414.30 ± 0.41	427.97	0.64	1.03	2.46
20	323.15	0.3	69.88 ± 0.02	305.73 ± 0.23	316.48	0.76	0.90	3.73
20	323.15	0.4	72.72 ± 0.06	235.05 ± 0.61	241.10	0.86	0.91	4.58
20	323.15	0.5	74.80 ± 0.02	182.64 ± 0.13	185.99	0.94	0.92	5.19
20	323.15	0.6	76.31 ± 0.02	140.62 ± 0.12	142.21	1.00	0.93	5.65
20	323.15	0.7	77.49 ± 0.02	104.32 ± 0.09	105.17	1.04	0.94	6.00
20	323.15	0.8	78.31 ± 0.01	72.02 ± 0.03	72.30	1.08	0.95	6.26
20	323.15	0.9	78.83 ± 0.02	42.14 ± 0.03	42.06	1.10	0.96	6.46

Continued on next page

p [MPa]	T [K]	x_{H_2}	L [Å]	ρ / [kg/m^3] ρ^{MD}	ρ^{RFP}	Z Z^{MD}	Z^{RFP}	E_{tot} [kJ/mol]
20	323.15	1.0	79.10 ± 0.03	13.53 ± 0.02	13.45	1.11	0.97	6.59
20	348.15	0.0	61.75 ± 0.08	620.72 ± 2.37	626.22	0.49	0.98	0.47
20	348.15	0.1	66.82 ± 0.07	443.10 ± 1.43	459.29	0.62	0.98	2.58
20	348.15	0.2	70.92 ± 0.07	331.49 ± 0.98	345.44	0.74	0.99	3.92
20	348.15	0.3	73.88 ± 0.10	258.72 ± 1.05	268.02	0.84	1.00	4.81
20	348.15	0.4	75.99 ± 0.02	205.96 ± 0.18	211.39	0.91	1.00	5.45
20	348.15	0.5	77.58 ± 0.03	163.66 ± 0.17	166.77	0.97	1.01	5.92
20	348.15	0.6	78.77 ± 0.01	127.83 ± 0.03	129.41	1.02	1.01	6.31
20	348.15	0.7	79.67 ± 0.01	95.96 ± 0.01	96.68	1.05	1.01	6.60
20	348.15	0.8	80.35 ± 0.01	66.67 ± 0.02	66.93	1.08	1.02	6.83
20	348.15	0.9	80.78 ± 0.03	39.16 ± 0.05	39.14	1.10	1.02	7.01
20	348.15	1.0	81.00 ± 0.03	12.60 ± 0.01	12.56	1.11	1.02	7.12
20	373.15	0.0	67.85 ± 0.06	467.88 ± 1.15	480.55	0.61	1.03	2.71
20	373.15	0.1	71.97 ± 0.05	354.59 ± 0.67	369.17	0.72	1.03	4.11
20	373.15	0.2	74.91 ± 0.01	281.37 ± 0.12	291.90	0.82	0.92	5.02
20	373.15	0.3	77.14 ± 0.04	227.24 ± 0.35	234.64	0.89	0.93	5.70
20	373.15	0.4	78.77 ± 0.03	184.91 ± 0.25	189.41	0.95	0.94	6.20
20	373.15	0.5	80.01 ± 0.02	149.24 ± 0.11	151.76	0.99	0.95	6.61
20	373.15	0.6	81.01 ± 0.02	117.54 ± 0.08	119.02	1.03	0.95	6.93
20	373.15	0.7	81.74 ± 0.05	88.88 ± 0.16	89.59	1.06	0.96	7.19
20	373.15	0.8	82.27 ± 0.02	62.12 ± 0.03	62.37	1.08	0.97	7.40
20	373.15	0.9	82.62 ± 0.02	36.60 ± 0.03	36.62	1.09	0.98	7.55
20	373.15	1.0	82.77 ± 0.02	11.81 ± 0.01	11.79	1.10	0.98	7.66

Continued on next page

p [MPa]	T [K]	x_{H_2}	L [Å]	ρ / [kg/m^3] ρ^{MD}	ρ^{RFP}	Z Z^{MD}	Z^{RFP}	E_{tot} [kJ/mol]
20	398.15	0.0	73.04 ± 0.01	375.14 ± 0.15	385.81	0.71	0.99	4.29
20	398.15	0.1	76.04 ± 0.01	300.72 ± 0.08	311.92	0.80	0.99	5.26
20	398.15	0.2	78.29 ± 0.07	246.42 ± 0.66	255.41	0.87	1.00	5.96
20	398.15	0.3	79.97 ± 0.02	203.99 ± 0.18	210.16	0.93	1.00	6.48
20	398.15	0.4	81.25 ± 0.01	168.46 ± 0.07	172.36	0.98	1.01	6.91
20	398.15	0.5	82.26 ± 0.02	137.28 ± 0.11	139.63	1.01	1.01	7.26
20	398.15	0.6	83.06 ± 0.03	109.03 ± 0.13	110.38	1.04	1.02	7.54
20	398.15	0.7	83.63 ± 0.02	82.97 ± 0.07	83.56	1.06	1.02	7.77
20	398.15	0.8	84.08 ± 0.02	58.20 ± 0.05	58.42	1.08	1.02	7.95
20	398.15	0.9	84.33 ± 0.01	34.42 ± 0.01	34.42	1.09	1.02	8.08
20	398.15	1.0	84.43 ± 0.03	11.12 ± 0.01	11.11	1.09	1.02	8.19
20	423.15	0.0	77.21 ± 0.02	317.52 ± 0.20	327.10	0.79	1.02	5.49
20	423.15	0.1	79.40 ± 0.01	264.10 ± 0.12	273.25	0.86	0.73	6.21
20	423.15	0.2	81.07 ± 0.03	221.98 ± 0.27	228.84	0.91	0.76	6.76
20	423.15	0.3	82.44 ± 0.03	186.20 ± 0.23	191.27	0.96	0.79	7.21
20	423.15	0.4	83.48 ± 0.04	155.35 ± 0.21	158.64	1.00	0.82	7.58
20	423.15	0.5	84.32 ± 0.04	127.48 ± 0.18	129.57	1.03	0.85	7.88
20	423.15	0.6	84.97 ± 0.02	101.85 ± 0.08	103.05	1.05	0.87	8.13
20	423.15	0.7	85.43 ± 0.02	77.85 ± 0.07	78.37	1.07	0.89	8.33
20	423.15	0.8	85.78 ± 0.02	54.79 ± 0.04	54.98	1.08	0.91	8.49
20	423.15	0.9	85.99 ± 0.03	32.47 ± 0.03	32.47	1.09	0.93	8.62
20	423.15	1.0	86.09 ± 0.01	10.50 ± 0.01	10.50	1.09	0.94	8.72
25	323.15	0.0	56.08 ± 0.03	828.89 ± 1.24	834.21	0.49	0.96	-2.45

Continued on next page

p [MPa]	T [K]	x_{H_2}	L [Å]	ρ / [kg/m^3] ρ^{MD}	ρ^{RFP}	Z Z^{MD}	Z^{RFP}	E_{tot} [kJ/mol]
25	323.15	0.1	58.28 ± 0.02	667.96 ± 0.53	660.61	0.55	0.97	-0.28
25	323.15	0.2	61.61 ± 0.02	505.83 ± 0.38	508.88	0.66	0.98	1.64
25	323.15	0.3	64.90 ± 0.04	381.66 ± 0.64	387.88	0.77	0.99	3.08
25	323.15	0.4	67.64 ± 0.03	292.10 ± 0.45	297.32	0.87	1.00	4.10
25	323.15	0.5	69.69 ± 0.01	225.83 ± 0.14	228.86	0.95	1.01	4.85
25	323.15	0.6	71.27 ± 0.01	172.58 ± 0.10	174.38	1.01	1.02	5.42
25	323.15	0.7	72.43 ± 0.02	127.74 ± 0.10	128.55	1.06	1.02	5.84
25	323.15	0.8	73.23 ± 0.03	88.09 ± 0.11	88.16	1.10	1.03	6.16
25	323.15	0.9	73.80 ± 0.02	51.36 ± 0.04	51.21	1.13	1.03	6.40
25	323.15	1.0	74.09 ± 0.01	16.46 ± 0.01	16.38	1.14	1.03	6.56
25	348.15	0.0	59.24 ± 0.02	703.21 ± 0.67	711.60	0.54	0.80	-0.37
25	348.15	0.1	62.44 ± 0.02	543.13 ± 0.46	551.93	0.63	0.83	1.64
25	348.15	0.2	65.79 ± 0.04	415.37 ± 0.75	426.44	0.74	0.85	3.16
25	348.15	0.3	68.49 ± 0.03	324.69 ± 0.41	332.46	0.84	0.87	4.24
25	348.15	0.4	70.67 ± 0.01	256.10 ± 0.08	261.47	0.92	0.89	5.03
25	348.15	0.5	72.27 ± 0.02	202.45 ± 0.18	205.40	0.98	0.90	5.63
25	348.15	0.6	73.58 ± 0.01	156.84 ± 0.08	158.79	1.04	0.92	6.11
25	348.15	0.7	74.50 ± 0.01	117.40 ± 0.05	118.27	1.08	0.93	6.46
25	348.15	0.8	75.17 ± 0.01	81.45 ± 0.04	81.71	1.10	0.95	6.74
25	348.15	0.9	75.61 ± 0.02	47.76 ± 0.04	47.73	1.12	0.96	6.95
25	348.15	1.0	75.81 ± 0.05	15.37 ± 0.03	15.32	1.13	0.97	7.10
25	373.15	0.0	63.31 ± 0.03	576.06 ± 0.79	588.46	0.62	0.98	1.63
25	373.15	0.1	66.61 ± 0.01	447.32 ± 0.23	460.40	0.72	0.99	3.23

Continued on next page

p [MPa]	T [K]	x_{H_2}	L [Å]	ρ / [kg/m^3] ρ^{MD}	ρ^{RFP}	Z Z^{MD}	Z^{RFP}	E_{tot} [kJ/mol]
25	373.15	0.2	69.46 ± 0.02	352.87 ± 0.26	364.55	0.81	1.00	4.38
25	373.15	0.3	71.58 ± 0.03	284.39 ± 0.42	291.79	0.89	1.01	5.21
25	373.15	0.4	73.30 ± 0.04	229.43 ± 0.33	234.34	0.96	1.01	5.86
25	373.15	0.5	74.63 ± 0.03	183.89 ± 0.21	186.93	1.01	1.02	6.36
25	373.15	0.6	75.62 ± 0.03	144.48 ± 0.18	146.10	1.05	1.02	6.75
25	373.15	0.7	76.40 ± 0.02	108.85 ± 0.08	109.68	1.08	1.03	7.07
25	373.15	0.8	76.93 ± 0.01	75.95 ± 0.02	76.21	1.10	1.03	7.31
25	373.15	0.9	77.28 ± 0.03	44.72 ± 0.05	44.71	1.12	1.03	7.50
25	373.15	1.0	77.41 ± 0.02	14.43 ± 0.01	14.40	1.13	0.85	7.63
25	398.15	0.0	67.50 ± 0.05	475.26 ± 1.15	487.81	0.70	0.87	3.30
25	398.15	0.1	70.37 ± 0.03	379.36 ± 0.50	392.67	0.79	0.89	4.52
25	398.15	0.2	72.61 ± 0.04	309.00 ± 0.55	319.46	0.87	0.90	5.40
25	398.15	0.3	74.28 ± 0.04	254.49 ± 0.36	261.22	0.93	0.91	6.07
25	398.15	0.4	75.64 ± 0.03	208.86 ± 0.27	213.13	0.98	0.93	6.59
25	398.15	0.5	76.70 ± 0.02	169.41 ± 0.15	171.96	1.03	0.94	7.03
25	398.15	0.6	77.54 ± 0.04	134.02 ± 0.19	135.52	1.06	0.95	7.38
25	398.15	0.7	78.14 ± 0.02	101.72 ± 0.08	102.37	1.08	0.96	7.65
25	398.15	0.8	78.58 ± 0.01	71.28 ± 0.02	71.46	1.10	0.97	7.88
25	398.15	0.9	78.89 ± 0.02	42.05 ± 0.03	42.06	1.12	0.98	8.04
25	398.15	1.0	78.97 ± 0.01	13.60 ± 0.01	13.58	1.12	0.99	8.17
25	423.15	0.0	71.25 ± 0.06	404.06 ± 1.05	415.50	0.77	1.00	4.65
25	423.15	0.1	73.52 ± 0.02	332.75 ± 0.21	343.87	0.85	1.00	5.57
25	423.15	0.2	75.27 ± 0.02	277.35 ± 0.19	285.81	0.91	1.01	6.28

Continued on next page

p [MPa]	T [K]	x_{H_2}	L [Å]	ρ / [kg/m^3] ρ^{MD}	ρ^{RFP}	Z Z^{MD}	Z^{RFP}	E_{tot} [kJ/mol]
25	423.15	0.3	76.64 ± 0.03	231.74 ± 0.30	237.46	0.96	1.01	6.84
25	423.15	0.4	77.77 ± 0.02	192.11 ± 0.16	196.03	1.01	1.02	7.30
25	423.15	0.5	78.63 ± 0.01	157.21 ± 0.04	159.54	1.04	1.02	7.67
25	423.15	0.6	79.32 ± 0.01	125.21 ± 0.07	126.55	1.07	1.03	7.98
25	423.15	0.7	79.82 ± 0.03	95.43 ± 0.09	96.05	1.09	1.03	8.23
25	423.15	0.8	80.18 ± 0.02	67.10 ± 0.05	67.30	1.10	1.03	8.43
25	423.15	0.9	80.40 ± 0.02	39.72 ± 0.03	39.73	1.11	0.89	8.58
25	423.15	1.0	80.46 ± 0.02	12.85 ± 0.01	12.85	1.11	0.90	8.70
30	323.15	0.0	55.37 ± 0.01	860.84 ± 0.21	870.43	0.57	0.91	-2.81
30	323.15	0.1	56.85 ± 0.04	719.62 ± 1.66	707.86	0.62	0.92	-0.78
30	323.15	0.2	59.18 ± 0.01	570.68 ± 0.35	564.61	0.70	0.93	1.08
30	323.15	0.3	61.73 ± 0.03	443.56 ± 0.55	443.13	0.79	0.95	2.55
30	323.15	0.4	64.04 ± 0.03	344.08 ± 0.51	345.05	0.88	0.96	3.69
30	323.15	0.5	66.00 ± 0.01	265.79 ± 0.15	267.16	0.97	0.96	4.55
30	323.15	0.6	67.53 ± 0.03	202.93 ± 0.30	203.82	1.04	0.97	5.20
30	323.15	0.7	68.67 ± 0.02	149.90 ± 0.16	150.25	1.09	0.98	5.70
30	323.15	0.8	69.49 ± 0.01	103.06 ± 0.04	103.01	1.13	0.99	6.07
30	323.15	0.9	70.04 ± 0.01	60.06 ± 0.03	59.84	1.16	1.00	6.35
30	323.15	1.0	70.31 ± 0.02	19.26 ± 0.01	19.15	1.17	1.00	6.54
30	348.15	0.0	57.79 ± 0.01	757.25 ± 0.23	766.83	0.60	1.01	-0.95
30	348.15	0.1	59.96 ± 0.03	613.18 ± 1.05	614.98	0.67	1.01	0.98
30	348.15	0.2	62.48 ± 0.03	484.96 ± 0.67	488.55	0.76	1.02	2.54
30	348.15	0.3	64.86 ± 0.04	382.34 ± 0.62	386.81	0.85	1.02	3.76

Continued on next page

p	T	x_{H_2}	L	ρ / [kg/m^3]		Z		E_{tot}
[MPa]	[K]		[Å]	ρ^{MD}	ρ^{RFP}	Z^{MD}	Z^{RFP}	[kJ/mol]
30	348.15	0.4	66.88 ± 0.04	302.13 ± 0.51	305.97	0.93	1.02	4.67
30	348.15	0.5	68.44 ± 0.01	238.40 ± 0.14	240.69	1.00	1.03	5.37
30	348.15	0.6	69.66 ± 0.01	184.83 ± 0.04	186.04	1.05	1.03	5.91
30	348.15	0.7	70.62 ± 0.01	137.79 ± 0.06	138.49	1.10	1.03	6.34
30	348.15	0.8	71.26 ± 0.02	95.58 ± 0.08	95.63	1.13	0.91	6.66
30	348.15	0.9	71.68 ± 0.03	56.04 ± 0.08	55.85	1.15	0.92	6.91
30	348.15	1.0	71.90 ± 0.01	18.01 ± 0.01	17.94	1.16	0.93	7.08
30	373.15	0.0	60.82 ± 0.03	649.67 ± 0.82	661.87	0.66	0.94	0.88
30	373.15	0.1	63.29 ± 0.05	521.50 ± 1.20	530.51	0.74	0.95	2.53
30	373.15	0.2	65.66 ± 0.02	417.72 ± 0.43	425.73	0.82	0.96	3.80
30	373.15	0.3	67.73 ± 0.05	335.74 ± 0.74	342.49	0.90	0.97	4.78
30	373.15	0.4	69.35 ± 0.03	270.93 ± 0.40	275.36	0.97	0.97	5.53
30	373.15	0.5	70.62 ± 0.02	217.02 ± 0.17	219.53	1.03	0.98	6.11
30	373.15	0.6	71.62 ± 0.02	170.09 ± 0.15	171.44	1.07	0.99	6.58
30	373.15	0.7	72.39 ± 0.02	127.94 ± 0.11	128.60	1.10	0.99	6.95
30	373.15	0.8	72.92 ± 0.01	89.22 ± 0.04	89.32	1.13	1.00	7.24
30	373.15	0.9	73.26 ± 0.01	52.49 ± 0.01	52.39	1.14	1.01	7.46
30	373.15	1.0	73.39 ± 0.02	16.94 ± 0.01	16.88	1.15	1.01	7.62
30	398.15	0.0	64.16 ± 0.05	553.42 ± 1.35	567.77	0.72	1.01	2.52
30	398.15	0.1	66.56 ± 0.03	448.30 ± 0.63	461.20	0.80	1.02	3.87
30	398.15	0.2	68.54 ± 0.01	367.35 ± 0.19	376.41	0.88	1.02	4.88
30	398.15	0.3	70.21 ± 0.03	301.41 ± 0.35	307.85	0.94	1.02	5.68
30	398.15	0.4	71.53 ± 0.02	246.88 ± 0.19	250.93	1.00	1.03	6.30

Continued on next page

p [MPa]	T [K]	x_{H_2}	L [Å]	ρ / [kg/m^3] ρ^{MD}	ρ^{RFP}	Z Z^{MD}	Z^{RFP}	E_{tot} [kJ/mol]
30	398.15	0.5	72.60 ± 0.03	199.70 ± 0.26	202.22	1.04	1.03	6.81
30	398.15	0.6	73.43 ± 0.03	157.79 ± 0.17	159.20	1.08	1.03	7.22
30	398.15	0.7	74.07 ± 0.02	119.41 ± 0.09	120.16	1.11	0.70	7.54
30	398.15	0.8	74.50 ± 0.02	83.66 ± 0.06	83.84	1.13	0.74	7.81
30	398.15	0.9	74.74 ± 0.02	49.43 ± 0.04	49.35	1.14	0.77	8.01
30	398.15	1.0	74.87 ± 0.01	15.96 ± 0.01	15.94	1.15	0.80	8.15
30	423.15	0.0	67.35 ± 0.03	478.38 ± 0.65	491.99	0.78	0.83	3.92
30	423.15	0.1	69.38 ± 0.01	395.89 ± 0.14	407.18	0.86	0.86	4.99
30	423.15	0.2	71.08 ± 0.02	329.34 ± 0.28	338.00	0.92	0.88	5.83
30	423.15	0.3	72.43 ± 0.02	274.51 ± 0.24	280.35	0.98	0.90	6.49
30	423.15	0.4	73.55 ± 0.01	227.16 ± 0.12	231.05	1.02	0.92	7.03
30	423.15	0.5	74.44 ± 0.01	185.26 ± 0.05	187.78	1.06	0.94	7.48
30	423.15	0.6	75.13 ± 0.02	147.35 ± 0.10	148.79	1.09	0.95	7.84
30	423.15	0.7	75.65 ± 0.01	112.10 ± 0.06	112.85	1.11	0.97	8.13
30	423.15	0.8	75.98 ± 0.01	78.84 ± 0.03	79.04	1.13	0.98	8.37
30	423.15	0.9	76.19 ± 0.04	46.68 ± 0.07	46.66	1.14	0.99	8.55
30	423.15	1.0	76.24 ± 0.02	15.11 ± 0.01	15.11	1.14	1.00	8.68
35	323.15	0.0	54.79 ± 0.02	888.51 ± 0.80	899.26	0.65	1.01	-3.12
35	323.15	0.1	55.89 ± 0.01	757.42 ± 0.56	743.29	0.68	1.02	-1.16
35	323.15	0.2	57.57 ± 0.02	619.71 ± 0.67	605.99	0.75	1.02	0.63
35	323.15	0.3	59.54 ± 0.04	494.14 ± 0.92	486.19	0.83	1.03	2.13
35	323.15	0.4	61.51 ± 0.02	388.28 ± 0.35	384.86	0.91	1.03	3.33
35	323.15	0.5	63.26 ± 0.02	301.89 ± 0.34	300.73	0.99	1.04	4.28

Continued on next page

p [MPa]	T [K]	x_{H_2}	L [Å]	ρ / [kg/m^3] ρ^{MD}	ρ^{RFP}	Z Z^{MD}	Z^{RFP}	E_{tot} [kJ/mol]
35	323.15	0.6	64.66 ± 0.01	231.07 ± 0.06	230.50	1.06	0.79	5.00
35	323.15	0.7	65.77 ± 0.01	170.57 ± 0.08	170.28	1.12	0.81	5.56
35	323.15	0.8	66.58 ± 0.01	117.17 ± 0.06	116.88	1.16	0.83	5.99
35	323.15	0.9	67.09 ± 0.01	68.36 ± 0.05	67.96	1.18	0.86	6.30
35	323.15	1.0	67.35 ± 0.02	21.92 ± 0.02	21.78	1.20	0.88	6.52
35	348.15	0.0	56.85 ± 0.01	795.33 ± 0.39	807.54	0.67	0.90	-1.35
35	348.15	0.1	58.41 ± 0.02	663.50 ± 0.77	661.32	0.73	0.91	0.49
35	348.15	0.2	60.33 ± 0.03	538.57 ± 0.80	536.41	0.80	0.93	2.07
35	348.15	0.3	62.34 ± 0.02	430.57 ± 0.49	431.57	0.88	0.94	3.35
35	348.15	0.4	64.06 ± 0.01	343.86 ± 0.21	344.69	0.96	0.96	4.33
35	348.15	0.5	65.54 ± 0.01	271.50 ± 0.04	272.47	1.02	0.97	5.12
35	348.15	0.6	66.70 ± 0.03	210.51 ± 0.30	211.08	1.08	0.98	5.73
35	348.15	0.7	67.60 ± 0.03	157.09 ± 0.18	157.31	1.12	0.99	6.21
35	348.15	0.8	68.24 ± 0.01	108.85 ± 0.02	108.71	1.16	1.00	6.59
35	348.15	0.9	68.66 ± 0.01	63.77 ± 0.02	63.54	1.18	1.01	6.86
35	348.15	1.0	68.83 ± 0.01	20.53 ± 0.01	20.44	1.19	1.01	7.06
35	373.15	0.0	59.27 ± 0.03	701.93 ± 0.89	715.25	0.71	1.02	0.34
35	373.15	0.1	61.14 ± 0.02	578.40 ± 0.64	584.25	0.78	1.02	1.99
35	373.15	0.2	63.09 ± 0.01	470.85 ± 0.14	475.76	0.85	1.03	3.34
35	373.15	0.3	64.86 ± 0.01	382.41 ± 0.26	386.27	0.93	1.03	4.39
35	373.15	0.4	66.34 ± 0.02	309.47 ± 0.22	312.04	0.99	1.03	5.23
35	373.15	0.5	67.58 ± 0.03	247.64 ± 0.35	249.37	1.05	0.84	5.89
35	373.15	0.6	68.53 ± 0.01	194.10 ± 0.12	194.96	1.09	0.86	6.42

Continued on next page

p [MPa]	T [K]	x_{H_2}	L [Å]	ρ / [kg/m^3] ρ^{MD}	ρ^{RFP}	Z Z^{MD}	Z^{RFP}	E_{tot} [kJ/mol]
35	373.15	0.7	69.30 ± 0.01	145.81 ± 0.03	146.34	1.13	0.88	6.84
35	373.15	0.8	69.81 ± 0.02	101.69 ± 0.11	101.69	1.16	0.89	7.17
35	373.15	0.9	70.12 ± 0.02	59.87 ± 0.04	59.68	1.17	0.91	7.42
35	373.15	1.0	70.24 ± 0.03	19.32 ± 0.02	19.26	1.18	0.92	7.60
35	398.15	0.0	61.97 ± 0.02	614.19 ± 0.62	629.17	0.76	0.94	1.92
35	398.15	0.1	63.89 ± 0.03	506.98 ± 0.64	517.25	0.83	0.95	3.33
35	398.15	0.2	65.64 ± 0.02	418.21 ± 0.42	425.44	0.90	0.96	4.44
35	398.15	0.3	67.17 ± 0.02	344.23 ± 0.34	349.43	0.96	0.97	5.33
35	398.15	0.4	68.44 ± 0.02	281.94 ± 0.26	285.43	1.02	0.98	6.04
35	398.15	0.5	69.43 ± 0.02	228.32 ± 0.18	230.25	1.07	0.99	6.61
35	398.15	0.6	70.25 ± 0.02	180.23 ± 0.14	181.36	1.10	1.00	7.07
35	398.15	0.7	70.85 ± 0.01	136.48 ± 0.06	136.93	1.13	1.00	7.45
35	398.15	0.8	71.28 ± 0.03	95.49 ± 0.11	95.58	1.15	1.01	7.75
35	398.15	0.9	71.54 ± 0.02	56.38 ± 0.06	56.29	1.17	1.02	7.97
35	398.15	1.0	71.62 ± 0.01	18.22 ± 0.01	18.21	1.17	1.02	8.13
35	423.15	0.0	64.66 ± 0.04	540.61 ± 0.94	555.23	0.81	1.03	3.30
35	423.15	0.1	66.41 ± 0.01	451.50 ± 0.12	461.84	0.88	1.03	4.48
35	423.15	0.2	68.00 ± 0.05	376.18 ± 0.75	384.53	0.94	1.03	5.42
35	423.15	0.3	69.29 ± 0.02	313.63 ± 0.31	319.40	1.00	1.03	6.18
35	423.15	0.4	70.31 ± 0.01	260.04 ± 0.10	263.41	1.04	0.88	6.79
35	423.15	0.5	71.19 ± 0.02	211.87 ± 0.14	214.15	1.08	0.89	7.29
35	423.15	0.6	71.84 ± 0.01	168.52 ± 0.08	169.72	1.11	0.91	7.70
35	423.15	0.7	72.31 ± 0.01	128.36 ± 0.06	128.76	1.13	0.92	8.04

Continued on next page

p [MPa]	T [K]	x_{H_2}	L [Å]	ρ / [kg/m^3] ρ^{MD}	ρ^{RFP}	Z Z^{MD}	Z^{RFP}	E_{tot} [kJ/mol]
35	423.15	0.8	72.71 ± 0.01	89.99 ± 0.06	90.21	1.15	0.93	8.31
35	423.15	0.9	72.86 ± 0.01	53.37 ± 0.03	53.28	1.16	0.94	8.52
35	423.15	1.0	72.89 ± 0.04	17.29 ± 0.03	17.27	1.16	0.95	8.67
40	323.15	0.0	54.34 ± 0.01	910.98 ± 0.22	923.33	0.72	0.96	-3.37
40	323.15	0.1	55.17 ± 0.01	787.52 ± 0.23	771.80	0.75	0.97	-1.47
40	323.15	0.2	56.40 ± 0.01	659.29 ± 0.45	638.61	0.80	0.98	0.28
40	323.15	0.3	57.97 ± 0.02	535.40 ± 0.44	520.84	0.87	0.99	1.78
40	323.15	0.4	59.63 ± 0.02	426.27 ± 0.41	418.17	0.95	1.00	3.04
40	323.15	0.5	61.18 ± 0.01	333.79 ± 0.15	330.08	1.03	1.00	4.03
40	323.15	0.6	62.43 ± 0.03	256.74 ± 0.40	254.52	1.09	1.01	4.83
40	323.15	0.7	63.46 ± 0.01	189.88 ± 0.11	188.70	1.15	1.01	5.44
40	323.15	0.8	64.23 ± 0.01	130.55 ± 0.05	129.81	1.19	1.02	5.91
40	323.15	0.9	64.70 ± 0.01	76.22 ± 0.04	75.61	1.21	1.02	6.25
40	323.15	1.0	64.93 ± 0.01	24.45 ± 0.01	24.28	1.23	1.03	6.50
40	348.15	0.0	56.12 ± 0.01	827.03 ± 0.55	839.93	0.74	1.03	-1.70
40	348.15	0.1	57.26 ± 0.01	704.14 ± 0.23	697.55	0.78	1.03	0.09
40	348.15	0.2	58.76 ± 0.01	582.93 ± 0.35	574.58	0.84	1.03	1.67
40	348.15	0.3	60.37 ± 0.01	474.25 ± 0.06	468.67	0.92	0.91	2.98
40	348.15	0.4	61.93 ± 0.02	380.42 ± 0.33	378.17	0.99	0.92	4.05
40	348.15	0.5	63.25 ± 0.02	302.03 ± 0.25	300.85	1.05	0.93	4.90
40	348.15	0.6	64.33 ± 0.02	234.66 ± 0.20	233.98	1.11	0.94	5.57
40	348.15	0.7	65.19 ± 0.01	175.21 ± 0.11	174.78	1.15	0.95	6.10
40	348.15	0.8	65.80 ± 0.01	121.40 ± 0.05	120.98	1.19	0.96	6.51

Continued on next page

p [MPa]	T [K]	x_{H_2}	L [Å]	ρ / [kg/m^3] ρ^{MD}	ρ^{RFP}	Z Z^{MD}	Z^{RFP}	E_{tot} [kJ/mol]
40	348.15	0.9	66.18 ± 0.01	71.22 ± 0.01	70.81	1.21	0.96	6.82
40	348.15	1.0	66.33 ± 0.02	22.94 ± 0.03	22.82	1.21	0.97	7.04
40	373.15	0.0	58.19 ± 0.01	741.73 ± 0.39	756.66	0.77	0.98	-0.08
40	373.15	0.1	59.57 ± 0.01	625.53 ± 0.36	626.81	0.82	0.99	1.55
40	373.15	0.2	61.14 ± 0.04	517.54 ± 1.06	516.96	0.89	0.99	2.93
40	373.15	0.3	62.65 ± 0.01	424.31 ± 0.25	423.84	0.95	1.00	4.06
40	373.15	0.4	64.00 ± 0.01	344.74 ± 0.22	344.61	1.02	1.01	4.96
40	373.15	0.5	65.20 ± 0.01	275.80 ± 0.09	276.50	1.08	1.01	5.69
40	373.15	0.6	66.10 ± 0.01	216.37 ± 0.12	216.69	1.12	1.02	6.27
40	373.15	0.7	66.77 ± 0.01	163.01 ± 0.06	162.93	1.16	1.02	6.74
40	373.15	0.8	67.29 ± 0.01	113.53 ± 0.06	113.36	1.18	1.02	7.10
40	373.15	0.9	67.58 ± 0.01	66.88 ± 0.02	66.62	1.20	1.03	7.38
40	373.15	1.0	67.69 ± 0.02	21.59 ± 0.02	21.53	1.20	1.03	7.58
40	398.15	0.0	60.44 ± 0.03	662.11 ± 0.91	677.62	0.80	1.03	1.43
40	398.15	0.1	61.96 ± 0.03	555.88 ± 0.91	563.19	0.87	1.03	2.87
40	398.15	0.2	63.45 ± 0.02	463.03 ± 0.44	467.25	0.93	0.67	4.05
40	398.15	0.3	64.81 ± 0.01	383.13 ± 0.19	386.12	0.99	0.71	5.01
40	398.15	0.4	65.96 ± 0.01	314.87 ± 0.21	316.61	1.04	0.75	5.79
40	398.15	0.5	66.94 ± 0.02	254.84 ± 0.19	256.02	1.09	0.79	6.42
40	398.15	0.6	67.69 ± 0.02	201.42 ± 0.15	202.00	1.13	0.82	6.94
40	398.15	0.7	68.28 ± 0.01	152.45 ± 0.06	152.70	1.16	0.85	7.35
40	398.15	0.8	68.67 ± 0.01	106.81 ± 0.06	106.70	1.18	0.87	7.68
40	398.15	0.9	68.91 ± 0.01	63.07 ± 0.01	62.91	1.19	0.90	7.94

Continued on next page

p [MPa]	T [K]	x_{H_2}	L [Å]	ρ / [kg/m^3] ρ^{MD}	ρ^{RFP}	Z Z^{MD}	Z^{RFP}	E_{tot} [kJ/mol]
40	398.15	1.0	68.96 ± 0.01	20.41 ± 0.01	20.38	1.19	0.92	8.12
40	423.15	0.0	62.74 ± 0.01	591.71 ± 0.12	607.12	0.85	0.93	2.80
40	423.15	0.1	64.21 ± 0.04	499.50 ± 1.01	508.45	0.91	0.95	4.04
40	423.15	0.2	65.61 ± 0.02	418.71 ± 0.34	425.44	0.97	0.97	5.06
40	423.15	0.3	66.78 ± 0.03	350.23 ± 0.47	354.58	1.02	0.98	5.88
40	423.15	0.4	67.78 ± 0.03	290.22 ± 0.34	293.10	1.07	0.99	6.56
40	423.15	0.5	68.61 ± 0.02	236.67 ± 0.18	238.64	1.11	1.00	7.12
40	423.15	0.6	69.20 ± 0.02	188.51 ± 0.17	189.35	1.13	1.01	7.58
40	423.15	0.7	69.68 ± 0.01	143.46 ± 0.07	143.79	1.16	1.02	7.95
40	423.15	0.8	70.01 ± 0.01	100.81 ± 0.05	100.83	1.17	1.03	8.25
40	423.15	0.9	70.17 ± 0.02	59.74 ± 0.05	59.62	1.18	1.03	8.49
40	423.15	1.0	70.20 ± 0.01	19.35 ± 0.01	19.36	1.18	1.04	8.66
45	323.15	0.0	53.97 ± 0.01	929.81 ± 0.35	944.10	0.79	1.04	-3.58
45	323.15	0.1	54.58 ± 0.01	813.27 ± 0.25	795.72	0.82	0.77	-1.72
45	323.15	0.2	55.55 ± 0.01	690.02 ± 0.46	665.50	0.86	0.80	-0.00
45	323.15	0.3	56.80 ± 0.02	569.36 ± 0.51	549.52	0.92	0.82	1.51
45	323.15	0.4	58.19 ± 0.01	458.77 ± 0.15	446.44	0.99	0.85	2.78
45	323.15	0.5	59.48 ± 0.03	363.21 ± 0.49	355.76	1.06	0.87	3.82
45	323.15	0.6	60.65 ± 0.01	280.05 ± 0.16	276.17	1.13	0.89	4.66
45	323.15	0.7	61.58 ± 0.02	207.88 ± 0.20	205.63	1.18	0.91	5.32
45	323.15	0.8	62.29 ± 0.01	143.11 ± 0.02	141.88	1.22	0.92	5.83
45	323.15	0.9	62.73 ± 0.01	83.61 ± 0.05	82.83	1.25	0.94	6.21
45	323.15	1.0	62.92 ± 0.02	26.88 ± 0.03	26.66	1.26	0.95	6.48

Continued on next page

p [MPa]	T [K]	x_{H_2}	L [Å]	ρ / [kg/m^3] ρ^{MD}	ρ^{RFP}	Z Z^{MD}	Z^{RFP}	E_{tot} [kJ/mol]
45	348.15	0.0	55.56 ± 0.02	852.22 ± 0.87	866.91	0.80	0.97	-1.98
45	348.15	0.1	56.44 ± 0.01	735.24 ± 0.42	727.29	0.84	0.98	-0.21
45	348.15	0.2	57.60 ± 0.01	618.98 ± 0.36	606.03	0.89	0.99	1.34
45	348.15	0.3	58.92 ± 0.02	509.89 ± 0.60	499.92	0.96	1.00	2.69
45	348.15	0.4	60.25 ± 0.02	413.28 ± 0.50	407.17	1.02	1.01	3.80
45	348.15	0.5	61.42 ± 0.03	329.90 ± 0.44	326.21	1.08	1.01	4.69
45	348.15	0.6	62.42 ± 0.01	256.88 ± 0.08	254.88	1.14	1.02	5.42
45	348.15	0.7	63.22 ± 0.02	192.09 ± 0.17	190.99	1.18	1.03	6.00
45	348.15	0.8	63.78 ± 0.01	133.30 ± 0.08	132.51	1.21	1.03	6.45
45	348.15	0.9	64.12 ± 0.01	78.29 ± 0.03	77.70	1.23	1.03	6.78
45	348.15	1.0	64.26 ± 0.01	25.23 ± 0.02	25.09	1.24	1.04	7.02
45	373.15	0.0	57.36 ± 0.01	774.60 ± 0.08	790.38	0.82	0.83	-0.42
45	373.15	0.1	58.43 ± 0.02	662.77 ± 0.58	661.72	0.87	0.85	1.19
45	373.15	0.2	59.70 ± 0.01	555.92 ± 0.16	551.40	0.93	0.87	2.59
45	373.15	0.3	61.01 ± 0.03	459.31 ± 0.57	456.17	0.99	0.88	3.76
45	373.15	0.4	62.18 ± 0.01	375.85 ± 0.26	373.46	1.05	0.90	4.72
45	373.15	0.5	63.22 ± 0.02	302.42 ± 0.23	301.08	1.10	0.92	5.50
45	373.15	0.6	64.07 ± 0.03	237.55 ± 0.36	236.76	1.15	0.93	6.13
45	373.15	0.7	64.72 ± 0.01	179.05 ± 0.03	178.42	1.18	0.94	6.63
45	373.15	0.8	65.20 ± 0.02	124.82 ± 0.10	124.38	1.21	0.96	7.04
45	373.15	0.9	65.44 ± 0.01	73.65 ± 0.02	73.20	1.22	0.97	7.35
45	373.15	1.0	65.52 ± 0.03	23.80 ± 0.03	23.71	1.23	0.98	7.56
45	398.15	0.0	59.30 ± 0.02	701.05 ± 0.74	717.19	0.85	0.99	1.04

Continued on next page

p [MPa]	T [K]	x_{H_2}	L [Å]	ρ / [kg/m^3] ρ^{MD}	ρ^{RFP}	Z Z^{MD}	Z^{RFP}	E_{tot} [kJ/mol]
45	398.15	0.1	60.49 ± 0.03	597.33 ± 0.74	601.45	0.91	1.00	2.48
45	398.15	0.2	61.79 ± 0.02	501.23 ± 0.47	503.08	0.97	1.00	3.72
45	398.15	0.3	62.97 ± 0.01	417.82 ± 0.29	418.41	1.02	1.01	4.73
45	398.15	0.4	64.03 ± 0.01	344.28 ± 0.04	344.72	1.07	1.02	5.57
45	398.15	0.5	64.88 ± 0.01	279.80 ± 0.15	279.70	1.12	1.02	6.25
45	398.15	0.6	65.61 ± 0.01	221.27 ± 0.13	221.20	1.16	1.03	6.81
45	398.15	0.7	66.14 ± 0.01	167.76 ± 0.10	167.52	1.18	1.03	7.26
45	398.15	0.8	66.51 ± 0.01	117.55 ± 0.03	117.23	1.20	1.03	7.62
45	398.15	0.9	66.72 ± 0.03	69.50 ± 0.09	69.22	1.22	1.04	7.90
45	398.15	1.0	66.73 ± 0.01	22.53 ± 0.01	22.47	1.22	0.87	8.10
45	423.15	0.0	61.35 ± 0.01	633.03 ± 0.32	650.34	0.89	0.88	2.39
45	423.15	0.1	62.54 ± 0.01	540.49 ± 0.33	548.22	0.94	0.90	3.65
45	423.15	0.2	63.69 ± 0.01	457.86 ± 0.07	461.30	0.99	0.91	4.73
45	423.15	0.3	64.76 ± 0.03	384.13 ± 0.45	386.15	1.05	0.92	5.61
45	423.15	0.4	65.70 ± 0.01	318.66 ± 0.12	320.20	1.09	0.94	6.35
45	423.15	0.5	66.47 ± 0.01	260.26 ± 0.06	261.34	1.13	0.95	6.96
45	423.15	0.6	67.04 ± 0.01	207.35 ± 0.10	207.73	1.16	0.96	7.46
45	423.15	0.7	67.50 ± 0.02	157.83 ± 0.11	157.98	1.18	0.97	7.87
45	423.15	0.8	67.78 ± 0.01	111.09 ± 0.02	110.93	1.20	0.98	8.20
45	423.15	0.9	67.94 ± 0.01	65.81 ± 0.01	65.67	1.21	0.99	8.45
45	423.15	1.0	67.93 ± 0.01	21.36 ± 0.01	21.36	1.21	1.00	8.64
50	323.15	0.0	53.64 ± 0.01	947.08 ± 0.62	962.45	0.86	1.00	-3.79
50	323.15	0.1	54.09 ± 0.01	835.23 ± 0.35	816.42	0.89	1.01	-1.95

Continued on next page

p [MPa]	T [K]	x_{H_2}	L [Å]	ρ / [kg/m^3] ρ^{MD}	ρ^{RFP}	Z Z^{MD}	Z^{RFP}	E_{tot} [kJ/mol]
50	323.15	0.2	54.84 ± 0.01	717.14 ± 0.39	688.40	0.92	1.01	-0.25
50	323.15	0.3	55.83 ± 0.01	599.43 ± 0.11	573.90	0.98	1.02	1.26
50	323.15	0.4	57.00 ± 0.01	487.91 ± 0.18	470.82	1.04	1.02	2.55
50	323.15	0.5	58.14 ± 0.01	388.93 ± 0.10	378.43	1.10	1.03	3.64
50	323.15	0.6	59.16 ± 0.02	301.78 ± 0.24	295.70	1.16	1.03	4.52
50	323.15	0.7	59.99 ± 0.01	224.79 ± 0.12	221.23	1.21	1.03	5.22
50	323.15	0.8	60.65 ± 0.01	155.07 ± 0.01	153.15	1.25	1.03	5.76
50	323.15	0.9	61.05 ± 0.01	90.70 ± 0.02	89.64	1.28	0.90	6.17
50	323.15	1.0	61.23 ± 0.01	29.16 ± 0.01	28.93	1.29	0.91	6.45
50	348.15	0.0	55.07 ± 0.01	875.04 ± 0.58	890.10	0.87	0.92	-2.24
50	348.15	0.1	55.73 ± 0.01	763.72 ± 0.54	752.45	0.90	0.93	-0.49
50	348.15	0.2	56.68 ± 0.01	649.63 ± 0.45	632.63	0.95	0.94	1.06
50	348.15	0.3	57.78 ± 0.01	540.80 ± 0.34	526.68	1.00	0.95	2.43
50	348.15	0.4	58.91 ± 0.02	442.07 ± 0.47	432.59	1.06	0.96	3.58
50	348.15	0.5	59.94 ± 0.01	354.87 ± 0.18	348.90	1.12	0.97	4.52
50	348.15	0.6	60.83 ± 0.01	277.53 ± 0.04	273.97	1.17	0.98	5.28
50	348.15	0.7	61.55 ± 0.02	208.16 ± 0.23	206.04	1.21	0.99	5.90
50	348.15	0.8	62.07 ± 0.01	144.63 ± 0.10	143.33	1.24	0.99	6.38
50	348.15	0.9	62.39 ± 0.01	84.98 ± 0.03	84.24	1.26	1.00	6.75
50	348.15	1.0	62.51 ± 0.01	27.41 ± 0.01	27.27	1.27	1.01	7.00
50	373.15	0.0	56.69 ± 0.01	802.05 ± 0.53	818.76	0.88	1.01	-0.72
50	373.15	0.1	57.54 ± 0.01	694.06 ± 0.53	691.10	0.92	1.02	0.88
50	373.15	0.2	58.57 ± 0.02	588.56 ± 0.67	580.82	0.98	1.02	2.30

Continued on next page

p [MPa]	T [K]	x_{H_2}	L [Å]	ρ / [kg/m^3] ρ^{MD}	ρ^{RFP}	Z Z^{MD}	Z^{RFP}	E_{tot} [kJ/mol]
50	373.15	0.3	59.64 ± 0.02	491.70 ± 0.61	484.28	1.03	1.02	3.50
50	373.15	0.4	60.70 ± 0.02	404.14 ± 0.35	399.12	1.09	1.03	4.50
50	373.15	0.5	61.61 ± 0.02	326.78 ± 0.36	323.42	1.13	1.03	5.33
50	373.15	0.6	62.38 ± 0.01	257.45 ± 0.11	255.27	1.18	1.03	6.00
50	373.15	0.7	63.00 ± 0.01	194.10 ± 0.03	192.92	1.21	1.03	6.55
50	373.15	0.8	63.44 ± 0.01	135.50 ± 0.01	134.76	1.24	0.63	6.98
50	373.15	0.9	63.65 ± 0.01	80.03 ± 0.04	79.47	1.25	0.69	7.31
50	373.15	1.0	63.71 ± 0.01	25.89 ± 0.01	25.79	1.25	0.73	7.54
50	398.15	0.0	58.40 ± 0.02	733.85 ± 0.68	750.33	0.91	0.77	0.69
50	398.15	0.1	59.39 ± 0.01	631.29 ± 0.35	634.01	0.95	0.81	2.16
50	398.15	0.2	60.40 ± 0.01	536.72 ± 0.27	534.06	1.00	0.84	3.41
50	398.15	0.3	61.45 ± 0.01	449.60 ± 0.05	446.96	1.06	0.86	4.47
50	398.15	0.4	62.39 ± 0.02	372.09 ± 0.29	370.08	1.10	0.89	5.36
50	398.15	0.5	63.20 ± 0.01	302.78 ± 0.19	301.40	1.15	0.91	6.09
50	398.15	0.6	63.85 ± 0.01	240.07 ± 0.02	239.06	1.18	0.93	6.69
50	398.15	0.7	64.36 ± 0.01	182.03 ± 0.08	181.45	1.21	0.95	7.18
50	398.15	0.8	64.69 ± 0.01	127.76 ± 0.05	127.22	1.23	0.96	7.57
50	398.15	0.9	64.86 ± 0.01	75.66 ± 0.02	75.24	1.24	0.98	7.87
50	398.15	1.0	64.88 ± 0.02	24.52 ± 0.02	24.47	1.24	0.99	8.09
50	423.15	0.0	60.21 ± 0.01	669.62 ± 0.26	686.95	0.93	1.00	2.02
50	423.15	0.1	61.21 ± 0.01	576.58 ± 0.34	582.58	0.98	1.01	3.32
50	423.15	0.2	62.18 ± 0.03	491.99 ± 0.60	492.86	1.03	1.02	4.43
50	423.15	0.3	63.15 ± 0.03	414.32 ± 0.54	414.48	1.08	1.03	5.37

Continued on next page

p [MPa]	T [K]	x_{H_2}	L [Å]	ρ / [kg/m^3]		Z		E_{tot} [kJ/mol]
				ρ^{MD}	ρ^{RFP}	Z^{MD}	Z^{RFP}	
50	423.15	0.4	63.96 ± 0.01	345.34 ± 0.23	344.94	1.12	1.03	6.15
50	423.15	0.5	64.66 ± 0.01	282.68 ± 0.07	282.32	1.16	1.04	6.80
50	423.15	0.6	65.23 ± 0.02	225.15 ± 0.20	224.93	1.19	1.04	7.35
50	423.15	0.7	65.63 ± 0.02	171.70 ± 0.16	171.36	1.21	0.75	7.79
50	423.15	0.8	65.89 ± 0.01	120.89 ± 0.04	120.52	1.22	0.78	8.15
50	423.15	0.9	66.02 ± 0.02	71.75 ± 0.05	71.46	1.23	0.81	8.43
50	423.15	1.0	65.98 ± 0.02	23.31 ± 0.02	23.29	1.23	0.83	8.63

S10.2 Thermodynamic factors, viscosities, self-diffusion, Maxwell-Stefan and Fick diffusion coefficients

Table S3: Viscosities (η), thermodynamic factors (Γ), self-diffusion coefficients (D^{self}) of CO_2 and H_2, Maxwell-Stefan diffusion coefficients (D^{MS}), and Fick diffusion coefficients obtained from MD simulations of CO_2-H_2 mixtures comprising of 120 molecules are presented as functions of pressure, temperature, and mole fraction of H_2. The viscosity predictions from REFPROP[3] (η^{RFP}) are provided for comparison. The tabulated self-, Maxwell-Stefan (MS), and Fick diffusion coefficients have been corrected for finite-size effects using Eqs. 1, 2, and 4 of the main text, respectively. The average box lengths of the cubic simulation boxes with 120 molecules follow from the densities provided in Table S2.

p	T	x_{H_2}	Γ^{RFP}	η / [μPas]		D^{self} / [10^{-8}m^2/s]		D^{MS}	D^{Fick}
[MPa]	[K]			η^{MD}	η^{RFP}	$D^{\text{self}}_{CO_2}$	$D^{\text{self}}_{H_2}$	[10^{-6}m^2/s]	[10^{-6}m^2/s]
5	323.15	0.0	1.00	16.0 ± 2.8	17.3	21.92	-	-	-
5	323.15	0.1	0.96	16.9 ± 0.6	17.1	25.26	134.96	1.24 ± 0.04	1.20 ± 0.04
5	323.15	0.2	0.93	17.3 ± 1.9	16.8	28.67	145.08	1.26 ± 0.01	1.18 ± 0.01
5	323.15	0.3	0.91	16.8 ± 1.9	16.6	32.74	160.19	1.34 ± 0.03	1.23 ± 0.03
5	323.15	0.4	0.91	15.8 ± 0.2	16.4	37.04	177.09	1.36 ± 0.06	1.23 ± 0.05
5	323.15	0.5	0.90	16.3 ± 0.8	16.1	43.92	194.10	1.37 ± 0.35	1.24 ± 0.32
5	323.15	0.6	0.91	15.7 ± 0.4	15.8	51.30	214.42	1.44 ± 0.05	1.31 ± 0.05
5	323.15	0.7	0.92	14.4 ± 1.0	15.4	61.78	237.97	1.52 ± 0.09	1.40 ± 0.09
5	323.15	0.8	0.94	14.0 ± 1.1	14.6	75.43	264.68	1.41 ± 0.02	1.33 ± 0.02
5	323.15	0.9	0.97	12.2 ± 0.3	13.2	95.20	296.24	1.35 ± 0.03	1.30 ± 0.02
5	323.15	1.0	1.00	9.7 ± 1.1	9.5	-	332.34	-	-
5	348.15	0.0	1.00	17.5 ± 0.4	18.3	26.90	-	-	-
5	348.15	0.1	0.98	17.5 ± 0.5	18.1	30.25	155.38	1.50 ± 0.08	1.47 ± 0.07
5	348.15	0.2	0.96	18.0 ± 1.3	17.8	34.27	170.83	1.51 ± 0.08	1.45 ± 0.08

Continued on next page

p [MPa]	T [K]	x_{H_2}	Γ^{RFP}	η / [μPas] η^{MD}	η^{RFP}	D^{self} / [10^{-8}m^2/s] $D^{self}_{CO_2}$	$D^{self}_{H_2}$	D^{MS} [10^{-6}m^2/s]	D^{Fick} [10^{-6}m^2/s]
5	348.15	0.3	0.94	16.7 ± 0.6	17.6	39.20	185.94	1.46 ± 0.02	1.38 ± 0.02
5	348.15	0.4	0.93	15.8 ± 1.1	17.4	44.70	203.92	1.56 ± 0.07	1.45 ± 0.06
5	348.15	0.5	0.93	17.9 ± 2.0	17.1	51.11	224.04	1.60 ± 0.06	1.49 ± 0.06
5	348.15	0.6	0.93	17.6 ± 1.1	16.8	60.11	247.74	1.64 ± 0.01	1.52 ± 0.01
5	348.15	0.7	0.94	14.7 ± 0.8	16.3	72.09	275.79	1.67 ± 0.11	1.56 ± 0.10
5	348.15	0.8	0.95	14.6 ± 0.5	15.5	88.64	300.38	1.76 ± 0.16	1.67 ± 0.15
5	348.15	0.9	0.97	12.7 ± 0.8	13.9	108.69	335.95	1.67 ± 0.03	1.62 ± 0.03
5	348.15	1.0	1.00	9.7 ± 0.5	10.0	-	370.68	-	-
5	373.15	0.0	1.00	18.2 ± 0.7	19.3	31.52	-	-	-
5	373.15	0.1	0.98	21.2 ± 4.9	19.1	35.45	178.60	1.67 ± 0.01	1.64 ± 0.01
5	373.15	0.2	0.97	18.4 ± 0.6	18.9	40.07	193.15	1.74 ± 0.02	1.69 ± 0.02
5	373.15	0.3	0.96	17.8 ± 0.8	18.7	45.41	216.39	1.87 ± 0.09	1.79 ± 0.09
5	373.15	0.4	0.95	17.6 ± 0.4	18.4	50.72	232.03	1.86 ± 0.07	1.76 ± 0.07
5	373.15	0.5	0.94	17.8 ± 0.2	18.1	58.00	253.51	1.82 ± 0.01	1.72 ± 0.01
5	373.15	0.6	0.94	17.4 ± 0.7	17.8	67.84	280.37	1.84 ± 0.08	1.74 ± 0.08
5	373.15	0.7	0.95	16.4 ± 1.1	17.2	79.83	307.12	2.06 ± 0.34	1.95 ± 0.32
5	373.15	0.8	0.96	15.6 ± 1.3	16.3	98.54	343.77	1.96 ± 0.27	1.88 ± 0.25
5	373.15	0.9	0.98	13.2 ± 1.3	14.6	127.45	382.58	1.69 ± 0.09	1.65 ± 0.09
5	373.15	1.0	1.00	10.2 ± 0.7	10.5	-	423.91	-	-
5	398.15	0.0	1.00	20.0 ± 0.7	20.3	37.18	-	-	-
5	398.15	0.1	0.99	20.4 ± 1.2	20.1	42.28	202.56	1.89 ± 0.06	1.87 ± 0.06
5	398.15	0.2	0.98	20.9 ± 1.2	19.9	45.42	228.84	1.96 ± 0.05	1.92 ± 0.05
5	398.15	0.3	0.97	19.2 ± 1.0	19.7	52.07	242.25	2.09 ± 0.10	2.03 ± 0.09

Continued on next page

p [MPa]	T [K]	x_{H_2}	Γ^{RFP}	η / [μPas] η^{MD}	η^{RFP}	D^{self} / [10^{-8}m^2/s] $D^{self}_{CO_2}$	$D^{self}_{H_2}$	D^{MS} [10^{-6}m^2/s]	D^{Fick} [10^{-6}m^2/s]
5	398.15	0.4	0.96	16.9 ± 3.2	19.5	58.77	263.13	1.93 ± 0.06	1.86 ± 0.06
5	398.15	0.5	0.95	17.9 ± 0.8	19.1	67.64	283.38	2.01 ± 0.06	1.92 ± 0.06
5	398.15	0.6	0.95	17.1 ± 1.5	18.7	78.31	312.91	2.12 ± 0.07	2.02 ± 0.06
5	398.15	0.7	0.96	18.3 ± 1.5	18.1	90.45	345.28	2.10 ± 0.07	2.01 ± 0.07
5	398.15	0.8	0.96	16.8 ± 1.2	17.1	108.75	372.84	2.04 ± 0.05	1.96 ± 0.04
5	398.15	0.9	0.98	14.8 ± 1.1	15.3	139.93	426.12	2.22 ± 0.06	2.17 ± 0.06
5	398.15	1.0	1.00	10.7 ± 1.0	10.9	-	471.04	-	-
5	423.15	0.0	1.00	20.1 ± 1.1	21.3	42.27	-	-	-
5	423.15	0.1	0.99	20.6 ± 0.9	21.1	46.67	224.49	2.16 ± 0.03	2.15 ± 0.03
5	423.15	0.2	0.98	17.5 ± 6.2	21.0	52.27	249.24	2.17 ± 0.01	2.14 ± 0.01
5	423.15	0.3	0.98	19.6 ± 0.4	20.7	59.24	272.64	2.33 ± 0.05	2.27 ± 0.05
5	423.15	0.4	0.97	21.3 ± 2.0	20.5	67.23	289.44	2.32 ± 0.12	2.25 ± 0.11
5	423.15	0.5	0.96	20.0 ± 1.4	20.1	75.59	314.57	2.27 ± 0.13	2.19 ± 0.13
5	423.15	0.6	0.96	18.5 ± 0.9	19.7	85.74	352.51	2.28 ± 0.03	2.19 ± 0.03
5	423.15	0.7	0.96	17.9 ± 1.5	19.0	101.81	376.94	2.36 ± 0.21	2.27 ± 0.21
5	423.15	0.8	0.97	17.0 ± 1.2	17.9	123.77	420.83	2.22 ± 0.04	2.15 ± 0.04
5	423.15	0.9	0.98	14.7 ± 2.1	16.0	153.43	480.22	2.36 ± 0.11	2.31 ± 0.11
5	423.15	1.0	1.00	10.8 ± 0.8	11.4	-	520.37	-	-
10	323.15	0.0	1.00	25.5 ± 0.9	28.4	6.20	-	-	-
10	323.15	0.1	0.85	19.7 ± 0.2	21.2	10.22	46.91	0.45 ± 0.01	0.39 ± 0.01
10	323.15	0.2	0.77	18.1 ± 0.6	19.3	12.87	59.74	0.55 ± 0.01	0.42 ± 0.01
10	323.15	0.3	0.72	17.3 ± 0.2	18.3	15.44	70.49	0.64 ± 0.06	0.47 ± 0.04
10	323.15	0.4	0.72	17.0 ± 0.6	17.6	18.39	82.12	0.64 ± 0.01	0.47 ± 0.01

Continued on next page

p	T	x_{H_2}	Γ^{RFP}	η / [μPas]		D^{self} / [$10^{-8}m^2/s$]		D^{MS}	D^{Fick}
[MPa]	[K]			η^{MD}	η^{RFP}	$D^{self}_{CO_2}$	$D^{self}_{H_2}$	[$10^{-6}m^2/s$]	[$10^{-6}m^2/s$]
10	323.15	0.5	0.75	16.2 ± 0.4	17.1	21.64	91.59	0.70 ± 0.02	0.52 ± 0.01
10	323.15	0.6	0.80	15.7 ± 0.7	16.5	25.94	103.77	0.74 ± 0.03	0.59 ± 0.02
10	323.15	0.7	0.85	16.8 ± 2.1	15.9	31.25	116.98	0.79 ± 0.05	0.67 ± 0.05
10	323.15	0.8	0.91	13.3 ± 0.8	15.0	38.66	131.79	0.72 ± 0.01	0.66 ± 0.01
10	323.15	0.9	0.97	12.3 ± 0.5	13.4	50.20	149.38	0.75 ± 0.02	0.72 ± 0.02
10	323.15	1.0	1.00	10.2 ± 1.8	9.6	-	166.46	-	-
10	348.15	0.0	1.00	21.0 ± 0.4	22.2	10.90	-	-	-
10	348.15	0.1	0.93	19.5 ± 0.5	20.6	13.48	63.53	0.60 ± 0.01	0.56 ± 0.01
10	348.15	0.2	0.88	18.8 ± 0.4	19.7	15.93	73.46	0.69 ± 0.01	0.61 ± 0.01
10	348.15	0.3	0.85	19.6 ± 1.8	19.0	18.75	85.18	0.73 ± 0.02	0.62 ± 0.02
10	348.15	0.4	0.83	18.0 ± 0.7	18.5	21.85	95.53	0.75 ± 0.02	0.63 ± 0.01
10	348.15	0.5	0.83	17.6 ± 1.4	18.0	25.42	107.30	0.77 ± 0.05	0.64 ± 0.04
10	348.15	0.6	0.85	16.4 ± 0.3	17.4	29.96	118.14	0.83 ± 0.02	0.70 ± 0.02
10	348.15	0.7	0.88	15.8 ± 0.6	16.7	35.99	133.49	0.84 ± 0.02	0.74 ± 0.02
10	348.15	0.8	0.91	14.4 ± 0.3	15.8	43.64	150.66	0.89 ± 0.06	0.81 ± 0.05
10	348.15	0.9	0.95	13.4 ± 1.0	14.1	55.04	170.38	0.87 ± 0.03	0.83 ± 0.03
10	348.15	1.0	1.00	9.8 ± 0.3	10.1	-	189.67	-	-
10	373.15	0.0	1.00	21.6 ± 0.7	21.8	14.07	-	-	-
10	373.15	0.1	0.96	20.9 ± 2.2	21.0	16.65	77.96	0.73 ± 0.01	0.70 ± 0.01
10	373.15	0.2	0.92	21.2 ± 2.6	20.3	19.27	88.73	0.81 ± 0.02	0.74 ± 0.02
10	373.15	0.3	0.90	19.7 ± 1.5	19.8	22.35	98.83	0.84 ± 0.01	0.75 ± 0.01
10	373.15	0.4	0.88	18.9 ± 1.1	19.3	25.76	111.48	0.86 ± 0.04	0.76 ± 0.04
10	373.15	0.5	0.88	18.1 ± 0.9	18.9	29.55	122.08	0.93 ± 0.08	0.82 ± 0.07

Continued on next page

p [MPa]	T [K]	x_{H_2}	Γ^{RFP}	η / [μPas] η^{MD}	η^{RFP}	D^{self} / [10^{-8}m^2/s] $D^{self}_{CO_2}$	$D^{self}_{H_2}$	D^{MS} [10^{-6}m^2/s]	D^{Fick} [10^{-6}m^2/s]
10	373.15	0.6	0.88	18.1 ± 0.8	18.3	34.05	136.30	0.90 ± 0.02	0.79 ± 0.02
10	373.15	0.7	0.90	18.6 ± 2.7	17.6	41.78	151.76	0.93 ± 0.05	0.83 ± 0.04
10	373.15	0.8	0.92	15.6 ± 0.9	16.6	50.72	169.05	1.02 ± 0.06	0.94 ± 0.06
10	373.15	0.9	0.96	14.8 ± 2.1	14.8	63.15	192.91	0.95 ± 0.04	0.91 ± 0.04
10	373.15	1.0	1.00	9.9 ± 0.3	10.6	-	212.76	-	-
10	398.15	0.0	1.00	21.0 ± 1.8	22.2	17.24	-	-	-
10	398.15	0.1	0.97	21.2 ± 1.9	21.6	19.68	91.91	0.88 ± 0.01	0.85 ± 0.01
10	398.15	0.2	0.95	22.0 ± 1.8	21.1	22.25	101.59	0.89 ± 0.01	0.84 ± 0.01
10	398.15	0.3	0.93	20.3 ± 1.0	20.7	25.34	113.95	1.02 ± 0.06	0.95 ± 0.05
10	398.15	0.4	0.91	19.7 ± 0.7	20.2	28.83	125.96	1.01 ± 0.02	0.92 ± 0.02
10	398.15	0.5	0.90	19.6 ± 1.4	19.8	33.32	137.76	1.03 ± 0.05	0.93 ± 0.04
10	398.15	0.6	0.90	18.5 ± 0.9	19.2	39.29	154.84	1.04 ± 0.03	0.94 ± 0.02
10	398.15	0.7	0.91	17.2 ± 0.3	18.5	45.86	169.54	1.12 ± 0.01	1.02 ± 0.01
10	398.15	0.8	0.93	15.9 ± 0.3	17.4	57.28	189.93	1.09 ± 0.02	1.02 ± 0.02
10	398.15	0.9	0.96	14.0 ± 0.6	15.4	69.30	212.05	1.09 ± 0.04	1.04 ± 0.04
10	398.15	1.0	1.00	10.3 ± 0.2	11.0	-	236.14	-	-
10	423.15	0.0	1.00	23.3 ± 1.2	22.8	19.99	-	-	-
10	423.15	0.1	0.98	22.4 ± 0.6	22.4	22.90	104.34	1.00 ± 0.03	0.98 ± 0.03
10	423.15	0.2	0.96	21.0 ± 0.2	22.0	25.57	116.03	1.06 ± 0.03	1.02 ± 0.03
10	423.15	0.3	0.94	20.8 ± 0.7	21.6	28.91	129.73	1.11 ± 0.04	1.05 ± 0.04
10	423.15	0.4	0.93	20.3 ± 0.4	21.2	33.17	141.51	1.12 ± 0.06	1.04 ± 0.06
10	423.15	0.5	0.92	20.5 ± 0.9	20.7	37.63	157.07	1.16 ± 0.03	1.07 ± 0.03
10	423.15	0.6	0.92	19.9 ± 1.3	20.1	43.09	173.19	1.12 ± 0.09	1.03 ± 0.08

Continued on next page

p [MPa]	T [K]	x_{H_2}	Γ^{RFP}	η / [μPas] η^{MD}	η^{RFP}	D^{self} / [10^{-8}m^2/s] $D^{self}_{CO_2}$	$D^{self}_{H_2}$	D^{MS} [10^{-6}m^2/s]	D^{Fick} [10^{-6}m^2/s]
10	423.15	0.7	0.92	18.4 ± 1.1	19.3	53.16	191.77	1.17 ± 0.01	1.08 ± 0.01
10	423.15	0.8	0.94	17.2 ± 1.1	18.2	63.55	208.51	1.16 ± 0.01	1.09 ± 0.01
10	423.15	0.9	0.96	16.1 ± 1.6	16.1	78.48	236.12	1.18 ± 0.02	1.13 ± 0.02
10	423.15	1.0	1.00	11.2 ± 0.5	11.5	-	259.70	-	-
15	323.15	0.0	1.00	57.7 ± 0.7	56.5	2.68	-	-	-
15	323.15	0.1	0.71	32.0 ± 3.1	33.4	5.07	18.91	0.18 ± 0.01	0.13 ± 0.01
15	323.15	0.2	0.55	23.1 ± 1.0	24.7	7.60	31.20	0.29 ± 0.01	0.16 ± 0.01
15	323.15	0.3	0.48	19.3 ± 0.8	21.3	9.81	41.07	0.37 ± 0.01	0.18 ± 0.01
15	323.15	0.4	0.50	18.1 ± 0.5	19.5	12.03	49.70	0.42 ± 0.01	0.21 ± 0.01
15	323.15	0.5	0.57	17.4 ± 0.7	18.3	14.67	58.63	0.45 ± 0.01	0.26 ± 0.01
15	323.15	0.6	0.67	17.4 ± 2.3	17.3	17.51	67.05	0.48 ± 0.01	0.32 ± 0.01
15	323.15	0.7	0.79	15.6 ± 0.8	16.4	21.46	77.06	0.51 ± 0.01	0.40 ± 0.01
15	323.15	0.8	0.90	14.2 ± 0.4	15.3	26.41	88.59	0.52 ± 0.01	0.47 ± 0.01
15	323.15	0.9	0.97	12.3 ± 0.3	13.6	34.40	99.04	0.53 ± 0.02	0.52 ± 0.02
15	323.15	1.0	1.00	9.4 ± 0.4	9.8	-	113.71	-	-
15	348.15	0.0	1.00	32.0 ± 0.5	34.8	5.26	-	-	-
15	348.15	0.1	0.85	25.3 ± 2.9	26.4	7.79	32.60	0.31 ± 0.01	0.26 ± 0.01
15	348.15	0.2	0.76	20.4 ± 1.0	23.0	9.97	42.09	0.39 ± 0.01	0.29 ± 0.01
15	348.15	0.3	0.71	20.0 ± 1.1	21.1	12.09	51.43	0.44 ± 0.01	0.32 ± 0.01
15	348.15	0.4	0.70	19.6 ± 1.1	19.9	14.69	60.78	0.50 ± 0.02	0.35 ± 0.01
15	348.15	0.5	0.72	18.5 ± 1.0	19.0	17.22	69.48	0.54 ± 0.03	0.39 ± 0.02
15	348.15	0.6	0.76	17.3 ± 0.5	18.1	20.15	78.53	0.55 ± 0.03	0.42 ± 0.02
15	348.15	0.7	0.82	16.9 ± 0.5	17.2	24.76	88.65	0.58 ± 0.01	0.47 ± 0.01

Continued on next page

p [MPa]	T [K]	x_{H_2}	Γ^{RFP}	η / [μPas] η^{MD}	η^{RFP}	D^{self} / [10^{-8}m^2/s] $D^{self}_{CO_2}$	$D^{self}_{H_2}$	D^{MS} [10^{-6}m^2/s]	D^{Fick} [10^{-6}m^2/s]
15	348.15	0.8	0.88	15.0 ± 0.8	16.1	30.22	100.80	0.59 ± 0.01	0.52 ± 0.01
15	348.15	0.9	0.95	13.4 ± 0.9	14.3	40.47	113.52	0.59 ± 0.01	0.56 ± 0.01
15	348.15	1.0	1.00	10.4 ± 0.9	10.2	-	127.84	-	-
15	373.15	0.0	1.00	26.4 ± 1.6	27.8	8.14	-	-	-
15	373.15	0.1	0.92	23.1 ± 0.6	24.6	10.19	43.44	0.43 ± 0.02	0.39 ± 0.01
15	373.15	0.2	0.86	24.4 ± 5.9	22.7	12.31	52.53	0.48 ± 0.01	0.41 ± 0.01
15	373.15	0.3	0.82	21.3 ± 1.1	21.5	14.41	61.57	0.53 ± 0.01	0.43 ± 0.01
15	373.15	0.4	0.80	19.3 ± 0.5	20.5	17.01	69.69	0.57 ± 0.01	0.46 ± 0.01
15	373.15	0.5	0.80	19.1 ± 0.4	19.7	19.73	79.58	0.63 ± 0.04	0.50 ± 0.03
15	373.15	0.6	0.82	18.5 ± 0.4	18.9	23.35	89.33	0.62 ± 0.01	0.51 ± 0.01
15	373.15	0.7	0.85	17.3 ± 0.7	18.0	28.13	100.31	0.64 ± 0.01	0.54 ± 0.01
15	373.15	0.8	0.89	15.8 ± 0.5	16.9	34.55	113.37	0.70 ± 0.02	0.63 ± 0.02
15	373.15	0.9	0.94	13.6 ± 0.4	14.9	43.93	128.79	0.70 ± 0.04	0.66 ± 0.03
15	373.15	1.0	1.00	11.1 ± 1.0	10.7	-	143.91	-	-
15	398.15	0.0	1.00	24.9 ± 0.4	26.0	10.46	-	-	-
15	398.15	0.1	0.95	23.2 ± 1.0	24.2	12.47	54.41	0.52 ± 0.01	0.50 ± 0.01
15	398.15	0.2	0.91	23.6 ± 1.6	23.0	14.56	62.72	0.59 ± 0.03	0.54 ± 0.02
15	398.15	0.3	0.87	22.1 ± 0.7	22.1	16.96	71.49	0.62 ± 0.01	0.54 ± 0.01
15	398.15	0.4	0.86	21.6 ± 1.2	21.3	19.59	80.36	0.66 ± 0.01	0.56 ± 0.01
15	398.15	0.5	0.85	19.6 ± 0.7	20.5	22.75	90.30	0.67 ± 0.01	0.57 ± 0.01
15	398.15	0.6	0.85	18.9 ± 1.0	19.8	27.22	100.31	0.70 ± 0.02	0.60 ± 0.02
15	398.15	0.7	0.87	17.9 ± 0.6	18.9	31.69	112.90	0.71 ± 0.01	0.62 ± 0.01
15	398.15	0.8	0.90	16.5 ± 0.8	17.6	39.02	126.34	0.74 ± 0.01	0.67 ± 0.01

Continued on next page

p [MPa]	T [K]	x_{H_2}	Γ^{RFP}	η / [μPas] η^{MD}	η^{RFP}	D^{self} / [10^{-8}m^2/s] $D^{self}_{CO_2}$	$D^{self}_{H_2}$	D^{MS} [10^{-6}m^2/s]	D^{Fick} [10^{-6}m^2/s]
15	398.15	0.9	0.94	14.4 ± 0.3	15.6	49.63	144.12	0.73 ± 0.04	0.69 ± 0.03
15	398.15	1.0	1.00	11.5 ± 1.0	11.1	-	158.95	-	-
15	423.15	0.0	1.00	24.5 ± 0.9	25.6	12.65	-	-	-
15	423.15	0.1	0.96	23.7 ± 0.7	24.4	14.68	64.13	0.61 ± 0.02	0.59 ± 0.02
15	423.15	0.2	0.93	22.7 ± 0.9	23.5	16.80	72.71	0.67 ± 0.02	0.62 ± 0.02
15	423.15	0.3	0.91	21.5 ± 0.4	22.7	19.19	82.02	0.68 ± 0.01	0.62 ± 0.01
15	423.15	0.4	0.89	22.0 ± 0.7	22.1	21.99	91.29	0.71 ± 0.01	0.63 ± 0.01
15	423.15	0.5	0.88	20.7 ± 0.6	21.4	25.26	102.28	0.74 ± 0.02	0.65 ± 0.02
15	423.15	0.6	0.88	19.7 ± 0.6	20.6	29.44	113.81	0.78 ± 0.02	0.68 ± 0.02
15	423.15	0.7	0.89	19.4 ± 0.9	19.7	35.44	126.36	0.92 ± 0.11	0.82 ± 0.10
15	423.15	0.8	0.91	16.9 ± 0.6	18.4	44.01	141.19	0.79 ± 0.04	0.72 ± 0.04
15	423.15	0.9	0.95	14.7 ± 0.7	16.2	54.42	159.56	0.85 ± 0.01	0.81 ± 0.01
15	423.15	1.0	1.00	11.3 ± 0.6	11.6	-	182.40	-	-
20	323.15	0.0	1.00	68.8 ± 0.8	68.7	2.24	-	-	-
20	323.15	0.1	0.68	42.7 ± 1.3	44.9	3.49	11.32	0.11 ± 0.01	0.08 ± 0.01
20	323.15	0.2	0.49	29.7 ± 2.5	31.7	5.24	19.05	0.19 ± 0.01	0.09 ± 0.01
20	323.15	0.3	0.40	23.0 ± 0.8	25.2	7.07	27.48	0.28 ± 0.03	0.11 ± 0.01
20	323.15	0.4	0.40	20.3 ± 0.6	21.8	8.90	34.91	0.31 ± 0.01	0.12 ± 0.01
20	323.15	0.5	0.47	18.2 ± 0.3	19.8	11.06	41.95	0.34 ± 0.01	0.16 ± 0.01
20	323.15	0.6	0.57	17.1 ± 0.5	18.3	13.20	49.60	0.36 ± 0.01	0.21 ± 0.01
20	323.15	0.7	0.70	16.0 ± 0.6	17.0	16.19	57.26	0.38 ± 0.02	0.27 ± 0.01
20	323.15	0.8	0.83	14.6 ± 0.5	15.7	20.84	65.94	0.40 ± 0.01	0.33 ± 0.01
20	323.15	0.9	0.94	13.1 ± 0.5	13.8	28.53	76.47	0.41 ± 0.01	0.39 ± 0.01

Continued on next page

p [MPa]	T [K]	x_{H_2}	Γ^{RFP}	η / [μPas] η^{MD}	η^{RFP}	D^{self} / [10^{-8}m^2/s] $D^{self}_{CO_2}$	$D^{self}_{H_2}$	D^{MS} [10^{-6}m^2/s]	D^{Fick} [10^{-6}m^2/s]
20	323.15	1.0	1.00	9.5 ± 0.2	9.9	-	86.03	-	-
20	348.15	0.0	1.00	46.6 ± 0.4	49.1	3.52	-	-	-
20	348.15	0.1	0.80	32.1 ± 1.1	34.7	5.26	19.18	0.19 ± 0.01	0.15 ± 0.01
20	348.15	0.2	0.67	25.4 ± 0.4	27.7	7.13	27.65	0.26 ± 0.01	0.18 ± 0.01
20	348.15	0.3	0.61	22.4 ± 0.2	24.0	9.02	35.06	0.32 ± 0.01	0.19 ± 0.01
20	348.15	0.4	0.60	19.8 ± 0.9	21.7	10.82	42.94	0.35 ± 0.01	0.21 ± 0.01
20	348.15	0.5	0.63	19.2 ± 1.1	20.2	13.11	50.32	0.39 ± 0.01	0.25 ± 0.01
20	348.15	0.6	0.68	19.1 ± 2.0	18.9	15.63	57.04	0.43 ± 0.02	0.30 ± 0.01
20	348.15	0.7	0.76	17.7 ± 1.3	17.8	18.84	65.99	0.44 ± 0.01	0.33 ± 0.01
20	348.15	0.8	0.85	15.2 ± 0.4	16.4	24.11	75.04	0.45 ± 0.01	0.38 ± 0.01
20	348.15	0.9	0.93	13.5 ± 0.3	14.5	30.44	86.87	0.53 ± 0.08	0.49 ± 0.07
20	348.15	1.0	1.00	9.6 ± 0.7	10.3	-	97.03	-	-
20	373.15	0.0	1.00	35.1 ± 0.3	37.2	5.34	-	-	-
20	373.15	0.1	0.88	27.5 ± 0.3	30.0	7.17	27.38	0.27 ± 0.01	0.23 ± 0.01
20	373.15	0.2	0.79	24.6 ± 0.7	26.1	8.96	35.23	0.34 ± 0.01	0.27 ± 0.01
20	373.15	0.3	0.74	22.4 ± 0.5	23.7	10.82	43.27	0.38 ± 0.01	0.28 ± 0.01
20	373.15	0.4	0.72	20.9 ± 0.6	22.0	12.84	50.12	0.41 ± 0.01	0.30 ± 0.01
20	373.15	0.5	0.73	19.7 ± 0.6	20.8	15.24	57.56	0.45 ± 0.02	0.33 ± 0.01
20	373.15	0.6	0.76	19.0 ± 0.4	19.6	18.10	66.62	0.52 ± 0.06	0.40 ± 0.05
20	373.15	0.7	0.80	18.0 ± 1.1	18.5	21.74	75.40	0.51 ± 0.02	0.41 ± 0.01
20	373.15	0.8	0.86	16.0 ± 0.7	17.1	27.71	85.63	0.50 ± 0.01	0.43 ± 0.01
20	373.15	0.9	0.93	13.9 ± 1.2	15.1	34.69	97.16	0.51 ± 0.02	0.48 ± 0.02
20	373.15	1.0	1.00	10.7 ± 0.3	10.8	-	108.10	-	-

Continued on next page

p	T	x_{H_2}	Γ^{RFP}	η / [μPas]		D^{self} / [10^{-8}m^2/s]		D^{MS}	D^{Fick}
[MPa]	[K]			η^{MD}	η^{RFP}	$D^{self}_{CO_2}$	$D^{self}_{H_2}$	[10^{-6}m^2/s]	[10^{-6}m^2/s]
20	398.15	0.0	1.00	30.2 ± 1.0	31.9	7.29	-	-	-
20	398.15	0.1	0.92	26.0 ± 0.6	28.0	8.96	35.89	0.34 ± 0.01	0.32 ± 0.01
20	398.15	0.2	0.86	25.0 ± 1.9	25.5	10.76	44.05	0.40 ± 0.01	0.34 ± 0.01
20	398.15	0.3	0.82	23.0 ± 0.5	23.8	12.58	50.97	0.45 ± 0.02	0.37 ± 0.01
20	398.15	0.4	0.80	21.6 ± 1.4	22.5	15.00	58.52	0.48 ± 0.03	0.38 ± 0.02
20	398.15	0.5	0.79	20.2 ± 0.8	21.4	17.21	66.72	0.51 ± 0.02	0.40 ± 0.02
20	398.15	0.6	0.81	19.5 ± 0.8	20.4	20.48	74.56	0.53 ± 0.01	0.42 ± 0.01
20	398.15	0.7	0.83	18.9 ± 0.8	19.3	24.68	83.98	0.56 ± 0.03	0.47 ± 0.03
20	398.15	0.8	0.87	17.1 ± 0.8	17.9	30.22	95.10	0.58 ± 0.02	0.51 ± 0.02
20	398.15	0.9	0.93	15.2 ± 1.3	15.8	37.95	107.94	0.56 ± 0.01	0.52 ± 0.01
20	398.15	1.0	1.00	10.9 ± 0.5	11.2	-	121.09	-	-
20	423.15	0.0	1.00	29.1 ± 1.3	29.8	9.05	-	-	-
20	423.15	0.1	0.95	26.7 ± 0.6	27.3	10.83	44.04	0.42 ± 0.01	0.40 ± 0.01
20	423.15	0.2	0.90	24.3 ± 0.2	25.6	12.50	51.34	0.46 ± 0.02	0.41 ± 0.02
20	423.15	0.3	0.87	23.7 ± 1.5	24.2	14.41	59.56	0.52 ± 0.02	0.45 ± 0.02
20	423.15	0.4	0.85	22.3 ± 1.3	23.1	16.92	66.57	0.54 ± 0.01	0.46 ± 0.01
20	423.15	0.5	0.84	21.3 ± 1.1	22.1	19.47	74.77	0.57 ± 0.02	0.48 ± 0.01
20	423.15	0.6	0.84	20.2 ± 0.6	21.2	22.87	83.57	0.59 ± 0.02	0.50 ± 0.02
20	423.15	0.7	0.86	19.0 ± 0.7	20.1	27.35	94.49	0.60 ± 0.01	0.52 ± 0.01
20	423.15	0.8	0.89	17.5 ± 0.9	18.6	35.38	105.52	0.63 ± 0.03	0.56 ± 0.03
20	423.15	0.9	0.94	14.9 ± 0.5	16.4	42.40	120.56	0.62 ± 0.01	0.58 ± 0.01
20	423.15	1.0	1.00	11.1 ± 0.3	11.7	-	134.35	-	-
25	323.15	0.0	1.00	78.4 ± 1.3	77.4	2.01	-	-	-

Continued on next page

p [MPa]	T [K]	x_{H_2}	Γ^{RFP}	η / [μPas] η^{MD}	η^{RFP}	D^{self} / [10^{-8}m^2/s] $D^{self}_{CO_2}$	$D^{self}_{H_2}$	D^{MS} [10^{-6}m^2/s]	D^{Fick} [10^{-6}m^2/s]
25	323.15	0.1	0.68	52.1 ± 0.7	53.0	2.89	8.56	0.09 ± 0.01	0.06 ± 0.01
25	323.15	0.2	0.48	35.5 ± 0.5	38.0	4.12	13.72	0.14 ± 0.01	0.07 ± 0.01
25	323.15	0.3	0.38	26.8 ± 0.5	29.3	5.63	20.01	0.19 ± 0.01	0.07 ± 0.01
25	323.15	0.4	0.37	22.3 ± 0.2	24.5	7.18	26.00	0.24 ± 0.01	0.09 ± 0.01
25	323.15	0.5	0.41	19.8 ± 0.6	21.4	8.97	31.94	0.28 ± 0.01	0.12 ± 0.01
25	323.15	0.6	0.51	17.7 ± 0.5	19.3	10.95	38.84	0.30 ± 0.01	0.15 ± 0.01
25	323.15	0.7	0.63	16.8 ± 0.5	17.7	13.58	45.46	0.33 ± 0.02	0.21 ± 0.01
25	323.15	0.8	0.77	15.7 ± 0.7	16.1	16.83	52.82	0.35 ± 0.04	0.27 ± 0.03
25	323.15	0.9	0.89	12.9 ± 0.2	14.1	22.13	61.21	0.35 ± 0.01	0.31 ± 0.01
25	323.15	1.0	1.00	9.8 ± 0.4	10.0	-	71.11	-	-
25	348.15	0.0	1.00	57.4 ± 0.8	59.0	2.93	-	-	-
25	348.15	0.1	0.78	39.9 ± 0.3	42.4	4.13	13.48	0.14 ± 0.01	0.11 ± 0.01
25	348.15	0.2	0.63	29.6 ± 1.1	32.7	5.58	19.74	0.19 ± 0.01	0.12 ± 0.01
25	348.15	0.3	0.56	25.1 ± 0.4	27.2	7.09	25.90	0.24 ± 0.01	0.13 ± 0.01
25	348.15	0.4	0.54	21.8 ± 0.7	23.8	8.72	32.35	0.28 ± 0.01	0.15 ± 0.01
25	348.15	0.5	0.56	20.3 ± 0.5	21.6	10.58	38.50	0.32 ± 0.02	0.18 ± 0.01
25	348.15	0.6	0.62	20.1 ± 4.1	19.8	13.09	45.77	0.36 ± 0.02	0.23 ± 0.01
25	348.15	0.7	0.71	17.4 ± 0.8	18.3	15.88	52.84	0.37 ± 0.01	0.26 ± 0.01
25	348.15	0.8	0.81	15.6 ± 0.5	16.8	19.56	60.85	0.37 ± 0.01	0.30 ± 0.01
25	348.15	0.9	0.91	13.4 ± 0.4	14.7	24.51	70.52	0.39 ± 0.01	0.35 ± 0.01
25	348.15	1.0	1.00	10.2 ± 0.3	10.5	-	78.70	-	-
25	373.15	0.0	1.00	43.5 ± 0.5	46.4	4.12	-	-	-
25	373.15	0.1	0.85	33.0 ± 0.5	36.0	5.55	19.54	0.19 ± 0.01	0.16 ± 0.01

Continued on next page

p	T	x_{H_2}	Γ^{RFP}	η / [μPas]		D^{self} / [10^{-8}m^2/s]		D^{MS}	D^{Fick}
[MPa]	[K]			η^{MD}	η^{RFP}	$D^{self}_{CO_2}$	$D^{self}_{H_2}$	[10^{-6}m^2/s]	[10^{-6}m^2/s]
25	373.15	0.2	0.75	28.0 ± 0.7	30.0	7.03	26.07	0.24 ± 0.01	0.18 ± 0.01
25	373.15	0.3	0.69	24.6 ± 0.9	26.2	8.61	32.32	0.29 ± 0.01	0.20 ± 0.01
25	373.15	0.4	0.66	22.9 ± 1.2	23.7	10.32	38.56	0.33 ± 0.01	0.22 ± 0.01
25	373.15	0.5	0.67	21.3 ± 0.8	21.9	12.41	45.52	0.36 ± 0.01	0.24 ± 0.01
25	373.15	0.6	0.70	21.1 ± 1.4	20.4	14.91	52.24	0.39 ± 0.01	0.27 ± 0.01
25	373.15	0.7	0.76	18.2 ± 0.6	19.0	17.85	59.94	0.40 ± 0.01	0.30 ± 0.01
25	373.15	0.8	0.83	16.1 ± 0.3	17.5	21.71	68.65	0.44 ± 0.02	0.37 ± 0.02
25	373.15	0.9	0.91	14.5 ± 0.7	15.3	28.27	78.06	0.42 ± 0.01	0.38 ± 0.01
25	373.15	1.0	1.00	10.3 ± 0.2	10.9	-	88.49	-	-
25	398.15	0.0	1.00	41.3 ± 11.0	38.9	5.54	-	-	-
25	398.15	0.1	0.90	30.2 ± 0.5	32.5	6.97	26.03	0.25 ± 0.01	0.23 ± 0.01
25	398.15	0.2	0.83	26.6 ± 0.8	28.6	8.57	32.51	0.30 ± 0.01	0.25 ± 0.01
25	398.15	0.3	0.78	24.8 ± 0.4	25.9	10.19	38.44	0.35 ± 0.01	0.27 ± 0.01
25	398.15	0.4	0.75	22.8 ± 1.2	23.9	12.00	44.97	0.38 ± 0.01	0.29 ± 0.01
25	398.15	0.5	0.75	20.8 ± 0.3	22.4	14.05	51.73	0.40 ± 0.01	0.30 ± 0.01
25	398.15	0.6	0.76	20.1 ± 0.4	21.1	16.56	59.48	0.42 ± 0.01	0.32 ± 0.01
25	398.15	0.7	0.80	18.3 ± 0.8	19.7	20.05	66.87	0.44 ± 0.02	0.35 ± 0.01
25	398.15	0.8	0.85	16.8 ± 0.5	18.2	24.67	76.79	0.48 ± 0.01	0.41 ± 0.01
25	398.15	0.9	0.92	14.3 ± 0.3	15.9	31.13	87.44	0.50 ± 0.06	0.46 ± 0.05
25	398.15	1.0	1.00	11.1 ± 0.3	11.3	-	98.55	-	-
25	423.15	0.0	1.00	32.5 ± 0.3	34.9	7.02	-	-	-
25	423.15	0.1	0.93	29.2 ± 0.9	30.8	8.52	32.25	0.31 ± 0.01	0.29 ± 0.01
25	423.15	0.2	0.88	26.0 ± 0.5	28.0	10.06	39.12	0.36 ± 0.01	0.31 ± 0.01

Continued on next page

p [MPa]	T [K]	x_{H_2}	Γ^{RFP}	η / [μPas] η^{MD}	η^{RFP}	D^{self} / [10^{-8}m^2/s] $D^{self}_{CO_2}$	$D^{self}_{H_2}$	D^{MS} [10^{-6}m^2/s]	D^{Fick} [10^{-6}m^2/s]
25	423.15	0.3	0.84	25.6 ± 2.1	25.9	11.74	45.50	0.39 ± 0.01	0.33 ± 0.01
25	423.15	0.4	0.81	23.3 ± 0.6	24.3	13.62	52.22	0.42 ± 0.01	0.34 ± 0.01
25	423.15	0.5	0.80	22.2 ± 0.6	23.0	15.84	59.06	0.47 ± 0.01	0.37 ± 0.01
25	423.15	0.6	0.80	20.6 ± 0.2	21.8	18.77	66.90	0.49 ± 0.01	0.39 ± 0.01
25	423.15	0.7	0.83	19.3 ± 0.7	20.5	22.24	75.31	0.49 ± 0.01	0.41 ± 0.01
25	423.15	0.8	0.87	17.7 ± 0.6	18.9	27.38	84.76	0.52 ± 0.03	0.45 ± 0.02
25	423.15	0.9	0.92	15.0 ± 0.8	16.5	34.73	96.83	0.52 ± 0.01	0.48 ± 0.01
25	423.15	1.0	1.00	11.3 ± 0.5	11.8	-	107.97	-	-
30	323.15	0.0	1.00	86.4 ± 0.7	84.8	1.86	-	-	-
30	323.15	0.1	0.69	60.5 ± 0.2	59.3	2.55	7.27	0.07 ± 0.01	0.05 ± 0.01
30	323.15	0.2	0.49	41.7 ± 0.4	43.2	3.52	10.95	0.11 ± 0.01	0.06 ± 0.01
30	323.15	0.3	0.38	30.3 ± 0.4	33.1	4.72	15.59	0.16 ± 0.01	0.06 ± 0.01
30	323.15	0.4	0.35	25.0 ± 0.6	26.9	6.12	20.75	0.20 ± 0.01	0.07 ± 0.01
30	323.15	0.5	0.38	21.2 ± 0.4	23.1	7.64	26.33	0.23 ± 0.01	0.09 ± 0.01
30	323.15	0.6	0.46	20.0 ± 1.4	20.4	9.44	31.95	0.26 ± 0.01	0.12 ± 0.01
30	323.15	0.7	0.57	17.3 ± 0.6	18.4	11.68	37.99	0.29 ± 0.02	0.17 ± 0.01
30	323.15	0.8	0.71	15.2 ± 0.3	16.5	14.82	44.68	0.29 ± 0.01	0.20 ± 0.01
30	323.15	0.9	0.86	12.8 ± 0.4	14.3	18.85	52.25	0.30 ± 0.01	0.26 ± 0.01
30	323.15	1.0	1.00	10.3 ± 0.6	10.2	-	59.42	-	-
30	348.15	0.0	1.00	65.7 ± 0.6	66.7	2.54	-	-	-
30	348.15	0.1	0.77	46.1 ± 0.5	48.7	3.51	10.61	0.11 ± 0.01	0.08 ± 0.01
30	348.15	0.2	0.62	34.8 ± 0.5	37.4	4.67	15.22	0.15 ± 0.01	0.09 ± 0.01
30	348.15	0.3	0.53	27.6 ± 1.0	30.3	5.99	20.62	0.20 ± 0.01	0.10 ± 0.01

Continued on next page

p [MPa]	T [K]	x_{H_2}	Γ^{RFP}	η / [μPas]		D^{self} / [10^{-8}m^2/s]		D^{MS} [10^{-6}m^2/s]	D^{Fick} [10^{-6}m^2/s]
				η^{MD}	η^{RFP}	$D^{self}_{CO_2}$	$D^{self}_{H_2}$		
30	348.15	0.4	0.50	24.5 ± 1.5	26.0	7.42	25.98	0.24 ± 0.01	0.12 ± 0.01
30	348.15	0.5	0.52	22.0 ± 1.8	23.0	9.14	31.58	0.26 ± 0.01	0.14 ± 0.01
30	348.15	0.6	0.58	19.5 ± 0.7	20.8	10.91	37.15	0.28 ± 0.01	0.16 ± 0.01
30	348.15	0.7	0.66	17.8 ± 0.6	18.9	13.54	43.72	0.31 ± 0.01	0.20 ± 0.01
30	348.15	0.8	0.77	16.3 ± 0.5	17.1	16.77	50.45	0.32 ± 0.01	0.24 ± 0.01
30	348.15	0.9	0.88	14.2 ± 1.0	14.9	21.91	59.11	0.32 ± 0.01	0.28 ± 0.01
30	348.15	1.0	1.00	10.3 ± 0.7	10.6	-	67.40	-	-
30	373.15	0.0	1.00	51.4 ± 1.3	54.0	3.44	-	-	-
30	373.15	0.1	0.84	38.8 ± 0.8	41.6	4.59	14.96	0.15 ± 0.01	0.12 ± 0.01
30	373.15	0.2	0.72	31.3 ± 0.7	33.7	5.88	20.22	0.20 ± 0.01	0.14 ± 0.01
30	373.15	0.3	0.65	27.1 ± 1.0	28.9	7.22	25.82	0.23 ± 0.01	0.15 ± 0.01
30	373.15	0.4	0.62	24.0 ± 0.3	25.6	8.79	31.44	0.27 ± 0.01	0.17 ± 0.01
30	373.15	0.5	0.63	22.3 ± 1.2	23.1	10.58	37.05	0.30 ± 0.01	0.19 ± 0.01
30	373.15	0.6	0.66	21.5 ± 2.3	21.2	12.64	43.34	0.33 ± 0.01	0.22 ± 0.01
30	373.15	0.7	0.72	17.0 ± 3.2	19.5	15.37	49.97	0.35 ± 0.01	0.25 ± 0.01
30	373.15	0.8	0.80	16.6 ± 0.3	17.8	19.01	57.62	0.36 ± 0.01	0.29 ± 0.01
30	373.15	0.9	0.89	15.0 ± 1.1	15.5	23.92	66.03	0.36 ± 0.01	0.32 ± 0.01
30	373.15	1.0	1.00	10.4 ± 0.5	11.0	-	74.20	-	-
30	398.15	0.0	1.00	42.9 ± 1.1	45.5	4.57	-	-	-
30	398.15	0.1	0.89	34.4 ± 0.4	37.1	5.78	20.07	0.20 ± 0.01	0.17 ± 0.01
30	398.15	0.2	0.80	30.3 ± 1.3	31.8	7.10	25.90	0.24 ± 0.01	0.20 ± 0.01
30	398.15	0.3	0.74	26.9 ± 0.6	28.1	8.62	30.90	0.28 ± 0.01	0.21 ± 0.01
30	398.15	0.4	0.71	24.0 ± 0.4	25.5	10.13	36.97	0.31 ± 0.01	0.22 ± 0.01

Continued on next page

p [MPa]	T [K]	x_{H_2}	Γ^{RFP}	η / [μPas]		D^{self} / [10^{-8}m^2/s]		D^{MS}	D^{Fick}
				η^{MD}	η^{RFP}	$D^{self}_{CO_2}$	$D^{self}_{H_2}$	[10^{-6}m^2/s]	[10^{-6}m^2/s]
30	398.15	0.5	0.71	22.2 ± 0.7	23.5	12.02	42.73	0.35 ± 0.02	0.25 ± 0.02
30	398.15	0.6	0.72	20.9 ± 0.6	21.8	14.31	49.35	0.37 ± 0.01	0.26 ± 0.01
30	398.15	0.7	0.76	18.9 ± 0.6	20.2	17.36	56.46	0.38 ± 0.01	0.29 ± 0.01
30	398.15	0.8	0.82	17.2 ± 0.3	18.5	21.27	64.21	0.41 ± 0.03	0.34 ± 0.03
30	398.15	0.9	0.90	15.0 ± 0.5	16.1	27.10	74.09	0.42 ± 0.03	0.38 ± 0.03
30	398.15	1.0	1.00	10.7 ± 0.1	11.4	-	83.15	-	-
30	423.15	0.0	1.00	37.9 ± 1.2	40.3	5.73	-	-	-
30	423.15	0.1	0.92	31.9 ± 1.0	34.6	7.00	25.47	0.24 ± 0.01	0.22 ± 0.01
30	423.15	0.2	0.85	28.5 ± 0.8	30.6	8.35	30.68	0.29 ± 0.01	0.24 ± 0.01
30	423.15	0.3	0.81	25.6 ± 0.4	27.8	9.84	36.51	0.33 ± 0.01	0.26 ± 0.01
30	423.15	0.4	0.78	24.2 ± 0.6	25.7	11.67	42.50	0.36 ± 0.01	0.28 ± 0.01
30	423.15	0.5	0.76	22.9 ± 0.4	23.9	13.77	49.00	0.38 ± 0.01	0.29 ± 0.01
30	423.15	0.6	0.77	22.3 ± 2.5	22.4	16.08	55.20	0.40 ± 0.01	0.31 ± 0.01
30	423.15	0.7	0.80	20.0 ± 0.4	20.9	19.04	63.55	0.43 ± 0.01	0.34 ± 0.01
30	423.15	0.8	0.84	18.4 ± 0.4	19.2	24.32	71.16	0.46 ± 0.04	0.39 ± 0.04
30	423.15	0.9	0.91	15.7 ± 1.1	16.7	29.57	81.57	0.43 ± 0.02	0.40 ± 0.01
30	423.15	1.0	1.00	11.7 ± 0.8	11.9	-	91.97	-	-
35	323.15	0.0	1.00	92.7 ± 0.8	91.3	1.73	-	-	-
35	323.15	0.1	0.70	66.8 ± 1.0	64.8	2.32	6.36	0.07 ± 0.01	0.05 ± 0.01
35	323.15	0.2	0.50	47.7 ± 0.5	47.7	3.15	9.27	0.10 ± 0.01	0.05 ± 0.01
35	323.15	0.3	0.38	35.1 ± 0.8	36.6	4.15	13.09	0.14 ± 0.01	0.05 ± 0.01
35	323.15	0.4	0.34	28.4 ± 1.2	29.4	5.38	17.37	0.17 ± 0.01	0.06 ± 0.01
35	323.15	0.5	0.35	22.9 ± 0.1	24.7	6.75	22.23	0.21 ± 0.01	0.07 ± 0.01

Continued on next page

p [MPa]	T [K]	x_{H_2}	Γ^{RFP}	η / [μPas] η^{MD}	η^{RFP}	D^{self} / [10^{-8}m^2/s] $D^{self}_{CO_2}$	$D^{self}_{H_2}$	D^{MS} [10^{-6}m^2/s]	D^{Fick} [10^{-6}m^2/s]
35	323.15	0.6	0.42	20.0 ± 0.4	21.6	8.36	27.09	0.23 ± 0.01	0.10 ± 0.01
35	323.15	0.7	0.53	18.1 ± 0.9	19.1	10.25	32.85	0.24 ± 0.01	0.13 ± 0.01
35	323.15	0.8	0.67	15.6 ± 0.3	16.9	13.15	38.85	0.26 ± 0.01	0.17 ± 0.01
35	323.15	0.9	0.83	14.4 ± 1.1	14.6	17.05	44.87	0.26 ± 0.01	0.22 ± 0.01
35	323.15	1.0	1.00	10.2 ± 0.4	10.3	-	52.23	-	-
35	348.15	0.0	1.00	72.3 ± 1.2	73.3	2.35	-	-	-
35	348.15	0.1	0.77	52.5 ± 0.5	54.0	3.09	8.84	0.09 ± 0.01	0.07 ± 0.01
35	348.15	0.2	0.62	39.0 ± 0.3	41.5	4.09	12.72	0.13 ± 0.01	0.08 ± 0.01
35	348.15	0.3	0.52	34.3 ± 5.6	33.4	5.23	17.15	0.17 ± 0.01	0.09 ± 0.01
35	348.15	0.4	0.48	26.3 ± 0.6	28.0	6.55	21.82	0.20 ± 0.01	0.10 ± 0.01
35	348.15	0.5	0.49	23.2 ± 0.6	24.5	7.97	26.88	0.23 ± 0.01	0.11 ± 0.01
35	348.15	0.6	0.54	20.6 ± 1.2	21.7	9.73	31.98	0.25 ± 0.01	0.14 ± 0.01
35	348.15	0.7	0.62	18.4 ± 0.6	19.5	11.98	37.60	0.27 ± 0.01	0.17 ± 0.01
35	348.15	0.8	0.73	16.4 ± 0.5	17.5	14.85	44.16	0.28 ± 0.01	0.21 ± 0.01
35	348.15	0.9	0.86	13.9 ± 0.3	15.1	18.92	50.72	0.29 ± 0.01	0.25 ± 0.01
35	348.15	1.0	1.00	10.1 ± 0.1	10.7	-	58.28	-	-
35	373.15	0.0	1.00	59.4 ± 0.9	60.5	3.08	-	-	-
35	373.15	0.1	0.83	44.2 ± 0.6	46.5	3.99	12.33	0.12 ± 0.01	0.10 ± 0.01
35	373.15	0.2	0.71	35.3 ± 1.2	37.4	5.09	16.82	0.16 ± 0.01	0.11 ± 0.01
35	373.15	0.3	0.63	29.8 ± 1.5	31.4	6.31	21.49	0.20 ± 0.01	0.13 ± 0.01
35	373.15	0.4	0.60	25.5 ± 0.6	27.4	7.70	26.33	0.23 ± 0.01	0.14 ± 0.01
35	373.15	0.5	0.59	23.4 ± 1.2	24.4	9.21	31.61	0.26 ± 0.01	0.16 ± 0.01
35	373.15	0.6	0.63	20.7 ± 0.7	22.1	11.14	36.85	0.28 ± 0.01	0.18 ± 0.01

Continued on next page

p [MPa]	T [K]	x_{H_2}	Γ^{RFP}	η / [μPas] η^{MD}	η^{RFP}	D^{self} / [10^{-8}m^2/s] $D^{self}_{CO_2}$	$D^{self}_{H_2}$	D^{MS} [10^{-6}m^2/s]	D^{Fick} [10^{-6}m^2/s]
35	373.15	0.7	0.69	19.4 ± 0.5	20.1	13.41	42.85	0.30 ± 0.01	0.21 ± 0.01
35	373.15	0.8	0.77	15.7 ± 1.6	18.1	16.69	49.43	0.32 ± 0.01	0.24 ± 0.01
35	373.15	0.9	0.88	14.3 ± 0.3	15.7	21.46	57.21	0.32 ± 0.01	0.28 ± 0.01
35	373.15	1.0	1.00	10.5 ± 0.2	11.2	-	65.08	-	-
35	398.15	0.0	1.00	49.2 ± 1.5	51.5	3.94	-	-	-
35	398.15	0.1	0.88	39.2 ± 1.2	41.5	5.02	16.51	0.16 ± 0.01	0.14 ± 0.01
35	398.15	0.2	0.78	32.6 ± 1.4	34.8	6.13	20.97	0.20 ± 0.01	0.16 ± 0.01
35	398.15	0.3	0.72	28.6 ± 0.2	30.4	7.46	25.44	0.24 ± 0.01	0.17 ± 0.01
35	398.15	0.4	0.68	25.2 ± 0.5	27.1	8.95	30.95	0.27 ± 0.01	0.18 ± 0.01
35	398.15	0.5	0.67	23.7 ± 0.4	24.6	10.57	35.95	0.34 ± 0.07	0.23 ± 0.05
35	398.15	0.6	0.69	23.7 ± 2.9	22.5	12.45	42.04	0.31 ± 0.01	0.21 ± 0.01
35	398.15	0.7	0.74	19.1 ± 0.4	20.7	15.05	47.83	0.34 ± 0.01	0.25 ± 0.01
35	398.15	0.8	0.80	17.8 ± 0.6	18.8	18.53	55.23	0.34 ± 0.01	0.27 ± 0.01
35	398.15	0.9	0.89	14.5 ± 0.2	16.3	24.37	64.02	0.36 ± 0.03	0.32 ± 0.03
35	398.15	1.0	1.00	11.1 ± 0.3	11.6	-	72.72	-	-
35	423.15	0.0	1.00	42.3 ± 1.1	45.5	4.96	-	-	-
35	423.15	0.1	0.91	36.1 ± 0.9	38.3	6.07	20.93	0.20 ± 0.01	0.19 ± 0.01
35	423.15	0.2	0.84	31.2 ± 0.7	33.3	7.27	25.62	0.24 ± 0.01	0.20 ± 0.01
35	423.15	0.3	0.78	27.6 ± 0.2	29.8	8.62	30.59	0.28 ± 0.01	0.22 ± 0.01
35	423.15	0.4	0.75	26.1 ± 1.1	27.1	10.13	35.72	0.31 ± 0.01	0.23 ± 0.01
35	423.15	0.5	0.73	23.4 ± 0.6	24.9	11.89	41.45	0.33 ± 0.01	0.24 ± 0.01
35	423.15	0.6	0.74	22.0 ± 0.5	23.1	14.15	47.47	0.35 ± 0.01	0.26 ± 0.01
35	423.15	0.7	0.77	19.6 ± 0.2	21.3	17.00	53.94	0.38 ± 0.03	0.29 ± 0.02

Continued on next page

p	T	x_{H_2}	Γ^{RFP}	η / [μPas]		D^{self} / [10^{-8}m^2/s]		D^{MS}	D^{Fick}
[MPa]	[K]			η^{MD}	η^{RFP}	$D^{self}_{CO_2}$	$D^{self}_{H_2}$	[10^{-6}m^2/s]	[10^{-6}m^2/s]
35	423.15	0.8	0.82	18.0 ± 0.2	19.4	20.61	61.37	0.38 ± 0.01	0.32 ± 0.01
35	423.15	0.9	0.90	15.0 ± 0.1	16.9	26.38	70.79	0.38 ± 0.01	0.35 ± 0.01
35	423.15	1.0	1.00	11.2 ± 0.2	12.0	-	80.06	-	-
40	323.15	0.0	1.00	99.6 ± 1.2	97.3	1.63	-	-	-
40	323.15	0.1	0.71	72.2 ± 0.7	69.7	2.16	5.77	0.06 ± 0.01	0.04 ± 0.01
40	323.15	0.2	0.51	52.0 ± 0.2	51.7	2.84	8.02	0.09 ± 0.01	0.04 ± 0.01
40	323.15	0.3	0.39	38.7 ± 0.3	39.8	3.75	11.22	0.12 ± 0.01	0.05 ± 0.01
40	323.15	0.4	0.33	30.2 ± 0.2	31.8	4.77	15.00	0.16 ± 0.01	0.05 ± 0.01
40	323.15	0.5	0.34	24.5 ± 0.9	26.3	6.06	19.03	0.18 ± 0.01	0.06 ± 0.01
40	323.15	0.6	0.40	21.6 ± 1.1	22.5	7.50	23.75	0.21 ± 0.01	0.08 ± 0.01
40	323.15	0.7	0.50	18.4 ± 0.4	19.8	9.32	28.58	0.22 ± 0.01	0.11 ± 0.01
40	323.15	0.8	0.64	15.9 ± 0.2	17.4	11.87	33.79	0.23 ± 0.01	0.15 ± 0.01
40	323.15	0.9	0.81	13.5 ± 0.1	14.8	15.68	40.18	0.23 ± 0.01	0.19 ± 0.01
40	323.15	1.0	1.00	10.1 ± 0.1	10.5	-	46.22	-	-
40	348.15	0.0	1.00	79.4 ± 0.6	79.1	2.17	-	-	-
40	348.15	0.1	0.78	58.7 ± 0.6	58.8	2.81	7.94	0.08 ± 0.01	0.06 ± 0.01
40	348.15	0.2	0.62	44.2 ± 0.5	45.3	3.66	10.83	0.11 ± 0.01	0.07 ± 0.01
40	348.15	0.3	0.51	34.2 ± 0.5	36.2	4.69	14.57	0.14 ± 0.01	0.07 ± 0.01
40	348.15	0.4	0.47	28.5 ± 0.6	30.1	5.84	18.62	0.18 ± 0.01	0.08 ± 0.01
40	348.15	0.5	0.47	24.7 ± 0.6	25.7	7.13	23.10	0.20 ± 0.01	0.10 ± 0.01
40	348.15	0.6	0.51	21.1 ± 0.4	22.7	8.76	27.76	0.23 ± 0.01	0.12 ± 0.01
40	348.15	0.7	0.59	19.2 ± 0.5	20.2	10.72	32.92	0.25 ± 0.01	0.15 ± 0.01
40	348.15	0.8	0.70	16.6 ± 0.4	17.9	13.85	38.65	0.25 ± 0.01	0.17 ± 0.01

Continued on next page

p [MPa]	T [K]	x_{H_2}	Γ^{RFP}	η / [μPas] η^{MD}	η^{RFP}	D^{self} / [10^{-8}m^2/s] $D^{self}_{CO_2}$	$D^{self}_{H_2}$	D^{MS} [10^{-6}m^2/s]	D^{Fick} [10^{-6}m^2/s]
40	348.15	0.9	0.84	14.2 ± 0.6	15.4	17.19	45.40	0.27 ± 0.01	0.23 ± 0.01
40	348.15	1.0	1.00	10.4 ± 0.3	10.9	-	52.01	-	-
40	373.15	0.0	1.00	63.7 ± 0.3	66.2	2.80	-	-	-
40	373.15	0.1	0.83	49.3 ± 0.2	51.0	3.58	10.61	0.11 ± 0.01	0.09 ± 0.01
40	373.15	0.2	0.70	39.0 ± 0.7	40.9	4.58	14.04	0.14 ± 0.01	0.10 ± 0.01
40	373.15	0.3	0.62	32.1 ± 0.2	33.9	5.66	18.23	0.18 ± 0.01	0.11 ± 0.01
40	373.15	0.4	0.58	28.0 ± 0.4	29.0	6.86	22.52	0.20 ± 0.01	0.12 ± 0.01
40	373.15	0.5	0.57	23.9 ± 0.5	25.7	8.30	27.54	0.23 ± 0.01	0.13 ± 0.01
40	373.15	0.6	0.60	21.7 ± 0.8	23.0	9.95	32.08	0.25 ± 0.01	0.15 ± 0.01
40	373.15	0.7	0.66	19.1 ± 0.5	20.7	12.38	37.44	0.27 ± 0.01	0.18 ± 0.01
40	373.15	0.8	0.75	17.5 ± 0.3	18.5	14.97	43.55	0.28 ± 0.01	0.21 ± 0.01
40	373.15	0.9	0.86	14.5 ± 0.7	15.9	19.55	50.88	0.29 ± 0.01	0.25 ± 0.01
40	373.15	1.0	1.00	10.7 ± 0.2	11.3	-	57.53	-	-
40	398.15	0.0	1.00	54.2 ± 0.8	56.9	3.56	-	-	-
40	398.15	0.1	0.87	43.0 ± 0.6	45.6	4.47	13.92	0.14 ± 0.01	0.12 ± 0.01
40	398.15	0.2	0.77	35.6 ± 0.5	37.9	5.50	17.71	0.17 ± 0.01	0.13 ± 0.01
40	398.15	0.3	0.70	30.7 ± 0.6	32.4	6.61	22.15	0.21 ± 0.01	0.14 ± 0.01
40	398.15	0.4	0.66	26.6 ± 0.4	28.7	8.00	26.72	0.23 ± 0.01	0.15 ± 0.01
40	398.15	0.5	0.65	24.6 ± 0.5	25.7	9.44	31.58	0.26 ± 0.01	0.17 ± 0.01
40	398.15	0.6	0.67	21.6 ± 0.4	23.3	11.33	36.82	0.28 ± 0.01	0.18 ± 0.01
40	398.15	0.7	0.71	20.0 ± 0.3	21.2	13.64	42.44	0.30 ± 0.01	0.21 ± 0.01
40	398.15	0.8	0.78	17.5 ± 0.4	19.1	16.77	48.52	0.31 ± 0.01	0.24 ± 0.01
40	398.15	0.9	0.88	15.5 ± 1.5	16.5	21.24	56.52	0.33 ± 0.01	0.29 ± 0.01

Continued on next page

p [MPa]	T [K]	x_{H_2}	Γ^{RFP}	η / [μPas] η^{MD}	η^{RFP}	D^{self} / [10^{-8}m^2/s] $D^{self}_{CO_2}$	$D^{self}_{H_2}$	D^{MS} [10^{-6}m^2/s]	D^{Fick} [10^{-6}m^2/s]
40	398.15	1.0	1.00	11.1 ± 0.1	11.7	-	64.00	-	-
40	423.15	0.0	1.00	46.8 ± 0.6	50.3	4.38	-	-	-
40	423.15	0.1	0.90	39.6 ± 1.4	41.9	5.35	17.42	0.17 ± 0.01	0.15 ± 0.01
40	423.15	0.2	0.82	34.7 ± 2.1	35.9	6.45	21.57	0.21 ± 0.01	0.17 ± 0.01
40	423.15	0.3	0.76	30.0 ± 0.8	31.7	7.63	26.13	0.24 ± 0.01	0.18 ± 0.01
40	423.15	0.4	0.73	27.0 ± 0.8	28.5	9.05	30.92	0.27 ± 0.01	0.19 ± 0.01
40	423.15	0.5	0.71	24.4 ± 1.0	25.9	10.62	36.00	0.29 ± 0.01	0.21 ± 0.01
40	423.15	0.6	0.72	22.4 ± 0.3	23.8	12.66	41.39	0.31 ± 0.01	0.22 ± 0.01
40	423.15	0.7	0.75	20.4 ± 0.4	21.8	15.14	47.69	0.32 ± 0.01	0.24 ± 0.01
40	423.15	0.8	0.81	18.2 ± 0.3	19.7	18.51	54.32	0.34 ± 0.01	0.27 ± 0.01
40	423.15	0.9	0.89	16.1 ± 1.4	17.1	23.32	62.34	0.35 ± 0.01	0.31 ± 0.01
40	423.15	1.0	1.00	11.7 ± 0.7	12.1	-	70.81	-	-
45	323.15	0.0	1.00	106.0 ± 1.2	102.9	1.56	-	-	-
45	323.15	0.1	0.72	77.0 ± 0.5	74.3	2.01	5.29	0.05 ± 0.01	0.04 ± 0.01
45	323.15	0.2	0.52	57.7 ± 1.7	55.4	2.67	7.33	0.08 ± 0.01	0.04 ± 0.01
45	323.15	0.3	0.39	42.5 ± 0.3	42.7	3.45	9.97	0.11 ± 0.01	0.04 ± 0.01
45	323.15	0.4	0.33	32.7 ± 0.4	34.0	4.40	13.36	0.14 ± 0.01	0.05 ± 0.01
45	323.15	0.5	0.32	26.2 ± 0.3	27.9	5.50	16.91	0.17 ± 0.01	0.05 ± 0.01
45	323.15	0.6	0.37	21.9 ± 0.4	23.6	6.92	20.82	0.19 ± 0.01	0.07 ± 0.01
45	323.15	0.7	0.47	19.0 ± 0.1	20.4	8.54	25.43	0.21 ± 0.01	0.10 ± 0.01
45	323.15	0.8	0.61	17.0 ± 0.6	17.8	10.89	30.62	0.21 ± 0.01	0.13 ± 0.01
45	323.15	0.9	0.79	13.7 ± 0.5	15.1	14.12	36.20	0.21 ± 0.01	0.17 ± 0.01
45	323.15	1.0	1.00	10.2 ± 0.1	10.6	-	41.86	-	-

Continued on next page

p [MPa]	T [K]	x_{H_2}	Γ^{RFP}	η / [μPas] η^{MD}	η^{RFP}	D^{self} / [10^{-8}m^2/s] $D^{self}_{CO_2}$	$D^{self}_{H_2}$	D^{MS} [10^{-6}m^2/s]	D^{Fick} [10^{-6}m^2/s]
45	348.15	0.0	1.00	86.1 ± 1.2	84.5	2.02	-	-	-
45	348.15	0.1	0.78	63.3 ± 0.9	63.1	2.60	7.14	0.07 ± 0.01	0.06 ± 0.01
45	348.15	0.2	0.62	48.4 ± 0.6	48.7	3.35	9.69	0.10 ± 0.01	0.06 ± 0.01
45	348.15	0.3	0.51	38.3 ± 1.9	38.9	4.26	12.83	0.13 ± 0.01	0.06 ± 0.01
45	348.15	0.4	0.45	30.4 ± 0.2	32.0	5.33	16.38	0.16 ± 0.01	0.07 ± 0.01
45	348.15	0.5	0.45	25.4 ± 0.4	27.1	6.53	20.29	0.19 ± 0.01	0.08 ± 0.01
45	348.15	0.6	0.49	22.1 ± 0.4	23.5	8.01	24.75	0.21 ± 0.01	0.10 ± 0.01
45	348.15	0.7	0.56	19.5 ± 0.8	20.8	9.85	29.55	0.22 ± 0.01	0.12 ± 0.01
45	348.15	0.8	0.68	17.1 ± 0.4	18.3	12.34	34.71	0.23 ± 0.01	0.16 ± 0.01
45	348.15	0.9	0.82	14.3 ± 0.2	15.6	15.64	40.76	0.25 ± 0.01	0.20 ± 0.01
45	348.15	1.0	1.00	10.8 ± 0.4	11.0	-	47.17	-	-
45	373.15	0.0	1.00	70.1 ± 0.7	71.3	2.58	-	-	-
45	373.15	0.1	0.83	53.7 ± 0.5	55.1	3.29	9.48	0.10 ± 0.01	0.08 ± 0.01
45	373.15	0.2	0.70	42.3 ± 0.6	44.0	4.14	12.41	0.12 ± 0.01	0.09 ± 0.01
45	373.15	0.3	0.61	34.5 ± 0.4	36.3	5.16	16.10	0.15 ± 0.01	0.09 ± 0.01
45	373.15	0.4	0.56	29.0 ± 0.1	30.8	6.23	19.78	0.19 ± 0.01	0.11 ± 0.01
45	373.15	0.5	0.55	24.9 ± 0.4	26.8	7.62	24.15	0.20 ± 0.01	0.11 ± 0.01
45	373.15	0.6	0.57	22.7 ± 0.5	23.8	9.06	28.53	0.23 ± 0.01	0.13 ± 0.01
45	373.15	0.7	0.63	19.7 ± 0.3	21.2	11.03	33.26	0.25 ± 0.01	0.16 ± 0.01
45	373.15	0.8	0.73	17.6 ± 0.9	18.8	13.73	39.31	0.26 ± 0.01	0.19 ± 0.01
45	373.15	0.9	0.85	14.9 ± 0.8	16.1	17.57	45.52	0.27 ± 0.01	0.23 ± 0.01
45	373.15	1.0	1.00	10.7 ± 0.3	11.4	-	52.40	-	-
45	398.15	0.0	1.00	59.4 ± 0.7	61.7	3.24	-	-	-

Continued on next page

p [MPa]	T [K]	x_{H_2}	Γ^{RFP}	η / [μPas] η^{MD}	η^{RFP}	D^{self} / [10^{-8}m^2/s] $D^{self}_{CO_2}$	$D^{self}_{H_2}$	D^{MS} [10^{-6}m^2/s]	D^{Fick} [10^{-6}m^2/s]
45	398.15	0.1	0.87	47.0 ± 0.6	49.3	4.05	12.26	0.12 ± 0.01	0.10 ± 0.01
45	398.15	0.2	0.76	38.4 ± 0.5	40.7	4.98	15.57	0.15 ± 0.01	0.12 ± 0.01
45	398.15	0.3	0.69	33.5 ± 0.9	34.6	5.99	19.45	0.18 ± 0.01	0.12 ± 0.01
45	398.15	0.4	0.64	28.6 ± 0.5	30.1	7.23	23.32	0.21 ± 0.01	0.13 ± 0.01
45	398.15	0.5	0.63	25.0 ± 0.6	26.8	8.60	27.95	0.24 ± 0.01	0.15 ± 0.01
45	398.15	0.6	0.64	22.8 ± 0.7	24.1	10.35	32.55	0.26 ± 0.01	0.17 ± 0.01
45	398.15	0.7	0.69	20.9 ± 0.8	21.7	12.32	37.67	0.27 ± 0.01	0.18 ± 0.01
45	398.15	0.8	0.76	18.4 ± 0.7	19.4	15.25	43.90	0.29 ± 0.02	0.22 ± 0.01
45	398.15	0.9	0.87	15.7 ± 0.8	16.7	19.79	50.68	0.29 ± 0.01	0.25 ± 0.01
45	398.15	1.0	1.00	11.1 ± 0.1	11.8	-	58.25	-	-
45	423.15	0.0	1.00	51.7 ± 0.5	54.8	3.99	-	-	-
45	423.15	0.1	0.90	44.0 ± 1.6	45.3	4.81	15.08	0.15 ± 0.01	0.13 ± 0.01
45	423.15	0.2	0.81	36.2 ± 0.4	38.5	5.80	18.73	0.18 ± 0.01	0.15 ± 0.01
45	423.15	0.3	0.75	31.7 ± 0.5	33.5	6.93	22.95	0.21 ± 0.01	0.16 ± 0.01
45	423.15	0.4	0.71	29.9 ± 4.2	29.9	8.17	27.00	0.24 ± 0.01	0.17 ± 0.01
45	423.15	0.5	0.69	26.0 ± 0.9	27.0	9.76	31.82	0.26 ± 0.01	0.18 ± 0.01
45	423.15	0.6	0.70	22.8 ± 0.4	24.5	11.51	36.60	0.28 ± 0.01	0.19 ± 0.01
45	423.15	0.7	0.73	20.8 ± 1.2	22.3	13.77	41.91	0.29 ± 0.01	0.21 ± 0.01
45	423.15	0.8	0.79	18.3 ± 0.2	20.0	16.97	48.92	0.32 ± 0.01	0.25 ± 0.01
45	423.15	0.9	0.88	15.7 ± 0.2	17.3	21.67	55.98	0.32 ± 0.01	0.28 ± 0.01
45	423.15	1.0	1.00	11.7 ± 0.3	12.2	-	64.12	-	-
50	323.15	0.0	1.00	111.1 ± 1.3	108.3	1.48	-	-	-
50	323.15	0.1	0.73	82.8 ± 1.0	78.6	1.92	4.93	0.05 ± 0.01	0.04 ± 0.01

Continued on next page

p [MPa]	T [K]	x_{H_2}	Γ^{RFP}	η / [μPas]		D^{self} / [10^{-8}m^2/s]		D^{MS} [10^{-6}m^2/s]	D^{Fick} [10^{-6}m^2/s]
				η^{MD}	η^{RFP}	$D^{self}_{CO_2}$	$D^{self}_{H_2}$		
50	323.15	0.2	0.52	61.5 ± 0.6	58.8	2.46	6.70	0.07 ± 0.01	0.04 ± 0.01
50	323.15	0.3	0.39	46.0 ± 0.6	45.4	3.20	9.01	0.10 ± 0.01	0.04 ± 0.01
50	323.15	0.4	0.32	35.0 ± 0.4	36.1	4.08	11.97	0.13 ± 0.01	0.04 ± 0.01
50	323.15	0.5	0.31	28.1 ± 0.5	29.5	5.10	15.22	0.16 ± 0.01	0.05 ± 0.01
50	323.15	0.6	0.35	23.7 ± 1.1	24.7	6.38	18.95	0.18 ± 0.01	0.06 ± 0.01
50	323.15	0.7	0.45	19.9 ± 0.5	21.1	7.92	22.88	0.19 ± 0.01	0.08 ± 0.01
50	323.15	0.8	0.59	17.1 ± 0.2	18.2	10.07	27.64	0.20 ± 0.01	0.12 ± 0.01
50	323.15	0.9	0.78	13.7 ± 0.6	15.3	12.91	32.79	0.20 ± 0.01	0.16 ± 0.01
50	323.15	1.0	1.00	10.4 ± 0.1	10.8	-	38.29	-	-
50	348.15	0.0	1.00	90.4 ± 1.0	89.5	1.94	-	-	-
50	348.15	0.1	0.78	68.5 ± 0.8	67.1	2.44	6.56	0.07 ± 0.01	0.05 ± 0.01
50	348.15	0.2	0.62	51.9 ± 1.2	51.9	3.12	8.78	0.09 ± 0.01	0.06 ± 0.01
50	348.15	0.3	0.50	40.3 ± 0.8	41.4	3.96	11.55	0.12 ± 0.01	0.06 ± 0.01
50	348.15	0.4	0.44	32.3 ± 0.2	34.0	4.90	14.80	0.14 ± 0.01	0.06 ± 0.01
50	348.15	0.5	0.43	27.5 ± 1.4	28.5	6.01	18.27	0.18 ± 0.01	0.08 ± 0.01
50	348.15	0.6	0.47	23.5 ± 1.2	24.5	7.37	22.14	0.19 ± 0.01	0.09 ± 0.01
50	348.15	0.7	0.54	20.0 ± 0.5	21.3	9.16	26.63	0.21 ± 0.01	0.12 ± 0.01
50	348.15	0.8	0.66	17.5 ± 0.5	18.7	11.47	31.72	0.22 ± 0.01	0.14 ± 0.01
50	348.15	0.9	0.81	14.5 ± 0.4	15.8	14.77	37.29	0.23 ± 0.01	0.19 ± 0.01
50	348.15	1.0	1.00	10.7 ± 0.1	11.2	-	42.80	-	-
50	373.15	0.0	1.00	74.9 ± 0.8	76.1	2.45	-	-	-
50	373.15	0.1	0.83	58.0 ± 0.7	58.9	3.08	8.63	0.09 ± 0.01	0.07 ± 0.01
50	373.15	0.2	0.69	45.4 ± 0.4	47.0	3.84	11.09	0.11 ± 0.01	0.08 ± 0.01

Continued on next page

p [MPa]	T [K]	x_{H_2}	Γ^{RFP}	η / [μPas]		D^{self} / [10^{-8}m^2/s]		D^{MS} [10^{-6}m^2/s]	D^{Fick} [10^{-6}m^2/s]
				η^{MD}	η^{RFP}	$D^{self}_{CO_2}$	$D^{self}_{H_2}$		
50	373.15	0.3	0.60	37.2 ± 0.8	38.6	4.72	14.24	0.14 ± 0.01	0.08 ± 0.01
50	373.15	0.4	0.54	31.2 ± 0.6	32.5	5.73	17.70	0.17 ± 0.01	0.09 ± 0.01
50	373.15	0.5	0.53	26.3 ± 0.3	28.0	7.01	21.60	0.19 ± 0.01	0.10 ± 0.01
50	373.15	0.6	0.55	22.9 ± 0.5	24.5	8.41	25.60	0.21 ± 0.01	0.12 ± 0.01
50	373.15	0.7	0.61	21.2 ± 0.8	21.8	10.26	30.47	0.23 ± 0.01	0.14 ± 0.01
50	373.15	0.8	0.71	17.7 ± 0.3	19.2	12.79	35.65	0.24 ± 0.01	0.17 ± 0.01
50	373.15	0.9	0.84	16.1 ± 1.4	16.4	16.06	41.70	0.25 ± 0.01	0.21 ± 0.01
50	373.15	1.0	1.00	11.1 ± 0.1	11.6	-	48.04	-	-
50	398.15	0.0	1.00	64.1 ± 1.1	66.2	3.02	-	-	-
50	398.15	0.1	0.86	50.9 ± 0.5	52.9	3.72	10.91	0.11 ± 0.01	0.09 ± 0.01
50	398.15	0.2	0.75	41.5 ± 0.6	43.5	4.56	13.79	0.13 ± 0.01	0.10 ± 0.01
50	398.15	0.3	0.67	34.5 ± 1.6	36.7	5.52	17.04	0.16 ± 0.01	0.11 ± 0.01
50	398.15	0.4	0.63	30.0 ± 0.5	31.6	6.62	20.88	0.19 ± 0.01	0.12 ± 0.01
50	398.15	0.5	0.61	26.6 ± 0.8	27.8	7.94	24.94	0.21 ± 0.01	0.13 ± 0.01
50	398.15	0.6	0.62	23.1 ± 0.3	24.9	9.52	29.53	0.23 ± 0.01	0.15 ± 0.01
50	398.15	0.7	0.67	20.3 ± 0.7	22.3	11.55	34.30	0.26 ± 0.01	0.17 ± 0.01
50	398.15	0.8	0.75	18.3 ± 0.1	19.8	14.14	39.61	0.26 ± 0.01	0.20 ± 0.01
50	398.15	0.9	0.86	16.3 ± 0.6	16.9	17.75	46.00	0.26 ± 0.01	0.23 ± 0.01
50	398.15	1.0	1.00	11.6 ± 0.2	11.9	-	53.02	-	-
50	423.15	0.0	1.00	57.3 ± 0.5	59.0	3.64	-	-	-
50	423.15	0.1	0.89	45.8 ± 0.5	48.5	4.45	13.54	0.13 ± 0.01	0.12 ± 0.01
50	423.15	0.2	0.80	39.0 ± 0.9	40.9	5.35	16.68	0.16 ± 0.01	0.13 ± 0.01
50	423.15	0.3	0.73	33.7 ± 0.6	35.4	6.39	20.24	0.19 ± 0.01	0.14 ± 0.01

Continued on next page

p [MPa]	T [K]	x_{H_2}	Γ^{RFP}	η / [μPas]		D^{self} / [10^{-8}m^2/s]		D^{MS} [10^{-6}m^2/s]	D^{Fick} [10^{-6}m^2/s]
				η^{MD}	η^{RFP}	$D^{self}_{CO_2}$	$D^{self}_{H_2}$		
50	423.15	0.4	0.69	29.9 ± 0.5	31.1	7.56	24.18	0.22 ± 0.01	0.15 ± 0.01
50	423.15	0.5	0.67	26.9 ± 0.7	28.0	8.93	28.63	0.24 ± 0.01	0.16 ± 0.01
50	423.15	0.6	0.68	23.9 ± 1.0	25.2	10.64	33.01	0.26 ± 0.01	0.18 ± 0.01
50	423.15	0.7	0.71	24.8 ± 6.0	22.8	12.79	38.12	0.27 ± 0.01	0.19 ± 0.01
50	423.15	0.8	0.78	18.8 ± 0.6	20.3	15.64	44.13	0.29 ± 0.01	0.22 ± 0.01
50	423.15	0.9	0.87	16.2 ± 0.7	17.4	19.87	50.83	0.30 ± 0.01	0.26 ± 0.01
50	423.15	1.0	1.00	12.1 ± 0.2	12.3	-	58.14	-	-

S10.3 Phase equilibria for CO_2-NaCl brine, H_2-NaCl brine, and CO_2-H_2-NaCl systems

Table S4: Phase equilibria for CO_2-NaCl brine systems from CFCMC simulations[24–26] for various pressures, temperatures, and NaCl concentrations (c_{NaCl} in units of mol NaCl/kg H_2O). Solubilities of CO_2 in the liquid-rich phase are denoted by $x_{\mathrm{CO_2}}$, the water content in the gas-rich phase is denoted by $y_{\mathrm{H_2O}}$, and the fugacity coefficients (ϕ) of the two species in the gas-rich phase are compared to the corresponding values from REFPROP (RFP)[3]. Fugacity coefficients from REFPROP for CO_2 and H_2O at a given pressure, and temperature are obtained at the gas-phase composition obtained from CFCMC simulations.

p	T	c_{NaCl}	$x_{\mathrm{CO_2}}$ / [10^{-4}]	$y_{\mathrm{H_2O}}$ / [10^{-4}]	$\phi_{\mathrm{CO_2}}$		$\phi_{\mathrm{H_2O}}$	
[MPa]	[K]				CFCMC	RFP	CFCMC	RFP
5	323.15	0	102 ± 7	32 ± 3	0.81 ± 0.01	0.82	0.74 ± 0.01	0.60
5	423.15	0	63 ± 2	1294 ± 7	0.94 ± 0.00	0.93	0.76 ± 0.00	0.81
10	323.15	0	156 ± 7	30 ± 4	0.65 ± 0.01	0.64	0.49 ± 0.01	0.26
10	423.15	0	126 ± 3	745 ± 10	0.88 ± 0.00	0.87	0.68 ± 0.00	0.67
30	323.15	0	186 ± 5	39 ± 6	0.34 ± 0.00	0.33	0.14 ± 0.00	0.09
30	423.15	0	270 ± 4	458 ± 15	0.71 ± 0.00	0.69	0.41 ± 0.00	0.36
50	323.15	0	206 ± 12	33 ± 5	0.30 ± 0.00	0.29	0.10 ± 0.00	0.08
50	423.15	0	345 ± 10	428 ± 18	0.66 ± 0.00	0.63	0.29 ± 0.01	0.29
5	323.15	1	79 ± 5	31 ± 4	0.82 ± 0.01	0.82	0.75 ± 0.01	0.60
5	423.15	1	53 ± 2	1246 ± 7	0.93 ± 0.00	0.93	0.76 ± 0.00	0.81
10	323.15	1	126 ± 14	31 ± 6	0.66 ± 0.01	0.64	0.49 ± 0.01	0.26
10	423.15	1	102 ± 3	707 ± 10	0.88 ± 0.00	0.87	0.69 ± 0.00	0.67
30	323.15	1	145 ± 9	27 ± 5	0.34 ± 0.00	0.33	0.14 ± 0.00	0.09
30	423.15	1	218 ± 9	438 ± 19	0.71 ± 0.00	0.69	0.41 ± 0.00	0.36
50	323.15	1	166 ± 4	34 ± 3	0.29 ± 0.00	0.29	0.10 ± 0.00	0.08

Continued on next page

p [MPa]	T [K]	c_{NaCl}	x_{CO_2} / [10^{-4}]	y_{H_2O} / [10^{-4}]	ϕ_{CO_2}		ϕ_{H_2O}	
					CFCMC	RFP	CFCMC	RFP
50	423.15	1	270 ± 8	400 ± 21	0.66 ± 0.00	0.63	0.30 ± 0.00	0.29
5	323.15	2	67 ± 5	31 ± 3	0.83 ± 0.00	0.82	0.76 ± 0.00	0.60
5	423.15	2	41 ± 2	1197 ± 6	0.94 ± 0.00	0.93	0.77 ± 0.00	0.81
10	323.15	2	95 ± 8	24 ± 4	0.65 ± 0.01	0.64	0.50 ± 0.01	0.26
10	423.15	2	83 ± 4	675 ± 9	0.87 ± 0.00	0.87	0.69 ± 0.00	0.67
30	323.15	2	117 ± 7	34 ± 7	0.34 ± 0.00	0.33	0.15 ± 0.00	0.09
30	423.15	2	175 ± 7	406 ± 5	0.71 ± 0.01	0.69	0.42 ± 0.01	0.36
50	323.15	2	129 ± 7	33 ± 6	0.29 ± 0.00	0.29	0.10 ± 0.00	0.08
50	423.15	2	218 ± 6	384 ± 16	0.66 ± 0.00	0.63	0.31 ± 0.00	0.29

Table S5: Phase equilibria for H_2-NaCl brine systems from CFCMC simulations[24–26] for various pressures, temperatures, and NaCl concentrations (c_{NaCl} in units of mol NaCl/kg H_2O). Solubilities of H_2 in the liquid-rich phase is denoted by x_{H_2}, the water content in the gas-rich phase is denoted by y_{H_2O}, and the fugacity coefficients (ϕ) of the two species in the gas-rich phase are compared to the corresponding values from REFPROP (RFP)[3]. Fugacity coefficients from REFPROP for H_2 and H_2O at a given pressure, and temperature are obtained at the gas-phase composition obtained from CFCMC simulations.

p [MPa]	T [K]	c_{NaCl}	x_{H_2} / [10^{-4}]	y_{H_2O} / [10^{-4}]	ϕ_{H_2} CFCMC	ϕ_{H_2} RFP	ϕ_{H_2O} CFCMC	ϕ_{H_2O} RFP
5	323.15	0	7 ± 2	26 ± 4	1.02 ± 0.01	1.03	0.97 ± 0.01	0.96
5	423.15	0	11 ± 0	1185 ± 11	1.02 ± 0.00	1.02	0.83 ± 0.00	0.96
10	323.15	0	13 ± 2	12 ± 2	1.04 ± 0.01	1.06	0.97 ± 0.01	0.92
10	423.15	0	24 ± 1	609 ± 15	1.04 ± 0.00	1.05	0.84 ± 0.00	0.95
30	323.15	0	42 ± 5	4 ± 1	1.17 ± 0.01	1.19	1.01 ± 0.01	0.85
30	423.15	0	71 ± 3	213 ± 6	1.14 ± 0.01	1.15	0.89 ± 0.01	0.94
50	323.15	0	60 ± 6	3 ± 1	1.31 ± 0.00	1.34	1.08 ± 0.00	0.85
50	423.15	0	113 ± 5	134 ± 9	1.24 ± 0.01	1.26	0.95 ± 0.01	0.98
5	323.15	1	6 ± 1	25 ± 5	1.01 ± 0.01	1.03	0.96 ± 0.01	0.96
5	423.15	1	10 ± 1	1149 ± 5	1.02 ± 0.00	1.02	0.84 ± 0.00	0.96
10	323.15	1	12 ± 2	15 ± 2	1.05 ± 0.02	1.06	0.98 ± 0.02	0.92
10	423.15	1	20 ± 1	580 ± 7	1.04 ± 0.01	1.05	0.84 ± 0.00	0.95
30	323.15	1	33 ± 3	5 ± 1	1.16 ± 0.01	1.19	1.01 ± 0.00	0.85
30	423.15	1	62 ± 1	202 ± 6	1.14 ± 0.01	1.15	0.90 ± 0.00	0.94
50	323.15	1	54 ± 2	3 ± 1	1.30 ± 0.00	1.34	1.07 ± 0.00	0.85
50	423.15	1	96 ± 3	133 ± 3	1.24 ± 0.01	1.26	0.95 ± 0.00	0.98
5	323.15	2	5 ± 1	21 ± 2	1.02 ± 0.01	1.03	0.97 ± 0.01	0.96

Continued on next page

p [MPa]	T [K]	c_{NaCl}	x_{H_2} / [10^{-4}]	y_{H_2O} / [10^{-4}]	ϕ_{H_2}		ϕ_{H_2O}	
					CFCMC	RFP	CFCMC	RFP
5	423.15	2	9 ± 1	1095 ± 12	1.02 ± 0.00	1.02	0.84 ± 0.00	0.96
10	323.15	2	9 ± 2	14 ± 3	1.05 ± 0.00	1.06	0.98 ± 0.00	0.92
10	423.15	2	18 ± 1	561 ± 5	1.04 ± 0.00	1.05	0.85 ± 0.00	0.95
30	323.15	2	28 ± 3	5 ± 2	1.17 ± 0.01	1.19	1.02 ± 0.01	0.85
30	423.15	2	55 ± 3	197 ± 7	1.14 ± 0.00	1.15	0.90 ± 0.00	0.95
50	323.15	2	47 ± 6	2 ± 3	1.30 ± 0.01	1.34	1.08 ± 0.01	0.85
50	423.15	2	81 ± 2	125 ± 4	1.25 ± 0.01	1.26	0.97 ± 0.00	0.98

Table S6: Phase equilibria for H_2-CO_2-NaCl brine systems from CFCMC simulations[24–26] for various pressures, temperatures, and NaCl concentrations (c_{NaCl} in units of mol NaCl/kg H_2O). Solubilities of H_2 and CO_2 in the liquid-rich phase are denoted by x_{H_2} and x_{CO_2}, respectively, and the water content in the gas-rich phase is denoted by y_{H_2O}.

p [MPa]	T [K]	c_{NaCl}	x_{H_2} / [10^{-4}]	x_{CO_2} / [10^{-4}]	y_{H_2} / [10^{-4}]	y_{H_2O} / [10^{-4}]
5	323.15	0	49 ± 9	4 ± 1	5029 ± 8	33 ± 4
5	348.15	0	43 ± 3	3 ± 0	4997 ± 4	86 ± 8
5	373.15	0	34 ± 2	4 ± 0	4906 ± 3	246 ± 9
5	398.15	0	33 ± 1	5 ± 0	4726 ± 7	601 ± 13
5	423.15	0	34 ± 2	6 ± 1	4394 ± 5	1258 ± 9
10	323.15	0	89 ± 7	7 ± 0	5074 ± 8	20 ± 3
10	348.15	0	73 ± 7	9 ± 1	5038 ± 10	54 ± 8
10	373.15	0	63 ± 2	9 ± 3	4983 ± 8	143 ± 11
10	398.15	0	60 ± 4	11 ± 1	4882 ± 13	333 ± 21
10	423.15	0	64 ± 2	13 ± 1	4702 ± 10	690 ± 23
30	323.15	0	138 ± 8	23 ± 4	5115 ± 9	13 ± 5
30	348.15	0	135 ± 10	28 ± 4	5097 ± 10	31 ± 4
30	373.15	0	133 ± 5	30 ± 5	5072 ± 10	70 ± 9
30	398.15	0	132 ± 8	35 ± 2	5021 ± 7	158 ± 3
30	423.15	0	140 ± 3	44 ± 4	4942 ± 10	309 ± 19
50	323.15	0	143 ± 8	49 ± 2	5092 ± 9	16 ± 2
50	348.15	0	149 ± 3	46 ± 2	5093 ± 6	32 ± 5
50	373.15	0	154 ± 7	51 ± 2	5078 ± 13	62 ± 11
50	398.15	0	164 ± 4	59 ± 4	5044 ± 5	134 ± 8
50	423.15	0	188 ± 5	73 ± 4	4996 ± 6	249 ± 7

Continued on next page

p [MPa]	T [K]	c_{NaCl}	x_{H_2} / [10^{-4}]	x_{CO_2} / [10^{-4}]	y_{H_2} / [10^{-4}]	y_{H_2O} / [10^{-4}]
5	323.15	1	42 ± 4	3 ± 1	5023 ± 4	32 ± 5
5	348.15	1	33 ± 4	3 ± 1	4982 ± 6	96 ± 7
5	373.15	1	29 ± 3	4 ± 1	4905 ± 2	237 ± 5
5	398.15	1	27 ± 1	5 ± 1	4736 ± 6	570 ± 12
5	423.15	1	26 ± 0	5 ± 1	4417 ± 9	1201 ± 16
10	323.15	1	69 ± 14	8 ± 3	5053 ± 19	20 ± 3
10	348.15	1	60 ± 6	7 ± 1	5029 ± 7	49 ± 6
10	373.15	1	53 ± 4	8 ± 1	4974 ± 6	142 ± 7
10	398.15	1	50 ± 3	11 ± 2	4882 ± 5	312 ± 9
10	423.15	1	49 ± 2	11 ± 1	4704 ± 5	662 ± 9
30	323.15	1	112 ± 13	22 ± 2	5087 ± 15	13 ± 4
30	348.15	1	98 ± 3	24 ± 5	5061 ± 4	32 ± 3
30	373.15	1	102 ± 4	29 ± 4	5041 ± 6	67 ± 5
30	398.15	1	105 ± 4	32 ± 3	4998 ± 6	152 ± 6
30	423.15	1	112 ± 7	38 ± 2	4926 ± 8	297 ± 4
50	323.15	1	120 ± 6	37 ± 6	5080 ± 9	13 ± 3
50	348.15	1	116 ± 4	39 ± 4	5069 ± 2	25 ± 3
50	373.15	1	121 ± 5	46 ± 5	5049 ± 10	60 ± 4
50	398.15	1	141 ± 7	53 ± 4	5029 ± 6	127 ± 10
50	423.15	1	148 ± 5	60 ± 6	4976 ± 12	230 ± 7
5	323.15	2	32 ± 2	3 ± 2	5015 ± 4	28 ± 4
5	348.15	2	28 ± 3	3 ± 1	4981 ± 2	87 ± 6
5	373.15	2	25 ± 2	3 ± 1	4907 ± 8	229 ± 16
5	398.15	2	22 ± 1	4 ± 1	4738 ± 3	558 ± 4

Continued on next page

p [MPa]	T [K]	c_{NaCl}	x_{H_2} / [10^{-4}]	x_{CO_2} / [10^{-4}]	y_{H_2} / [10^{-4}]	$y_{\text{H}_2\text{O}}$ / [10^{-4}]
5	423.15	2	22 ± 1	4 ± 1	4436 ± 12	1156 ± 23
10	323.15	2	51 ± 3	6 ± 2	5040 ± 3	14 ± 5
10	348.15	2	41 ± 6	7 ± 1	5008 ± 6	54 ± 7
10	373.15	2	45 ± 5	6 ± 1	4978 ± 11	122 ± 14
10	398.15	2	40 ± 3	7 ± 1	4878 ± 7	308 ± 10
10	423.15	2	39 ± 4	10 ± 1	4708 ± 6	638 ± 8
30	323.15	2	90 ± 8	19 ± 5	5067 ± 11	13 ± 4
30	348.15	2	84 ± 8	18 ± 3	5053 ± 10	28 ± 7
30	373.15	2	86 ± 9	20 ± 2	5032 ± 8	70 ± 4
30	398.15	2	89 ± 7	26 ± 3	4991 ± 8	146 ± 8
30	423.15	2	90 ± 5	32 ± 3	4913 ± 8	291 ± 10
50	323.15	2	99 ± 6	35 ± 7	5062 ± 13	11 ± 5
50	348.15	2	100 ± 5	33 ± 5	5053 ± 8	33 ± 4
50	373.15	2	98 ± 4	37 ± 1	5034 ± 6	57 ± 9
50	398.15	2	108 ± 9	45 ± 3	5003 ± 13	125 ± 10
50	423.15	2	119 ± 6	52 ± 2	4958 ± 14	220 ± 17

Table S7: The fugacity coefficients (ϕ) of all species in the gas-rich phase obtained from CFCMC simulations[24–26] are compared to the corresponding values from REFPROP (RFP). Fugacity coefficients from REFPROP for H_2, CO_2 and H_2O at given pressure, and temperature are obtained at the gas-phase composition obtained from CFCMC simulations.

p	T	c_{NaCl}	ϕ_{H_2}		ϕ_{CO_2}		ϕ_{H_2O}	
[MPa]	[K]		CFCMC	RFP	CFCMC	RFP	CFCMC	RFP
5	323.15	0	0.87 ± 0.01	0.86	1.06 ± 0.01	1.07	0.83 ± 0.01	0.86
5	348.15	0	0.89 ± 0.01	0.89	1.04 ± 0.01	1.06	0.84 ± 0.01	0.89
5	373.15	0	0.92 ± 0.01	0.91	1.04 ± 0.01	1.06	0.83 ± 0.01	0.90
5	398.15	0	0.93 ± 0.01	0.93	1.03 ± 0.01	1.06	0.81 ± 0.01	0.91
5	423.15	0	0.95 ± 0.01	0.94	1.04 ± 0.01	1.06	0.78 ± 0.01	0.91
10	323.15	0	0.77 ± 0.01	0.75	1.13 ± 0.01	1.16	0.71 ± 0.01	0.75
10	348.15	0	0.81 ± 0.01	0.80	1.11 ± 0.01	1.13	0.75 ± 0.01	0.79
10	373.15	0	0.85 ± 0.01	0.84	1.09 ± 0.01	1.12	0.76 ± 0.01	0.83
10	398.15	0	0.88 ± 0.01	0.87	1.08 ± 0.01	1.11	0.75 ± 0.01	0.85
10	423.15	0	0.91 ± 0.01	0.89	1.08 ± 0.01	1.10	0.73 ± 0.01	0.86
30	323.15	0	0.52 ± 0.01	0.49	1.46 ± 0.02	1.50	0.41 ± 0.01	0.48
30	348.15	0	0.61 ± 0.01	0.58	1.38 ± 0.02	1.42	0.50 ± 0.01	0.56
30	373.15	0	0.70 ± 0.01	0.66	1.33 ± 0.01	1.36	0.56 ± 0.01	0.63
30	398.15	0	0.76 ± 0.01	0.72	1.29 ± 0.01	1.32	0.59 ± 0.01	0.68
30	423.15	0	0.81 ± 0.01	0.77	1.26 ± 0.01	1.29	0.59 ± 0.01	0.71
50	323.15	0	0.44 ± 0.01	0.42	1.77 ± 0.03	1.81	0.29 ± 0.01	0.38
50	348.15	0	0.55 ± 0.01	0.52	1.65 ± 0.01	1.69	0.38 ± 0.01	0.47
50	373.15	0	0.64 ± 0.01	0.60	1.56 ± 0.01	1.60	0.44 ± 0.01	0.54
50	398.15	0	0.72 ± 0.01	0.68	1.51 ± 0.01	1.54	0.49 ± 0.01	0.60

Continued on next page

p [MPa]	T [K]	c_{NaCl}	ϕ_{H_2} CFCMC	ϕ_{H_2} RFP	ϕ_{CO_2} CFCMC	ϕ_{CO_2} RFP	ϕ_{H_2O} CFCMC	ϕ_{H_2O} RFP
50	423.15	0	0.79 ± 0.01	0.74	1.45 ± 0.01	1.48	0.51 ± 0.01	0.65
5	323.15	1	0.87 ± 0.01	0.86	1.06 ± 0.01	1.07	0.83 ± 0.01	0.86
5	348.15	1	0.89 ± 0.01	0.89	1.04 ± 0.01	1.06	0.84 ± 0.01	0.89
5	373.15	1	0.91 ± 0.01	0.91	1.03 ± 0.01	1.06	0.83 ± 0.01	0.90
5	398.15	1	0.93 ± 0.01	0.93	1.04 ± 0.01	1.05	0.82 ± 0.01	0.91
5	423.15	1	0.95 ± 0.01	0.94	1.04 ± 0.01	1.06	0.78 ± 0.01	0.91
10	323.15	1	0.76 ± 0.01	0.75	1.12 ± 0.02	1.16	0.70 ± 0.01	0.75
10	348.15	1	0.82 ± 0.01	0.80	1.11 ± 0.01	1.13	0.75 ± 0.01	0.79
10	373.15	1	0.86 ± 0.01	0.84	1.11 ± 0.01	1.12	0.77 ± 0.01	0.83
10	398.15	1	0.88 ± 0.01	0.87	1.08 ± 0.01	1.11	0.76 ± 0.01	0.85
10	423.15	1	0.91 ± 0.01	0.89	1.09 ± 0.01	1.10	0.74 ± 0.01	0.86
30	323.15	1	0.52 ± 0.01	0.49	1.47 ± 0.01	1.51	0.41 ± 0.01	0.47
30	348.15	1	0.61 ± 0.01	0.58	1.39 ± 0.01	1.43	0.50 ± 0.01	0.56
30	373.15	1	0.69 ± 0.01	0.65	1.33 ± 0.01	1.37	0.56 ± 0.01	0.62
30	398.15	1	0.76 ± 0.01	0.72	1.29 ± 0.01	1.33	0.59 ± 0.01	0.68
30	423.15	1	0.82 ± 0.01	0.77	1.27 ± 0.01	1.30	0.60 ± 0.01	0.71
50	323.15	1	0.44 ± 0.01	0.42	1.78 ± 0.01	1.81	0.29 ± 0.01	0.38
50	348.15	1	0.54 ± 0.01	0.51	1.66 ± 0.01	1.70	0.38 ± 0.01	0.46
50	373.15	1	0.64 ± 0.01	0.60	1.57 ± 0.01	1.61	0.45 ± 0.01	0.54
50	398.15	1	0.72 ± 0.01	0.68	1.50 ± 0.01	1.54	0.49 ± 0.01	0.60
50	423.15	1	0.79 ± 0.01	0.74	1.45 ± 0.01	1.49	0.51 ± 0.01	0.65
5	323.15	2	0.86 ± 0.01	0.86	1.05 ± 0.01	1.07	0.82 ± 0.01	0.86
5	348.15	2	0.89 ± 0.01	0.89	1.05 ± 0.01	1.06	0.84 ± 0.01	0.89

Continued on next page

p [MPa]	T [K]	c_{NaCl}	ϕ_{H_2} CFCMC	ϕ_{H_2} RFP	ϕ_{CO_2} CFCMC	ϕ_{CO_2} RFP	ϕ_{H_2O} CFCMC	ϕ_{H_2O} RFP
5	373.15	2	0.91 ± 0.01	0.91	1.04 ± 0.01	1.06	0.84 ± 0.01	0.90
5	398.15	2	0.93 ± 0.01	0.93	1.04 ± 0.01	1.05	0.82 ± 0.01	0.91
5	423.15	2	0.95 ± 0.01	0.94	1.04 ± 0.01	1.06	0.79 ± 0.01	0.91
10	323.15	2	0.76 ± 0.01	0.75	1.13 ± 0.01	1.16	0.71 ± 0.01	0.75
10	348.15	2	0.82 ± 0.01	0.80	1.12 ± 0.01	1.13	0.76 ± 0.01	0.79
10	373.15	2	0.85 ± 0.01	0.84	1.10 ± 0.01	1.12	0.77 ± 0.01	0.83
10	398.15	2	0.88 ± 0.01	0.87	1.09 ± 0.01	1.11	0.76 ± 0.01	0.85
10	423.15	2	0.91 ± 0.01	0.89	1.08 ± 0.01	1.10	0.74 ± 0.01	0.86
30	323.15	2	0.52 ± 0.01	0.49	1.48 ± 0.01	1.51	0.41 ± 0.01	0.47
30	348.15	2	0.62 ± 0.01	0.58	1.39 ± 0.01	1.43	0.50 ± 0.01	0.56
30	373.15	2	0.69 ± 0.01	0.65	1.33 ± 0.02	1.37	0.56 ± 0.01	0.62
30	398.15	2	0.76 ± 0.01	0.72	1.29 ± 0.01	1.33	0.60 ± 0.01	0.68
30	423.15	2	0.81 ± 0.01	0.77	1.26 ± 0.01	1.30	0.60 ± 0.01	0.71
50	323.15	2	0.44 ± 0.01	0.42	1.77 ± 0.01	1.82	0.29 ± 0.01	0.38
50	348.15	2	0.54 ± 0.01	0.51	1.66 ± 0.01	1.70	0.37 ± 0.01	0.46
50	373.15	2	0.63 ± 0.01	0.60	1.57 ± 0.01	1.61	0.45 ± 0.01	0.54
50	398.15	2	0.72 ± 0.01	0.67	1.50 ± 0.01	1.54	0.49 ± 0.01	0.60
50	423.15	2	0.78 ± 0.01	0.74	1.45 ± 0.01	1.49	0.52 ± 0.01	0.65

Bibliography

S1 Martyna, G. J.; Klein, M. L.; Tuckerman, M. Nosé-Hoover chains: The canonical ensemble via continuous dynamics. *J. Chem. Phys.* **1992**, *97*, 2635.

S2 Kamberaj, H.; Low, R. J.; Neal, M. P. Time reversible and symplectic integrators for molecular dynamics simulations of rigid molecules. *J. Chem. Phys.* **2005**, *122*, 224114.

S3 Lemmon, E. W.; Huber, M. L.; McLinden, M. O. NIST reference fluid thermodynamic and transport properties–REFPROP. *NIST standard reference database* **2002**, *23*.

S4 Jamali, S. H.; Wolff, L.; Becker, T. M.; De Groen, M.; Ramdin, M.; Hartkamp, R.; Bardow, A.; Vlugt, T. J. H.; Moultos, O. A. OCTP: A tool for on-the-fly calculation of transport properties of fluids with the order-n algorithm in LAMMPS. *J. Chem. Inf. Model.* **2019**, *59*, 1290–1294.

S5 Frenkel, D.; Smit, B. *Understanding Molecular Simulation, From Algorithms to Applications*, 3rd ed.; Elsevier Science, Oxford, 2023.

S6 Allen, M. P.; Tildesley, D. *Computer Simulation of Liquids*, 2nd ed.; Oxford University Press, Oxford, 2017.

S7 Yeh, I. C.; Hummer, G. System-size dependence of diffusion coefficients and viscosities from molecular dynamics simulations with periodic boundary conditions. *J. Phys. Chem. B* **2004**, *108*, 15873.

S8 Celebi, A. T.; Jamali, S. H.; Bardow, A.; Vlugt, T. J. H.; Moultos, O. A. Finite-size effects of diffusion coefficients computed from molecular dynamics: a review of what we have learned so far. *Mol. Sim.* **2021**, *47*, 831–845.

S9 Moultos, O. A.; Zhang, Y.; Tsimpanogiannis, I. N.; Economou, I. G.; Maginn, E. J. System-Size Corrections for the Self-Diffusion Coefficients Calculated from Molecular

Dynamics Simulations: The Case of CO_2, n-Alkanes, and poly(ethylene glycol) dimenthyl ethers. *J. Chem. Phys.* **2016**, *145*, 074109.

S10 Cussler, E. L. *Diffusion: Mass transfer in fluid systems*, 3rd ed.; Cambridge University press, Cambridge, 2009.

S11 Barrat, J.-L.; Hansen, J.-P. *Basic Concepts for Simple and Complex Liquids*, 1st ed.; Cambridge University Press, Cambridge, 2003.

S12 Taylor, R.; Kooijman, H. A. Composition derivatives of activity coefficient models (for the estimation of thermodynamic factors in diffusion). *Chem. Eng. Commun.* **1991**, *102*, 87–106.

S13 Taylor, R.; Krishna, R. *Multicomponent Mass Transfer*, 1st ed.; John Wiley & Sons, New York, 1993.

S14 Liu, X.; Schnell, S. K.; Simon, J.-M.; Bedeaux, D.; Kjelstrup, S.; Bardow, A.; Vlugt, T. J. H. Fick Diffusion Coefficients of Liquid Mixtures Directly Obtained from Equilibrium Molecular Dynamics. *J. Phys. Chem. B* **2011**, *115*, 12921.

S15 Liu, X.; Martín-Calvo, A.; McGarrity, E.; Schnell, S. K.; Calero, S.; Simon, J.-M.; Bedeaux, D.; Kjelstrup, S.; Bardow, A.; Vlugt, T. J. H. Fick Diffusion Coefficients in Ternary Liquid Systems from Equilibrium Molecular Simulations. *Ind. Eng. Chem. Res.* **2012**, *51*, 10247.

S16 Liu, X.; Schnell, S. K.; Simon, J.-M.; Krüger, P.; Bedeaux, D.; Kjelstrup, S.; Bardow, A.; Vlugt, T. J. H. Diffusion Coefficients from Molecular Dynamics Simulations in Binary and Ternary Mixtures. *Int. J. Thermophys.* **2013**, *34*, 1169.

S17 Jamali, S. H.; Wolff, L.; Becker, T. M.; Bardow, A.; Vlugt, T. J. H.; Moultos, O. A.

Finite-Size Effects of Binary Mutual Diffusion Coefficients from Molecular Dynamics. *J. Chem. Theory Comput.* **2018**, *14*, 2667.

S18 Potoff, J. J.; Siepmann, J. I. Vapor-liquid Equilibria of Mixtures Containing Alkanes, Carbon Dioxide and Nitrogen. *AIChE J.* **2001**, *47*, 1676.

S19 Marx, D.; Nielaba, P. Path-integral Monte Carlo techniques for rotational motion in two dimensions: Quenched, annealed, and no-spin quantum-statistical averages. *Phys. Rev. A* **1992**, *45*, 8968–8971.

S20 Rahbari, A.; Brenkman, J.; Hens, R.; Ramdin, M.; van den Broeke, L. J. P.; Schoon, R.; Henkes, R.; Moultos, O. A.; Vlugt, T. J. H. Solubility of Water in Hydrogen at High Pressures: A Molecular Simulation Study. *J. Chem. Eng. Data* **2019**, *64*, 4103.

S21 Rahbari, A.; Garcia-Navarro, J. C.; Ramdin, M.; van den Broeke, L. J. P.; Moultos, O. A.; Dubbeldam, D.; Vlugt, T. J. H. Effect of water content on thermodynamic properties of compressed hydrogen. *J. Chem. Eng. Data* **2021**, *66*, 2071.

S22 Zeron, I. M.; Abascal, J. L. F.; Vega, C. A force field of Li^+, Na^+, K^+, Mg^{2+}, Ca^{2+}, Cl^-, and SO_4^{2-} in aqueous solution based on the TIP4P/2005 water model and scaled charges for the ions. *J. Chem. Phys.* **2019**, *151*.

S23 van Rooijen, W. A.; Habibi, P.; Xu, K.; Dey, P.; Vlugt, T. J. H.; Hajibeygi, H.; Moultos, O. A. Interfacial Tensions, Solubilities, and Transport Properties of the H_2/H_2O/NaCl System: A Molecular Simulation Study. *J. Chem. Eng. Data* **2024**, *69*, 307–319.

S24 Hens, R.; Rahbari, A.; Caro-Ortiz, S.; Dawass, N.; Erdős, M.; Poursaeidesfahani, A.; Salehi, H. S.; Celebi, A. T.; Ramdin, M.; Moultos, O. A.; Dubbeldam, D.; Vlugt, T. J. H. Brick-CFCMC: Open source software for Monte Carlo simulations of phase and

reaction equilibria using the Continuous Fractional Component method. *J. Chem. Inf. Model.* **2020**, *60*, 2678–2682.

S25 Polat, H. M.; Salehi, H. S.; Hens, R.; Wasik, D. O.; Rahbari, A.; De Meyer, F.; Houriez, C.; Coquelet, C.; Calero, S.; Dubbeldam, D.; ; Moultos, O. A.; Vlugt, T. J. H. New features of the open source Monte Carlo software Brick-CFCMC: Thermodynamic integration and hybrid trial moves. *J. Chem. Inf. Model.* **2021**, *61*, 3752–3757.

S26 Rahbari, A.; Hens, R.; Ramdin, M.; Moultos, O. A.; Dubbeldam, D.; Vlugt, T. J. H. Recent advances in the continuous fractional component Monte Carlo methodology. *Mol. Sim.* **2021**, *47*, 804–823.
